# An integrative multiomics random forest framework for robust biomarker discovery

**DOI:** 10.1093/gigascience/giaf148

**Published:** 2025-12-09

**Authors:** Wei Zhang, Hanchen Huang, Lily Wang, Brian D Lehmann, X Steven Chen

**Affiliations:** Division of Biostatistics and Bioinformatics, Department of Public Health Sciences, University of Miami Miller School of Medicine, Miami, FL 33136, USA; Division of Biostatistics and Bioinformatics, Department of Public Health Sciences, University of Miami Miller School of Medicine, Miami, FL 33136, USA; Sylvester Comprehensive Cancer Center, University of Miami Miller School of Medicine, Miami, FL 33136, USA; Division of Biostatistics and Bioinformatics, Department of Public Health Sciences, University of Miami Miller School of Medicine, Miami, FL 33136, USA; Sylvester Comprehensive Cancer Center, University of Miami Miller School of Medicine, Miami, FL 33136, USA; Dr. John T. Macdonald Foundation Department of Human Genetics, University of Miami Miller School of Medicine, Miami, FL 33136, USA; John P. Hussman Institute for Human Genomics, University of Miami Miller School of Medicine, Miami, FL 33136, USA; Division of Hematology and Oncology, Department of Medicine, Vanderbilt University Medical Center, Nashville, TN 37232, USA; Division of Biostatistics and Bioinformatics, Department of Public Health Sciences, University of Miami Miller School of Medicine, Miami, FL 33136, USA; Sylvester Comprehensive Cancer Center, University of Miami Miller School of Medicine, Miami, FL 33136, USA

**Keywords:** Multi-omics integration, Multivariate random forest, Machine learning, Biomarker discovery

## Abstract

**Background:**

High-throughput technologies now produce a wide array of omics data, from genomic and transcriptomic profiles to epigenomic and proteomic measurements. Integrating multiple omics layers measured on the same samples can reveal cross-layer molecular hubs that single-layer analyses miss. However, many existing integrative methods rely on linear assumptions or univariate feature importance, limiting their ability to capture nonlinear and interaction-driven dependencies across data modalities.

**Results:**

We present an unsupervised, multivariate random forest (MRF) framework with an inverse minimal depth (IMD) importance to prioritize shared biomarkers across omics. In each forest, one layer serves as a multivariate response and the other as predictors; IMD summarizes how early a predictor (or response maximal splitting response variable) appears across trees, yielding interpretable, cross-layer feature rankings. We provide two IMD-based selection strategies and introduce an optional IMD power transform to enhance sensitivity to interaction signals. In extensive simulations spanning linear, nonlinear, and interaction regimes, our method matches sparse partial least squares/canonical correlation analysis under linear settings and outperforms them as nonlinearity increases, while adapted univariate ensemble learners (random forest, gradient boosting machine, XGBoost) underperform in the multivariate, unsupervised context. Applied to breast invasive carcinoma and colon adenocarcinoma in The Cancer Genome Atlas (TCGA), MRF-IMD identifies genes, CpGs, and microRNAs enriched for cancer-relevant pathways and yields more robust survival stratification than linear integrators with matched model sizes. In a TCGA pan-cancer analysis, MRF-IMD features achieve a higher Adjusted Rand Index than alternatives and recover coherent tumor-type clusters; in the Alzheimer’s Disease Neuroimaging Initiative (ADNI), the integrative signature improves dementia progression stratification over a published methylation risk score.

**Conclusions:**

MRF-IMD provides a scalable and interpretable framework for multiomics integration that reliably identifies cross-layer biomarkers when nonlinear and interaction-driven dependencies are present. This approach advances robust biomarker discovery beyond the limits of linear integrative methods.

## Introduction

Recent technological advances in high-throughput sequencing, mass spectrometry, and imaging have led to a surge in multiomics data that span the genome, epigenome, transcriptome, proteome, and metabolome. However, each type of data alone captures only a slice of disease biology. Integrating these diverse data sources can provide a more comprehensive picture of complex biological systems than analyzing any single omics layer alone. Multiomics analysis has been implemented in many studies for biomarker discovery, disease subtyping, and disease insights [1]. A key goal in multiomics integration is to extract “shared” biomarkers from multiple data, that is, to identify molecular features that are biologically relevant consistently across different omics type. These biomarkers typically indicate robust, system-level regulatory mechanisms that single-omics analyses may miss [1, 2]. Furthermore, integrating complementary data sources reduces noise and mitigates biological heterogeneity, enhancing the precision and clinical relevance of patient stratification and prognosis [3]. In general, multiomics approaches tend to yield more reliable biomarkers and disease signatures than single-modality analyses, as demonstrated in recent studies: methods like DIABLO, which, based on the sparse partial least squares (SPLS) method, seek common information across data types by selecting subsets of features that jointly capture variance in each dataset [4]. When done effectively, integration can highlight shared molecular features across different data types, offering new insights into disease mechanisms, patient stratification, and potential biomarkers for clinical applications [4–7].

Despite the promise of multiomics integration, extracting shared signals across heterogeneous datasets remains challenging. Traditional penalized integration methods, such as SPLS [4, 8, 9] and canonical correlation analysis (CCA) [10–12], focus largely on linear relationships. Although widely used, these approaches can struggle in high-dimensional settings, are prone to overfitting, and may fail to capture nonlinear interactions. For example, in “XOR” simulation, where 2 variables interact multiplicatively to determine the response, both SPLS or CCA, which optimize linear covariance/correlation, tend to yield near-random feature rankings and fail to recover the interacting pair (as we also show in our simulations). Nonlinear extensions, including kernel CCA [13, 14], help address some of these issues but often face scalability and interpretability limitations, making them less suitable for many practical scenarios.

Ensemble learning techniques, particularly random forests, are valued for their robustness, ability to model nonlinearities, and relative resilience to overfitting [15]. Extending random forests to handle multiple response variables leads to multivariate random forests (MRFs) [16], which are well positioned to tackle complex multiomics data. However, applications of MRFs to multiomics integration have been limited, leaving an opportunity to develop methods that exploit the strengths of this approach for biomarker discovery and feature selection.

In this study, we introduce a new MRF-based framework that employs the inverse minimal depth (IMD) metric for unsupervised variable selection across multiple omics datasets. We model the relationships between each pair of 2 omics by assigning one omics to the response space and the other omics to the feature space in an ensemble of decision trees. After fitting the forest, we compute the IMD to quantify feature importance and identify key variables shared across different data layers. We then extend our framework from pairwise (2-omics) integration to a comprehensive multiomics approach by modeling different layer pairs guided by prior knowledge or precomputed interrelationships. This strategy naturally reduces the risk of selecting noise variables and helps focus on those with consistent impact across datasets. To show that our method can effectively capture shared biomarkers in complex datasets, we benchmarked it against established integration approaches, including SPLS, CCA, and several nonlinear ensemble methods such as gradient boosting machine (GBM) and XGBoost through multiple simulations. We found that methods like SPLS and CCA are not stable in capturing the important features when data types are nonlinear or contains interaction settings. Other nonlinear methods are easily failed as they are not designed for multivariate analysis. Moreover, we validated our framework using several clinical cohorts, including breast invasive carcinoma (BRCA) and colon adenocarcinoma (COAD) in The Cancer Genome Atlas (TCGA), demonstrating superior ability to uncover biologically relevant pathways and to stratify patients by prognostic outcome compared to traditional integration methods such as SPLS and CCA. We further applied our approach to the TCGA pan-cancer (PANCAN) and Alzheimer’s Disease Neuroimaging Initiative (ADNI) datasets, identifying biomarker panels tied to key biological pathways that show promise for enhancing molecular subtyping of PAN cancer, and prognosis of dementia onset.

In summary, our MRF-IMD framework provides a robust and flexible solution for multiomics integration. By embracing nonlinear relationships, addressing high-dimensionality, and maintaining interpretability, this approach has the potential to advance biomarker discovery and contribute valuable insights to complex biological and clinical problems.

## Material and Methods

### Maximal splitting response variable

Consider 2 datasets ${{{{\bf X}}}_{n \times p}}$ and ${{{{\bf Y}}}_{n \times q}}$, where *n* is the number of samples, and *p* and *q* represent the number of features of ${\boldsymbol{X}}$ and ${\boldsymbol{Y}}$, respectively. Our goal is to integrate these datasets using an MRF approach. In this framework, we use a splitting rule that considers all response variables together, rather than handling them individually. We begin with a splitting rule introduced by Tang and Ishwaran [17] that extends the traditional univariate splitting criterion to a multivariate setting. This rule extends univariate splitting by summing the splitting criterion across all response outcomes ${{Y}_{.j}}$. The splitting criterion for node *t* is defined as:


(1) \begin{eqnarray*}
{{{\boldsymbol{G}}}_{\boldsymbol{q}}}\left( {{\boldsymbol{s}},{\boldsymbol{t}}} \right) &=& \mathop \sum \limits_{{\boldsymbol{j}} = 1}^{\boldsymbol{q}} \left\{ {\mathop \sum \limits_{{\boldsymbol{i}} \in {{{\boldsymbol{t}}}_{\boldsymbol{L}}}} {{{\left( {{{{\boldsymbol{Y}}}_{{\boldsymbol{ij}}}} - {\bar{Y}_{{{{\boldsymbol{t}}}_{{{{\boldsymbol{L}}}_{\boldsymbol{j}}}}}}}} \right)}}^2} + \mathop \sum \limits_{{\boldsymbol{i}} \in {{{\boldsymbol{t}}}_{\boldsymbol{R}}}} {{{\left( {{{{\boldsymbol{Y}}}_{{\boldsymbol{ij}}}} - {\bar{Y}_{{{{\boldsymbol{t}}}_{{{{\boldsymbol{R}}}_{\boldsymbol{j}}}}}}}} \right)}}^2}} \right\} \\
&=& \mathop \sum \limits_{{\boldsymbol{j}} = 1}^{\boldsymbol{q}} {{{\boldsymbol{G}}}_{\boldsymbol{j}}}\left( {{\boldsymbol{s}},{\boldsymbol{t}}} \right)
\end{eqnarray*}

where ${{t}_{{{L}_j}}}$ and ${{t}_{{{R}_j}}}$ represent the left and right daughter nodes for ${{j}_{{\mathrm{th}}}}$ response coordinate, and ${\bar{Y}_{{{t}_{{{L}_j}}}}}$ and ${\bar{Y}_{{{t}_{{{R}_j}}}}}$ are the sample means in ${{t}_{{{L}_j}}}$ and ${{t}_{{{R}_j}}}$. To determine the best split, we minimize ${{G}_q}( {s,t} )$, ensuring all response variables ${{Y}_{.1}},\cdots ,{{Y}_{.q}}$ are measured on the same scale by standardizing them to a 0–1 scale: $Y_{ij}^{\mathrm{*}} = \frac{{\sqrt n ( {{{Y}_{ij}} - {\bar{Y}_{{{t}_j}}}} )}}{{\sqrt {\mathop \sum \nolimits_{i\in t} {{{( {{{Y}_{ij}} - {\bar{Y}_{{{t}_j}}}} )}}^2}} }}$, where


\begin{eqnarray*}
\frac{1}{n}\mathop \sum \limits_{i\in t} Y_{ij}^{\mathrm{*}} = 0,\frac{1}{n}\mathop \sum \limits_{i\in t} Y_{ij}^{{\mathrm{*}}2} = 1,\quad {\mathrm{for\ }}1 \le j \le q
\end{eqnarray*}

This standardization ensures that the contributions of all outcomes are comparable, preventing any single outcome from dominating the splitting process. After simplifying the expression, the minimization of ${{G}_q}( {s,t} )$ becomes equivalent to maximizing:


(2) \begin{eqnarray*}
G_q^{\mathrm{*}}\left( {s,t} \right) = \mathop \sum \limits_{j = 1}^q \left\{ {\frac{1}{{{{n}_{{{t}_L}}}}}{{{\left( {\mathop \sum \limits_{i\in {{t}_L}} Y_{ij}^{\mathrm{*}}} \right)}}^2} + \frac{1}{{{{n}_{{{t}_R}}}}}{{{\left( {\mathop \sum \limits_{i\in {{t}_R}} Y_{ij}^{\mathrm{*}}} \right)}}^2}} \right\}
\end{eqnarray*}

In the case of 2-omics data, we treat one dataset as the response and the other as the predictor and apply the MRF model using these splitting rules.

Using the above framework, we now focus on 2-omics data. For each response variable ${{Y}_j}$, we define the splitting statistic as:


(3) \begin{eqnarray*}
{{G}_j} = \frac{1}{{{{n}_{{{t}_L}}}}}{{\left( {\mathop \sum \limits_{i\in {{t}_L}} Y_{{\mathrm{ij}}}^{\mathrm{*}}} \right)}^2} + \frac{1}{{{{n}_{{{t}_R}}}}}{{\left( {\mathop \sum \limits_{i\in {{t}_R}} Y_{{\mathrm{ij}}}^{\mathrm{*}}} \right)}^2}
\end{eqnarray*}

This statistic quantifies how well a split separates the values of ${{Y}_j}$ in the response ${{\bf Y}}$ across the left and right daughter nodes. For each node split, we identify the maximal splitting response variable (MSRV) as the response variable that maximizes the multivariate splitting rule $G_q^{\mathrm{*}}( {s,t} )$, meaning it has the largest contribution to the split. The MSRV represents the variable most associated with the predictors at that particular node. A detailed explanation and study of MSRV is described in Supplementary Note 1.

### Inverse minimal depth

#### Minimal depth

**Figure 1:**
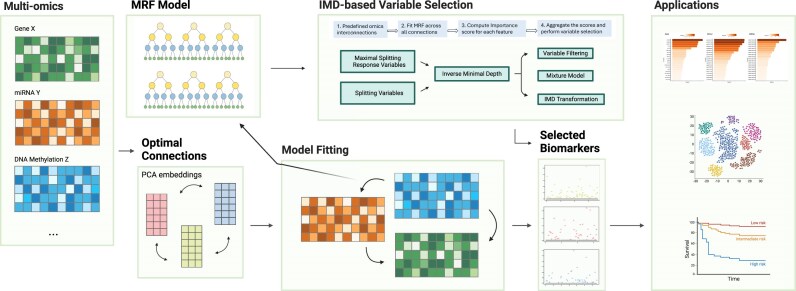
Workflow of the MRF-IMD framework. The overall workflow of our integrative multiomics biomarker discovery pipeline using the MRF-IMD strategy.

Minimal depth, introduced by Ishwaran et al. [18, 19], is a variable selection method that efficiently ranks strong variables higher than weak ones. The minimal depth of a variable refers to the shortest distance from the root of a decision tree to the node where the variable appears. Let ${{D}_v}$ denote the minimal depth of a variable *v* and $D( T )$ represent the depth of a tree *T*. It has been proved that the distribution of ${{D}_v}$ is:


(4) \begin{eqnarray*}
\begin{array}{*{20}{c}} {\mathbb{P}\left\{ {{{D}_v} = d\mid \mathcal{l}_0^{\mathrm{*}},\cdots,\mathcal{l}_{D\left( T \right) - 1}^{\mathrm{*}}} \right\} = \left[ {\mathop \prod \limits_{j = 0}^{d - 1} {{{\left( {1 - {{\pi }_{v,j}}{{\theta }_{v,j}}} \right)}}^{\mathcal{l}_j^{\mathrm{*}}}}} \right]}\\
{\times \,\left[ {1 - {{{\left( {1 - {{\pi }_{v,d}}{{\theta }_{v,d}}} \right)}}^{\mathcal{l}_d^{\mathrm{*}}}}} \right], 0 \le d \le D\left( T \right) - 1} \end{array}
\end{eqnarray*}

where ${{\pi }_{v,j}}$ is the probability of *v* selected as a candidate variable for splitting of node *t* at depth *j*, ${{\theta }_{v,j}}$ is the probability of *v* splits a node *t* at depth *j*, and $\mathcal{l}_j^{\mathrm{*}}$ is the number of nodes at depth *j*. Note that if the tree is a balanced tree, $\mathcal{l}_j^{\mathrm{*}} = {{2}^j}$ at depth *j*. For example, in Fig. 1, the root node variable ${{X}_{20}}$ is assigned a minimal depth ${{D}_{{{X}_{20}}}} = 0$. The left daughter node of ${{X}_{20}}$, ${{X}_6}$ has a minimal depth of ${{D}_{{{X}_6}}} = 1$. The original study on minimal depth proposed 2 strategies for identifying strong variables. The first strategy uses the mean minimal depth under the null hypothesis that a variable *v* is a weak variable. Given *v* is a weak variable, the distribution of the minimal depth of *v* is


(5) \begin{eqnarray*}
\mathbb{P}\left\{ {{{D}_v} = d\mid v\,\,{\mathrm{is\ a\ weak\ variable}}} \right\} \approx {{\left( {1 - \frac{1}{p}} \right)}^{{{L}_d}}}\left[ {1 - {{{\left( {1 - \frac{1}{p}} \right)}}^{\mathcal{{l}_d}}}} \right]\\
\end{eqnarray*}

where ${{L}_d} = 1 + 2 + \cdots + {{2}^{d - 1}} = \mathcal{{l}_d} - 1$, and *p* is the number of features in the dataset. The threshold works well when *n* is large and is more computationally efficient than permutation-based variable importance (VIMP) and jointly VIMP in high-dimensional MRF models. However, when dimensionality is high, meaning $p \gg \mathcal{{l}_{D( T )}}$, the threshold will fail because all the probabilities $\mathbb{P}\{ {{{D}_v} = d\mid v\,\,{\mathrm{is\ a\ weak\ variable}}} \}$ will approach 0. Later, we will demonstrate that the threshold fails in high-dimensional noise settings with multivariate outcomes. In cases where the mean threshold approach fails, a second strategy, known as variable hunting [19], involves iteratively selecting random subsets of variables, fitting the forest, and combining minimal depth with joint VIMP to prioritize the strongest variables. While effective when the number of features *p* greatly exceeds the number of samples (i.e., $p \gg n$), this method becomes computationally inefficient and can struggle in high-dimensional noise settings with multivariate outcomes. To overcome these limitations, we propose a variation of the minimal depth approach, which we outline in the next section. This variation allows for the selection of strong variables in both the response and predictor spaces within the MRF model.

#### Distribution of inverse minimal depth

To incorporate minimal depth into our variable selection method, we introduce a new statistic called inverse minimal depth (IMD), defined as:


(6) \begin{eqnarray*}
{{{\mathrm{M}}}_{\mathrm{v}}} = \left\{ {\begin{array}{@{}*{2}{c}@{}} {\frac{1}{{{{{\mathrm{D}}}_{\mathrm{v}}} + 1}}}&{{\mathrm{\ if\ v}}\in \mathcal{F}}\\ 0&{{\mathrm{\ if\ v}} \notin \mathcal{F}} \end{array}} \right.
\end{eqnarray*}

where $\mathcal{F}$ is the set of variables selected in the tree. For example, as shown Fig. 1, ${{{\mathrm{X}}}_{20}}$ has a minimal depth of ${{{\mathrm{D}}}_{{{{\mathrm{X}}}_{20}}}} = 0$, and its IMD is ${{M}_{{{X}_{20}}}} = 1$. Similarly, ${{{\mathrm{X}}}_6}$ has a minimal depth of ${{{\mathrm{D}}}_{{{{\mathrm{X}}}_6}}} = 1$, and its IMD is ${{M}_{{{X}_6}}} = 1/2$. In this setup, larger IMD values correspond to stronger variables, making it easier to identify them. Additionally, we apply a penalization technique that assigns an IMD of 0 to variables that are not selected in the tree. When ${\mathrm{v}}\in \mathcal{F}$, the distribution of ${\mathrm{D}}_{\mathrm{v}}$ can be directly transformed from the distribution of ${{{\mathrm{D}}}_{\mathrm{v}}}$ as follows:


(7) \begin{eqnarray*}
\mathbb{P}\left\{ {{{M}_v} = {\mathrm{m}}} \right\} = \left[ {\mathop \prod \limits_{{\mathrm{j}} = 0}^{1/{\mathrm{M}} - 1} {{{\left( {1 - {{{\mathrm{\pi }}}_{{\mathrm{v}},{\mathrm{j}}}}{{{\mathrm{\theta }}}_{{\mathrm{v}},{\mathrm{j}}}}} \right)}}^{\mathcal{l}_{\mathrm{j}}^{\mathrm{*}}}}} \right]\left[ {1 - {{{\left( {1 - {{{\mathrm{\pi }}}_{{\mathrm{v}},{\mathrm{d}}}}{{{\mathrm{\theta }}}_{{\mathrm{v}},{\mathrm{d}}}}} \right)}}^{\mathcal{l}_{{{{\mathrm{d}}}^{\mathrm{I}}}}^{\mathrm{*}}}}} \right], 0 < {\mathrm{m}} \le 1 \\
\end{eqnarray*}

Note that $\mathcal{l}_m^{\mathrm{*}}$ is the same value as in the distribution of minimal depth. With IMD, values are confined between 0 and 1. Variables not selected (${\mathrm{v}} \notin \mathcal{F}$) have ${{M}_v} = 0$, while stronger variables exhibit higher IMD values. The overall IMD for a variable ${\mathrm{v}}$ across the forest is calculated as the average IMD across all trees:


(8) \begin{eqnarray*}
{{M}_v} = \frac{{\mathop \sum \nolimits_{{\mathrm{b}}\in {\mathrm{B}}} M_v^{\left( b \right)}}}{{\mathrm{B}}}
\end{eqnarray*}

To evaluate the importance of variables in the response space, we assigned the IMD to the variables selected as the MSRV in each node. This is intuitive because the variables with lower IMD in ${{\bf X}}$ are more likely to be noise variables. As a result, when a decision tree node splits using these noise variables, it is less likely to select stronger, more influential variables from the response set ${{\bf Y}}$ as MSRV. This pattern reflects the structure of decision trees, where stronger variables, having greater predictive power, tend to appear earlier and higher in the tree. To further investigate the relationship between IMD and key MRF parameters—such as the number of trees, tree depth, and the proportion of response variables in each split—we conducted extensive simulations, detailed in Supplementary Note 3. Additionally, to compare the performance of the original MD and the enhanced IMD metric, we performed simulations outlined in Supplementary Note 4.

### Variable selection in high-dimensional 2-omics data

As previously noted, one method for variable selection based on minimal depth involves using a predefined threshold derived from the distribution of weak variables’ minimal depth. However, when $p \gg \mathcal{{l}_{D( T )}}$, all the probabilities in (5) approach zero [18]. Applying the same thresholding method to IMD yields similar challenges in high-dimensional datasets, where thresholding values also tend toward zero. To address this, we propose 2 additional methods for detecting strong variables that are not based on weak variable distributions. First, we introduce these approaches for 2-omics data in a multivariate random forest. Then, we extend the selection framework to multiomics variable selection.

#### Variable filtering

As the IMD of noise variables is close to or hovers over 0, we can select the threshold by multiplying a parameter ${\mathrm{\tau }}$ to the standard deviation of IMD: ${\mathrm{\tau }} \cdot {{{\mathrm{\sigma }}}_{\mathrm{m}}}$. The total number of variables selected can be controlled by varying the ${\mathrm{\tau }}.$ To determine the optimal value of ${\mathrm{\tau }}$, we use the mean out-of-bag (OOB) errors of both response and predictor variables for tuning. The mean OOB error is averaged across all the OOB errors in ${{\bf X}}$ and ${{\bf Y}}$. In the MRF setting, for each ${{{\mathrm{j}}}_{{\mathrm{th}}}}$ response in ${{{{\bf Y}}}_{{\mathrm{n}} \times {\mathrm{q}}}}$, the loss function becomes ${\mathrm{l}}( {{\mathrm{\hat{f}}}} ) = {{( {{{\bf Y}} - {\mathrm{\hat{f}}}( {{\bf X}} )} )}^2}$. Therefore, in the estimation of prediction error for response ${{{\mathrm{Y}}}_{\mathrm{j}}}$, the OOB sample can be defined as:


(9) \begin{eqnarray*}
{{l}_j}\left( {\hat{f},{\mathfrak{L}}_{\mathfrak{n}}^{OOB},X} \right) = \frac{1}{{\left| {{\mathfrak{L}}_{\mathfrak{n}}^{OOB}} \right|}}\mathop \sum \limits_{i:\left( {{{X}_i},{{Y}_{ij}}} \right) \in {\mathcal{L}}_n^{OOB}} {{\left( {{{Y}_{ij}} - \hat{f}\left( {{{X}_i} \cdot } \right)} \right)}^{2}}
\end{eqnarray*}

Similarly, the OOB errors of predictors ${{\bf X}}$ can be derived from the forest weights statistics. The OOB prediction of ${{\bf X}}$ can be formulated as follows:


(10) \begin{eqnarray*}
\widehat {f_{{\mathrm{OOB}}}^X} = \mathop \sum \limits_{t = 1}^n \frac{{\mathop \sum \nolimits_{b = 1}^{B - {{B}_i}} {{1}_{\left\{ {{{n}_{b,i}} = 0} \right\}}}{{1}_{\left\{ {{{{{\bf X}}}_{{\bf i}}}\in {{R}_j}} \right\}}}{{x}_t}}}{{\left( {B - {{B}_i}} \right)\mathop \sum \nolimits_{k = 1}^n {{1}_{\left\{ {{{n}_{b,k}} = 0} \right\}}}{{1}_{\left\{ {{{{{\bf X}}}_{{\bf k}}}\in {{R}_j}} \right\}}}}}
\end{eqnarray*}

(11) \begin{eqnarray*}
{{l}_j}\left( {\widehat {{{f}^{\boldsymbol{X}}}},{\mathfrak{L}}_{\mathfrak{n}}^{OOB},{\boldsymbol{X}}} \right) = \frac{1}{{\left| {{\mathfrak{L}}_{\mathfrak{n}}^{OOB}} \right|}}\mathop \sum \limits_{i:\left( {{{{\boldsymbol{X}}}_{\boldsymbol{i}}},{{X}_{ij}}} \right) \in {\mathcal{L}}_n^{OOB}} {{\left( {{{X}_{ij}} - \widehat {{{f}^X}}\left( {{{X}_i} \cdot } \right)} \right)}^{2}}
\end{eqnarray*}

With a step size of 0.1, we computed the mean OOB error using the variables above $\tau \cdot {{\sigma }_{{{d}^{{\bf I}}}}}$. To stabilize the OOB error, we repeated the model fittings *k* times and averaged the results to select the optimal $\tau $ based on a tolerable error deviation.

#### Detecting signals with mixture model

Given the distribution of differences in IMD between strong variables and noise variables, we can identify strong cross-correlated variables by fitting a 2-component mixture model to the forest IMD. We describe the univariate Gaussian mixture model as follows:


(12) \begin{eqnarray*}
{\mathrm{f}}\left( {{\mathrm{x}};{\mathrm{\Theta }}} \right) = {\mathrm{p}}\phi \left( {{\mathrm{x}};{{{\mathrm{\mu }}}_1},{\mathrm{\sigma }}_1^2} \right) + \left( {1 - {\mathrm{p}}} \right)\phi \left( {{\mathrm{x}};{{{\mathrm{\mu }}}_2},{\mathrm{\sigma }}_2^2} \right),
\end{eqnarray*}

where $\phi ( \cdot )$ is the normal distribution. As the forest IMD ranges from 0 to 1, we consider modeling the IMD using truncated distribution. A previous study used the truncated normal mixture model to model the intraclass correlation coefficient of DNA methylation probes. The distribution of the truncated normal mixture model is as follows:


(13) \begin{eqnarray*}
{\mathrm{f}}\left( {{\mathrm{x}};{\mathrm{\Theta }}} \right) = {\mathrm{p}}\frac{{\phi \left( {{\mathrm{x}};{{{\mathrm{\mu }}}_1},{\mathrm{\sigma }}_1^2} \right)}}{{1 - {\mathrm{\Phi }}\left( {0;{{{\mathrm{\mu }}}_1},{\mathrm{\sigma }}_1^2} \right)}} + \left( {1 - {\mathrm{p}}} \right)\phi \left( {{\mathrm{x}};{{{\mathrm{\mu }}}_2},{\mathrm{\sigma }}_2^2} \right),
\end{eqnarray*}

where $p\in [ {0,1} ]$ is the proportion of the first component, and the intraclass correlation is bounded by $( {0,1} )$. Here, we assume that the noise variables have relatively low forest IMD and more likely lie in the first component modeled by the normal or truncated normal distribution. To estimate the parameter of the mixture model, we used the expectation–maximization (EM) algorithm for the model fitting [20, 21]. To accommodate the variables with forest IMD = 0, we used the modified log-likelihood function proposed in:


(14) \begin{eqnarray*}
{\mathrm{log}}L\left( {\Theta ;x} \right) = {{n}_0}{\mathrm{log}}\left( {{{p}_0}} \right) + \mathop \sum \limits_{x\in {{d}^{{\bf I}}}\neq 0} {\mathrm{log}}\left( {{{p}_1}{{f}_1}\left( x \right)} \right)\\
+ \mathop \sum \limits_{x\in {{d}^{{\bf I}}}\neq 0} {\mathrm{log}}\left( {{{p}_2}{{f}_2}\left( x \right)} \right)
\end{eqnarray*}

where ${{p}_0}$ is the proportion of forest IMD = 0, ${{p}_1} = p( {1 - {{p}_0}} )$, and $p2 = ( {1 - p} )( {1 - {{p}_0}} )$. In the forest IMD, we separated the forest IMD = 0 and modeled the forest IMD $> 0$ using (12) or (13). Supplementary Fig. S1a shows the density of modeling the forest IMD using Gaussian mixture and truncated normal mixture models. The data are simulated by the latent model with the settings of $p = q = 500$, $n = 200$, and the first 30 variables of each dataset are cross-correlated with each other. Here, we can see that the forest IMD has a skewed distribution. The Gaussian mixture model shows a better fit of the forest IMD. For each component, the posterior probabilities can be calculated as:


(15) \begin{eqnarray*}
P{{r}_i}\left( x \right) = \frac{{{{p}_i}{{f}_i}\left( x \right)}}{{\sum{{p}_j}{{f}_j}\left( x \right)}},\quad j\in 0,1,2
\end{eqnarray*}

We selected variables with $P{{r}_1}( x ) < pr$ as the important variables, where $pr$ is a predefined value (i.e., $pr = 0.05$).

#### IMD transformation

In the previous section, we discovered that the mean IMD of strong variables is close to the mean IMD of noise variables in high-dimensional settings. However, the forest IMD of strong or cross-correlated variables may be low and mingled with noise variables. This can be challenging for the mixture model to capture due to the sparsity of strong variables. To address this issue, we propose a third method that explores the distribution of IMD for both noise and strong variables. Using the latent model, we simulated 2 datasets with the same settings as in the previous section. From Supplementary Fig. S1b (top panel), it is clear that the forest IMD of noise variables skews heavily toward the lower end of the IMD scale, clustering near zero. In contrast, the cross-correlated variables exhibit a broader distribution starting from zero, although this is less apparent due to their sparsity. This makes distinguishing between noise and strong variables using only a threshold on the original forest IMD difficult. Let $\mu $ (displayed as a black dashed line), ${{\mu }_{\textit{Noise}}}$ (displayed as a red dashed line), and ${{\mu }_{\textit{Strong}}}$ (displayed as a blue dashed line) denote the mean forest IMD of all variables, noise variables, and cross-correlated variables, respectively. It is clear to see that ${{\mu }_{\textit{Noise}}} \le \mu \le {{\mu }_{\textit{Strong}}}$ and that ${{\mu }_{\textit{Noise}}}$ tends toward $\mu $.

Based on these findings, we standardize the forest IMD of variable *v* using the mean $\mu $ and the standard error of IMD of *v*. The standardization is represented by:


(16) \begin{eqnarray*}
{{t}_{{{M}_v}}} = \frac{{{{M}_v} - \mu }}{{SE\left( {{{M}_v}} \right)}}
\end{eqnarray*}

We denote ${{{\mathrm{t}}}_{{{M}_v}}}$ as the *t*-score IMD of variable ${\mathrm{v}}$. This transformation yields a symmetric distribution (Supplementary Fig. S1b, bottom panel). The *t*-score IMD effectively differentiates between noise and cross-correlated variables. Noise variables cluster below the mean (${\mathrm{\mu }}$), while cross-correlated variables significantly diverge from ${\mathrm{\mu }}$. Using the lower tail of the *t*-distribution at the 0.05 level (${{{\mathrm{t}}}_{0.05,{\mathrm{df}} = {\mathrm{ntree}} - 1}}$, denoted by the navy line), we identified the cross-correlated variables that have ${{{\mathrm{t}}}_{{{M}_v}}} > {{{\mathrm{t}}}_{0.05,{\mathrm{df}} = {\mathrm{ntree}} - 1}}$. This specific threshold represents a point below which only an expected 5${\mathrm{\% }}$ of the IMD values for cross-correlated variables fall, thus indicating a higher level of importance.

### Multiomics framework

We now extend the variable selection framework to multiomics data. While we have introduced the variable selection method in 2-omics data, it is essential to recognize that the choice of which dataset to assign as responses or predictors can influence the results, particularly in complex datasets. Additionally, the strength of connections between datasets plays a critical role in multiomics variable selection, as some omics layers may share minimal information, which can introduce bias into the selection process. To address this, we introduce an algorithm designed to efficiently identify optimal connections among multiomics datasets. If a predefined connection structure is available, such as prior biological knowledge or experimentally verified links, we use these connections directly. Otherwise, we apply the method described below to infer data-driven connections.

To improve computational efficiency and address the high-dimensionality challenge in multiomics data analysis, we applied principal component analysis (PCA) to each dataset. PCA reduces the dimensionality of the datasets by selecting components that explain a predefined level of cumulative variance. This ensures that we retain the most relevant information while minimizing computational complexity. For each reduced dataset, we conducted MRF modeling, matching each dataset as a response to all others as predictors. We evaluated these models by calculating the mean OOB error, which was used to rank the models. The direction with the lowest OOB error was selected as the optimal connection between the datasets. This process enhances efficiency by avoiding exhaustive pairwise modeling and retains the most informative variables for further analysis.

For multiomics datasets, let $\mathcal{X} = \{ {{{{{\bf X}}}^{( 1 )}},{{{{\bf X}}}^{( 2 )}},\cdots,{{{{\bf X}}}^{( K )}}} \}$ denote a multiomics dataset with *K* omics data, where ${{{{\bf X}}}^{( k )}} = [ {X_1^{( k )},\cdots,X_{{{p}_k}}^{( k )}} ]\in {{\mathbb{R}}^{N \times {{p}_k}}}$ denotes the ${{k}_{{\mathrm{th}}}}$ omics data with *N* data samples and ${{p}_k}$ features. Let $\mathcal{M}$ be the the model collection that contains all optimal connected MRF models, and ${{{{\bf m}}}_{{{X}^{( i )}} \leftarrow {{X}^{( j )}}}}$ is the model in $\mathcal{M}$ with the direction of ${{X}^{( i )}}$ as responses and ${{X}^{( j )}}$ as predictors. Algorithm 2 in Supplementary Note 5 summarizes the framework of multiomics variable selection under the variable filtering and mixture model methods. First, we compute the mean IMD for each dataset across model set $\mathcal{M}$. Instead of individual IMD, we select the important variables based on the mean IMD. For multiomics variable selection under the IMD transformation, we choose the variables that the majority of the model selects (see Algorithm 3 in Supplementary Note 6).

### Simulation study

We designed a comprehensive simulation study to evaluate the performance of the proposed IMD-based methods under various conditions. Two different models were used: a **latent model** and a **nonlinear regression model**. For each model, we generated synthetic multiomics datasets to assess the impact of dimensionality, noise, and varying proportions of cross-correlated variables on model performance.

To evaluate the performance of our proposed methods in variable selection, we conducted a comprehensive simulation study. We generated synthetic multiomics datasets under various conditions to assess the impact of noise, high dimensionality, and different proportions of cross-correlated variables on the accuracy of our model. The simulation was designed to mimic realistic data integration challenges, where datasets contain a mixture of relevant and irrelevant variables. We compared our IMD-based methods with existing techniques such as SPLS, penalized matrix decomposition CCA (PMDCCA), and sparse regularized generalized CCA (SGCCA), assessing their ability to select important variables across different scenarios.

To benchmark our IMD‐based selection against nonlinear ensemble learners, we also included random forest (RF) permutation importance measurement, GBMs, and XGBoost. Because these algorithms lack native support for multivariate or unsupervised multiomics variable selection, we adapted them by fitting a separate univariate model for each response, computing per-feature importance scores, and then averaging those scores across responses to produce a single global ranking. We applied this procedure to 3 simulation frameworks: (i) a latent-factor model, (ii) a nonlinear regression model, and (iii) an interaction model in which the outcome depends on pairwise predictor interactions, each time generating paired 2-omics data where one layer contained only true signal variables (as the outcome) and the other served as predictors. In every scenario, we compared the rankings produced by MRF-IMD to those from 3 integration methods (SPLS, PMDCCA, SGCCA) and the 3 ensemble learners, evaluating each method’s ability to elevate known signal features via area under the precision-recall curve (PR-AUC), true-positive rate in the top k highest-ranked features, and ranking stability across replicates.

#### Latent model

For the simulation of the linear models, we use the following model:


\begin{eqnarray*}
{{{{\bf X}}}^{\left( {\mathrm{m}} \right)}} = {{{\mathrm{g}}}_{\mathrm{m}}}\left( {\mathrm{u}} \right){\mathrm{w}}_{\mathrm{m}}^ \top + {{\epsilon }_{\mathrm{m}}},
\end{eqnarray*}

where ${{\epsilon }_{{\mathrm{mi}}}} \sim {{\mathbb{N}}_{{{{\mathrm{p}}}_{\mathrm{m}}}}}( {0,{{\bf \Sigma }}} )$ for ${\mathrm{i}} = 1,\cdots,{{{\mathrm{p}}}_{\mathrm{m}}}$, and ${\mathrm{u}}$ is generated from normal distribution ${\mathrm{u}} \sim {\mathrm{N}}( {0,{{{\mathrm{\sigma }}}^2}} )$ using mean $0$ and ${\mathrm{\sigma }} = 2$. For the kernel functions, we set ${{{\mathrm{g}}}_1}( {\mathrm{\mu }} ) = {{{\mathrm{\mu }}}^2}$, ${{{\mathrm{g}}}_2}( {\mathrm{\mu }} ) = {\mathrm{exp}}( {\mathrm{\mu }} )$, and ${{{\mathrm{g}}}_3}( {\mathrm{\mu }} ) = {\mathrm{\mu }}$ to transform the latent variable ${\mathrm{\mu }}$. The weights variable ${{{\mathrm{w}}}_{\mathrm{m}}}$ were first generated by variables ${\mathrm{w}}_{\mathrm{m}}^0$ from uniform distribution ${\mathrm{w}}_{\mathrm{m}}^0 \sim {\mathrm{U}}( { - 1,1} )$. Then, the variables were normalized in the following way to ensure that the sum of squares of ${{{\mathrm{w}}}_{\mathrm{m}}}$ is equal to 1:


\begin{eqnarray*}
\begin{array}{*{20}{r}} {{{{\mathrm{w}}}_{{\mathrm{mj}}}} = \frac{{{\mathrm{w}}_{{\mathrm{mj}}}^0}}{{\sqrt {\mathop \sum \nolimits_{{\mathrm{i}} = 1}^{{{{\mathrm{p}}}_{\mathrm{m}}}} {{{\left( {{\mathrm{w}}_{{\mathrm{mi}}}^0} \right)}}^2}} }}} \end{array}
\end{eqnarray*}

For this model, we generated 2-dataset and 3-dataset settings. For each dataset, only the first ${\mathrm{p}}_{\mathrm{m}}^{\mathrm{c}}$ of weights ${{{\mathrm{w}}}_{\mathrm{m}}}$ are nonzero and selected as the features to identify. Scenarios were generated based on the following parameters: ${\mathrm{n}},{{{\mathrm{p}}}_{\mathrm{m}}},{\mathrm{p}}_{\mathrm{m}}^{\mathrm{c}}$, where ${\mathrm{n}}$ represents the sample size of all datasets; ${{{\mathrm{p}}}_{\mathrm{m}}}$ represents the feature numbers of ${{{{\bf X}}}^{( {\mathrm{m}} )}}$; and ${\mathrm{p}}_{\mathrm{m}}^{\mathrm{c}}$ is the number of features that cross-correlated w.r.t. ${{{{\bf X}}}^{( {\mathrm{m}} )}}$. Here we set the diagonal of the variance–covariance matrix ${{\bf \Sigma }}$ to ${{0.3}^2}$, and all variables in the ${{{{\bf X}}}^{( {\mathrm{m}} )}}$ were rescaled to have a mean of 0 and a standard deviation of 1. To generate data, we used the scenarios described in Table 1A for the 2- and 3-data settings. For the ranking based comparison, we focused exclusively on the 2-omics settings. In these settings, one omics layer (the “outcome” block) contained only the ${\mathrm{p}}_{\mathrm{m}}^{\mathrm{c}}$ true predictors and the other layer served as its predictors. We then evaluated the ability of each model to recover the true features into the top ${\mathrm{p}}_{\mathrm{m}}^{\mathrm{c}}$ of its ranked list.

**Table 1:** Simulation model scenarios for integration variable selection comparison

(A) Latent model			
Scenario	S1	S2	S3
**Sample size**	100	200	200
**Dimension**	200	500	1,000
**True model size**			
2-omics	20	30	50
3-omics			
(B) Nonlinear regression model			
**Scenario**	**S1**	**S2**	**S3**
**Sample size**	100	200	200
**Dimension**	200	500	1,000
**True model size**			
Setting 1	20/5		
Setting 2	40/10		

#### Nonlinear regression model

This simulation model was inspired by the simulation model in Degenhardt et al. [22]. Let ${\mathrm{U}}$ be the basis variables that are generated from a distribution. For this model, we will add 3 additional parameters, ${\mathrm{g}}$, ${\mathrm{p}}_{\mathrm{d}}^1$, and ${\mathrm{p}}_{\mathrm{d}}^2$, where ${\mathrm{g}}$ represents the group size of each correlated group in ${{\bf X}}$, and ${\mathrm{p}}_{\mathrm{d}}^1$, ${\mathrm{p}}_{\mathrm{d}}^2$ represent the number of variables that are not cross-correlated in ${{\bf X}}$ and ${{\bf Y}}$, respectively. This time, we use ${{{\mathrm{p}}}_{\mathrm{l}}}$ to represent the total number of basis variables that formed ${{\bf Y}}$. To generate the correlation between ${{\bf X}}$, the simulation is according to:


\begin{eqnarray*}
{\mathrm{X}}_{\mathrm{i}}^{\left( {\mathrm{j}} \right)} = \left\{ {\begin{array}{@{}*{1}{c}@{}} {{{{\mathrm{U}}}_{\mathrm{i}}} + \left( {0.01 + \frac{{0.5\left( {{\mathrm{j}} - 1} \right)}}{{{\mathrm{g}} - 1}}} \right) \cdot \epsilon ,\ for\ g > 1}\\ {{{{\mathrm{X}}}_{\mathrm{i}}} = {{{\mathrm{U}}}_{\mathrm{i}}} + \epsilon ,\ for\ g = 1} \end{array}} \right.
\end{eqnarray*}

for ${\mathrm{j}} = 1,\cdots,{\mathrm{g}}$ and ${\mathrm{i}} = 1,\cdots,{\mathrm{\ }}{{{\mathrm{p}}}_{\mathrm{l}}}$, where ${{{\mathrm{X}}}^{( {\mathrm{j}} )}}$ denotes the ${{{\mathrm{j}}}_{{\mathrm{th}}}}$ variable in group ${\mathrm{i}}$. Note that when ${\mathrm{g}} = 1$, there is no correlation between ${{{\mathrm{X}}}_{\mathrm{i}}}$. When ${\mathrm{g}} > 1$, there will be ${\mathrm{g}}$ correlated variables in variable group ${\mathrm{i}}$, the increase in ${\mathrm{j}}$ will decrease the correlation between basis variables ${\mathrm{U}}_{\mathrm{i}}^{( {\mathrm{j}} )}$ and ${{{\mathrm{X}}}_{\mathrm{i}}}$. Also notice that the total number of features that cross-correlated variables in ${\mathrm{p}}_1^{\mathrm{c}}$ is equal to ${\mathrm{g}} \cdot {{{\mathrm{p}}}_{\mathrm{l}}}$.

To generate ${{\bf Y}}$, we use the following kernel function:


\begin{eqnarray*}
{{{\mathrm{Y}}}_{\mathrm{k}}} &=& {{{\mathrm{f}}}_{\mathrm{k}}}\left( {\mathrm{u}} \right) = 0.25{\mathrm{exp}}\left( {4{{{\mathrm{u}}}_{3 \cdot {\mathrm{k}} - 2}}} \right) + \frac{4}{{1 + {\mathrm{exp}}\left( { - 20\left( {{{{\mathrm{u}}}_{3 \cdot {\mathrm{k}} - 1}} - 0.5} \right)} \right)}},\\
&&{\mathrm{k}} = 1,\cdots,{\mathrm{p}}_2^{\mathrm{c}}
\end{eqnarray*}

where each cross-correlated ${{{\mathrm{Y}}}_{\mathrm{k}}}$ is formed by 2 basis variables for a total of $2 \cdot {\mathrm{p}}_2^{\mathrm{c}}$ number of basis variables that formed all variables in ${\boldsymbol{Y}}$. Therefore, ${{{\mathrm{p}}}_{\mathrm{l}}} = 2 \cdot {\mathrm{p}}_2^{\mathrm{c}}$.

Finally, we will generate 2 sets of noise variables for ${{\bf X}}$ and ${{\bf Y}}$, respectively, using a multivariate Gaussian distribution with a mean of and an identity variance–covariance matrix. These 2 sets of variables are neither cross-correlated nor inner-correlated to each other. The number of independent sets for ${{\bf X}}$ is ${\mathrm{p}}_{\mathrm{d}}^1$, and the number of independent sets for ${{\bf Y}}$ is ${\mathrm{p}}_{\mathrm{d}}^2$. Note that all variables in the ${{\bf X}}$ and ${{\bf Y}}$ matrix are rescaled to have a mean of 0 and a standard deviation of 1 after being generated by the above models. We generated 2 settings of cross-correlated variables, and in each setting, we generated 3 different dimensional scenarios, as shown in Table 1B. For the ranking-based simulation, the true signal set comprises the first $p_1^c = g{{p}_l}$ features of ${{\bf X}}$. Each method produce a ranking over all ${{p}_1}$ candidate features, and we qualify the recovery of the $p_1^c$ true features.

#### Three-way nonlinear interaction

To further challenge the ability of each method to uncover higher-order effects for the ranking-based comparison, we simulated a purely nonlinear 3-way interaction. We generated $n = 200$ samples of $p = 300$ independent predictors ${{X}_i} \sim N( {0,1} )$, then selected $( {{{X}_1},{{X}_2},{{X}_3}} )$ as the shared interacting trio and added 2 “side” predictions per outcome $( {{{X}_4},{{X}_5}} )$ for ${{Y}_1}$ and $( {{{X}_6},{{X}_7}} )$ for ${{Y}_2}$. Specifically, the latent signals were

${{{\mathrm{\eta }}}_1} = ( {X_1^2 - 1} )( {{{X}_2} + 0.5} )( {{{X}_3} - 0.5} ) + {{X}_4} + {{X}_5},\quad {{{\mathrm{\eta }}}_2} = ( {X_1^2 - 0.5} )( {{{X}_2} + 0.5} )( {{{X}_3} - 0.5} ) + {{X}_6} - {{X}_7}.$ and set the corresponding response


\begin{eqnarray*}
{{Y}_1} = 1.5{{\eta }_1} + {{\epsilon }_1},\ {{Y}_2} = 2{{\eta }_2} + {{\epsilon }_2}\quad {{\epsilon }_{k\in 1,2}} \sim \mathcal{N}\left( {0,{{\sigma }^2}} \right),
\end{eqnarray*}

After centering and scaling all columns of *X* and *Y* to unit variance, we tasked each algorithm with ranking the full set of 300 predictors. We then compared nonlinear ensemble learners (random forest with permutation-importance, GBM, XGBoost) against multiomics integration methods (SPLS, PMDCCA, SGCCA) by measuring each method’s PR-AUC and true‐positive rate among the top $k{{p}_c}$ features per response.

All MRF‐IMD models were implemented via the *rfsrc* function in the randomForestSRC R package, using its out‐of‐the‐box settings (default mtry, nodesize, and splitting rules) across every simulation scenario. For the integration variable selection comparison, we compared our methods to SPLS (mixOmics R package), PMDCCA (PMA R package), and SGCCA (regularized generalized CCA [RGCCA] R package). For PMDCCA and SGCCA, we selected variables tuned by the build-in tuning functions of their packages. For SPLS, we directly entered the number of true variables to the function. For the ranking-based comparison, we added the ensemble methods, random forests (permutation‐importance), GBM, and XGBoost and recorded the importance scores generated by their respective R packages (randomForest, gbm, and xgboost). For the integrative methods (SPLS, PMDCCA, and SGCCA), we collapsed each into a single ranking by averaging the absolute loadings over the first 5 components. This approach ensured that every method returned a complete ranking over the same candidate set.

### Evaluation metrics

For each simulation scenario, we evaluated the performance of the variable selection methods using the following metrics: recall, precision, PR-AUC, and model size.

#### Recall

This metric measures the proportion of true important variables identified by the model. Let ${\mathrm{tp}}$ be the number of variables the model selected as important that are true important variables and ${\mathrm{fn}}$ be the number of variables that the model selected as important variables that are noise variables. The recall equals $\frac{{{\mathrm{tp}}}}{{{\mathrm{tp}} + {\mathrm{fn}}}}$. A higher recall indicates that more important variables were selected.

#### Precision

This metric measures the accuracy of the variable selection, focusing on the proportion of correctly identified important variables out of all selected variables. Let ${\mathrm{fp}}$ be the number of variables that the model selected as important that are true nonimportant variables and ${\mathrm{tp}}$ as described above. The precision equals $\frac{{{\mathrm{tp}}}}{{{\mathrm{tp}} + {\mathrm{fp}}}}$. Higher precision indicates that fewer noise variables were incorrectly selected.

#### PR-AUC

This is a common metric for imbalanced datasets where important variables (positive cases) are fewer compared to noise variables (negative cases). PR-AUC ranges from 0 to 1, with a value of 1 indicating a perfect classifier. We reported the average PR-AUC across all datasets.

#### Model size

This represents the number of variables selected by the model. We calculated the average model size for each simulation scenario and reported the standard deviation to capture the variability in model size across replicates. For stability, each scenario was simulated 50 times, and all evaluation metrics (except model size) were averaged across replicates. For model size, we reported the average and standard deviation to assess the variability in the number of selected variables.

### Real data preprocessing

#### TCGA data

To demonstrate the effectiveness of our proposed methods, we applied our methods to 3 TCGA datasets: BRCA [23], COAD [24], and pan-cancer [25]. For TCGA-BRCA and TCGA-COAD data, 3 types of omics data (i.e., mRNA expression data [Gene], miRNA expression data [miRNA], and DNA methylation data [Methyl]) were selected to perform the analysis. For TCGA–pan-cancer, we selected the ATAC sequencing (ATAC-seq) data and RNA sequencing (RNA-seq) datasets. All the RNA-seq data, which were log_2_-transformed transcripts per million (TPM) for each cancer type, were obtained from the R package *TCGAbiolinks*. Other datasets were downloaded through UCSC Xena [26]. For TCGA pan-cancer, we selected the ATAC sequencing data and RNA-seq datasets. Each of these data types provides unique, yet complementary, information for distinguishing between different types or states. Analyzing RNA-seq and ATAC-seq independently, however, can lead to inconsistent classifications. Furthermore, studying these 2 modalities in isolation may diminish the overall power of the analysis, as they both represent the same fundamental types or states. Only samples that existed across all data types were included in our study. We applied log_2_ transformation to mRNA and miRNA expression data. For DNA methylation, we initially filtered out probes not included in the Illumina Infinium HumanMethylation450k BeadChip to ensure a better interpretation of results. For miRNA, we removed all the variables containing missing values. To streamline our analysis, we limited the features of mRNA and DNA methylation data to the top 2,000 most variable expressions in both the BRCA and COAD datasets. For the pan-cancer dataset, we restricted ATAC-seq and RNA-seq to the top 50,000 and 5,000 most variable expressions, respectively. Table 2 summarizes the datasets used in the analysis.

**Table 2:** Summary of datasets

Dataset	Number of arrays	Number of arrays for training	Number of samples	Number of arrays selected by
				MRF-IMD
TCGA-	*mRNA, DNAm, miRNA*			
BRCA	20,530, 485,577, 2,238	2,000, 2,000, 228	674	102, 141, 22
COAD	20,530, 485,577, 2,113	2,000, 2,000, 252	257	139, 107, 18
TCGA-	*ATAC-seq, RNA-seq*			
Pan-cancer	562,709, 59,390	50,000, 5,000	383	186, 300
ADNI	*Gene expression, DNAm*			
	49,395, 734,743	2,000, 2,000	Total: 468CN: 198, MCI: 288	161, 54

#### ADNI data

We analyzed data from the ADNI, including DNA methylation profiles, for 538 cognitively normal (CN) participants and patients with mild cognitive impairment (MCI), measured on Illumina HumanMethylation EPIC v1 arrays preprocessed by Zhang et al. [27], and matched gene expression data from Affymetrix Human Genome U219 microarrays. In this study, we mainly focus on dementia onset. Given the heterogeneity and complex progression of Alzheimer’s disease (AD), we first selected the top 2,000 CpG methylation sites most significantly associated with AD dementia onset, using *P* value–based screening in the Framingham Heart Study (FHS) dataset as described by Zhang et al. [27]. For gene expression, we removed probes lacking gene symbols, removed lowly expressed probes with gene expression below the 10th percentile in over 80% of samples, collapsed remaining probes by gene via median values, and finally selected the 2,000 most variable genes. Dementia conversion was defined as the conversion from CN to MCI or dementia and from MCI to dementia. After intersecting dementia phenotype, DNA methylation, and gene expression data, we obtained 468 common samples for our integrative analyses. DNA methylation, gene expression data, and the dementia status of the subjects were obtained from the ADNI study website (adni.loni.usc.edu). Table 2 summarizes the datasets used in this analysis.

## Results

### Summary of the MRF-IMD framework

Our integrative multiomics pipeline targets datasets in which multiple omics layers share the same samples. We aim to identify shared biomarkers, that is, variables in one omics block whose variation is strongly associated with features in another block. To do this, we first establish a connection structure between layers: if prior biological knowledge or experimentally verified links exist, we adopt these directly; otherwise, we infer an optimal connection based on cross-block correlation. Given this pairing, we fit an MRF model for each connected pair, treating one omics layer as the multivariate response and the other as predictors. From each tree in the forest, we compute the minimal depth of every predictor (the depth at which it first splits), convert these to IMD scores, and average across all trees to yield a global importance ranking. Finally, we apply 1 of 3 IMD-based selection strategies, filter (user-defined threshold), mixture (balanced sensitivity-specificity), or transformation (distribution-normalized ranking), to extract a compact set of cross-omics features. Downstream, these selected biomarkers are evaluated via functional enrichment, patient stratification, and clustering analyses. The entire workflow is illustrated in Fig. 1.

### Evaluation of simulated data

#### Integrative variable selection benchmark

**Figure 2:**
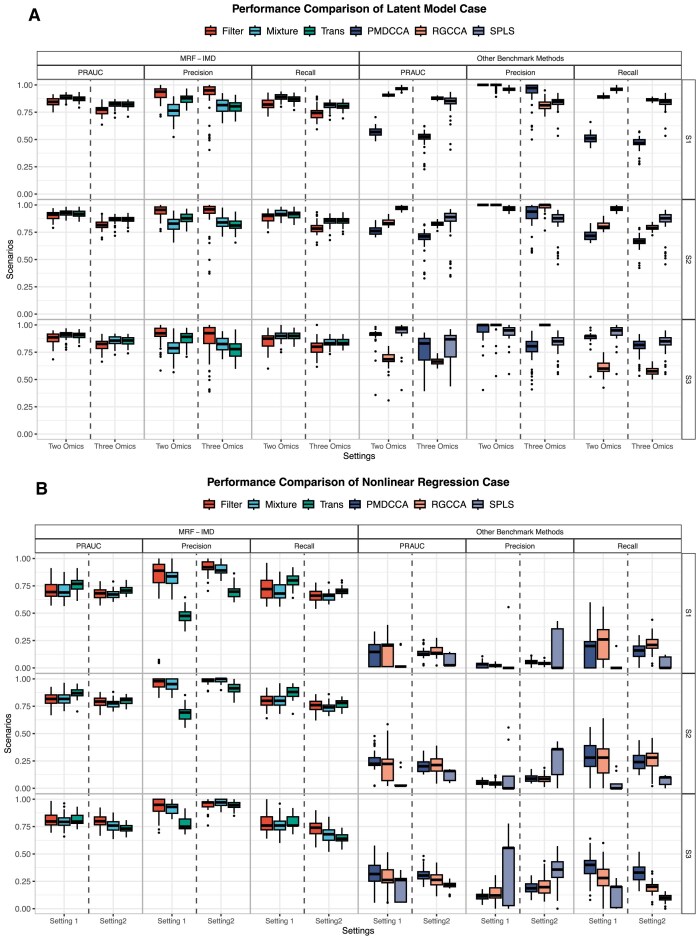
Simulation results. Boxplots of simulation results. Performance measures are PR-AUC, precision, and recall. In most scenarios, the MRF-IMD methods showed competitive selection outcomes in both (A) the latent model and (B) the nonlinear regression model.

The variable selection results are summarized in Fig. 2 and Table 3 (Supplementary Note 7). Under the latent‐factor model (Fig. 2A, Table 3A), all 3 MRF-IMD variants delivered competitive PR-AUC, precision and recall compared with SPLS, PMDCCA, and SGCCA, matching these methods in the very linear scenarios they were designed for. By contrast, in the nonlinear‐regression simulations (Fig. 2B, Table 3B), IMD-filter, IMD-mixture, and IMD-transformation significantly outperformed the CCA-based integrators, maintaining high PR-AUC and stable precision even as model complexity increased. Among the IMD methods, filter was most conservative, mixture struck the strongest sensitivity-specificity balance, and transformation excelled at highlighting signals when IMD weights were tightly clustered. Extended simulation results are provided in Supplementary Note 7.

**Table 3:** Simulation results. Performance measures are the mean of PR-AUC, precision, recall, and model size (standard deviations)

(A) Latent model										
Model	Scenario	Selection method	Setting							
			2-omics				3-omics			
			PR-AUC	Precision	Recall	Model size	PR-AUC	Precision	Recall	Model size
MRF-IMD	S1	Filter	0.90 (0.04)	0.87 (0.09)	0.88 (0.06)	41.36 (6.33)	0.82 (0.05)	0.86 (0.14)	0.79 (0.07)	57.78 (18.41)
		Mixture	0.90 (0.04)	0.86 (0.06)	0.88 (0.05)	41.08 (4.25)	0.85 (0.04)	0.87 (0.06)	0.82 (0.04)	57.16 (4.80)
		Trans	0.91 (0.03)	0.87 (0.06)	0.89 (0.04)	41.10 (3.48)	0.86 (0.04)	0.77 (0.08)	0.84 (0.04)	65.96 (7.28)
	S2	Filter	0.89 (0.05)	0.94 (0.05)	0.87 (0.06)	55.50 (5.65)	0.82 (0.04)	0.91 (0.12)	0.79 (0.05)	81.14 (23.95)
		Mixture	0.90 (0.04)	0.93 (0.04)	0.89 (0.04)	57.74 (3.44)	0.85 (0.03)	0.92 (0.04)	0.83 (0.04)	81.30 (4.77)
		Trans	0.92 (0.03)	0.88 (0.05)	0.91 (0.04)	62.54 (4.38)	0.87 (0.03)	0.82 (0.06)	0.85 (0.04)	94.44 (7.57)
	S3	Filter	0.83 (0.04)	0.94 (0.04)	0.81 (0.05)	87.14 (8.82)	0.77 (0.05)	0.91 (0.12)	0.74 (0.06)	126.28 (34.31)
		Mixture	0.86 (0.03)	0.90 (0.03)	0.84 (0.04)	94.32 (5.80)	0.81 (0.04)	0.92 (0.04)	0.79 (0.04)	128.62 (8.21)
		Trans	0.88 (0.03)	0.87 (0.04)	0.87 (0.03)	99.80 (6.60)	0.82 (0.03)	0.80 (0.06)	0.81 (0.04)	152.40 (13.99)
Other benchmarks	S1	PMDCCA	0.90 (0.09)	0.95 (0.10)	0.89 (0.06)	37.48 (3.22)	0.76 (0.15)	0.77 (0.13)	0.79 (0.10)	62.54 (5.51)
		RGCCA	0.69 (0.08)	0.98 (0.08)	0.62 (0.06)	25.40 (2.70)	0.66 (0.03)	1.00 (0.00)	0.57 (0.04)	34.24 (2.25)
		SPLS	0.94 (0.10)	0.93 (0.07)	0.93 (0.07)	40.00 (0.00)	0.79 (0.16)	0.82 (0.11)	0.82 (0.11)	60.00 (0.00)
	S2	PMDCCA	0.76 (0.04)	1.00 (0.00)	0.72 (0.04)	43.04 (2.64)	0.67 (0.12)	0.90 (0.12)	0.65 (0.08)	65.52 (4.44)
		RGCCA	0.84 (0.03)	1.00 (0.00)	0.81 (0.04)	48.46 (2.13)	0.82 (0.02)	0.98 (0.04)	0.79 (0.03)	72.68 (4.46)
		SPLS	0.97 (0.02)	0.97 (0.02)	0.97 (0.02)	60.00 (0.00)	0.82 (0.17)	0.83 (0.12)	0.83 (0.12)	90.00 (0.00)
	S3	PMDCCA	0.57 (0.04)	1.00 (0.00)	0.51 (0.05)	51.08 (4.51)	0.50 (0.09)	0.93 (0.12)	0.46 (0.06)	74.84 (5.24)
		RGCCA	0.91 (0.01)	0.99 (0.02)	0.89 (0.02)	90.36 (2.77)	0.88 (0.01)	0.82 (0.05)	0.87 (0.01)	159.36 (8.33)
		SPLS	0.97 (0.01)	0.96 (0.02)	0.96 (0.02)	100.00 (0.00)	0.83 (0.10)	0.83 (0.07)	0.83 (0.07)	150.00 (0.00)
(B) Nonlinear regression model										
Model	Scenario	Selection method	Setting							
			Setting 1				Setting 2			
			PR-AUC	Precision	Recall	Model size	PR-AUC	Precision	Recall	Model size
MRF-IMD	S1	Filter	0.71 (0.07)	0.77 (0.30)	0.73 (0.11)	73.06 (134.66)	0.68 (0.05)	0.92 (0.06)	0.66 (0.06)	36.02 (4.61)
		Mixture	0.70 (0.07)	0.82 (0.09)	0.70 (0.07)	21.54 (2.87)	0.67 (0.04)	0.90 (0.05)	0.65 (0.04)	36.20 (3.14)
		Trans	0.76 (0.06)	0.48 (0.06)	0.79 (0.06)	41.72 (3.99)	0.71 (0.04)	0.70 (0.06)	0.70 (0.04)	50.72 (3.73)
	S2	Filter	0.81 (0.07)	0.96 (0.05)	0.80 (0.07)	20.88 (2.47)	0.79 (0.05)	0.98 (0.02)	0.75 (0.06)	38.46 (3.35)
		Mixture	0.83 (0.06)	0.94 (0.05)	0.81 (0.06)	21.58 (2.12)	0.78 (0.04)	0.99 (0.02)	0.74 (0.05)	37.64 (2.43)
		Trans	0.87 (0.05)	0.68 (0.07)	0.87 (0.05)	32.00 (3.10)	0.80 (0.03)	0.91 (0.05)	0.77 (0.04)	42.48 (2.71)
	S3	Filter	0.81 (0.07)	0.92 (0.08)	0.78 (0.08)	21.34 (3.96)	0.80 (0.06)	0.95 (0.05)	0.73 (0.08)	38.56 (5.42)
		Mixture	0.80 (0.06)	0.92 (0.05)	0.77 (0.08)	20.86 (2.60)	0.76 (0.06)	0.96 (0.03)	0.68 (0.07)	35.30 (4.21)
		Trans	0.81 (0.05)	0.77 (0.06)	0.78 (0.06)	25.56 (2.06)	0.73 (0.04)	0.95 (0.04)	0.64 (0.05)	33.96 (2.69)
Other benchmarks	S1	PMDCCA	0.12 (0.11)	0.03 (0.03)	0.15 (0.15)	107.08 (45.68)	0.13 (0.06)	0.05 (0.03)	0.15 (0.07)	131.40 (34.01)
		RGCCA	0.15 (0.12)	0.02 (0.02)	0.23 (0.17)	226.58 (56.22)	0.15 (0.05)	0.04 (0.02)	0.22 (0.08)	260.08 (25.79)
		SPLS	0.04 (0.08)	0.09 (0.21)	0.03 (0.07)	9.00 (0.00)	0.06 (0.05)	0.13 (0.17)	0.04 (0.05)	14.00 (0.00)
	S2	PMDCCA	0.22 (0.12)	0.05 (0.03)	0.27 (0.16)	124.66 (33.19)	0.21 (0.06)	0.09 (0.03)	0.24 (0.08)	134.76 (11.47)
		RGCCA	0.22 (0.14)	0.04 (0.03)	0.26 (0.17)	137.80 (39.43)	0.22 (0.08)	0.09 (0.04)	0.26 (0.09)	149.80 (22.49)
		SPLS	0.07 (0.09)	0.14 (0.23)	0.05 (0.08)	9.00 (0.00)	0.13 (0.05)	0.27 (0.16)	0.08 (0.04)	14.00 (0.00)
	S3	PMDCCA	0.31 (0.12)	0.11 (0.03)	0.39 (0.12)	88.48 (12.20)	0.31 (0.06)	0.19 (0.05)	0.33 (0.08)	87.24 (8.20)
		RGCCA	0.27 (0.11)	0.14 (0.06)	0.28 (0.12)	48.86 (10.07)	0.26 (0.06)	0.21 (0.08)	0.20 (0.06)	49.28 (8.49)
		SPLS	0.20 (0.10)	0.39 (0.26)	0.14 (0.09)	9.00 (0.00)	0.21 (0.04)	0.32 (0.15)	0.09 (0.04)	14.00 (0.00)

#### Ranking-based comparison

In ranking‐based simulations across 3 models, latent, 3‐way interaction, and nonlinear regression models, our MRF-IMD methods consistently achieved competitive PR-AUC results at the top *k* predictors. The boxplot from Supplementary Fig. S2 shows that the IMD scores achieve the stable results across all scenarios. Extended simulation results are provided in Supplementary Note 7.

In summary, our simulation studies confirmed that the MRF-IMD framework can identify cross-correlated variables reliably under a wide array of conditions, outperforming several well-established methods, including integrative methods and nonlinear ensemble methods. These results suggest that by embracing nonlinear modeling, leveraging IMD-based variable selection, and providing flexible selection strategies, our framework is well suited for integrative multiomics analysis. This strong performance in controlled simulations sets the stage for more complex real-world applications and further supports the potential utility of MRF-IMD methods in guiding biomarker discovery and driving meaningful biological interpretations.

### Comprehensive analysis of individual cancer data: breast cancer and colorectal cancer

#### Data summary and model configurations

We applied our MRF-based framework to BRCA and COAD data from TCGA. For BRCA, we examined 2 directional models to reduce the model running: $gene \leftarrow \textit{methyl}$ and $mirna \leftarrow \textit{methyl}$. For COAD, we considered the $mirna \leftarrow \textit{methyl}$ and $mirna \leftarrow \textit{gene}$ configuration selected by the optimal connection. Key cohort and feature counts for each cancer and omics layer are summarized in Table 2.

#### Stability and sensitivity analysis

To assess the stability of the identified variables of our MRF-IMD method at different seed settings, we repeated running the MRF-IMD model 30× on different seeds and summarized (i) model size and (ii) the pairwise overlap coefficient (also known as the Szymkiewicz–Simpson coefficient intersection divided by the smaller set size) for each omics block. Across BRCA and COAD, **filter** yielded the most compact yet stable signatures, **mixture** provided a balanced middle ground, and **transformation** returned the broadest panels with still high reproducibility. These patterns were consistent across genes, CpGs, and miRNAs. Supplementary Fig. S3 summarizes the stability of gene, CpG, and miRNA selections across 30 MRF‐IMD runs for both TCGA‐BRCA (top panels) and TCGA‐COAD (bottom panels). In BRCA, the filter strategy consistently yields the most compact signatures (median $\approx \ $73 genes, 91 CpGs, 33 miRNAs) and attains high reproducibility (overlap coefficients $\approx \ $0.80 for genes, 0.90 for CpGs, 0.95 for miRNAs). The mixture approach selects intermediate‐sized sets (median $\approx $100 genes, 140 CpGs, 27 miRNAs) with slightly lower median overlaps (0.78/0.85/0.98), while transformation produces the largest signatures ($\approx \ $235 genes, 290 CpGs, 45 miRNAs) but still maintains strong stability (0.75/0.80/0.90). In COAD, all 3 methods expand their gene and CpG panels relative to BRCA, yet the same ranking of stability holds. Filter picks ~120 genes, 75 CpGs, and 43 miRNAs (overlaps $\approx \ $0.68/0.78/0.90); mixture yields a median selection of 145/125/15 features (overlaps $\approx \ $0.70/0.75/1.00); and transformation selects a median selection of 120/130/60 features with moderate reproducibility (0.62/0.65/0.85). These results demonstrate that, regardless of cohort or omics layer, IMD-transformation offers the best trade‐off between breadth and consistency, IMD-filter delivers the most parsimonious yet stable core signature, and IMD-mixture provides a middle ground in both model size and overlap.

#### Signature selection for comparative evaluation

For the rest of the analysis, we adopted the IMD-mixture variable selection strategy as it delivers a balanced signature size (neither too sparse nor overly broad) while maintaining high selection stability across seeds. For reproducible research, we selected the seed for the model fitting that was closest to the median across 30 seeds. To enable a fair head-to-head evaluation, we then configured SPLS, PMDCCA, and RGCCA to yield the similar total number of features. For SPLS (mixOmics), we specified the keepX vector in block.spls function to match the per‐component counts from IMD‐mixture. PMDCCA (PMA) was run with its default CCA.permute routine, which, if no penalty is supplied, automatically selects optimal penalty terms via permutation testing. For RGCCA (RGCCA), we manually set the shrinkage penalties to 0.1 to ensure the selection sparsity. All integrative models were run with 5 components for the downstream prognostic analyses, yielding comparable feature set sizes across methods.

#### Interpretation of selected biomarkers

**Figure 3:**
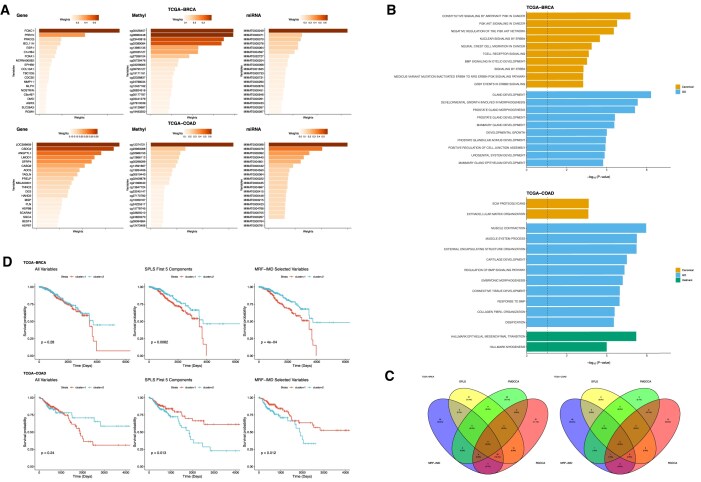
TCGA-BRCA and TCGA-COAD analysis results. (A) Top 20 variable weights chosen by 1 IMD-mixture model. (B) Functional enrichment analysis of the selected genes with 1 IMD-filter model performed using the clusterProfiler R package. Top 10 significant pathways of C2:CP, GO, and Hallmark (i.e., FDR < 0.05) were displayed for both TCGA-BRCA and TCGA-COAD. (C) Four-way Venn diagrams of the significant pathways selected by MRF-IMD, SPLS, PMDCCA, and RGCCA in BRCA and COAD. (D) Kaplan–Meier curves of the patient stratification results from all variables, SPLS 5 components, and the selected MRF-IMD-mixture methods.

Figure 3A (top panel) highlights the top 20 BRCA biomarkers by the IMD-mixture weight across genes (left), CpG probes (center), and miRNA (right). Prominent genes included *FOXC1, PRR15*, and *PRKCQ. FOXC1* drives epithelial–mesenchymal transition and correlates with poor survival in breast cancer, *PRR15* has recently been identified as a luminal‐subtype marker in hormone receptor–positive tumors [28], and *PRKCQ* suppresses ER${\mathrm{\alpha }}$ expression and is required for mammary tumorigenesis in triple-negative models [29]. Other high-ranking genes include *MMP11*, which is well documented in the literature as playing a pivotal role in breast cancer, showing high expression levels in early luminal subtypes [30, 31]. Additionally, *BCL11A* has also been shown to be related to triple-negative breast cancer [32]. Furthermore, *ESR1*, the gene that encodes the estrogen receptor (ER) along with pioneering transcription factor *FOXA1*, is well established factors in hormonally dependent breast cancer [33]. Among the DNA methylation features, probes such as cg03441279 in *BCL9* and cg12427162 in *SFT2D2* have been associated with breast cancer prognosis [31, 34]. On the miRNA side, MIMAT0003249 (hsa-miR-584-5p) and MIMAT0000064 (hsa-let-7c-5p) stood out, both having been previously implicated in breast cancer biology [35, 36].

For the TCGA-COAD dataset (Fig. 3A, bottom panel), 2 of the top genes selected by the model, *SFRP4* and *ANGPTL1*, are known to be highly expressed in colorectal cancer (CRC) and have been linked to poor clinical outcomes in patients with CRC [37–39]. Although fewer DNA methylation probes emerged prominently, cg12374721, which maps to the *PRAC2* locus, exceeded the 0.5 weight threshold. This site was previously flagged as a novel pan‐cancer methylation biomarker, showing consistent differential methylation in colon, rectal, and prostate tissues [40, 41]. For miRNAs, MIMAT0000098 (hsa-miR-100–5p) and MIMAT0000076 (hsa-miR-21–5p) are top ranked; miR-100 is significantly downregulated in colorectal tumors, and lower miR-100 expression correlates with advanced stage and poorer overall survival in patients with CRC [42]. Additionally, a study also shown that hsa-miR-21–5p is useful in the diagnosis of CRC [43].

#### Functional analysis and prognostic comparison

We then applied the functional enrichment analysis to the selected genes. The functional analysis was performed using the *clusterProfiler* R package focusing on Canonical (C2:CP), Gene Ontology (GO) (C5:BP), and Hallmark pathways from the Molecular Signatures Database [44]. Figure 3B displays the top 10 significant pathways (i.e., false discovery rate [FDR] < 0.05). In BRCA, the leading Canonical pathway hits are *Constitutive Signaling by Aberrant PI3K in Cancer, PI3K AKT Signaling in Cancer*, and multiple *ERBB4*-driven routes, reflecting the centrality of PI3K/AKT and ERBB networks in hormone receptor–positive breast tumors [45, 46]. In GO, *Gland Development, Mammary Gland Epithelium Development*, and *Positive Regulation of Cell–Cell Junction Assembly* are crucial in understanding tumorigenesis, particularly in cancers like breast cancer [47]. In COAD, enriched Canonical pathways are dominated by extracellular matrix (ECM) remodeling (*ECM Proteoglycans, Extracellular Matrix Organization*), while GO terms center on muscle and connective tissue processes (*Muscle Contraction, Cartilage Development, Collagen Fibril Organization*). Notably, the Hallmark sets *Epithelial–Mesenchymal Transition* and *Myogenesis* also score among the top hits, underlining the dual roles of stromal reprogramming and epithelial–mesenchymal transition (EMT) in colorectal cancer progression [48, 49]. All the significant pathways obtained from MRF-IMD–selected genes for BRCA and COAD are listed in Supplementary Table S1. In BRCA, MRF-IMD uniquely recovers PI3K/AKT- and ERBB-driven programs central to hormone receptor–positive disease, which linear integrators often miss at matched model sizes. In COAD, MRF-IMD enriches ECM remodeling and EMT pathways, aligning with stromal reprogramming in colorectal tumor progression.

Figure 3C shows the 4-way Venn diagrams of the significant pathways selected by MRF-IMD, SPLS, PMDCCA, and RGCCA in BRCA and COAD. In BRCA (left), MRF-IMD identified 55 unique pathways (25.3%), while SPLS, PMDCCA, and RGCCA only identified 27 (12.4%), 35 (16.1%), and 37 (17.1%) unique pathways, respectively. Only 37 pathways (17.1%) overlap between MRF‐IMD and SPLS, and 40 (20.3%) between MRF‐IMD and RGCCA; there are no shared pathways in PMDCCA, nor are there any common to all 4 methods. The core oncogenic pathways such as *Constitutive Signaling by Aberrant PI3K in Cancer* and *PI3K AKT Signaling in Cancer* are uniquely retrieved by MRF-IMD that the linear integrators might miss. In COAD, MRF-IMD again dominates with 48 unique enrichments (64.0%), whereas SPLS yields none, PMDCCA yields 2 (2.7%), and RGCCA yields 12 (16.0%). Only 4 pathways (5.3%) overlap between MRF‐IMD and RGCCA, and 2 (2.6%) between MRF‐IMD and PMDCCA; there are no shared pathways in SPLS or common to all 4 methods. Among its unique COAD hits, MRF-IMD highlights *Collagen Fibril Organization* and *Ossification*, underscoring its ability to capture tissue-specific remodeling programs that go beyond the canonical and component-based integrators.

An important goal in cancer multiomics studies is to identify biomarkers that not only reflect biological mechanisms but also correlate with clinical outcomes. To evaluate the prognostic value of the variables identified by our MRF-based methods, we applied the integrative nonnegative matrix factorization (IntNMF) method [50] to combine the selected variables from the 3 omics data types for both BRCA and COAD. This integration allowed us to cluster patients into 2 groups representing high- and low-risk survival profiles. Figure 3D shows the resulting Kaplan–Meier curves of the grouping results from clustering using all variables, top 5 SPLS components, and the variables selected by MRF-IMD-mixture methods. Table 4 reports the median log-rank *P* values across 30 seeds of MRF-IMD methods and log-rank *P* values of other benchmark methods. In BRCA, clustering on all variables yields no significant separation (*P* = 0.28), while SPLS and the CCA‐based methods achieve modest significance only when using all 5 components (SPLS: *P* = $8.2 \times {{10}^{ - 3}}$; PMDCCA: *P* = $1.4 \times {{10}^{ - 2}}$; RGCCA: *P* = $2.4 \times {{10}^{ - 2}}$) but fail when restricted to their selected features. In contrast, MRF-IMD’s filter and mixture strategies produce highly significant stratification (median *P* = $7.9 \times {{10}^{ - 4}}$ and $1.0 \times {{10}^{ - 3}}$ respectively), with the filter set delivering the strongest separation in the Kaplan–Meier curves (*P* = $4 \times {{10}^{ - 4}}$). In COAD, neither the full feature set (*P* = 0.24) nor SPLS-selected variables or RGCCA‐derived signatures yield significant stratification. SPLS with all 5 components and PMDCCA’s selected variables attain significance (*P* = $1.3 \times {{10}^{ - 2}}\ and\ 3.0 \times {{10}^{ - 2}}$), but once again, the MRF-IMD filter and mixture panels outperform, both achieving *P* = $1.2 - 1.4 \times {{10}^{ - 2}}$.

**Table 4:** TCGA-BRCA and COAD prognosis results

TCGA-BRCA			TCGA-COAD		
Methods		(Median) Log-rank *P* value	Methods		(Median) Log-rank *P* value
All variables		2.75E-01	All variables		2.43E-01
SPLS	All 5 components	8.21E-03	SPLS	All 5 components	1.31E-02
	Selected variables from first component	6.50E-02		Selected variables from first component	9.86E-01
PMDCCA	All 5 components	1.43E-02	PMDCCA	All 5 components	9.89E-01
	Selected variables from first component	6.15E-01		Selected variables from first component	2.99E-02
RGCCA	All 5 components	2.59E-02	RGCCA	All 5 components	8.73E-01
	Selected variables from first component	2.18E-02		Selected variables from first component	5.34E-02
MRF-IMD	Filter	7.94E-04	MRF-IMD	Filter	1.23E-02
	Mixture	1.02E-03		Mixture	1.38E-02
	Test	6.17E-02		Test	2.14E-02

Together, these results demonstrate that our MRF-IMD–derived biomarker sets consistently enable more robust risk stratification than existing integrative methods, confirming their potential clinical utility for patient stratification and prognostic modeling.

In summary, applying our MRF-based framework to BRCA and COAD datasets uncovered key genes, methylation probes, and miRNAs that are consistent with known cancer biology. The enriched pathways and strong associations with survival outcomes highlight the potential of these methods to identify meaningful biomarkers in multiomics data, guiding future research and, potentially, clinical translation.

### TCGA pan-cancer clustering analysis

#### Visualization of pan-cancer

**Figure 4:**
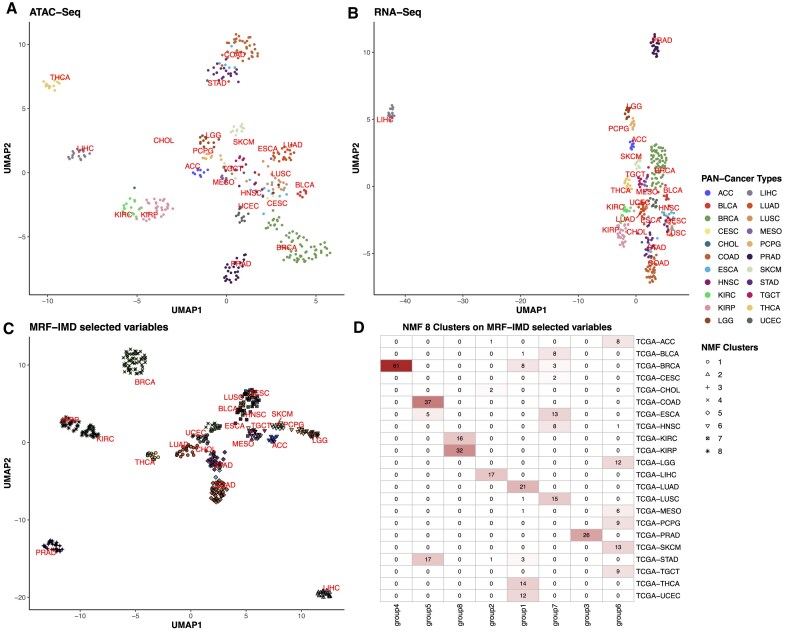
Two-dimensional UMAP embedding of TCGA pan-cancer data and confusion matrix. (A, B) UMAP of 5,000 RNA-seq and 50,000 ATAC-seq features. (C) UMAP of IntNMF embeddings of 300 RNA-seq and 186 ATAC-seq MRF-IMD prioritized features, colored by 22 pan- cancer types and shaped by the 8 IntNMF clusters. (D) Confusion matrix of 8 IntNMF clusters and 22 pan-cancer types.

To further demonstrate the flexibility and scalability of our framework, we applied the IMD-transformation variable selection method to a pan-cancer dataset from TCGA, encompassing 22 distinct cancer types. The summary of sample size and feature counts is detailed in Table 3. After processing and feature selection, we retained 186 ATAC-seq features and 300 RNA-seq features for integrative analysis using the IMD-transformation strategy. We first visualized the data using Uniform Manifold Approximation and Projection (UMAP), which revealed clear and distinct clusters that corresponded to different cancer types (Fig. 4). In the UMAP embeddings based on all ATAC-seq (Fig. 4A) or RNA-seq (Fig. 4B) features, tumor types form loose, overlapping clouds that reflect broad tissue similarities but offer limited discrimination. By contrast, the UMAP projection of the joint IntNMF embedding from MRF-IMD–prioritized ATAC and RNA features (Fig. 4C) revealed tightly bound, clearly separated clusters, demonstrating that these selected variables capture the core axes of regulatory and transcriptional variation across cancer types.

#### Pan-cancer clustering

We next applied IntNMF directly to the MRF-IMD feature set and determined an optimal rank of 8 clusters using the nmf.opt.k function from the IntNMF R package. Figure 4D shows the resulting confusion matrix, illustrating how our selected features effectively separated the samples into 8 clusters. Each cluster highlighted unique molecular characteristics and captured established patterns of tumor heterogeneity, ranging from a combined basal-like breast and uterine carcinoma cluster (group 1) through gastrointestinal adenocarcinomas (group 5) and hepatobiliary tumors (group 2) to hypermutated immunogenic cancers (group 3), squamous cell carcinomas (group 7), endocrine neoplasms (group 6), and renal epithelial tumors (group 8). A detailed description of these clusters is in Table 5. To further quantify the advantage conferred by MRF-IMD feature selection, we applied IntNMF clustering to the pan-cancer dataset, aiming for 20 clusters. We first excluded cancer types with fewer than 5 samples (TCGA-CESC and TCGA-CHOL), as very small cohorts can produce unstable clusters driven by outliers or noise. We then quantified cluster recovery by computing the Adjusted Rand Index (ARI) between the 20 IntNMF clusters on MRF-IMD features and the true TCGA tumor‐type labels (Supplementary Fig. S4a), benchmarking against 4 alternative strategies: Partitioning Around Medoids (PAM) clustering on 30 SPLS components (Supplementary Fig. S4b), IntNMF on the full feature set (Supplementary Fig. S4c), PAM clustering on RNA-seq data alone (Supplementary Fig. S4d), and PAM clustering on ATAC-seq data alone (Supplementary Fig. S4e). Although all approaches shown have a moderate to high performance, our method still shows an advantage, with a slightly higher ARI of 0.728. Nearly every TCGA cohort is assigned to a single dominant cluster: COAD (36 of 37 total samples in group 16), KIRP (31 of 32 in group 7), PRAD (25 of 26 in group 15), and UCEC (12 of 12 in group 11) all show almost perfect one-to-one mapping. In breast cancer, the algorithm distinguished 3 biologically meaningful subgroups: group 13 captured a pure basal-like subtype (all 13 basal tumors); group 5 combined all HER2 (9 of 10) and LumB (16 of 16) cases with most LumA (18 of 29) samples, reflecting a nonbasal, high-risk profile; and group 1 comprised predominantly LumA tumors (9 of 11) alongside normal controls, defining a lower-risk, luminal A–driven cluster. Smaller lineages, such as BLCA, ESCA, and STAD, likewise concentrate into their own clusters with minimal leakage. By contrast, the other methods produced more fragmented assignments and lower ARI scores, SPLS + PAM (ARI = 0.697), full-feature IntNMF (0.687), RNA-seq only (0.713), and ATAC-seq only (0.675), underscoring MRF-IMD’s superior ability to isolate coherent, biologically relevant tumor groups.

**Table 5:** Pan-cancer clustering annotations

Group	Cluster name (abbreviation)	TCGA cohorts	Key features
1	Basal-like Breast & UCEC (BRCA–UCEC)	BRCA, UCEC	High genomic instability; frequent TP53 and BRCA1/2 alterations; dysregulated DNA repair and cell cycle pathways.
2	Hepatobiliary Carcinomas (HBC)	LIHC, CHOL	Hepatic lineage tumors with altered metabolic programs and frequent TP53 mutations; cholangiocarcinoma-like epigenetic patterns.
3	Hypermutated/Immunogenic Tumors (HIM)	BLCA, SKCM	Extremely high mutational burden; strong immune infiltration signatures; enriched for PD-L1 expression and antigen presentation machinery.
4	Nonbasal Breast Cancer (Nonbasal BRCA)	BRCA	Luminal and HER2-positive subtypes; hormone receptor signaling; PI3K/mTOR pathway activation and endocrine therapy response markers.
5	Gastrointestinal Adenocarcinoma CIN (GA-CIN)	COAD, STAD, ESCA	Marked chromosomal instability (CIN); Wnt/β-catenin and TGF-β pathway dysregulation; common APC and TP53 alterations.
6	Endocrine Tumors (ENDO)	ACC, PCPG	Hormone-secreting neoplasms of adrenal cortex and chromaffin cells; endocrine axis gene dysregulation (e.g., steroidogenesis, catecholamine synthesis).
7	Squamous Cell Carcinomas (SCC)	HNSC, LUSC, ESCA, BLCA	Squamous histology tumors; frequent TP53 mutations; activation of RTK-RAS and PI3K pathways; strong epithelial-to-mesenchymal transition signatures.
8	Renal Epithelial Carcinomas (REC)	KIRC, KIRP	Clear-cell and papillary carcinomas; VHL/HIF pathway alterations; characteristic metabolic reprogramming (glycolysis, lipid metabolism).

We report ARI (chance-adjusted) rather than precision/recall because clustering is unsupervised and multiclass; ARI summarizes concordance without requiring a positive class. MRF-IMD features achieve the highest ARI among tested strategies and yield clean, tissue-coherent clusters.

By capturing these known and biologically meaningful patterns, our MRF-IMD framework shows its utility in differentiating tumor types, identifying key molecular signatures, and offering a more integrated view of tumor diversity. These results illustrate the method’s promise for guiding future studies on cancer classification, patient stratification, and uncovering novel therapeutic targets across a wide range of malignancies.

### Integrative analysis enhances prediction of dementia progression in the ADNI cohort

#### MRF-IMD–selected genes

**Figure 5:**
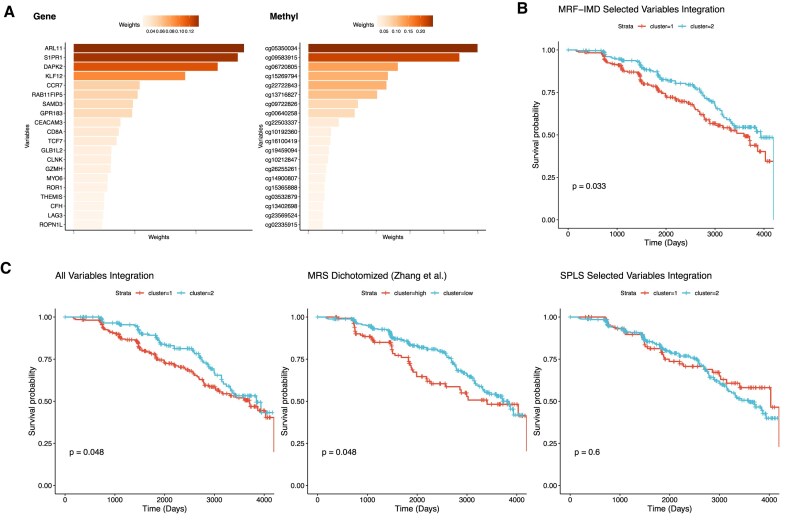
ADNI results. (A) Top 20 variable weights chosen by the IMD-mixture model. (B) stratification of Kaplan–Meier curves of MRF-IMD–selected variables using IntNMF. (C) Stratification of the full omics set integration using IntNMF (left), MRS dichotomized, cutoff determined by the MaxStat method (middle), and the first 5 SPLS components from the full omics set using IntNMF (right).

To further illustrate the superior results of our MRF-IMD method, we applied the variable selection to the ADNI data using the filtering strategy. A total of 161 genes and 54 CpG sites were selected by this strategy. Figure 5A shows the top 20 gene expression and DNA methylation features prioritized by our MRF‐IMD framework in the ADNI cohort. In the left panel, *ARL11* has the largest weights. While its direct role in AD is still under investigation, *ARL11* is known to be involved in apoptosis and immune system processes, which are critical components of neuroinflammation in AD [51]. Followed by *ARL11, S1PR1* plays a significant role in the neuroinflammatory processes of AD [52]. *DAPK2* indicates the involvement of death-associated kinase-mediated neuronal apoptosis and tau dysregulation, although the precise nature of its contribution to AD pathology requires further investigation. *CCR7* reflects its established involvement in chemokine-mediated microglial trafficking and neuroinflammation, with studies indicating that reduced *CCR7* expression on meningeal T cells in aging is linked to worsened glymphatic function, cognition, neuroinflammation, and ${\mathrm{\beta }} - $amyloid pathology [53, 54]. Functional analysis of the selected genes identified 36 pathways with FDR < 0.05 (Supplementary Table S2). The enrichment profile was dominated by lymphocyte (B cells, T cells, natural killer cells) programs, especially in T cells. Key terms included *T-Cell Differentiation and Activation, Lymphocyte Differentiation*, and *Activation*, supporting a peripheral inflammatory state relevant to AD [55, 56].

#### Pathway analysis of selected DNAm CpGs

To assess the biological relevance of our MRF-IMD–selected CpGs versus significant CpGs reported by Zhang et al. [27], we performed KEGG and GO enrichment with the missMethyl R package on both the 54 MRF-IMD prioritized sites (top 20 of 54 sites shown in Fig. 5A, right panel) and the 44 meta-analysis significant sites. Supplementary Table S3 displays the top 15 most significant pathways for each method. In the KEGG analysis, MRF-IMD CpGs showed strongest enrichment in *NF-κB Signaling, C-type Lectin Receptor Signaling*, and *Leukocyte Transendothelial Migration*. Together, these pathways indicate coordinated innate immune activation and immune cell trafficking across the endothelium, processes that escalate neuroinflammation and contribute to AD progression. These results are concordant with the pathway enrichments obtained from the selected gene set. By contrast, the Zhang et al. [27] meta-analysis CpGs were enriched for viral infection and adhesion processes. Top hits were *Virion–Ebolavirus, Lyssavirus and Morbillivirus, Cell Adhesion Molecules*, and *mTOR Signaling*, indicating a shift toward pathogen-related and cell–matrix interaction pathways.

In the GO analysis, MRF-IMD CpGs emphasized posttranscriptional RNA processing (Polyribonucleotide Nucleotidyltransferase Activity; Nuclear and Mitochondrial Polyadenylation-Dependent mRNA Catabolic Processes; Poly(U) RNA Binding), along with cytokine regulation via interleukin-1 type I/II receptor antagonist activity and neuromodulatory pathways, including Wnt signaling and galanin receptor binding (types 1–3). Notably, these CpG-derived enrichments align with the gene-based pathways through shared immune modules such as interleukin-1 receptor antagonist activity and Wnt signaling, while the RNA processing and galanin receptor terms appear CpG-specific. In contrast, the meta-analysis CpGs were dominated by cell division and cytokinesis terms (protein localization to division site, cleavage furrow, and mitotic cytokinetic regulation). These comparisons underscore that our MRF-IMD approach yields CpGs tied to innate immune signaling, mRNA processing, and metabolic regulation that were more detailed and emphasized core processes directly associated in AD.

#### Integrative validation on dementia progression

To demonstrate that our integrative variable selection outperforms both the single‐layer methylation risk score (MRS) from Zhang et al.[27] and an integrative approach without feature selection, we compared 3 stratifications of dementia conversion. We applied intNMF to the MRF-IMD–selected features, obtaining 2 clusters, and evaluated time to dementia conversion using Kaplan–Meier curves and a log-rank test. The MRF-IMD panel showed a significant separation (Fig. 5B; *P* = 0.033). We benchmarked 3 alternatives (Fig. 5C): (left) intNMF on the full, unfiltered full omics set (*P* = 0.048); (middle) the published MRS based on 151 CpGs, dichotomized at the data-driven cut-point using MaxStat (*P* = 0.048); and (right) intNMF on the first 5 SPLS components from the full omics set (*P* = 0.60). Across comparisons, the MRF-IMD hubs delivered the strongest prognostic discrimination, outperforming methylation-only scoring and unfiltered or dimension-reduced integration.

In the original Zhang et al. [27] study, the MRS was built via ridge regression on CpGs significantly associated with conversion and tested in a multivariate Cox model adjusted for age, sex, APOE $\epsilon $4 status, years of education, baseline diagnosis, and baseline Mini-Mental State Examination (MMSE) score (Surv(conversion event, follow-up) ~ MRS + covariates). When we substituted our IntNMF‐derived component from the MRF-IMD features into this identical Cox framework with the MRS, we observed a stronger association with progression to the next disease stage (Table 6). This result confirms that our integrative variable selection not only refines molecular subtyping but also enhances the prediction of disease progression beyond both single‐omics risk scores and nonprioritized integrative analyses.

**Table 6:** Results from the Cox regression model evaluating the association between the IntNMF‐derived component using the MRF-IMD prioritized variable and disease progression (CN to MCI/dementia, MCI to dementia) in 538 subjects, adjusted for age, sex, APOE ε4 status, baseline diagnosis, MMSE, and education using the ADNI dataset. Significant association was observed for the IntNMF-derived component using MRF-IMD prioritized variables (estimate = 0.201, *P* = 0.034), indicating that a higher IntNMF‐derived component increases risk.

Characteristic	Coefficient	HR (95% CI)	*P* value
**IntNMF‐derived component**	0.201	1.222 (1.016–1.471)	0.034
**Age, years**	0.063	1.071 (1.037–1.093)	3.16 × 10^−6^
**Male**	0.178	1.195 (0.834–1.714)	0.332
**Baseline diagnosis**			
CN		1 [Reference]	
MCI	0.423	1.527 (1.041–2.238)	0.03
***APOE* ε4 allele**	0.647	1.910 (1.472–2.478)	1.13 ×10^−6^
**MMSE**	−0.143	0.866 (0.772–0.972)	0.015
**Education, years**	−0.054	0.947 (0.887–1.012)	0.105

## Discussion

The continuous growth of high-throughput technologies has enabled the profiling of multiple omics layers—spanning the genome, epigenome, transcriptome, and beyond—within the same samples. This multiomics landscape offers the potential for more complete insights into disease mechanisms, biomarker discovery, and therapeutic targeting. However, integrating these disparate data types and identifying meaningful shared features remains an ongoing challenge.

In this study, we presented an MRF-based framework enhanced by the IMD metric to address these challenges. By combining the strengths of MRF for capturing nonlinear relationships and the IMD-based strategies for unsupervised feature selection, our approach provides a flexible and robust solution for multiomics integration. Unlike conventional linear methods such as SPLS and CCA, which often assume simpler data structures and can be prone to overfitting, the MRF-IMD framework scales well to complex, high-dimensional scenarios. To accommodate diverse analytical goals and user preferences, we offer 3 IMD-based selection strategies. The filter approach, selecting variables above a tunable threshold parameter, ${\mathrm{\tau }}$, gives the user direct control over signature sparsity via a standard deviation–based cutoff. For a balanced trade-off between sensitivity and specificity, the mixture strategy consistently produced intermediate-sized panels with strong predictive performance. Finally, when a broader, more exploratory feature set is desired, the transformation method excels at highlighting subtle signals by normalizing IMD distributions.

Our simulation studies showed that MRF-IMD methods consistently outperform established approaches in identifying cross-correlated variables. The results held true under diverse conditions, including linear, nonlinear, and interaction models, varying sample sizes, and multiple levels of dimensionality and noise. Notably, MRF-IMD matched the accuracy of SPLS, PMDCCA, and SGCCA in simple linear scenarios and greatly outperformed these methods as data complexity increased (Fig. 2; Table 3). Moreover, when benchmarked against popular nonlinear ensemble methods, such as univariate random forests, GBM, and XGBoost, on variable ranking tasks, our approach achieved markedly better performance (Supplementary Fig. S2). These findings highlight the robustness and adaptability of our method, reinforcing its suitability for real-world applications.

We further demonstrated the framework’s utility using multiomics data from TCGA. In breast and colorectal cancer, our approach uncovered known cancer-related genes, miRNAs, and DNA methylation features, as well as biologically relevant pathways. In breast cancer, our framework identified top gene candidates such as *FOXC1, PRR15*, and *PRKCQ*, along with key epigenetic (e.g., probes in *BCL9* and *SFT2D2*, associated with breast cancer prognosis) and miRNA features, such as miR-584–5p and let-7c-5p, that have documented roles in breast tumor progression. In colorectal cancer, the MRF-IMD–selected features likewise reflected key disease mechanisms. Top genes included *SFRP4* and *ANGPTL1*, which are both implicated in colorectal tumor aggressiveness. Our method also picked up a methylation site in the *PRAC2* locus (cg23960088), a region noted as a pan-cancer methylation biomarker in gastrointestinal and prostate tumors, indicating that the integrative approach can recover known epigenetic aberrations. These findings are well aligned with known tumor biology. In addition, an advantage of our integrative approach is evident in the pathway analysis of selected genes. In TCGA-BRCA, the MRF-IMD gene panel was highly enriched for signaling cascades central to breast cancer. For example, *PI3K/AKT Signaling* appeared as a top canonical pathway, along with several ERBB4/HER-family routes. This is consistent with the pivotal role of the PI3K–AKT–mTOR axis in driving ER-positive breast tumors and endocrine resistance [46] (Fig. 3B; Supplementary Table S1). In TCGA-COAD, the MRF-IMD–derived gene list showed strong enrichment for pathways related to the tumor microenvironment and EMT [57]. Top hits included *Extracellular Matrix Organization* and *Proteoglycan Remodeling* pathways, which align with the known importance of stromal reprogramming in colorectal cancer progression (Fig. 3B; Supplementary Table S1). It is worth emphasizing that many of these pathways were uniquely identified by MRF-IMD. Linear integrative methods and CCA/SPLS, when given a comparable number of features, often failed to enrich for these key pathways (Fig. 3C). Moreover, clustering patients based on selected variables revealed groups with distinct survival outcomes, underscoring the clinical relevance of our discoveries. Using the selected multiomic features for patient stratification yielded significant prognostic separations in both BRCA and COAD cohorts. In a pan-cancer setting, we showed that the MRF-IMD method could detect key molecular differences among diverse tumor types, identifying clusters with characteristic genomic instabilities, pathway alterations, and tissue-of-origin patterns. For instance, basal-like breast cancers clustered tightly with uterine serous carcinomas (group 1), reflecting their shared genomic profile of TP53 mutations and chromosomal instability [58]. Similarly, a cluster comprising colorectal, gastric, and esophageal adenocarcinomas emerged (group 5), consistent with the known CIN (chromosomal instability) phenotype common to gastrointestinal epithelial cancers. Other clusters aligned with established categories: we observed a grouping of squamous cell carcinomas across different organs (esophagus, lung, head/neck; group 7) characterized by TP53 mutations and RTK/RAS pathway activation, as well as a clear cluster of renal cell tumors (group 8) distinguished by their unique metabolic and microenvironment profiles. These results suggest that the approach has broad applicability, enhancing our understanding of tumor heterogeneity and potential therapeutic targets. Furthermore, clustering on MRF-IMD features achieved a high ARI relative to true tumor types, outperforming clustering based on all features or features from linear dimension reduction. In practical terms, our integrative selection could potentially facilitate tumor classification and subtyping in large heterogeneous datasets, focusing attention on the most informative genomic and epigenomic markers while filtering out noisy, uninformative variables.

In the ADNI cohort, MRF-IMD again proved its merit by uncovering biologically plausible and prognostically relevant markers of cognitive decline. Unlike cancer, where tissue-specific pathways dominate, Alzheimer’s disease involves complex systemic and brain processes. Our method highlighted genes such as *ARL11, S1PR1, CCR7*, and *DAPK2* among the top candidates. These findings illustrate how multiomics integration can spotlight targets that are not the most significant in any single data source but are critical when considering disease pathways together. Moreover, our integrative approach improved the prediction of clinical outcomes in the ADNI cohort. We compared our multiomics signature to a published MRS based on 151 CpGs from a large epigenome meta-analysis. Whereas the MRS alone did stratify patients to some degree, the MRF-IMD integrative signature achieved a more significant separation between progressors and nonprogressors (by Kaplan–Meier analysis). In fact, when we incorporated the integrative component into a Cox regression (mirroring the original study’s covariate-adjusted model), it yielded a stronger association with time to dementia than the MRS. This suggests that combining gene expression with methylation (guided by MRF-IMD to focus on the most relevant features) captures a more predictive composite biomarker of cognitive decline.

In summary, our method is particularly well suited for analyzing complex, cross-layer, and high-dimensional datasets, such as those encountered in cancer and neurodegenerative disorders. It also stabilizes “small-n, large-p” analyses, common in rare disease studies or clinical trials, by reducing the feature space to the most reproducible cross-omics signals. MRF-IMD is capable of uncovering biomarkers and regulatory patterns that may be missed by traditional linear methods, especially when nonlinear patterns or interactions are present. To ensure computational tractability and model convergence, we recommend a preliminary variable-filtering step, such as retaining the most variable features per layer (e.g., under 5,000 features). In downstream tasks like molecular subtyping and survival prediction, MRF-IMD produces compact, interpretable biomarker panels that achieve more robust patient stratification than single-omics approaches, as demonstrated across the TCGA-BRCA, TCGA-COAD, pan-cancer, and ADNI cohorts.

Our framework is most advantageous when (i) multiple omics layers share samples, (ii) cross-layer dependencies may be **nonlinear** or involve **interactions**, (iii) labels are scarce (unsupervised discovery), and (iv) stability/interpretability of selected features matter. In linear regimes with strong sparsity, SPLS/CCA can perform very well as our simulations confirm, but they degrade in nonlinear settings, where MRF-IMD maintains high PR-AUC and stable recovery. Conversely, deep learning models often require labels and do not provide transparent unsupervised importance, making them less suitable for our selection goal.

While our method provides clear advantages, some limitations remain. First, computation time may increase with more datasets and extreme high-dimensionality. Future research could focus on improving efficiency, potentially through parallelization or dimensionality reduction strategies that preserve essential biological signals. Second, further integration with downstream validation steps, such as experimental verification or functional assays, would help confirm the biological significance of the selected variables and strengthen the evidence for potential biomarkers. Third, although we prioritize cross-layer shared biomarkers to capture system-level regulators, we recognize that unique, layer-specific features (e.g., methylation marks reflecting environmental exposure or miRNAs mediating posttranscriptional control) also carry important biological information. Future work in developing a promising extension of the framework that extracts both omics-specific and shared biomarkers would enable more comprehensive biological insights. Fourth, we acknowledge that we did not benchmark MRF-IMD against deep learning–based integrative frameworks, a limitation of our current evaluation. Deep neural architectures (e.g., graph convolutional networks or variational autoencoders) have shown promise for multiomics integration, yet they are typically supervised, rely on large labeled datasets, and lack transparent, unsupervised feature‐importance mechanisms. Consequently, they cannot be directly applied to our unsupervised, multiresponse variable‐selection setting without substantial adaptation. Future work comparing our framework with unsupervised or semi-supervised deep learning methods could further enrich the benchmarking and broaden the understanding of integrative strategies. Finally, additional studies are needed to assess the utility of MRF-IMD–selected features in downstream applications, such as tumor subtype clustering, survival prognosis, and treatment-response prediction, to fully demonstrate the translational potential of our integrative selection strategy.

In conclusion, our MRF-IMD framework represents a step forward in integrative multiomics analysis. By balancing flexibility, scalability, interpretability, and robustness, it enables researchers to identify meaningful biomarkers and pathways that would be difficult to pinpoint using conventional approaches. As the field continues to generate increasingly complex data, methods like ours will be instrumental in translating multiomics information into actionable insights that advance our understanding of health and disease.

## Availability of Supporting Source Code and Requirements

Project name: An Integrative Multiomics Random Forest Framework for Robust Biomarker Discovery

Project homepage: https://github.com/TransBioInfoLab/multiRF-vs

Vignette: https://rpubs.com/noblegasss/multiRF-vs-vignette

Operating system: Linux (Ubuntu)

Programming language: R version 4.4.2

License: GPL 3.0 or higher

## Supplementary Material

giaf148_Supplemental_Files

giaf148_GIGA-D-25-00021_Original_Submission

giaf148_GIGA-D-25-00021_Revision_1

giaf148_Reviewer_1_Report_Original_Submission Moran Chen -- 3/6/2025

giaf148_Reviewer_2_Report_Revision_1 Yun-Juan Bao -- 11/16/2025

giaf148_Revision_2_Report_Original_Submission Yun-Juan Bao -- 3/16/2025

giaf148_Revision_3_Report_Original_Submission Yingxia Li -- 4/27/2025

## Data Availability

All TCGA datasets (mRNA, miRNA, DNA methylation, and ATAC-seq) used in this study were obtained from UCSC Xena [23–26]. TCGA RNA-seq datasets were accessed using the TCGAbiolinks R package [59]. ADNI datasets were downloaded from the ADNI database under standard data-use agreements [27]. Scripts for data preprocessing are available in the manuscript’s GitHub repository [60].

## Additional Files

**Supplementary Fig. S1**. (a) Modeling forest IMD with Gaussian mixture (blue curve) and truncated normal mixture (orange curve) models. The data are simulated by the latent model with the settings of *p* = *q* = 500, *n* = 200, and the first 30 variables of each dataset are cross-correlated with each other. The forest IMD has a skewed distribution, and the Gaussian mixture model fits better than the truncated normal mixture in this scenario. (b) Distribution of IMD of cross-correlated and noise variables. Top is original forest IMD of noise variables that skews heavily toward the lower end of the IMD scale, clustering near zero. In contrast, the cross-correlated variables exhibit a broader distribution, starting from zero, although this is less apparent due to their sparsity. Bottom is *t*-score forest IMD effectively differentiates between noise and cross-correlated variables. Noise variables cluster below the mean (μ), while cross-correlated variables significantly diverge from μ.

**Supplementary Fig. S2**. Boxplots of ranking-based simulation results. Performance is measured by PR-AUC. The top left shows results of the latent model, with each boxplot representing 3 different dimensionality scenarios (left to right). Results indicate that IMD measures outperform other nonlinear importance measures and are competitive with linear integrative methods. The right panel displays 2 different parameter settings of nonlinear regression simulation, where IMD scores outperform all other methods. The bottom left shows results of the 3-way interaction model, where IMD scores again demonstrate superior performance compared to all other methods.

**Supplementary Fig. S3**. Sensitivity analysis results show the stability of selections across 30 MRF-IMD runs using TCGA-BRCA (top) and TCGA-COAD (bottom). Results are summarized by model size and pairwise overlap coefficient (intersection divided by the smaller set size). These findings demonstrate that, regardless of cohort or omics layer, IMD-transformation offers the best trade‐off between breadth and consistency, IMD-filter delivers the most parsimonious yet stable core signature, and IMD-mixture provides a middle ground in both model size and overlap.

**Supplementary Fig. S4**. Confusion matrix comparing 20 pan-cancers against (a) NMF 20 groups using MRF-IMD selected variables, (b) SPLS 30 components with 20 groups, (c) NMF 20 groups using all variables, (d) RNA-seq 20 groups, and (e) ATAC-seq 20 groups.

**Supplementary Fig. S5**. An illustration of a tree in the MRF model. The datasets *X* and *Y* were generated using the latent model (details in simulation study), with the first 20 variables of each dataset being cross-correlated. The variables *Y_1_* to *Y_20_* are cross-correlated with variables *X_1_* to *X_20_*. Cross-correlated variables from *Y* are colored in yellow. Nearly half of the MSRVs identified at each node are cross-correlated variables.

**Supplementary Fig. S6**. Boxplot of selection frequency of variables in ***Y*** selected as MSRV. The red box (left) represents the selection frequency of cross-correlated variables, and the blue box (right) represents the selection frequency of noise. (a) Scenario 1: *q* = 200. (b) Scenario 2: *q* = 500. In both scenarios, cross-correlated variables are selected as MSRV more frequently than noise variables.

**Supplementary Fig. S7**. Impact of tree number on IMD. The top row shows boxplots of IMD values for models built with varying number of trees (50, 100, 300, 500, and 1,000). In both datasets, cross-correlated variables (shown in red) consistently have higher IMD values compared to noise variables (shown in blue), highlighting the greater importance of cross-correlated variables in the models. The bottom row shows the PR curve presented for each tree setting. The PRAUC improves when ntree increases.

**Supplementary Fig. S8**. Impact of tree depth on IMD. The top row displays boxplots of IMD values across different node depths (1, 2, 3, 5, 10, and Full). For both datasets, the mean and variation of IMD values for both cross-correlated and noise variables increase as tree depth grows. The bottom row shows the PR curve presented for each node depth setting. These results indicate that deeper trees improve variable important representation for dataset ***Y***, while dataset ***X*** maintains high variable important representation, even in very shallow trees.

**Supplementary Fig. S9**. Impact of proportion of response variables on IMD. The top row displays boxplots of IMD values across a different proportion of response variables. The bottom row shows the PR curve presented for each proportion setting. The results suggest that dataset ***Y*** is more sensitive to the proportion of included variables, impacting its overall PR-AUC performance more significantly than dataset ***X***.

**Supplementary Fig. S10**. Error plots of the values of IMD and MD. MD shows increased variation for noise variables (in blue) across all scenarios, whereas IMD remains stable and close to zero, demonstrating consistent performance regardless of noise.

**Supplementary Fig. S11**. Compare the values of IMD and MD with the true labels of the predictors. Left: Precision-recall (PR) curve derived from 50 replications. Right: Boxplot of PR-AUC obtained from each replication. The difference in performance of IMD and MD is supported by a Wilcoxon signed-rank test, which yields a significant *P* value of 0.00042.

**Supplementary Table S1**. Significant pathways enriched with MRF-IMD-selected genes for BRCA and COAD.

**Supplementary Table S2**. Significant pathways enriched in MRF-IMD-selected genes for ADNI.

**Supplementary Table S3**. Top 15 pathways found by the MRF-IMD priority CpG sites using missMethyl.

## Abbreviations

AD: Alzheimer’s disease; ADNI: Alzheimer’s Disease Neuroimaging Initiative; ARI: Adjusted Rand Index; ATAC-seq: ATAC sequencing; BRCA: breast invasive carcinoma; CCA: canonical correlation analysis; CN: cognitively normal; COAD: colon adenocarcinoma; CRC: colorectal cancer; ECM: extracellular matrix; EM: expectation–maximization; EMT: epithelial–mesenchymal transition; ER: estrogen receptor; FDR: false discovery rate; GBM: gradient boosting machine; GO: Gene Ontology; IMD: inverse minimal depth; IntNMF: integrative nonnegative matrix factorization; KEGG: Kyoto Encyclopedia of Genes and Genomes; MCI: mild cognitive impairment; MRF: multivariate random forest; MRS: methylation risk score; MSRV: maximal splitting response variable; OOB: out-of-bag; PCA: principal component analysis; PMDCCA: penalized matrix decomposition canonical correlation analysis; PR-AUC: area under the precision-recall curve; RF: random forest; RNA-seq: RNA sequencing; SGCCA: sparse regularized generalized canonical correlation analysis; SPLS: sparse partial least squares; TCGA: The Cancer Genome Atlas; TPM: transcripts per million; UMAP: Uniform Manifold Approximation and Projection.
