# Supplementary material for: An integrative multiomics random forest framework for robust biomarker discovery
Source: Gigascience. 2025 Dec 9;15:giaf148. doi: 10.1093/gigascience/giaf148 (PMC12821379; doi:10.1093/gigascience/giaf148)
Supplement: giaf148_Supplemental_Files [file giaf148_supplemental_files.zip › Supplementary Figures.pdf]

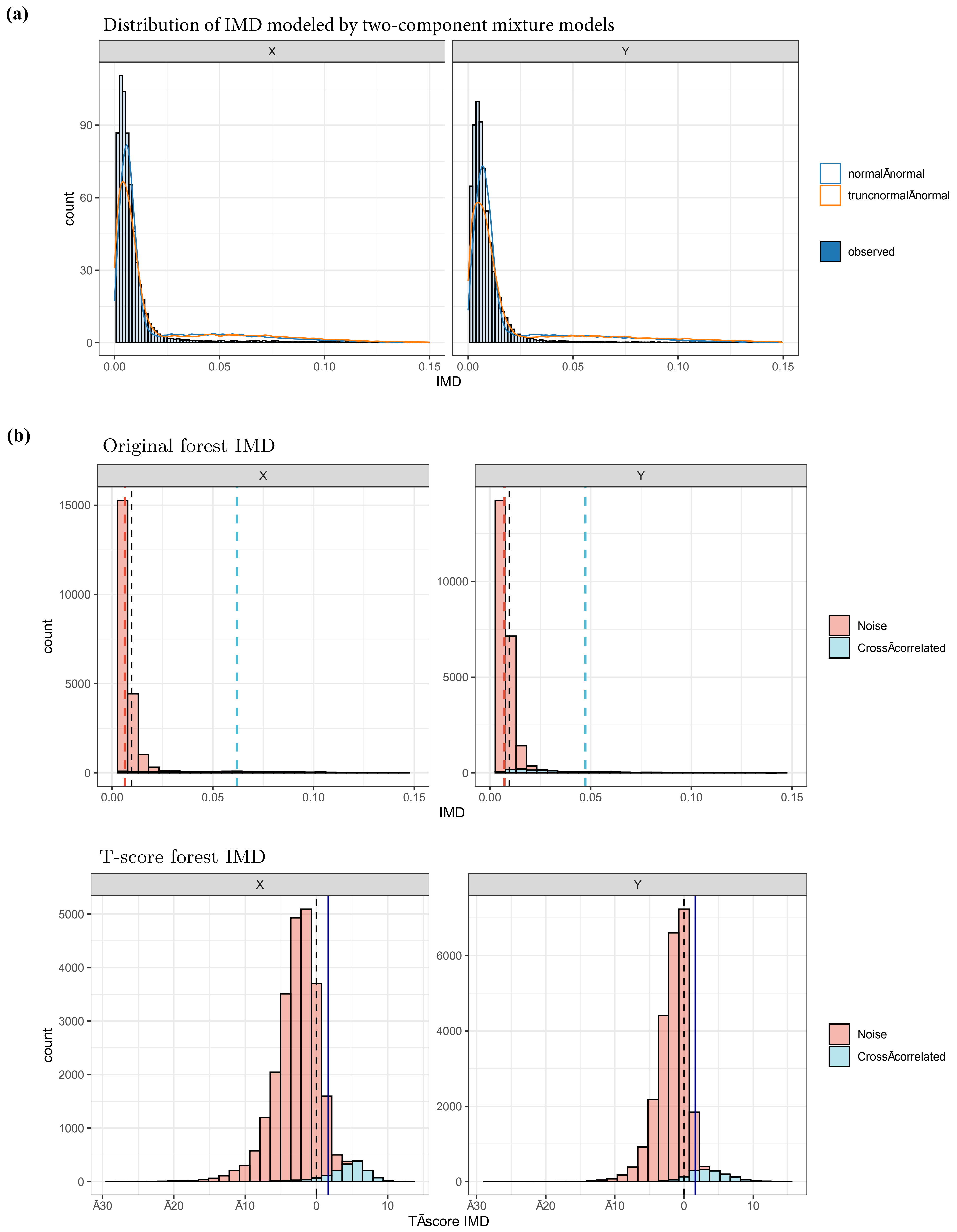

Supplementary Figure 1 (a) Modeling forest IMD with Gaussian mixture (blue curve) and truncated normal mixture (orange curve) models. The data is simulated by the latent model with the settings of $\boldsymbol{p}$ = q= 500, n = 200, and the first 30 variables of each dataset are cross-correlated with each other. The forest IMD has a skewed distribution, and the Gaussian mixture model fits better than the truncated normal mixture in this scenario. **(b)** Distribution of IMD of cross-correlated and noise variables. Top is original forest IMD of noise variables skews heavily toward the lower end of the IMD scale, clustering near zero. In contrast, the cross-correlated variables exhibit a broader distribution starting from zero, although this is less apparent due to their sparsity. Bottom is t-score forest IMD effectively differentiates between noise and cross-correlated variables. Noise variables cluster below the mean (μ), while cross-correlated variables significantly diverge from μ.

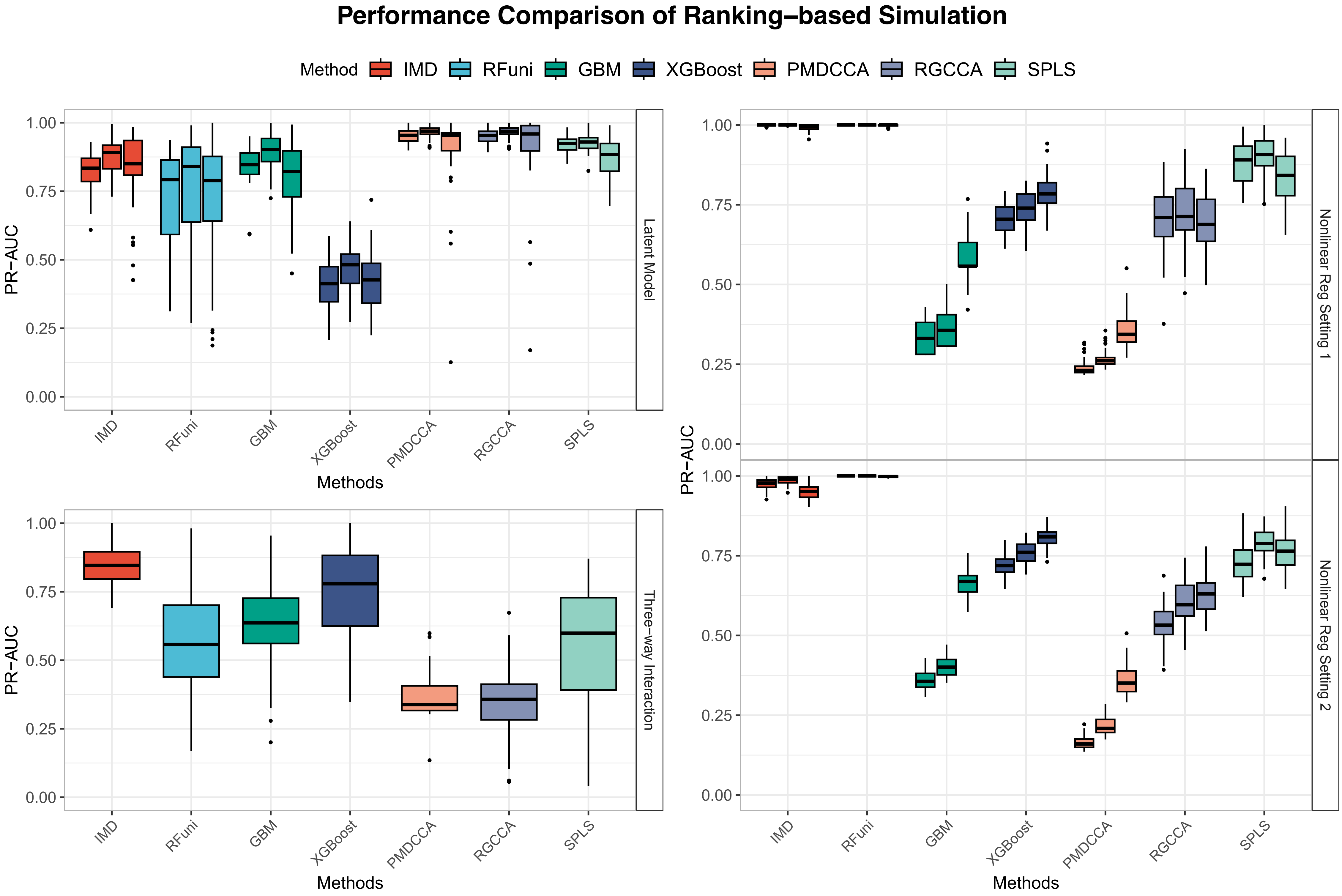

Supplementary Figure 2 Boxplots of ranking-based simulation results. Performance is measured by PR-AUC. The top left shows results of the latent model, with each boxplot representing three different dimensionality scenarios (left to right). Results indicate that IMD measures outperform other non-linear importance measures and are competitive with linear integrative methods. The right panel displays two different parameter settings of non-linear regression simulation, where IMD scores outperform all other methods. The bottom left shows results of the three-way interaction model, where IMD scores again demonstrate superior performance compared to all other methods.

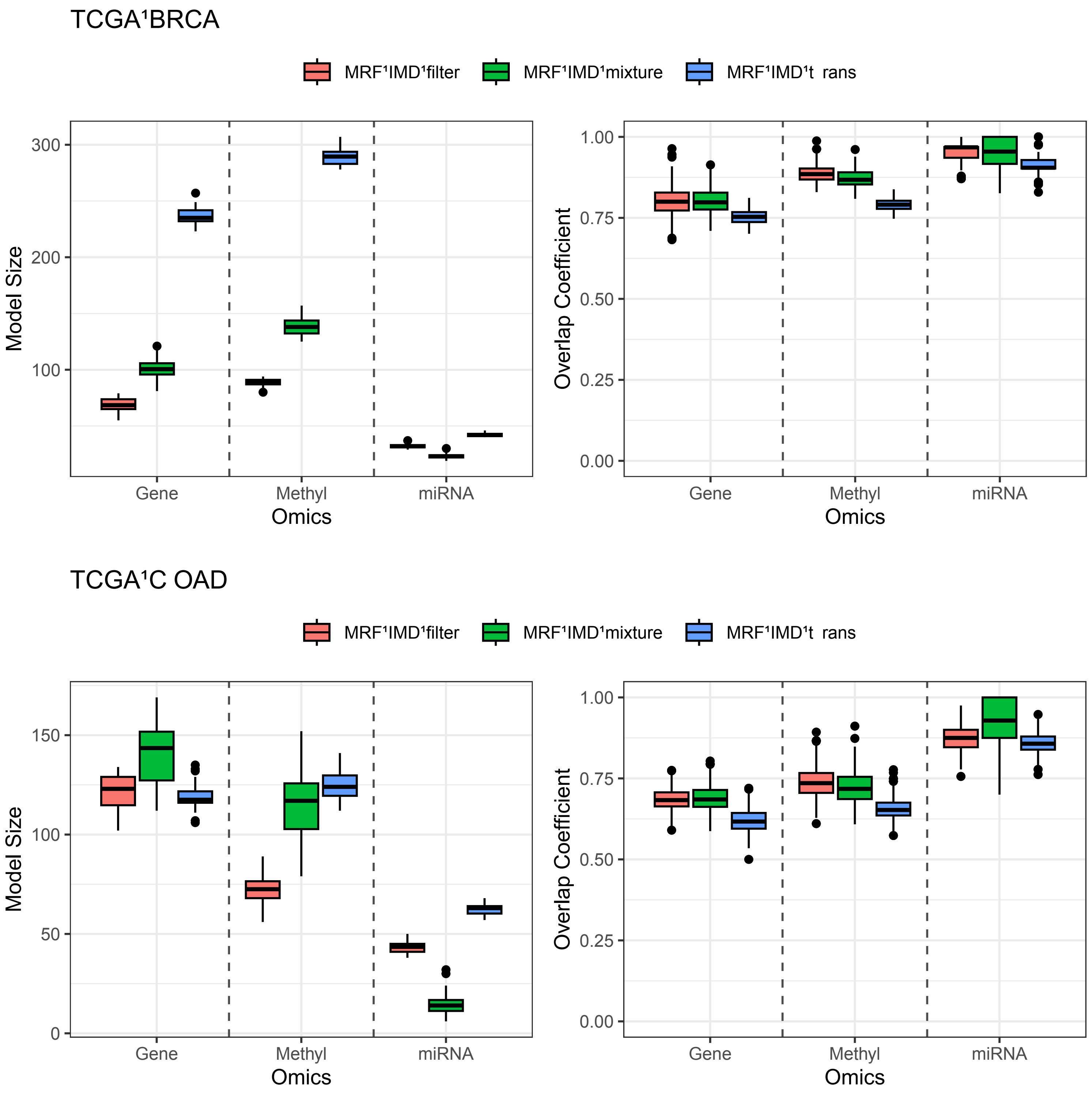

Supplementary Figure 3 Sensitivity analysis results show the stability of selections across 30 MRF-IMD runs using TCGA-BRCA (top) and TCGA-COAD (bottom). Results are summarized by model size and pairwise overlap coefficient (intersection divided by the smaller set size). These findings demonstrate that, regardless of cohort or omics layer, IMD-transformation offers the best trade‐off between breadth and consistency, IMD-filter delivers the most parsimonious yet stable core signature, and IMD-mixture provides a middle ground in both model size and overlap.

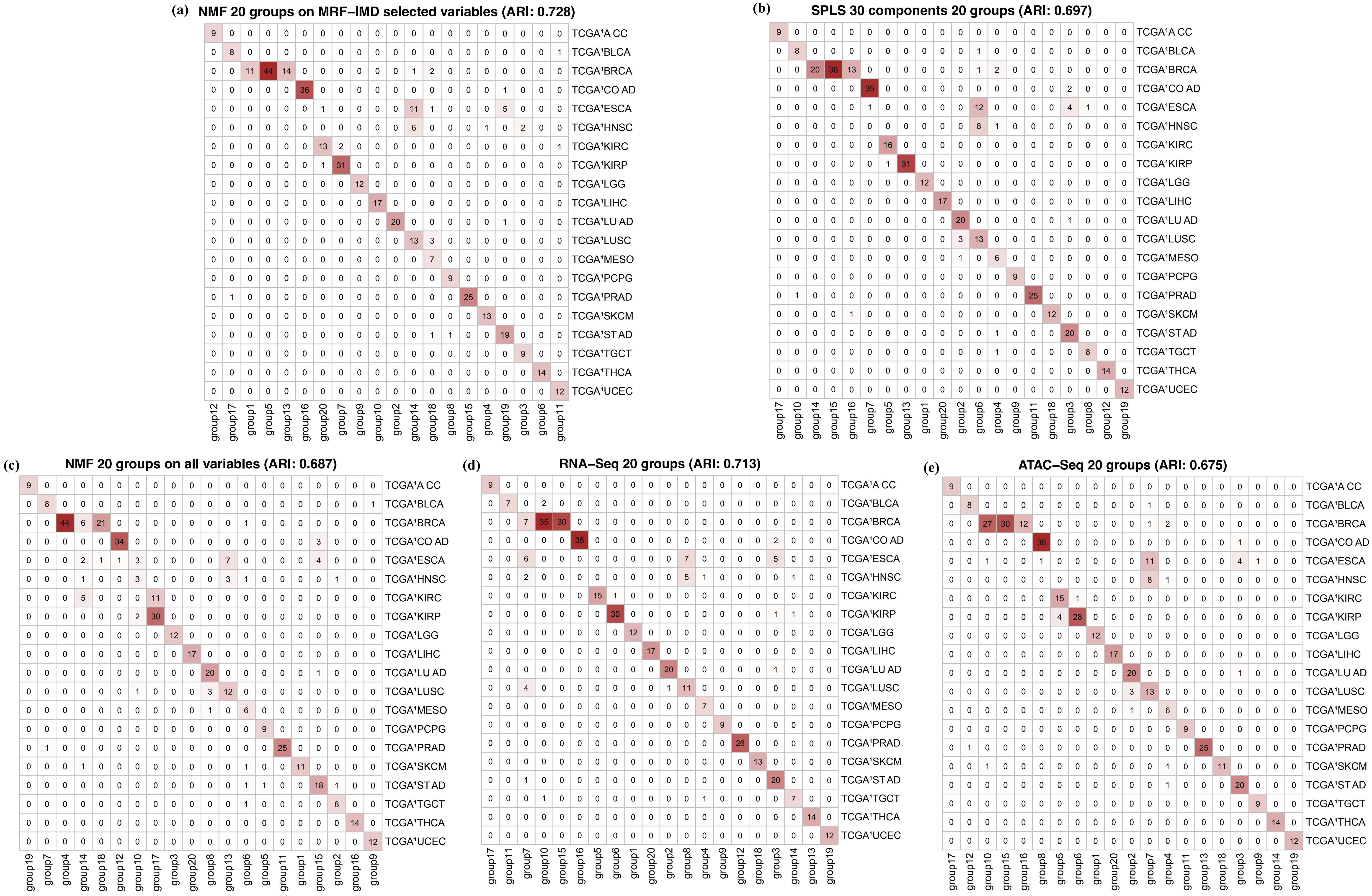

Supplementary Figure 4 Confusion matrix comparing 20 Pan-cancers against: (a) NMF 20 groups using MRF-IMD selected variables, (b) SPLS 30 components with 20 groups, (c) NMF 20 groups using all variables, (d) RNA-Seq 20 groups, and (e) ATAC-Seq 20 groups.

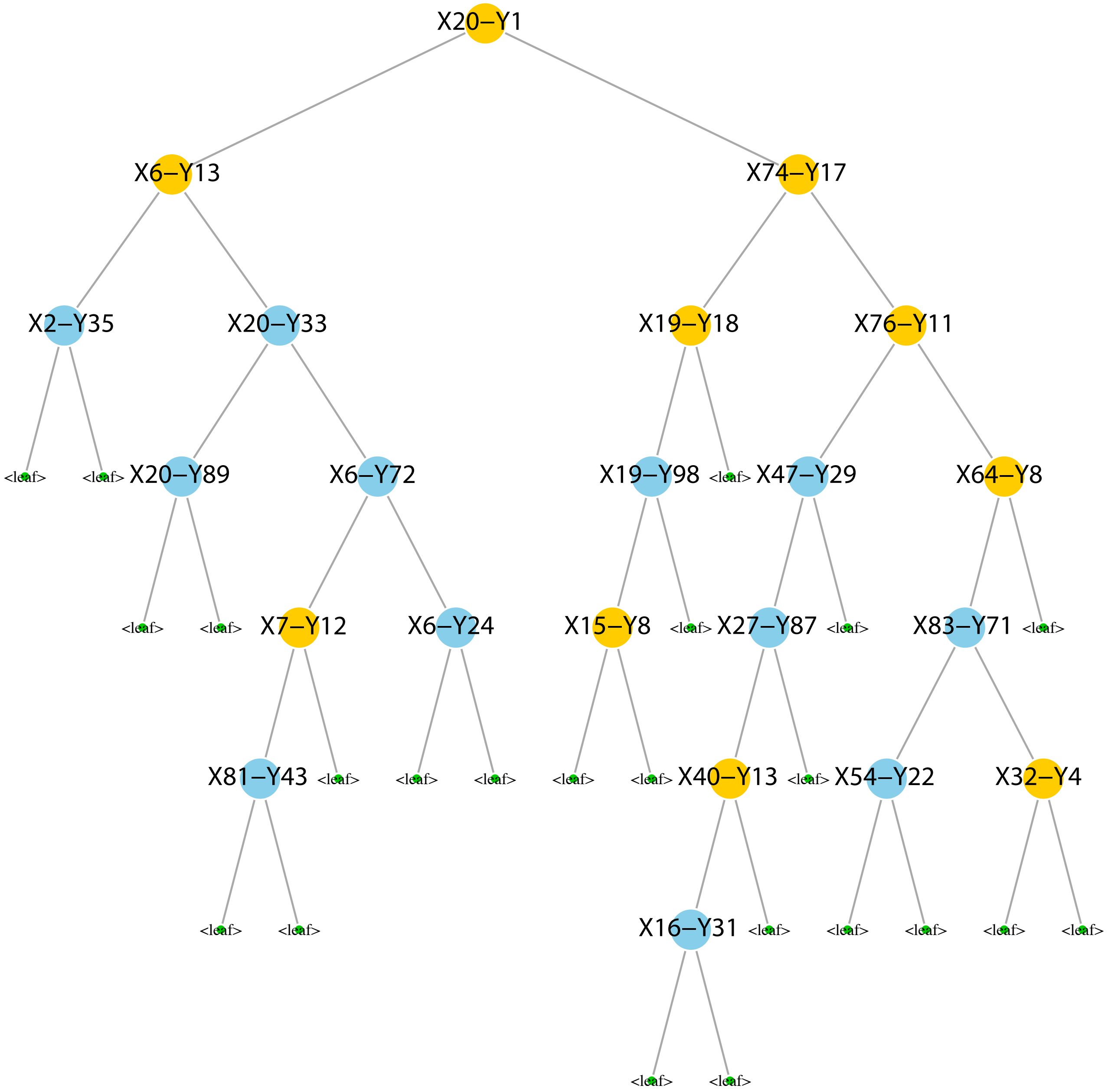

Supplementary Figure 5 An illustration of a tree in MRF model. The datasets X and Y were generated using the latent model (Details in Simulation Study), with the first 20 variables of each dataset being cross-correlated. The variables Y_1 to Y_{20} are cross-correlated with variables X_1 to X_{20}. Cross-correlated variables from Y are colored in yellow. Nearly half of the MSRVs identified at each node are cross-correlated variables from .

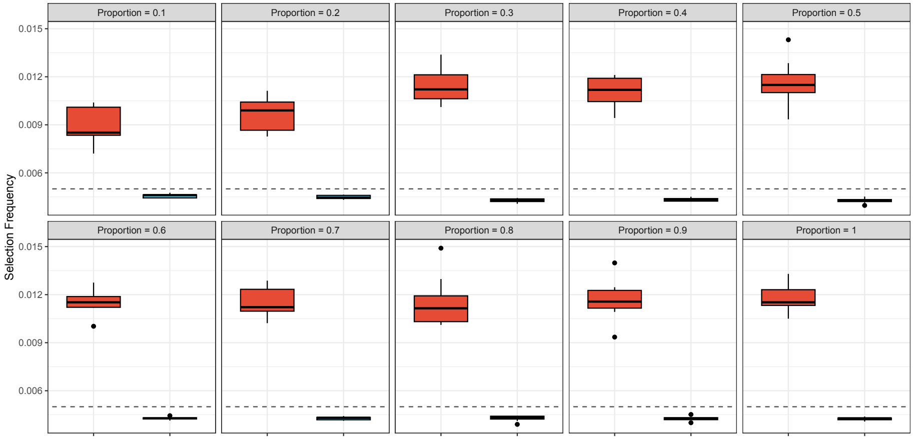

(a) Scenario 1: $q = 200$

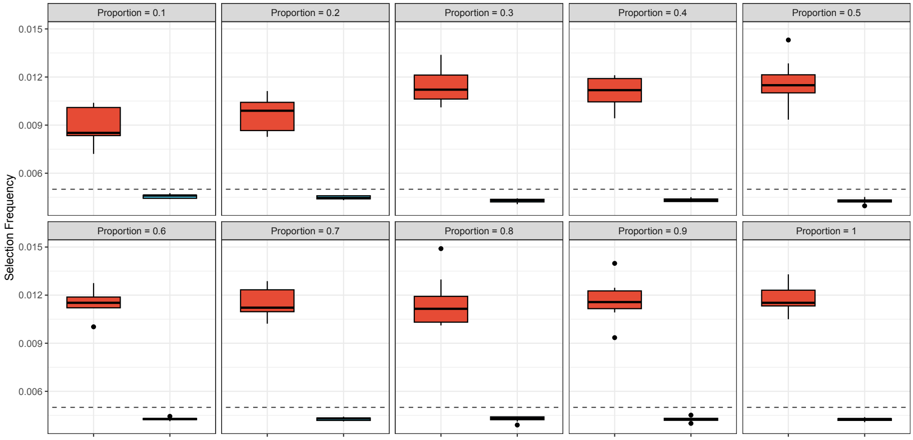

(b) Scenario 2: $q = 500$

Supplementary Figure 6 Box plot of selection frequency of variables in ***Y*** selected as MSRV. The red box (left) represents selection frequency of cross-correlated variables and blue box (right) represents selection frequency of noise. (a) Scenario 1: q = 200 (b) Scenario 2: q = 500. In both scenarios, cross-correlated variables are selected as MSRV more frequently than noise variables.

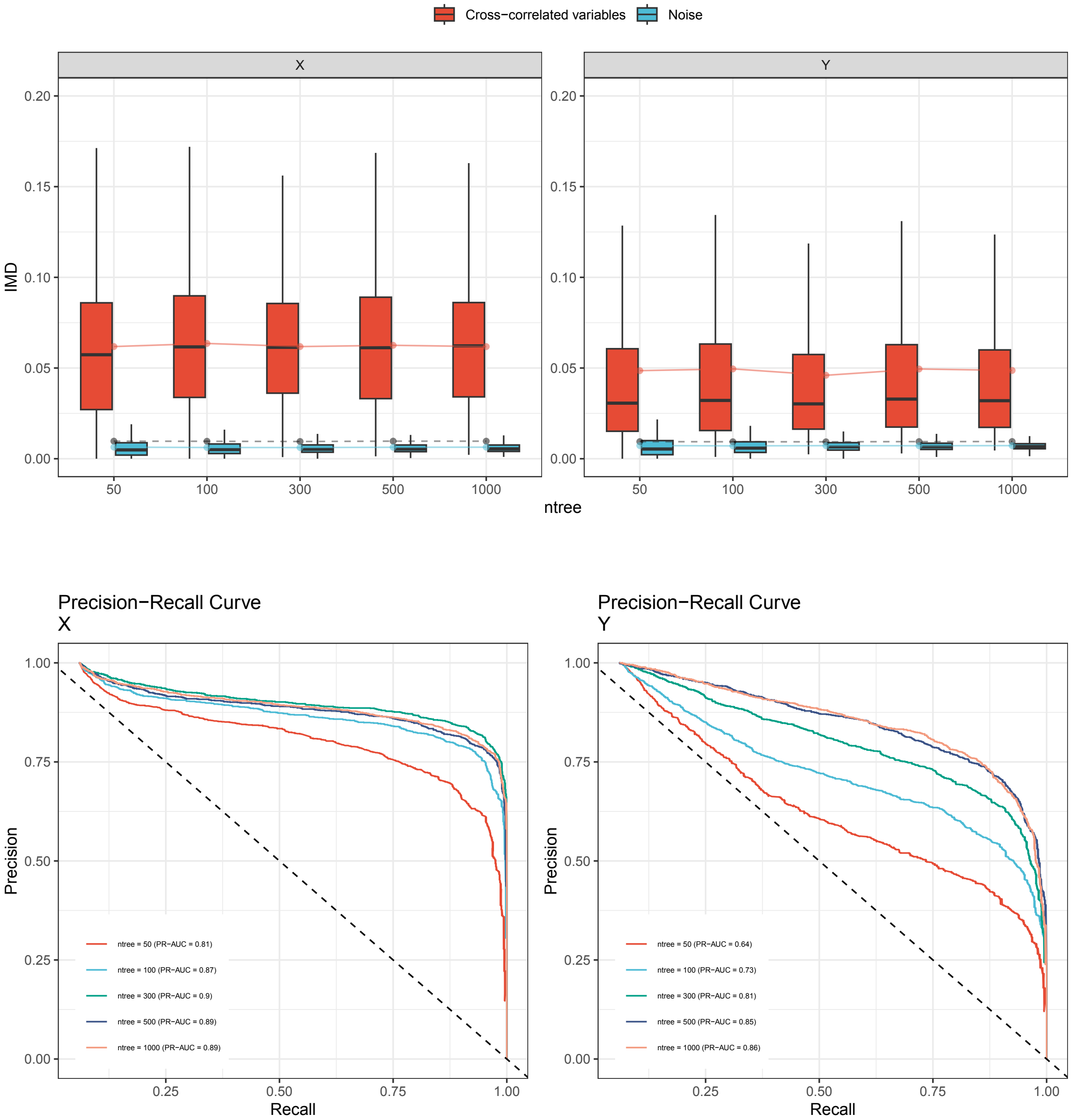

Supplementary Figure 7 Impact of tree number on IMD. The top row shows box plots of IMD values for models built with varying number of trees (50, 100, 300, 500, and 1000). In both datasets, cross-correlated variables (shown in red) consistently have higher IMD values compared to noise variables (shown in blue), highlighting the greater importance of cross-correlated variables in the models. The bottom row shows the PR curve presented for each tree setting. The PRAUC improves when ntree increases.

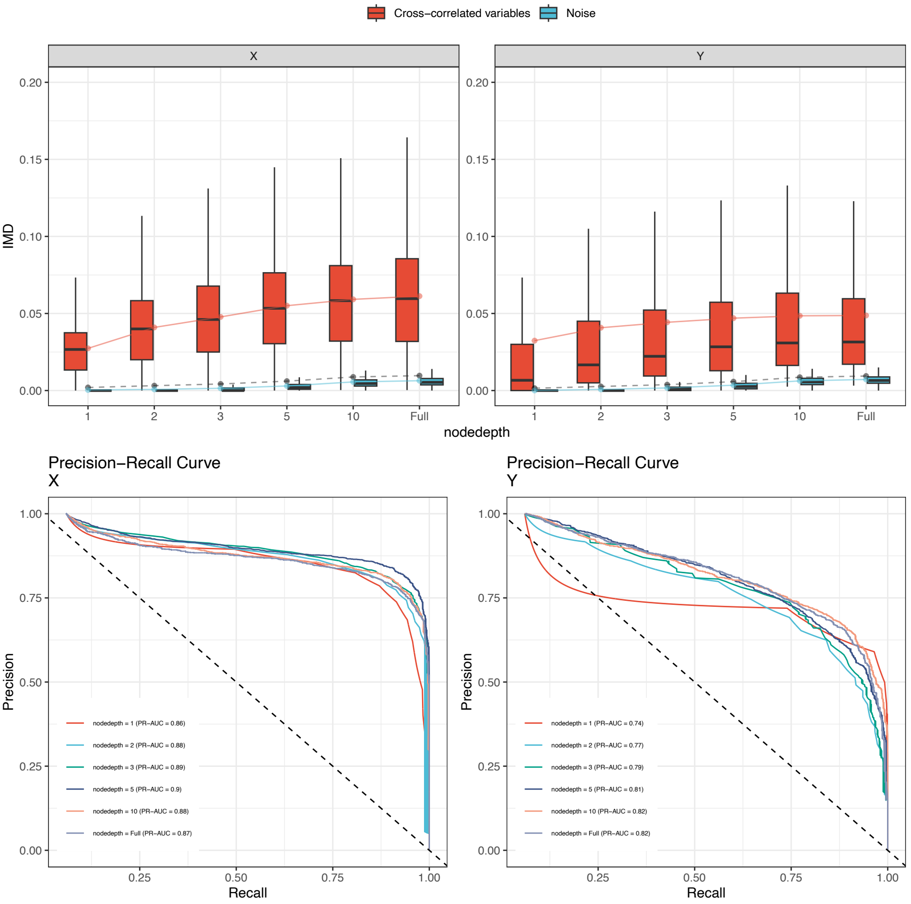

Supplementary Figure 8 Impact of tree depth on IMD. The top row displays box plots of IMD values across different node depths (1, 2, 3, 5, 10, and Full). For both datasets, the mean and variation of IMD values for both cross-correlated and noise variables increase as tree depth grows. The bottom row shows the PR curve presented for each node depth setting. These results indicate that deeper trees improve variable important representation for dataset ***Y***, while dataset ***X*** maintains high variable important representation even in very shallow trees.

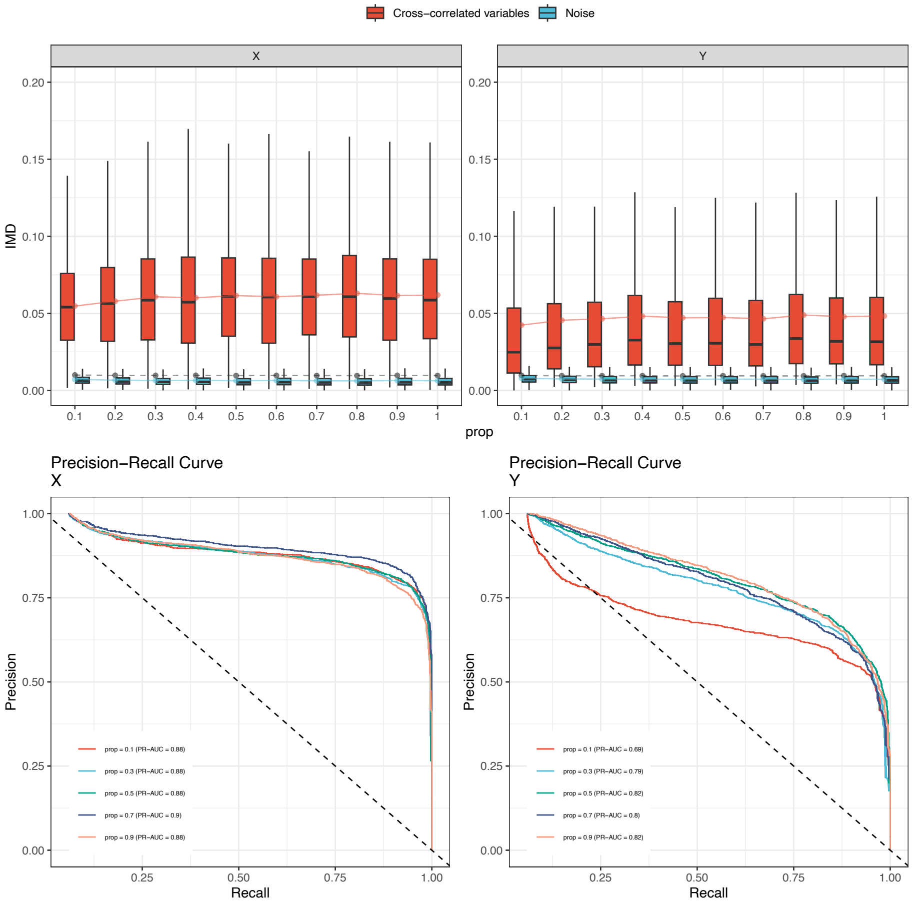

Supplementary Figure 9 Impact of proportion of response variables on IMD. The top row displays box plots of IMD values across different proportion of response variables. The bottom row shows the PR curve presented for each proportion setting. The results suggest that dataset ***Y*** is more sensitive to the proportion of included variables, impacting its overall PR-AUC performance more significantly than dataset ***X***.

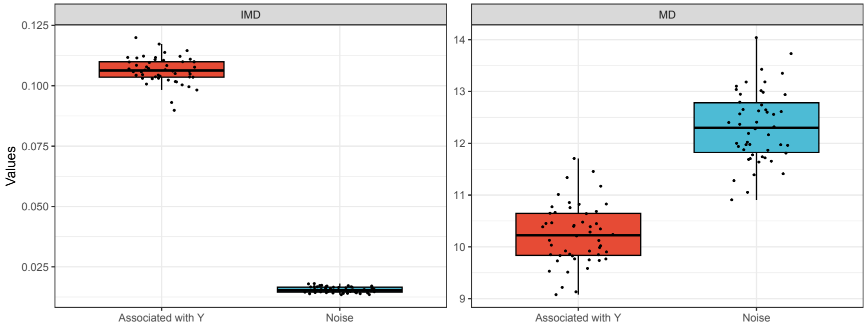

(a) Scenario 1: $q = 200$

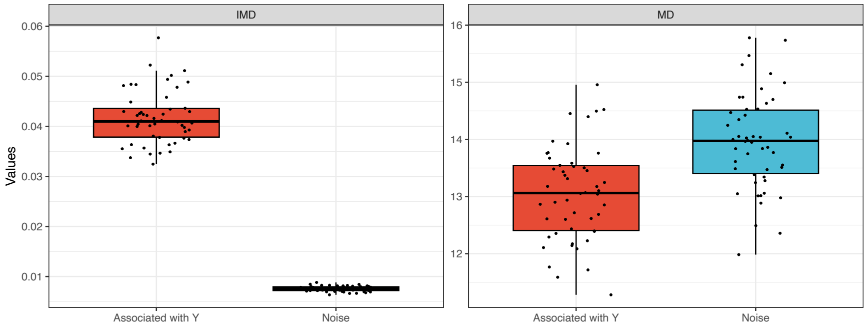

(b) Scenario 2: $q = 500$

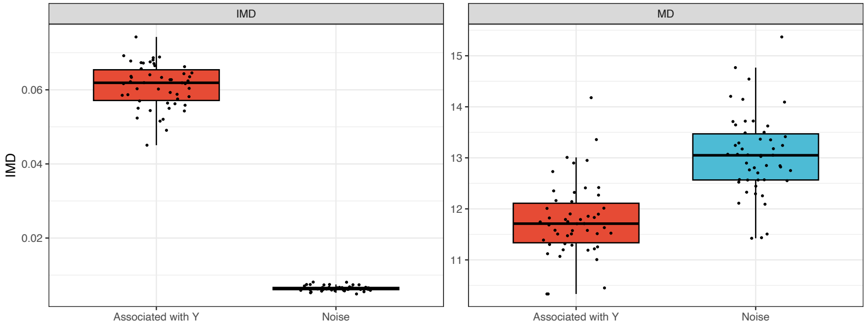

(c) Scenario 3: $q = 500$ with noise in $\boldsymbol{Y}$

Supplementary Figure 10 Errorplots of the values of IMD and MD. MD shows increased variation for noise variables (in blue) across all scenarios, whereas IMD remains stable and close to zero, demonstrating consistent performance regardless of noise.

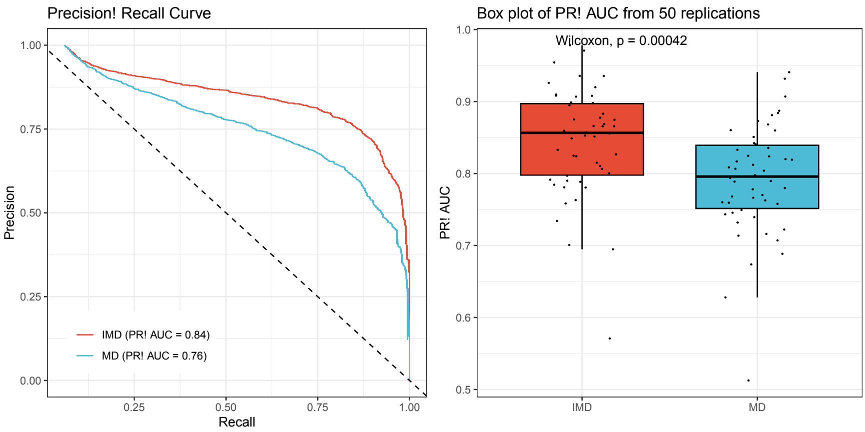

Supplementary Figure 11 Compare the values of IMD and MD with the true labels of the predictors. Left: Precision-recall (PR) curve derived from 50 replications; Right: Box plot of PR-AUC obtained from each replication. The difference in performance of IMD and MD is supported by a Wilcoxon signed-rank test, which yields a significant p-value of 0.00042.
