# Supplementary material for: An integrative multiomics random forest framework for robust biomarker discovery
Source: Gigascience. 2025 Dec 9;15:giaf148. doi: 10.1093/gigascience/giaf148 (PMC12821379; doi:10.1093/gigascience/giaf148)

GigaScience

An Integrative Multi-Omics Random Forest Framework for Robust Biomarker Discovery

--Manuscript Draft--

Manuscript Number:	GIGA-D-25-00021R1
Full Title:	An Integrative Multi-Omics Random Forest Framework for Robust Biomarker Discovery
Article Type:	Research
Funding Information:	National Cancer Center (R01CA200987) — Peofessor Xi Steven Chen
Abstract:	High-throughput technologies now produce a wide array of omics data, from genomic and transcriptomic profiles to epigenomic and proteomic measurements. Integrating multiple omics layers measured on the same samples can reveal cross-layer molecular hubs that single-layer analyses miss. We present an unsupervised, multivariate random forest (MRF) framework with an inverse minimal depth (IMD) importance to prioritize shared biomarkers across omics. In each forest, one layer serves as a multivariate response and the other as predictors; IMD summarizes how early a predictor (or response MSRV) appears across trees, yielding interpretable, cross-layer feature rankings. We provide three IMD-based selection strategies and introduce an optional IMD power transform to enhance sensitivity to interaction signals. In extensive simulations spanning linear, nonlinear, and interaction regimes, our method matches SPLS/CCA under linear settings and outperforms them as nonlinearity increases, while adapted univariate ensemble learners (RF, GBM, XGBoost) underperform in the multivariate, unsupervised context. Applied to TCGA BRCA and COAD, MRF-IMD identifies genes, CpGs, and miRNAs enriched for cancer-relevant pathways and yields more robust survival stratification than linear integrators with matched model sizes. In a TCGA pan-cancer analysis, MRF-IMD features achieve higher ARI than alternatives and recover coherent tumor-type clusters; in ADNI, the integrative signature improves dementia-progression stratification over a published methylation risk score. Our scalable, interpretable MRF-IMD framework advances reliable multi-omics biomarker discovery when nonlinear, cross-layer dependencies matter..
Corresponding Author:	Xi Steven Chen<br>University of Miami Miller School of Medicine: University of Miami School of Medicine<br>Miami, UNITED STATES
Corresponding Author Secondary Information:	
Corresponding Author's Institution:	University of Miami Miller School of Medicine: University of Miami School of Medicine
Corresponding Author's Secondary Institution:	
First Author:	Xi Steven Chen
First Author Secondary Information:	
Order of Authors:	Xi Steven Chen
	Wei Zhang
	Hanchen Huang
	Lily Wang
	Brian D. Lehmann
Order of Authors Secondary Information:	
Response to Reviewers:	Reviewer #1<br>This manuscript presents a novel multivariate random forest (MRF) framework enhanced by the inverse minimal depth (IMD) metric for integrative multi-omics biomarker discovery. The authors clearly demonstrate the robustness and superiority of the proposed methods through comprehensive simulation studies and validation on TCGA datasets. The manuscript provides clear methodological explanations, offering

valuable insights into its practical utility. I recommend accepting the manuscript after minor revisions.

We sincerely thank the reviewer for their positive assessment of our framework and their encouraging feedback. In response to the comments, we have made the following updates to enhance the clarity and reproducibility of our manuscript. All changes are marked in red in the revised version for ease of review. We believe these revisions address the reviewer's suggestions and further strengthen the presentation of our work. Thank you once again for your thoughtful comments.

Minor Concern:

1. Biological Interpretation Depth: While the authors identified biologically relevant biomarkers, the biological interpretations remain somewhat superficial. A deeper exploration of novel or less-known biomarkers in the context of disease mechanisms would strengthen the biological relevance of the findings.

We appreciate the reviewer for calling our attention to the need for a richer mechanistic discussion of novel biomarkers. To address this problem, we have substantially expanded the biological interpretation in the biomarker findings in the TCGA-BRCA, TCGA-COAD, and new added ADNI data for further biological interpretation. In the revised manuscript, we added detailed interpretation on the biomarkers found by our method and comprehensive explanation of the pathways found by our methods.

Revision

(1) In Results under "Comprehensive Analysis of Individual Cancer Data: Breast Cancer and Colorectal Cancer", added interpretation in subsection "Interpretation of Selected Biomarkers"

Interpretation of Selected Biomarkers Figure 3a top panel highlights the top 20 BRCA biomarkers by the IMD-mixture weight across genes (left), CpG probes (center), and miRNA (right). Prominent genes included FOXC1, PRR15, and PRKCQ. FOXC1 drives epithelial–mesenchymal transition and correlates with poor survival in breast cancer; PRR15 has recently been identified as a luminal-subtype marker in hormone receptor–positive tumors1; and PRKCQ suppresses ERα expression and is required for mammary tumorigenesis in triple-negative models2. Other high-ranking genes include MMP11, which is well-documented in the literature as playing a pivotal role in breast cancer, showing high expression levels in early luminal subtypes3,4. Additionally, BCL11A has also been shown to be related to triple-negative breast cancer5. Furthermore, ESR1, the gene that encodes the estrogen receptor (ER) along with pioneering transcription factor FOXA1 is well established factors in hormonally dependent breast cancer6. Among the DNA methylation features, probes such as cg03441279 in BCL9 and cg12427162 in SFT2D2 have been associated with breast cancer prognosis7. On the miRNA side, MIMAT0003249 (hsa-miR-584-5p) and MIMAT0000064 (hsa-let-7c-5p) stood out, both having been previously implicated in breast cancer biology8,9.

For the TCGA-COAD dataset (Figure 3a bottom panel), two of the top genes selected by the model, FRP4 and ANGPTL1, are known to be highly expressed in colorectal cancer (CRC) and have been linked to poor clinical outcomes in CRC patients10–12. Although fewer DNA methylation probes emerged prominently, cg12374721, which maps to the PRAC2 locus, exceeded the 0.5 weight threshold. This site was previously flagged as a novel pan-cancer methylation biomarker, showing consistent differential methylation in colon, rectal, and prostate tissues13,14. In miRNAs, MIMAT0000098 (hsa-miR-100-5p) and MIMAT0000076 (hsa-miR-21-5p) are top ranked lead the ranking; miR-100 is significantly downregulated in colorectal tumors, and lower miR-100 expression correlates with advanced stage and poorer overall survival in CRC patients15. Additionally, study also shown that hsa-miR-21-5p is useful in diagnosis of CRC16.

(2) In Results under "Comprehensive Analysis of Individual Cancer Data: Breast Cancer and Colorectal Cancer", added interpretation in subsection "Functional Analysis and Prognostic Comparison"

Functional Analysis and Prognostic Comparison We then applied the functional enrichment analysis to the selected genes. The functional analysis was performed using clusterProfiler R package focusing on Canonical (C2:CP), GO (C5:BP) and Hallmark pathways from the Molecular Signatures Database17. Figure 3b displays the top 10 significant pathways (i.e. FDR < 0.05). In BRCA, the leading Canonical hits are Constitutive Signaling by Aberrant PI3K in Cancer, PI3K AKT Signaling in Cancer, and multiple ERBB4-driven routes, reflecting the centrality of PI3K/AKT and ERBB networks in hormone-receptor-positive breast tumors18. Complementary GO Biological Processes such as Gland Development, Mammary Gland Epithelium Development,

Powered by Editorial Manager® and ProduXion Manager® from Aries Systems Corporation

and Positive Regulation of Cell–Cell Junction Assembly are crucial in understanding tumorigenesis, particularly in cancers like breast cancer19. In COAD, enriched Canonical pathways are dominated by extracellular matrix remodeling (ECM Proteoglycans, Extracellular Matrix Organization), while GO terms center on muscle and connective-tissue processes (Muscle Contraction, Cartilage Development, Collagen Fibril Organization). Notably, the Hallmark sets Epithelial–Mesenchymal Transition and Myogenesis also score among the top hits, underlining the dual roles of stromal reprogramming and EMT in colorectal cancer progression20,21.

2. Sensitivity Analysis of Randomness: The authors should conduct and discuss sensitivity analyses regarding different random states or random seeds to assess the stability of the method's results.
We thank the reviewer for raising the important issue of random-seed sensitivity. To quantify the robustness of our MRF-IMD selection, we repeated the full pipeline 30 times per cohort (BRCA/COAD) and summarized (i) Model Size and (ii) the Szymkiewicz–Simpson Overlap Coefficient, which is defined as the size of the intersection divided by the smaller size of the two sets22, across runs. Distributions were tight with high pairwise overlap across all three IMD strategies.
As summarized in Figure 1 (Supplementary Figure 3), across BRCA and COAD, Filter yielded the most compact yet stable signatures; Mixture provided a balanced middle ground; Transformation returned the broadest panels with still high reproducibility. These patterns were consistent across genes, CpGs, and miRNAs. These results demonstrate that our variable-selection results are highly stable: changing the random seed has only a minimal effect on which features are chosen. We have added a detailed description of this analysis in the “Stability and Sensitivity Analysis” subsection under “Comprehensive Analysis of Individual Cancer Data: Breast Cancer and Colorectal Cancer” in the Results.
Figure 1

Revision
In Results under “Comprehensive Analysis of Individual Cancer Data: Breast Cancer and Colorectal Cancer”, added subsection “Stability and Sensitivity Analysis”
Stability and Sensitivity Analysis To assess the stability of the identified variables, we assessed the stability of the identified variables of our MRF-IMD method at different seed settings. We repeated the MRF model analysis 30 times using different seeds for each combination and applied summarized (i) the number of variables selected and (ii) the pairwise overlap coefficient (also known as the Szymkiewicz–Simpson coefficient- intersection divided by the smaller set size) for each omics block. Across BRCA and COAD, Filter yielded the most compact yet stable signatures; Mixture provided a balanced middle ground; Transformation returned the broadest panels with still high reproducibility. These patterns were consistent across genes, CpGs, and miRNAs. Supplementary Figure 3 summarizes the stability of gene, CpG and miRNA selections across 30 MRF-IMD runs for both TCGA-BRCA (top panels) and TCGA-COAD (bottom panels). In BRCA, the filter strategy consistently yields the most compact signatures (median ≈73 genes, 91 CpGs, 33 miRNAs) and attains high reproducibility (overlap coefficients ≈70.80 for genes, 0.90 for CpGs, 0.95 for miRNAs). The mixture approach selects intermediate-sized sets (median ≈100 genes, 140 CpGs, 27 miRNAs) with slightly lower median overlaps (0.78/0.85/0.98), while transformation produces the largest signatures (≈235 genes, 290 CpGs, 45 miRNAs) but still maintains strong stability (0.75/0.80/0.90). In COAD, all three methods expand their gene and CpG panels relative to BRCA, yet the same ranking of stability holds. Filter picks ~120 genes, 75 CpGs, 43 miRNAs (overlaps≈0.68/0.78/0.90); mixture yields median selection of 145/125/15 features (overlaps ≈70.70/0.75/1.00); and transformation selects median selection of 120/130/60 features with moderate reproducibility (0.62/0.65/0.85). These results demonstrate that, regardless of cohort or omics layer, IMD-transformation offers the best trade-off between breadth and consistency, IMD-filter delivers the most parsimonious yet stable core signature, and IMD-mixture provides a middle ground in both model size and overlap.
3. Comparison with Existing Methods on Real Data: While the simulation studies provide thorough benchmarking, the manuscript could enhance its practical value by including detailed comparisons with methods such as SPLS, PMDCCA, and SGCCA using the real-world TCGA datasets.
We thank the reviewer for recommending direct benchmarks against SPLS, PMDCCA, and SGCCA. In response, we have extended our analyses in two key areas. First, for

the TCGA-BRCA and TCGA-COAD cohorts, we reran SPLS, PMDCCA, and SGCCA—tuning each to select the same number of features as MRF-IMD—and then applied identical pathway enrichment and survival-stratification workflows. The updated results (Figures 3b-c, Supplementary Table 1) demonstrate that MRF-IMD uncovers a broader, more biologically coherent set of pathways and achieves stronger separation of patient survival curves compared to these linear integrators. Second, in our pan-cancer clustering study, we added PAM clustering on SPLS-derived components as an additional comparator. The revised adjusted Rand index comparisons in Supplementary Figure 4 now include SPLS, further illustrating MRF-IMD's superior ability to recover true tumor-type labels across diverse cancer lineages.

Revision

(1) In Results under "Comprehensive Analysis of Individual Cancer Data: Breast Cancer and Colorectal Cancer", added subsection "Signature Selection for Comparative Evaluation"

Signature Selection for Comparative Evaluation For the rest of the analysis, we adopted the IMD-mixture variable selection strategy as it delivers a balanced signature size (neither too sparse nor overly broad) while maintaining high selection stability across seeds. For reproducible research, we selected the seed for the model fitting that was closest to the median across 30 seeds. To enable a fair head-to-head evaluation, we then configured SPLS, PMDCCA, and RGCCA to yield the similar total number of features. For SPLS (mixOmics), we specified the keepX vector in block.spls function to match the per-component counts from IMD-mixture. PMDCCA (PMA) was run with its default CCA.permute routine, which, if no penalty is supplied, automatically selects optimal penalty terms via permutation testing. For RGCCA (RGCCA), we manually set the shrinkage penalties to 0.1 to ensure the selection sparsity. All integrative models were run with five components for the downstream prognostic analyses, yielding comparable feature-set sizes across methods.

(2) In Results under "Comprehensive Analysis of Individual Cancer Data: Breast Cancer and Colorectal Cancer", added comparison in "Functional Analysis and Prognostic Comparison"

Figure 3c shows the four-way Venn diagrams of the significant pathways selected by MRF-IMD, SPLS, PMDCCA, and RGCCA in BRCA and COAD. In BRCA (left), MRF-IMD identified 55 unique pathways (25.3%), while SPLS, PMDCCA, and RGCCA only identified 27 (12.4%), 35 (16.1%), and 37 (17.1%) unique pathways, respectively. Only 37 pathways (17.1%) overlap between MRF-IMD and SPLS, and 40 (20.3%) between MRF-IMD and RGCCA; there are no shared pathways in PMDCCA or common to all four methods. The core oncogenic pathways such as Constitutive Signaling by Aberrant PI3K in Cancer and PI3K AKT Signaling in Cancer are uniquely retrieved by MRF-IMD that the linear integrators might miss. In COAD, MRF-IMD again dominates with 48 unique enrichments (64.0%), whereas SPLS yields none, PMDCCA yields 2 (2.7%), and RGCCA yields 12 (16.0%). Only 4 pathways (5.3%) overlap between MRF-IMD and RGCCA, and 2 (2.6%) between MRF-IMD and PMDCCA; there are no shared pathways in SPLS or common to all four methods. Among its unique COAD hits, MRF-IMD highlights Collagen Fibril Organization and Ossification, underscoring its ability to capture tissue-specific remodeling programs that go beyond the canonical and component-based integrators.

An important goal in cancer multi-omics studies is to identify biomarkers that not only reflect biological mechanisms but also correlate with clinical outcomes. To evaluate the prognostic value of the variables identified by our MRF-based methods, we applied integrative non-negative matrix factorization (IntNMF) method23 to combine the selected variables from the three omics data types for both BRCA and COAD. This integration allowed us to cluster patients into two groups representing high- and low-risk survival profiles. Figure 3d shows the resulting Kaplan–Meier curves of the grouping results from clustering using all variables, top 5 SPLS components, and the variables selected MRF-IMD-mixture methods. Table 4 reports the median log-rank p-values across 30 seeds of MRF-IMD methods and log-rank p-values of other benchmark methods. In BRCA, clustering on all variables yields no significant separation (P-value =0.28), while SPLS and the CCA-based methods achieve modest significance only when using all five components (SPLS: P-value $=8.2\times10^{-3}$; PMDCCA: P-value $=1.4\times10^{-2}$; RGCCA: P-value $=2.4\times10^{-2}$) but fail when restricted to their selected features. In contrast, MRF-IMD's filter and mixture strategies produce highly significant stratification (median P-value $=7.9\times10^{-4}$ and 1.0×10^{-3} respectively), with the filter set delivering the strongest separation in the Kaplan–Meier curves (P-value $=4\times10^{-4}$). In COAD, neither the full feature set (P-value =0.24) nor

Powered by Editorial Manager® and ProduXion Manager® from Aries Systems Corporation

SPLS selected variables-or RGCCA-derived signatures yield significant stratification. SPLS with all five components and PMDCCA's selected variables attain significance (P-value=1.3×10^(-2) and 3.0×10^(-2)), but once again the MRF-IMD filter and mixture panels outperform, both achieving P-value =1.2-1.4×10^(-2).
Together, these results demonstrate that our MRF-IMD–derived biomarker sets consistently enable more robust risk stratification than existing integrative methods, confirming their potential clinical utility for patient stratification and prognostic modeling.
(3) In Results, under "TCGA PAN Cancer Clustering Analysis", revised the clustering analysis into a subsection "Pan-Cancer Clustering" and added SPLS clustering comparison
Pan-Cancer Clustering We next applied IntNMF directly to the MRF-IMD feature set and determined an optimal rank of eight clusters using nmf.opt.k function from the IntNMF R package. Figure 4d shows the resulting confusion matrix, illustrating how our selected features effectively separated the samples into eight clusters. Each cluster highlighted unique molecular characteristics and captured established patterns of tumor heterogeneity, ranging from a combined basal-like breast and uterine carcinoma cluster (Group 1) through gastrointestinal adenocarcinomas (Group 5) and hepatobiliary tumors (Group 2) to hypermutated immunogenic cancers (Group 3), squamous-cell carcinomas (Group 7), endocrine neoplasms (Group 6), and renal epithelial tumors (Group 8). A detailed description of the these clusters is in Table 5. To further quantify the advantage conferred by MRF-IMD feature selection, we applied IntNMF clustering to the pan-cancer dataset, aiming for 20 clusters. We first excluded cancer types with fewer than five samples (TCGA-CESC and TCGA-CHOL), as very small cohorts can produce unstable clusters driven by outliers or noise. We then quantified cluster recovery by computing the adjusted rand index (ARI) between the 20 IntNMF clusters on MRF-IMD features and the true TCGA tumor-type labels (Supplementary Figure 4a), benchmarking against four alternative strategies: PAM clustering on 30 SPLS components (Supplementary Figure 4b); IntNMF on the full feature set (Supplementary Figure 4c); PAM clustering on RNA-seq data alone (Supplementary Figure 4d); and PAM clustering on ATAC-seq data alone (Supplementary Figure 4e). Although all approaches are shown moderate to high performance, our method still shows advantage with slightly higher ARI of 0.728. Nearly every TCGA cohort is assigned to a single dominant cluster: COAD (36 out of 37 total samples in group 16), KIRP (31 out of 32 in group 7), PRAD (25 out of 26 in group 15), and UCEC (12 out of 12 in group 11) all show almost perfect one-to-one mapping. In breast cancer, the algorithm distinguished three biologically meaningful subgroups: Group 13 captured a pure basal-like subtype (all 13 basal tumors); Group 5 combined all HER2 (9 out of 10) and LumB (16 out of 16) cases with most LumA (18 out of 29) samples, reflecting a non-basal, high-risk profile; and Group 1 comprised predominantly LumA tumors (9 out of 11) alongside normal controls, defining a lower-risk, luminal-A–driven cluster. Smaller lineages such as BLCA, ESCA, and STAD likewise concentrate into their own clusters with minimal leakage. By contrast, the other methods produced more fragmented assignments and lower ARI scores, sPLS+PAM (ARI = 0.697), full-feature IntNMF (0.687), RNA-seq only (0.713), and ATAC-seq only (0.675), underscoring MRF-IMD's superior ability to isolate coherent, biologically relevant tumor groups.
4. Applicability to Other Diseases: The authors primarily focus on cancer datasets. It is recommended to discuss potential applicability to other disease contexts, such as neurodegenerative or immunological diseases, to illustrate broader utility.
We thank the reviewer for this insightful suggestion. To demonstrate the broader utility of our MRF-IMD framework beyond oncology, we have now added an ADNI analysis in the Results ("Integrative Analysis Enhances Prediction of Dementia Progression in the ADNI Cohort"), in which MRF-IMD on paired blood transcriptome and methylome data robustly prioritizes known Alzheimer's hubs (e.g. CCR7, EPHX2) and outperforms both a single-omics methylation risk score and unfiltered integrative clustering for dementia-conversion stratification.
Revision
(1) In Material and Methods, added "ADNI Data" under section "Real Data Preprocessing"
ADNI Data We analyzed data from the Alzheimer's Disease Neuroimaging Initiative (ADNI), including DNA methylation profiles for 538 cognitive normal (CN) and mild cognitive impaired (MCI) patients measured on Illumina HumanMethylation EPIC v1 arrays preprocessed by Zhang et al.24 and matched gene-expression data from Affymetrix Human Genome U219 microarrays. In this study, we mainly focus on

dementia onsite. Given the heterogeneity and complex progression of Alzheimer's disease (AD), we first selected the top 2,000 CpG methylation sites most significantly associated with AD dementia onsite, using p-value–based screening in the Framingham Heart Study (FHS) dataset as described by Zhang et al.24 For expression, we removed probes lacking gene symbols, flagged and dropped any probe with gene expression below the 10th percentile in over 80% of samples, collapsed remaining probes by gene via median values, and finally selected the 2,000 most variable genes. Dementia conversion was defined as the conversion from CN to MCI or dementia, and from MCI to dementia. After intersecting on subject IDs, we obtained 468 common samples for all integrative analyses. DNA methylation, gene expression data and the dementia status of the subjects were obtained from the ADNI study website (adni.loni.usc.edu). Table 2 summarizes the datasets used in the analysis.
(2) In Results added section "Integrative Analysis Enhances Prediction of Dementia Progression in the ADNI Cohort"
Integrative Analysis Enhances Prediction of Dementia Progression in the ADNI Cohort
MRF-IMD Selected Genes To further illustrate the superior results of our MRF-IMD method, we applied the variable selection to the ADNI data using the filtering strategy. A total of 161 genes and 54 CpG sites were selected by the strategy. Figure 5a shows the top twenty gene-expression and DNA methylation features prioritized by our MRF-IMD framework in the ADNI cohort. On the left panel, ARL11 has the largest weights. While its direct role in AD is still under investigation, ARL11 is known to be involved in apoptosis and immune system processes, which are critical components of neuroinflammation in AD25. Followed by ARL11, S1PR1 plays a significant role in the neuroinflammatory processes of AD26. DAPK2 indicates the involvement of death-associated kinase-mediated neuronal apoptosis and tau dysregulation27, although the precise nature of its contribution to AD pathology requires further investigation. CCR7 reflects its established involvement in chemokine-mediated microglial trafficking and neuroinflammation, with studies indicating that reduced CCR7 expression on meningeal T cells in aging is linked to worsened glymphatic function, cognition, neuroinflammation, and β-amyloid pathology28. Functional analysis of the selected genes identified 36 pathways with FDR < 0.05 (Supplementary Table 2). The enrichment profile was dominated by Lymphocyte (B cells, T cells, NK cells) programs, especially in T cells. Key terms included T Cell Differentiation and Activation, Lymphocyte Differentiation and Activation, supporting a peripheral inflammatory state relevant to Alzheimer's disease29,30.
Pathway Analysis of Selected DNAm CpGs To assess the biological relevance of our MRF-IMD selected CpGs versus those significant CpGs reported by Zhang et al., we performed KEGG and GO enrichment with the missMethyl R package on both the 54 MRF-IMD prioritized sites (top 20 out of 54 sites showed in Figure 5a right panel) and the 44 meta-analysis significant sites. Supplementary Table 3 displays the top 15 most significant pathways for each method. In the KEGG analysis, MRF-IMD CpGs showed strongest enrichment in NF-κB Signaling, C-type lectin Receptor Signaling, and Leukocyte Transendothelial Migration. Together these pathways indicate coordinated innate immune activation and immune-cell trafficking across the endothelium, processes that escalate neuroinflammation and contribute to AD progression. These results are concordant with the pathway enrichments obtained from the selected gene set. By contrast, the Zhang et al. meta-analysis CpGs were enriched for viral-infection and adhesion processes, top hits were Virion–Ebolavirus, Lyssavirus and Morbillivirus, Cell Adhesion Molecules and mTOR Signaling, indicating a shift toward pathogen-related and cell–matrix interaction pathways.
In the GO analysis, MRF-IMD CpGs emphasized post-transcriptional RNA processing (Polyribonucleotide Nucleotidyltransferase Activity; Nuclear and Mitochondrial Polyadenylation-Dependent mRNA Catabolic Processes; Poly(U) RNA Binding), along with cytokine regulation via Interleukin-1 Type I/II Receptor Antagonist Activity and neuromodulatory pathways including Wnt Signaling and Galanin Receptor Binding (Types 1–3). Notably, these CpG-derived enrichments align with the gene-based pathways through shared immune modules such as Interleukin-1 Receptor Antagonist Activity and Wnt Signaling, while the RNA processing and galanin receptor terms appear CpG-specific. In contrast, the meta-analysis CpGs were dominated by cell-division and cytokinesis terms (Protein Localization to Division Site; Cleavage Furrow; Mitotic Cytokinetic Regulation). These comparisons underscore that our MRF-IMD approach yields CpGs tied to innate immune signaling, mRNA processing and metabolic regulation that were more detailed and emphasized core processes directly associated in AD.

Powered by Editorial Manager® and ProduXion Manager® from Aries Systems Corporation

Integrative Validation on Dementia Progression To demonstrate that our integrative variable-selection outperforms both the single-layer methylation risk score (MRS) from Zhang et al. and an integrative approach without feature selection, we compared three stratifications of dementia conversion. We applied intNMF to the MRF-IMD selected features, obtaining two clusters, and evaluated time to dementia conversion using Kaplan–Meier curves and a log-rank test. The MRF-IMD panel showed a significant separation (Figure 5b; P-value = 0.033). We benchmarked three alternatives (Figure 5c): (left) intNMF on the full, unfiltered full omics set (P-value = 0.048); (middle) the published MRS based on 151 CpGs, dichotomized at the data-driven cut-point using MaxStat (P-value = 0.048); and (right) intNMF on the first five SPLS components from the full omics set (P-value = 0.60). Across comparisons, the MRF-IMD hubs delivered the strongest prognostic discrimination, outperforming methylation-only scoring and unfiltered or dimension-reduced integration.
In the original Zhang et al. study, the MRS was built via ridge regression on CpGs significantly associated with conversion and tested in a multivariate Cox model adjusted for age, sex, APOE 4 status, years of education, baseline diagnosis, and baseline MMSE score (Surv(conversion event, follow-up) ~ MRS + covariates). When we substituted our IntNMF-derived component from the MRF-IMD features into this identical Cox framework with the MRS, we observed a stronger association with progression to the next disease stage (Table 6). This result confirms that our integrative variable-selection not only refines molecular subtyping but also enhances prediction of disease progression beyond both single-omics risk scores and non-prioritized integrative analyses.
Integrative Validation on Dementia Progression To demonstrate that our integrative variable-selection outperforms both the single-layer methylation risk score (MRS) from Zhang et al. and an integrative approach without feature selection, we compared three stratifications of dementia conversion (as defined in Zhang et al.). First, we dichotomized the published MRS using 151 CpGs at cut off determined by maximum rank statistic, which implemented in the MaxStat R package, and evaluated its Kaplan–Meier survival curves with a log-rank test. Second, we applied intNMF clustering to the full set of omics variables and assessed the resulting subgroups in the same way. Third, we repeated the intNMF procedure using only our MRF-IMD-prioritized features. As shown in Figure 5b, the MRF-IMD panel achieved the most significant separation (lowest P-value), indicating that focusing on cross-omics hub variables yields stronger prognostic discrimination than using methylation alone or an unfiltered integrative feature set.
In the original Zhang et al. study, the MRS was built via ridge regression on CpGs significantly associated with conversion and tested in a multivariate Cox model adjusted for age, sex, APOE ε4 status, years of education, baseline diagnosis, and baseline MMSE score (Surv(conversion event, follow-up) ~ MRS + covariates). When we substituted our intNMF-derived component from the MRF-IMD features into this identical Cox framework, we observed a stronger association with progression to the next disease stage (Table 7). This result confirms that our integrative variable-selection not only refines molecular subtyping but also enhances prediction of disease progression beyond both single-omics risk scores and non-prioritized integrative analyses.
5. Improved Visualization: Some figures in the manuscript have font sizes that are too small, which might impair readability. It is recommended to enlarge the text labels, legends, and axis annotations to ensure that all information is clearly visible and accessible. In Figure 8, the use of sub-labels (such as a, b, c) is mentioned in the text, but these labels are not visible in the figure itself.
We thank the reviewer for pointing out these important visualization issues. In response, we have revised all figures throughout the manuscript to improve overall clarity and readability.
Reviewer #2
The article presents an Integrative Multi-Omics Random Forest Framework for Robust Biomarker Discovery. It addresses the challenge of extracting key shared biomarkers from multiple omics data types by introducing a multivariate random forest-based approach enhanced by an inverse minimal depth metric.
We are grateful for your thoughtful evaluation and constructive feedback. All revisions are highlighted in red in the updated manuscript to facilitate your review. We believe these changes comprehensively address your concerns and significantly enhance the clarity, rigor, and overall presentation of our work. Thank you again for your valuable suggestions.

Powered by Editorial Manager® and ProduXion Manager® from Aries Systems Corporation

I have some concerns and comments below:
1. The new algorithm described in the study selected omics variables by assigning response variable to decision tree nodes. How the response variables relate to biological responses/outcomes? From the authors' description, it seems that the selected omics variables using the IMD are almighty, i.e., they can predict anything needed, such as prognosis, cancer types, and et al. Actually, the usual logic to select omics variables to predict prognosis is to evaluate the association between omics variables and survival time.

We appreciate the reviewer for this important question. We would like to clarify that our integrative variable selection method is an unsupervised procedure which no clinical labels ever enter the model in the training process. The “response” variable that the forest models is simply a second omics layer (e.g. miRNA when mRNA is the predictor set, or vice versa), not a clinical endpoint like survival time or tumor subtype. The inverse minimal depth (IMD) metric quantifies how early each variable appears in the splitting hierarchy of the forest, reflecting its importance in mediating cross-omics variation. Higher IMD values indicate that the selected variables are more likely to share information across layers, acting as hubs in the multi-omics network. These hubs coordinate core biological pathways, such as cell cycle control, DNA repair, and immune signaling, that both define molecular subtypes and drive differences in patient outcomes.

When these IMD-selected hubs are carried forward into downstream analyses, whether consensus clustering for subtyping or prognosis test, they yield more stable, biologically coherent clusters and higher prognostic accuracy than feature panels chosen by classical univariate survival filtering or single-omics integration methods. We agree with the reviewer that outcome-guided preselection can further strengthen variable choice, especially for a complex disorder such as Alzheimer’s disease (AD). Accordingly, we added a new ADNI analysis of AD dementia onset in which methylation CpGs were preselected from the epigenome-wide association study by Zhang et al.24 Incorporating this EWAS-informed prior into our model produced improved clustering stability and prognostic accuracy while preserving interpretability. We have revised the Methods and Results sections and added the corresponding figure and table to document these updates. We have revised our manuscript as follows:

Revision

(1) In Introduction, added a detailed explanation and references

Recent technological advances in high-throughput sequencing, mass spectrometry, and imaging have led to a surge in multi-omics data that span the genome, epigenome, transcriptome, proteome, and metabolome. However, each type of data alone captures only a slice of disease biology. Integrating these diverse data sources can provide a more comprehensive picture of complex biological systems than analyzing any single omics layer alone. Multi-omics analysis has been implemented in many studies for biomarker discovery, disease subtyping, and disease insights . A key goal in multi-omics integration is to extract “shared” biomarkers from multiple data – that is, to identify molecular features that are consistently important across different omics platforms. These biomarkers typically indicate robust, system-level regulatory mechanisms that single-omics analyses may miss31,32. Furthermore, integrating complementary data sources reduces noise and mitigates biological heterogeneity, enhancing the precision and clinical relevance of patient stratification and prognosis33. In general, multi-omics approaches tend to yield more reliable biomarkers and disease signatures than single-modality analyses, as demonstrated in recent studies: methods like DIABLO, which based on the sparse partial least squares (SPLS) method, seek common information across data types by selecting subsets of features that jointly capture variance in each dataset34. When done effectively, integration can highlight shared molecular features across different data types, offering new insights into disease mechanisms, patient stratification, and potential biomarkers for clinical applications34–37.

(2) In Introduction, added clarification on method description

In this study, we introduce a new MRF-based framework that employs the inverse minimal depth (IMD) metric for unsupervised variable selection across multiple omics datasets. We model the relationships between two omics by assigning one omics to the response space in and the other omics to the feature space in an ensemble of decision trees. After fitting the forest, we compute the IMD to quantify feature importance and identify key variables shared across different data layers. We then extend our framework from pairwise (two-omics) integration to a comprehensive multi-

Powered by Editorial Manager® and ProduXion Manager® from Aries Systems Corporation

	omics approach by modeling different layer pairs guided by prior knowledge or precomputed inter-relationships. This strategy naturally reduces the risk of selecting noise variables and helps focus on those with consistent impact across datasets.<br>(3) In Material and Methods, added “ADNI Data” under section “Real Data Preprocessing”<br>ADNI Data We analyzed data from the Alzheimer’s Disease Neuroimaging Initiative (ADNI), including DNA methylation profiles for 538 cognitive normal (CN) and mild cognitive impaired (MCI) patients measured on Illumina HumanMethylation EPIC v1 arrays preprocessed by Zhang et al.24 and matched gene-expression data from Affymetrix Human Genome U219 microarrays. In this study, we mainly focus on dementia onsite. Given the heterogeneity and complex progression of Alzheimer’s disease (AD), we first selected the top 2,000 CpG methylation sites most significantly associated with AD dementia onsite, using p-value–based screening in the Framingham Heart Study (FHS) dataset as described by Zhang et al.24 For expression, we removed probes lacking gene symbols, flagged and dropped any probe with gene expression below the 10th percentile in over 80% of samples, collapsed remaining probes by gene via median values, and finally selected the 2,000 most variable genes. Dementia conversion was defined as the conversion from CN to MCI or dementia, and from MCI to dementia. After intersecting on subject IDs, we obtained 468 common samples for all integrative analyses. DNA methylation, gene expression data and the dementia status of the subjects were obtained from the ADNI study website (adni.loni.usc.edu). Table 2 summarizes the datasets used in the analysis.<br>(4) In Results added section “Integrative Analysis Enhances Prediction of Dementia Progression in the ADNI Cohort”<br>Integrative Analysis Enhances Prediction of Dementia Progression in the ADNI Cohort MRF-IMD Selected Genes To further illustrate the superior results of our MRF-IMD method, we applied the variable selection to the ADNI data using the filtering strategy. A total of 161 genes and 54 CpG sites were selected by the strategy. Figure 5a shows the top twenty gene-expression and DNA methylation features prioritized by our MRF-IMD framework in the ADNI cohort. On the left panel, ARL11 has the largest weights. While its direct role in AD is still under investigation, ARL11 is known to be involved in apoptosis and immune system processes, which are critical components of neuroinflammation in AD25. Followed by ARL11, S1PR1 plays a significant role in the neuroinflammatory processes of AD26. DAPK2 indicates the involvement of death-associated kinase-mediated neuronal apoptosis and tau dysregulation27, although the precise nature of its contribution to AD pathology requires further investigation. CCR7 reflects its established involvement in chemokine-mediated microglial trafficking and neuroinflammation, with studies indicating that reduced CCR7 expression on meningeal T cells in aging is linked to worsened glymphatic function, cognition, neuroinflammation, and β-amyloid pathology28. Functional analysis of the selected genes identified 36 pathways with FDR < 0.05 (Supplementary Table 2). The enrichment profile was dominated by Lymphocyte (B cells, T cells, NK cells) programs, especially in T cells. Key terms included T Cell Differentiation and Activation, Lymphocyte Differentiation and Activation, supporting a peripheral inflammatory state relevant to Alzheimer’s disease29,30.<br>Pathway Analysis of Selected DNAm CpGs To assess the biological relevance of our MRF-IMD selected CpGs versus those significant CpGs reported by Zhang et al., we performed KEGG and GO enrichment with the missMethyl R package on both the 54 MRF-IMD prioritized sites (top 20 out of 54 sites showed in Figure 5a right panel) and the 44 meta-analysis significant sites. Supplementary Table 3 displays the top 15 most significant pathways for each method. In the KEGG analysis, MRF-IMD CpGs showed strongest enrichment in NF-κB Signaling, C-type lectin Receptor Signaling, and Leukocyte Transendothelial Migration. Together these pathways indicate coordinated innate immune activation and immune-cell trafficking across the endothelium, processes that escalate neuroinflammation and contribute to AD progression. These results are concordant with the pathway enrichments obtained from the selected gene set. By contrast, the Zhang et al. meta-analysis CpGs were enriched for viral-infection and adhesion processes, top hits were Virion–Ebolavirus, Lyssavirus and Morbillivirus, Cell Adhesion Molecules and mTOR Signaling, indicating a shift toward pathogen-related and cell–matrix interaction pathways.<br>In the GO analysis, MRF-IMD CpGs emphasized post-transcriptional RNA processing (Polyribonucleotide Nucleotidyltransferase Activity; Nuclear and Mitochondrial Polyadenylation-Dependent mRNA Catabolic Processes; Poly(U) RNA Binding), along with cytokine regulation via Interleukin-1 Type I/II Receptor Antagonist Activity and

neuromodulatory pathways including Wnt Signaling and Galanin Receptor Binding (Types 1–3). Notably, these CpG-derived enrichments align with the gene-based pathways through shared immune modules such as Interleukin-1 Receptor Antagonist Activity and Wnt Signaling, while the RNA processing and galanin receptor terms appear CpG-specific. In contrast, the meta-analysis CpGs were dominated by cell-division and cytokinesis terms (Protein Localization to Division Site; Cleavage Furrow; Mitotic Cytokinetic Regulation). These comparisons underscore that our MRF-IMD approach yields CpGs tied to innate immune signaling, mRNA processing and metabolic regulation that were more detailed and emphasized core processes directly associated in AD.

Integrative Validation on Dementia Progression To demonstrate that our integrative variable-selection outperforms both the single-layer methylation risk score (MRS) from Zhang et al. and an integrative approach without feature selection, we compared three stratifications of dementia conversion. We applied intNMF to the MRF-IMD selected features, obtaining two clusters, and evaluated time to dementia conversion using Kaplan–Meier curves and a log-rank test. The MRF-IMD panel showed a significant separation (Figure 5b; P-value = 0.033). We benchmarked three alternatives (Figure 5c): (left) intNMF on the full, unfiltered full omics set (P-value = 0.048); (middle) the published MRS based on 151 CpGs, dichotomized at the data-driven cut-point using MaxStat (P-value = 0.048); and (right) intNMF on the first five SPLS components from the full omics set (P-value = 0.60). Across comparisons, the MRF-IMD hubs delivered the strongest prognostic discrimination, outperforming methylation-only scoring and unfiltered or dimension-reduced integration.

In the original Zhang et al. study, the MRS was built via ridge regression on CpGs significantly associated with conversion and tested in a multivariate Cox model adjusted for age, sex, APOE 4 status, years of education, baseline diagnosis, and baseline MMSE score (Surv(conversion event, follow-up) ~ MRS + covariates). When we substituted our IntNMF-derived component from the MRF-IMD features into this identical Cox framework with the MRS, we observed a stronger association with progression to the next disease stage (Table 6). This result confirms that our integrative variable-selection not only refines molecular subtyping but also enhances prediction of disease progression beyond both single-omics risk scores and non-prioritized integrative analyses.

Integrative Validation on Dementia Progression To demonstrate that our integrative variable-selection outperforms both the single-layer methylation risk score (MRS) from Zhang et al. and an integrative approach without feature selection, we compared three stratifications of dementia conversion (as defined in Zhang et al.). First, we dichotomized the published MRS using 151 CpGs at cut off determined by maximum rank statistic, which implemented in the MaxStat R package, and evaluated its Kaplan–Meier survival curves with a log-rank test. Second, we applied intNMF clustering to the full set of omics variables and assessed the resulting subgroups in the same way. Third, we repeated the intNMF procedure using only our MRF-IMD-prioritized features. As shown in Figure 5b, the MRF-IMD panel achieved the most significant separation (lowest P-value), indicating that focusing on cross-omics hub variables yields stronger prognostic discrimination than using methylation alone or an unfiltered integrative feature set.

In the original Zhang et al. study, the MRS was built via ridge regression on CpGs significantly associated with conversion and tested in a multivariate Cox model adjusted for age, sex, APOE ε4 status, years of education, baseline diagnosis, and baseline MMSE score (Surv(conversion event, follow-up) ~ MRS + covariates). When we substituted our intNMF-derived component from the MRF-IMD features into this identical Cox framework, we observed a stronger association with progression to the next disease stage (Table 7). This result confirms that our integrative variable-selection not only refines molecular subtyping but also enhances prediction of disease progression beyond both single-omics risk scores and non-prioritized integrative analyses.

2. Following the discussion in 1, what is the biological meaning to extract shared biomarkers from multiple data layers? While it is straightforward to think that the shared biomarkers between multiple data layers or data types may induce the same biological responses, the unique biomarkers also matter depending on what biological responses we care.

Thank you for raising this point. We agree that, alongside cross-omics “shared” signals, layer-specific biomarkers can be biologically informative depending on the phenotype

Powered by Editorial Manager® and ProduXion Manager® from Aries Systems Corporation

under study. Our framework intentionally prioritizes biomarkers that shared similar information, which are features whose variation is concordant across omics, because these tend to capture upstream regulatory programs, are less sensitive to platform-specific noise, and thus yield more stable, clinically transferable signatures33. This focus does not exclude omics-specific biology: IMD will still retain strong within-omics features when they help explain cross-omics variation. In the revision, we clarify this rationale and outline a simple extension (“shared then unique”) that first prioritizes shared hubs with MRF IMD and, when a specific endpoint is of interest (e.g., survival), augments the panel with omics-specific markers tailored to that phenotype.

Looking ahead, we plan to extend the framework to simultaneously extract both shared cross-omics hubs and unique omics-specific signatures, thereby tailoring biomarker discovery to diverse biological and clinical objectives. To address this point, we have revised the Introduction as described in Comment 1, and we have added a new limitation paragraph in the Discussion to highlight these considerations and outline our planned extensions.

Revision

(1) In Introduction, added detailed explanation and references (see Revision (1) in Comment 1)

(2) In Discussion, added limitation

While our method provides clear advantages, some limitations remain. First, computation time may increase with more datasets and extreme high-dimensionality. Future research could focus on improving efficiency, potentially through parallelization or dimensionality reduction strategies that preserve essential biological signals. Second, further integration with downstream validation steps, such as experimental verification or functional assays, would help confirm the biological significance of the selected variables and strengthen the evidence for potential biomarkers. Third, although we prioritize cross-omics shared biomarkers to capture system-level regulators, we recognize that unique, omics-specific features (e.g., methylation marks reflecting environmental exposure or miRNAs mediating post-transcriptional control) also carry important biological information. Future work in developing a promising extension of the framework that extracts both omics-specific and shared biomarkers would enable more comprehensive biological insights.

3. The Introduction section is not sufficient. The biological significance and technical details of "extract shared biomarkers from multiple data layers" need to be explained in more details.

We thank the reviewer for the suggestion on adding the explanation for "extract shared biomarkers from multiple data layers". In response, we have expanded the Introduction (see Comment 1). Additionally, we also revised our Introduction section by adding more details and description of our methods, simulations, and data applications.

Revision

(1) In Introduction, added detailed explanation

A key goal in multi-omics integration is to extract “shared” biomarkers from multiple data layers – that is, to identify molecular features that are biologically relevant consistently across different omics types. These biomarkers typically indicate robust, system-level regulatory mechanisms that single-layer analyses may miss31,32.

(2) In Introduction, added clarification on method description and summary of applications

In this study, we introduce a new MRF-based framework that employs the inverse minimal depth (IMD) metric for unsupervised variable selection across multiple omics datasets. We model the relationships between each pair of two omics by assigning one omics to the response space in and the other omics to the feature space in an ensemble of decision trees. After fitting the forest, we compute the IMD to quantify feature importance. We then extend our framework from pairwise (two-omics) integration to a multi-omics approach by modeling different layer pairs guided by prior knowledge or precomputed inter-relationships. This strategy reduces the risk of selecting noise variables and helps focus on those with consistent impact across datasets. To show that our method can effectively capture shared biomarkers in complex datasets, we benchmarked it against established integration approaches, including SPLS, CCA, and several nonlinear ensemble methods such as gradient boosting machine (GBM) and XGBoost through multiple simulations. We found that methods like SPLS and CCA are not stable in capturing the important features when data types are in nonlinear or contains interaction settings. Moreover, we validated our framework using several clinical cohorts, including TCGA-BRCA and TCGA-COAD, demonstrating superior ability to uncover biologically relevant pathways and to stratify

	patients by prognostic outcome compared to traditional integration methods such as SPLS and CCA. We further applied our approach to the TCGA-PANCAN and ADNI datasets, identifying biomarker panels tied to key biological pathways that show promise for enhancing molecular subtyping of PAN cancer, and prognosis of dementia onset.<br>4. It is advised to provide some examples of the statement in the Introduction: "may fail to capture nonlinear interactions" of the current methods (sPLS, CCA).<br>We appreciate the reviewer's advice on providing some examples of the current methods, such as SPLS or CCA may fail to capture nonlinear interaction. We have provided an example in the Introduction section that illustrates how these methods might fail...
Additional Information:	
Question	Response
Are you submitting this manuscript to a special series or article collection?	No
**Experimental design and statistics**<br><br>Full details of the experimental design and statistical methods used should be given in the Methods section, as detailed in our Minimum Standards Reporting Checklist. Information essential to interpreting the data presented should be made available in the figure legends.<br><br>Have you included all the information requested in your manuscript?	Yes
**Resources**<br><br>A description of all resources used, including antibodies, cell lines, animals and software tools, with enough information to allow them to be uniquely identified, should be included in the Methods section. Authors are strongly encouraged to cite Research Resource Identifiers (RRIDs) for antibodies, model organisms and tools, where possible.<br><br>Have you included the information requested as detailed in our Minimum Standards Reporting Checklist?	Yes
**Availability of data and materials**<br><br>All datasets and code on which the	Yes

conclusions of the paper rely must be either included in your submission or deposited in publicly available repositories (where available and ethically appropriate), referencing such data using a unique identifier in the references and in the “Availability of Data and Materials” section of your manuscript.<br><br>Have you have met the above requirement as detected in our Minimum Standards Reporting Checklist?	
GigaScience has policies and guidelines in place for the use of generative AI-writing tools such as ChatGPT. If you have used such writing tools to assist with writing the manuscript this must be declared and cited in the text. Authors should not list AI-writing tools and other AI-assisted technologies as an author or co-author and should acknowledge that they are fully responsible for text generated or refined by AI-writing tools.<p><br><br>A summary of use (particularly in the introduction or among methods) needs to be included at the end of the paper, and the outputs should also be included as a supplementary file hosted in GigaDB or other open repositories. Please read our guidelines for more information. <p><br><br>By submitting to GigaScience, you are aware of the journal's AI-writing tools policy, and if you have declared use of such tools below, you have acknowledged this where appropriate in your manuscript and have made a summary of use and outputs available. <p><br><b>AI-assisted writing tools have been used in the preparation of this manuscript?	No

Manuscript

Click here to access/download;Manuscript;reply_to_reviewer_082525_clean.d

Reviewer #1

This manuscript presents a novel multivariate random forest (MRF) framework enhanced by the inverse minimal depth (IMD) metric for integrative multi-omics biomarker discovery. The authors clearly demonstrate the robustness and superiority of the proposed methods through comprehensive simulation studies and validation on TCGA datasets. The manuscript provides clear methodological explanations, offering valuable insights into its practical utility. I recommend accepting the manuscript after minor revisions.

We sincerely thank the reviewer for their positive assessment of our framework and their encouraging feedback. In response to the comments, we have made the following updates to enhance the clarity and reproducibility of our manuscript. All changes are marked in red in the revised version for ease of review. We believe these revisions address the reviewer's suggestions and further strengthen the presentation of our work. Thank you once again for your thoughtful comments.

Minor Concern:

1. Biological Interpretation Depth: While the authors identified biologically relevant biomarkers, the biological interpretations remain somewhat superficial. A deeper exploration of novel or less-known biomarkers in the context of disease mechanisms would strengthen the biological relevance of the findings.

We appreciate the reviewer for calling our attention to the need for a richer mechanistic discussion of novel biomarkers. To address this problem, we have substantially expanded the biological interpretation in the biomarker findings in the TCGA-BRCA, TCGA-COAD, and new added ADNI data for further biological interpretation. In the revised manuscript, we added detailed interpretation on the biomarkers found by our method and comprehensive explanation of the pathways found by our methods.

Revision

(1) In Results under "Comprehensive Analysis of Individual Cancer Data: Breast Cancer and Colorectal Cancer", added interpretation in subsection "Interpretation of Selected Biomarkers"

Interpretation of Selected Biomarkers Figure 3a top panel highlights the top 20 BRCA biomarkers by the IMD-mixture weight across genes (left), CpG probes (center), and miRNA (right). Prominent genes included *FOXC1*, *PRR15*, and *PRKCQ*. *FOXC1* drives epithelial–mesenchymal transition and correlates with poor survival in breast cancer; *PRR15* has recently been identified as a luminal-subtype marker in hormone receptor–positive tumors[1]; and *PRKCQ* suppresses ER α expression and is required for mammary tumorigenesis in triple-negative models[2]. Other high-ranking genes include *MMP11,* which is well-documented in the literature as playing a pivotal role in breast cancer, showing high expression levels in early luminal subtypes[3,4]. Additionally, *BCL11A* has also been shown to be related to triple-negative breast

cancer[5]. Furthermore, *ESR1*, the gene that encodes the estrogen receptor (ER) along with pioneering transcription factor *FOXA1* is well established factors in hormonally dependent breast cancer[6]. Among the DNA methylation features, probes such as cg03441279 in *BCL9* and cg12427162 in *SFT2D2* have been associated with breast cancer prognosis[7]. On the miRNA side, MIMAT0003249 (hsa-miR-584-5p) and MIMAT0000064 (hsa-let-7c-5p) stood out, both having been previously implicated in breast cancer biology[8,9].

For the TCGA-COAD dataset (Figure 3a bottom panel), two of the top genes selected by the model, *FRP4* and *ANGPTL1*, are known to be highly expressed in colorectal cancer (CRC) and have been linked to poor clinical outcomes in CRC patients[10–12]. Although fewer DNA methylation probes emerged prominently, cg12374721, which maps to the *PRAC2* locus, exceeded the 0.5 weight threshold. This site was previously flagged as a novel pan-cancer methylation biomarker, showing consistent differential methylation in colon, rectal, and prostate tissues[13,14]. In miRNAs, MIMAT0000098 (hsa-miR-100-5p) and MIMAT0000076 (hsa-miR-21-5p) are top ranked ~~lead the ranking~~; miR-100 is significantly downregulated in colorectal tumors, and lower miR-100 expression correlates with advanced stage and poorer overall survival in CRC patients[15]. Additionally, study also shown that hsa-miR-21-5p is useful in diagnosis of CRC[16].

(2) In Results under "Comprehensive Analysis of Individual Cancer Data: Breast Cancer and Colorectal Cancer", added interpretation in subsection "Functional Analysis and Prognostic Comparison"

Functional Analysis and Prognostic Comparison We then applied the functional enrichment analysis to the selected genes. The functional analysis was performed using clusterProfiler R package focusing on Canonical (C2:CP), GO (C5:BP) and Hallmark pathways from the Molecular Signatures Database[17]. Figure 3b displays the top 10 significant pathways (i.e. FDR < 0.05). In BRCA, the leading Canonical hits are *Constitutive Signaling by Aberrant PI3K in Cancer*, *PI3K AKT Signaling in Cancer*, and multiple ERBB4-driven routes, reflecting the centrality of PI3K/AKT and ERBB networks in hormone-receptor-positive breast tumors[18]. Complementary GO Biological Processes such as *Gland Development*, *Mammary Gland Epithelium Development*, and *Positive Regulation of Cell–Cell Junction Assembly* are crucial in understanding tumorigenesis, particularly in cancers like breast cancer[19]. In COAD, enriched Canonical pathways are dominated by extracellular matrix remodeling (*ECM Proteoglycans*, *Extracellular Matrix Organization*), while GO terms center on muscle and connective-tissue processes (*Muscle Contraction*, *Cartilage Development*, *Collagen Fibril Organization*). Notably, the Hallmark sets *Epithelial–Mesenchymal Transition* and *Myogenesis* also score among the top hits, underlining the dual roles of stromal reprogramming and EMT in colorectal cancer progression[20,21].

2. Sensitivity Analysis of Randomness: The authors should conduct and discuss sensitivity analyses regarding different random states or random seeds to assess the stability of the method's results.

We thank the reviewer for raising the important issue of random-seed sensitivity. To quantify the robustness of our MRF-IMD selection, we repeated the full pipeline 30 times per cohort (BRCA/COAD) and summarized (i) Model Size and (ii) the Szymkiewicz–Simpson Overlap Coefficient, which is defined as the size of the intersection divided by the smaller size of the two sets[22], across runs. Distributions were tight with high pairwise overlap across all three IMD strategies.

As summarized in Figure 1 (Supplementary Figure 3), across BRCA and COAD, **Filter** yielded the most compact yet stable signatures; **Mixture** provided a balanced middle ground; **Transformation** returned the broadest panels with still high reproducibility. These patterns were consistent across genes, CpGs, and miRNAs. These results demonstrate that our variable-selection results are highly stable: changing the random seed has only a minimal effect on which features are chosen. We have added a detailed description of this analysis in the “Stability and Sensitivity Analysis” subsection under “Comprehensive Analysis of Individual Cancer Data: Breast Cancer and Colorectal Cancer” in the Results.

Figure 1

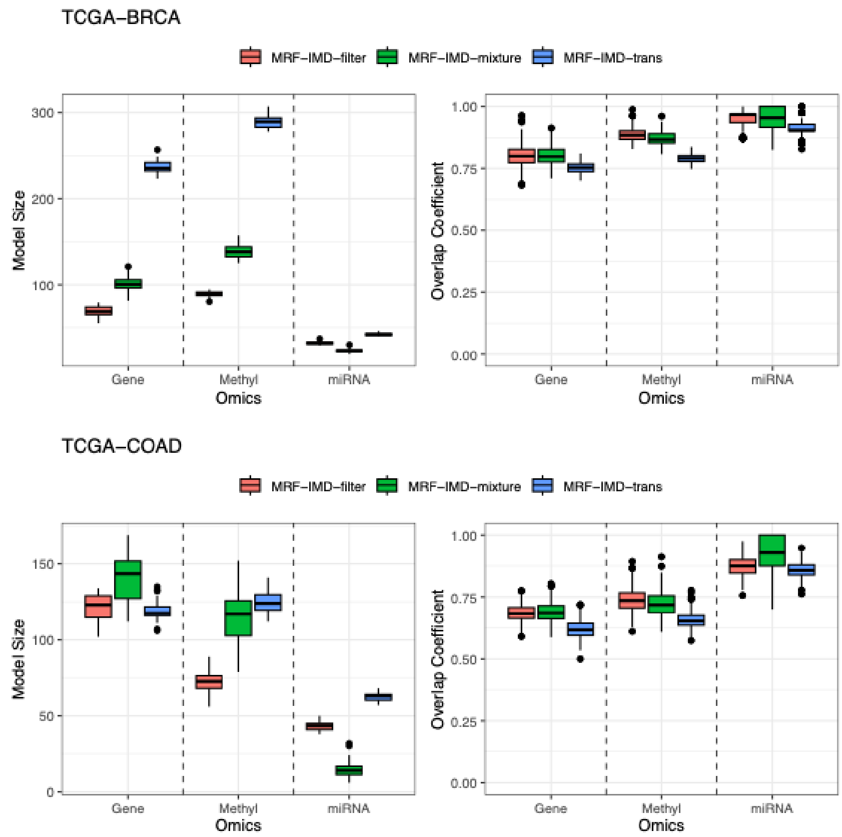

Revision

In Results under "Comprehensive Analysis of Individual Cancer Data: Breast Cancer and Colorectal Cancer", added subsection "Stability and Sensitivity Analysis"

Stability and Sensitivity Analysis To assess the stability of the identified variables, we assessed the stability of the identified variables of our MRF-IMD method at different seed settings. We repeated the MRF model analysis 30 times using different seeds for each combination and applied summarized (i) the number of variables selected and (ii) the pairwise overlap coefficient (also known as the Szymkiewicz–Simpson coefficient-intersection divided by the smaller set size) for each omics block. Across BRCA and COAD, Filter yielded the most compact yet stable signatures; Mixture provided a balanced middle ground; Transformation returned the broadest panels with still high reproducibility. These patterns were consistent across genes, CpGs, and miRNAs. Supplementary Figure 3 summarizes the stability of gene, CpG and miRNA selections across 30 MRF-IMD runs for both TCGA-BRCA (top panels) and TCGA-COAD (bottom panels). In BRCA, the filter strategy consistently yields the most compact signatures (median ≈73 genes, 91 CpGs, 33 miRNAs) and attains high reproducibility (overlap coefficients ≈70.80 for genes, 0.90 for CpGs, 0.95 for miRNAs). The mixture approach selects intermediate-sized sets (median ≈100 genes, 140 CpGs, 27 miRNAs) with slightly lower median overlaps (0.78/0.85/0.98), while transformation produces the largest

signatures (≈235 genes, 290 CpGs, 45 miRNAs) but still maintains strong stability (0.75/0.80/0.90). In COAD, all three methods expand their gene and CpG panels relative to BRCA, yet the same ranking of stability holds. Filter picks ~120 genes, 75 CpGs, 43 miRNAs (overlaps≈0.68/0.78/0.90); mixture yields median selection of 145/125/15 features (overlaps ≈70.70/0.75/1.00); and transformation selects median selection of 120/130/60 features with moderate reproducibility (0.62/0.65/0.85). These results demonstrate that, regardless of cohort or omics layer, IMD-transformation offers the best trade-off between breadth and consistency, IMD-filter delivers the most parsimonious yet stable core signature, and IMD-mixture provides a middle ground in both model size and overlap.

3. Comparison with Existing Methods on Real Data: While the simulation studies provide thorough benchmarking, the manuscript could enhance its practical value by including detailed comparisons with methods such as SPLS, PMDCCA, and SGCCA using the real-world TCGA datasets.

We thank the reviewer for recommending direct benchmarks against SPLS, PMDCCA, and SGCCA. In response, we have extended our analyses in two key areas. First, for the TCGA-BRCA and TCGA-COAD cohorts, we reran SPLS, PMDCCA, and SGCCA—tuning each to select the same number of features as MRF-IMD—and then applied identical pathway enrichment and survival-stratification workflows. The updated results (Figures 3b-c, Supplementary Table 1) demonstrate that MRF-IMD uncovers a broader, more biologically coherent set of pathways and achieves stronger separation of patient survival curves compared to these linear integrators. Second, in our pan-cancer clustering study, we added PAM clustering on SPLS-derived components as an additional comparator. The revised adjusted Rand index comparisons in Supplementary Figure 4 now include SPLS, further illustrating MRF-IMD's superior ability to recover true tumor-type labels across diverse cancer lineages.

Revision

(1) In Results under "Comprehensive Analysis of Individual Cancer Data: Breast Cancer and Colorectal Cancer", added subsection "Signature Selection for Comparative Evaluation"

Signature Selection for Comparative Evaluation For the rest of the analysis, we adopted the IMD-mixture variable selection strategy as it delivers a balanced signature size (neither too sparse nor overly broad) while maintaining high selection stability across seeds. For reproducible research, we selected the seed for the model fitting that was closest to the median across 30 seeds. To enable a fair head-to-head evaluation, we then configured SPLS, PMDCCA, and RGCCA to yield the similar total number of features. For SPLS (mixOmics), we specified the `keepX` vector in `block.spls` function to match the per-component counts from IMD-mixture. PMDCCA (PMA) was run with its default `CCA.permute` routine, which, if no penalty is supplied, automatically selects optimal penalty terms via permutation testing. For RGCCA (RGCCA), we manually set the shrinkage penalties to 0.1 to ensure the selection sparsity. All integrative models were run

with five components for the downstream prognostic analyses, yielding comparable feature-set sizes across methods.

(2) In Results under "Comprehensive Analysis of Individual Cancer Data: Breast Cancer and Colorectal Cancer", added comparison in "Functional Analysis and Prognostic Comparison"

Figure 3c shows the four-way Venn diagrams of the significant pathways selected by MRF-IMD, SPLS, PMDCCA, and RGCCA in BRCA and COAD. In BRCA (left), MRF-IMD identified 55 unique pathways (25.3%), while SPLS, PMDCCA, and RGCCA only identified 27 (12.4%), 35 (16.1%), and 37 (17.1%) unique pathways, respectively. Only 37 pathways (17.1%) overlap between MRF-IMD and SPLS, and 40 (20.3%) between MRF-IMD and RGCCA; there are no shared pathways in PMDCCA or common to all four methods. The core oncogenic pathways such as *Constitutive Signaling by Aberrant PI3K in Cancer* and *PI3K AKT Signaling in Cancer* are uniquely retrieved by MRF-IMD that the linear integrators might miss. In COAD, MRF-IMD again dominates with 48 unique enrichments (64.0%), whereas SPLS yields none, PMDCCA yields 2 (2.7%), and RGCCA yields 12 (16.0%). Only 4 pathways (5.3%) overlap between MRF-IMD and RGCCA, and 2 (2.6%) between MRF-IMD and PMDCCA; there are no shared pathways in SPLS or common to all four methods. Among its unique COAD hits, MRF-IMD highlights *Collagen Fibril Organization* and *Ossification*, underscoring its ability to capture tissue-specific remodeling programs that go beyond the canonical and component-based integrators.

An important goal in cancer multi-omics studies is to identify biomarkers that not only reflect biological mechanisms but also correlate with clinical outcomes. To evaluate the prognostic value of the variables identified by our MRF-based methods, we applied integrative non-negative matrix factorization (IntNMF) method[23] to combine the selected variables from the three omics data types for both BRCA and COAD. This integration allowed us to cluster patients into two groups representing high- and low-risk survival profiles. Figure 3d shows the resulting Kaplan–Meier curves of the grouping results from clustering using all variables, top 5 SPLS components, and the variables selected MRF-IMD-mixture methods. Table 4 reports the median log-rank p-values across 30 seeds of MRF-IMD methods and log-rank p-values of other benchmark methods. In BRCA, clustering on all variables yields no significant separation (*P*-value =0.28), while SPLS and the CCA-based methods achieve modest significance only when using all five components (SPLS: *P*-value $=8.2\times10^{-3}$; PMDCCA: *P*-value $=1.4\times10^{-2}$; RGCCA: *P*-value $=2.4\times10^{-2}$) but fail when restricted to their selected features. In contrast, MRF-IMD's filter and mixture strategies produce highly significant stratification (median *P*-value $=7.9\times10^{-4}$ and 1.0×10^{-3} respectively), with the filter set delivering the strongest separation in the Kaplan–Meier curves (*P*-value $=4\times10^{-4}$). In COAD, neither the full feature set (*P*-value =0.24) nor SPLS selected variables-or RGCCA-derived signatures yield significant stratification. SPLS with all five components and PMDCCA's selected variables attain significance (*P*-

value=1.3×10^{-2} *and* 3.0×10^{-2}), but once again the MRF-IMD filter and mixture panels outperform, both achieving *P*-value =$1.2 - 1.4 \times 10^{-2}$.

Together, these results demonstrate that our MRF-IMD–derived biomarker sets consistently enable more robust risk stratification than existing integrative methods, confirming their potential clinical utility for patient stratification and prognostic modeling.

(3) In Results, under "TCGA PAN Cancer Clustering Analysis", revised the clustering analysis into a subsection "Pan-Cancer Clustering" and added SPLS clustering comparison

Pan-Cancer Clustering We next applied IntNMF directly to the MRF-IMD feature set and determined an optimal rank of eight clusters using `nmf.opt.k` function from the IntNMF R package. Figure 4d shows the resulting confusion matrix, illustrating how our selected features effectively separated the samples into eight clusters. Each cluster highlighted unique molecular characteristics and captured established patterns of tumor heterogeneity, ranging from a combined basal-like breast and uterine carcinoma cluster (Group 1) through gastrointestinal adenocarcinomas (Group 5) and hepatobiliary tumors (Group 2) to hypermutated immunogenic cancers (Group 3), squamous-cell carcinomas (Group 7), endocrine neoplasms (Group 6), and renal epithelial tumors (Group 8). A detailed description of the these clusters is in Table 5. To further quantify the advantage conferred by MRF-IMD feature selection, we applied IntNMF clustering to the pan-cancer dataset, aiming for 20 clusters. We first excluded cancer types with fewer than five samples (TCGA-CESC and TCGA-CHOL), as very small cohorts can produce unstable clusters driven by outliers or noise. We then quantified cluster recovery by computing the adjusted rand index (ARI) between the 20 IntNMF clusters on MRF-IMD features and the true TCGA tumor-type labels (Supplementary Figure 4a), benchmarking against four alternative strategies: PAM clustering on 30 SPLS components (Supplementary Figure 4b); IntNMF on the full feature set (Supplementary Figure 4c); PAM clustering on RNA-seq data alone (Supplementary Figure 4d); and PAM clustering on ATAC-seq data alone (Supplementary Figure 4e). Although all approaches are shown moderate to high performance, our method still shows advantage with slightly higher ARI of 0.728. Nearly every TCGA cohort is assigned to a single dominant cluster: COAD (36 out of 37 total samples in group 16), KIRP (31 out of 32 in group 7), PRAD (25 out of 26 in group 15), and UCEC (12 out of 12 in group 11) all show almost perfect one-to-one mapping. In breast cancer, the algorithm distinguished three biologically meaningful subgroups: Group 13 captured a pure basal-like subtype (all 13 basal tumors); Group 5 combined all HER2 (9 out of 10) and LumB (16 out of 16) cases with most LumA (18 out of 29) samples, reflecting a non-basal, high-risk profile; and Group 1 comprised predominantly LumA tumors (9 out of 11) alongside normal controls, defining a lower-risk, luminal-A–driven cluster. Smaller lineages such as BLCA, ESCA, and STAD likewise concentrate into their own clusters with minimal leakage. By contrast, the other methods produced more fragmented assignments and lower ARI scores, sPLS+PAM (ARI = 0.697), full-feature IntNMF (0.687), RNA-seq only (0.713), and ATAC-seq only (0.675), underscoring MRF-IMD's superior ability to isolate coherent, biologically relevant tumor groups.

4. Applicability to Other Diseases: The authors primarily focus on cancer datasets. It is recommended to discuss potential applicability to other disease contexts, such as neurodegenerative or immunological diseases, to illustrate broader utility.

We thank the reviewer for this insightful suggestion. To demonstrate the broader utility of our MRF-IMD framework beyond oncology, we have now added an ADNI analysis in the Results ("Integrative Analysis Enhances Prediction of Dementia Progression in the ADNI Cohort"), in which MRF-IMD on paired blood transcriptome and methylome data robustly prioritizes known Alzheimer's hubs (e.g. CCR7, EPHX2) and outperforms both a single-omics methylation risk score and unfiltered integrative clustering for dementia-conversion stratification.

Revision

(1) In Material and Methods, added "ADNI Data" under section "Real Data Preprocessing"

ADNI Data We analyzed data from the Alzheimer's Disease Neuroimaging Initiative (ADNI), including DNA methylation profiles for 538 cognitive normal (CN) and mild cognitive impaired (MCI) patients measured on Illumina HumanMethylation EPIC v1 arrays preprocessed by Zhang et al.[24] and matched gene-expression data from Affymetrix Human Genome U219 microarrays. In this study, we mainly focus on dementia onsite. Given the heterogeneity and complex progression of Alzheimer's disease (AD), we first selected the top 2,000 CpG methylation sites most significantly associated with AD dementia onsite, using p-value–based screening in the Framingham Heart Study (FHS) dataset as described by Zhang et al.[24] For expression, we removed probes lacking gene symbols, flagged and dropped any probe with gene expression below the 10th percentile in over 80% of samples, collapsed remaining probes by gene via median values, and finally selected the 2,000 most variable genes. Dementia conversion was defined as the conversion from CN to MCI or dementia, and from MCI to dementia. After intersecting on subject IDs, we obtained 468 common samples for all integrative analyses. DNA methylation, gene expression data and the dementia status of the subjects were obtained from the ADNI study website (adni.loni.usc.edu). Table 2 summarizes the datasets used in the analysis.

(2) In Results added section "Integrative Analysis Enhances Prediction of Dementia Progression in the ADNI Cohort"

Integrative Analysis Enhances Prediction of Dementia Progression in the ADNI Cohort

MRF-IMD Selected Genes To further illustrate the superior results of our MRF-IMD method, we applied the variable selection to the ADNI data using the filtering strategy. A total of 161 genes and 54 CpG sites were selected by the strategy. Figure 5a shows the top twenty gene-expression and DNA methylation features prioritized by our MRF-IMD framework in the ADNI cohort. On the left panel, *ARL11* has the largest weights. While its direct role in AD is still under investigation, *ARL11* is known to be involved in apoptosis and immune system processes, which are critical components of neuroinflammation in AD[25]. Followed by *ARL11*, *S1PR1*

plays a significant role in the neuroinflammatory processes of AD[26]. *DAPK2* indicates the involvement of death-associated kinase-mediated neuronal apoptosis and tau dysregulation[27], although the precise nature of its contribution to AD pathology requires further investigation. *CCR7* reflects its established involvement in chemokine-mediated microglial trafficking and neuroinflammation, with studies indicating that reduced *CCR7* expression on meningeal T cells in aging is linked to worsened glymphatic function, cognition, neuroinflammation, and β –amyloid pathology[28]. Functional analysis of the selected genes identified 36 pathways with FDR < 0.05 (Supplementary Table 2). The enrichment profile was dominated by Lymphocyte (B cells, T cells, NK cells) programs, especially in T cells. Key terms included *T Cell Differentiation and Activation*, *Lymphocyte Differentiation and Activation*, supporting a peripheral inflammatory state relevant to Alzheimer's disease[29,30].

Pathway Analysis of Selected DNAm CpGs To assess the biological relevance of our MRF-IMD selected CpGs versus those significant CpGs reported by Zhang et al., we performed KEGG and GO enrichment with the missMethyl R package on both the 54 MRF-IMD prioritized sites (top 20 out of 54 sites showed in Figure 5a right panel) and the 44 meta-analysis significant sites. Supplementary Table 3 displays the top 15 most significant pathways for each method. In the KEGG analysis, MRF-IMD CpGs showed strongest enrichment in *NF-κB Signaling*, *C-type lectin Receptor Signaling*, and *Leukocyte Transendothelial Migration*. Together these pathways indicate coordinated innate immune activation and immune-cell trafficking across the endothelium, processes that escalate neuroinflammation and contribute to AD progression. These results are concordant with the pathway enrichments obtained from the selected gene set. By contrast, the Zhang et al. meta-analysis CpGs were enriched for viral-infection and adhesion processes, top hits were *Virion–Ebolavirus, Lyssavirus and Morbillivirus*, *Cell Adhesion Molecules* and *mTOR Signaling*, indicating a shift toward pathogen-related and cell–matrix interaction pathways.

In the GO analysis, MRF-IMD CpGs emphasized post-transcriptional RNA processing (*Polyribonucleotide Nucleotidyltransferase Activity*; *Nuclear and Mitochondrial Polyadenylation-Dependent mRNA Catabolic Processes*; *Poly(U) RNA Binding*), along with cytokine regulation via I*nterleukin-1 Type I/II Receptor Antagonist Activity* and neuromodulatory pathways including *Wnt Signaling* and *Galanin Receptor Binding (Types 1–3)*. Notably, these CpG-derived enrichments align with the gene-based pathways through shared immune modules such as *Interleukin-1 Receptor Antagonist Activity* and *Wnt Signaling*, while the RNA processing and galanin receptor terms appear CpG-specific. In contrast, the meta-analysis CpGs were dominated by cell-division and cytokinesis terms (*Protein Localization to Division Site*; *Cleavage Furrow*; *Mitotic Cytokinetic Regulation*). These comparisons underscore that our MRF-IMD approach yields CpGs tied to innate immune signaling, mRNA processing and metabolic regulation that were more detailed and emphasized core processes directly associated in AD.

Integrative Validation on Dementia Progression To demonstrate that our integrative variable-selection outperforms both the single-layer methylation risk score (MRS) from Zhang et al. and an integrative approach without feature selection, we compared three stratifications of dementia conversion. We applied

intNMF to the MRF-IMD selected features, obtaining two clusters, and evaluated time to dementia conversion using Kaplan–Meier curves and a log-rank test. The MRF-IMD panel showed a significant separation (Figure 5b; *P*-value = 0.033). We benchmarked three alternatives (Figure 5c): (left) intNMF on the full, unfiltered full omics set (*P*-value = 0.048); (middle) the published MRS based on 151 CpGs, dichotomized at the data-driven cut-point using MaxStat (*P*-value = 0.048); and (right) intNMF on the first five SPLS components from the full omics set (*P*-value = 0.60). Across comparisons, the MRF-IMD hubs delivered the strongest prognostic discrimination, outperforming methylation-only scoring and unfiltered or dimension-reduced integration.

In the original Zhang et al. study, the MRS was built via ridge regression on CpGs significantly associated with conversion and tested in a multivariate Cox model adjusted for age, sex, APOE ϵ4 status, years of education, baseline diagnosis, and baseline MMSE score (Surv(conversion event, follow-up) ~ MRS + covariates). When we substituted our IntNMF-derived component from the MRF-IMD features into this identical Cox framework with the MRS, we observed a stronger association with progression to the next disease stage (Table 6). This result confirms that our integrative variable-selection not only refines molecular subtyping but also enhances prediction of disease progression beyond both single-omics risk scores and non-prioritized integrative analyses.

Integrative Validation on Dementia Progression To demonstrate that our integrative variable-selection outperforms both the single-layer methylation risk score (MRS) from Zhang et al. and an integrative approach without feature selection, we compared three stratifications of dementia conversion (as defined in Zhang et al.). First, we dichotomized the published MRS using 151 CpGs at cut off determined by maximum rank statistic, which implemented in the MaxStat R package, and evaluated its Kaplan–Meier survival curves with a log-rank test. Second, we applied intNMF clustering to the full set of omics variables and assessed the resulting subgroups in the same way. Third, we repeated the intNMF procedure using only our MRF-IMD-prioritized features. As shown in Figure 5b, the MRF-IMD panel achieved the most significant separation (lowest P-value), indicating that focusing on cross-omics hub variables yields stronger prognostic discrimination than using methylation alone or an unfiltered integrative feature set.

In the original Zhang et al. study, the MRS was built via ridge regression on CpGs significantly associated with conversion and tested in a multivariate Cox model adjusted for age, sex, APOE ε4 status, years of education, baseline diagnosis, and baseline MMSE score (Surv(conversion event, follow-up) ~ MRS + covariates). When we substituted our intNMF-derived component from the MRF-IMD features into this identical Cox framework, we observed a stronger association with progression to the next disease stage (Table 7). This result confirms that our integrative variable-selection not only refines molecular subtyping but also enhances prediction of disease progression beyond both single-omics risk scores and non-prioritized integrative analyses.

5. Improved Visualization: Some figures in the manuscript have font sizes that are too small, which might impair readability. It is recommended to enlarge the text labels, legends, and axis annotations to ensure that all information is clearly visible and accessible. In Figure 8, the use of sub-labels (such as a, b, c) is mentioned in the text, but these labels are not visible in the figure itself.

We thank the reviewer for pointing out these important visualization issues. In response, we have revised all figures throughout the manuscript to improve overall clarity and readability.

Reviewer #2

The article presents an Integrative Multi-Omics Random Forest Framework for Robust Biomarker Discovery. It addresses the challenge of extracting key shared biomarkers from multiple omics data types by introducing a multivariate random forest-based approach enhanced by an inverse minimal depth metric.

We are grateful for your thoughtful evaluation and constructive feedback. All revisions are highlighted in red in the updated manuscript to facilitate your review. We believe these changes comprehensively address your concerns and significantly enhance the clarity, rigor, and overall presentation of our work. Thank you again for your valuable suggestions.

I have some concerns and comments below:

1. The new algorithm described in the study selected omics variables by assigning response variable to decision tree nodes. How the response variables relate to biological responses/outcomes? From the authors' description, it seems that the selected omics variables using the IMD are almighty, i.e., they can predict anything needed, such as prognosis, cancer types, and et al. Actually, the usual logic to select omics variables to predict prognosis is to evaluate the association between omics variables and survival time.

We appreciate the reviewer for this important question. We would like to clarify that our integrative variable selection method is an unsupervised procedure which no clinical labels ever enter the model in the training process. The "response" variable that the forest models is simply a second omics layer (e.g. miRNA when mRNA is the predictor set, or vice versa), not a clinical endpoint like survival time or tumor subtype. The inverse minimal depth (IMD) metric quantifies how early each variable appears in the splitting hierarchy of the forest, reflecting its importance in mediating cross-omics variation. Higher IMD values indicate that the selected variables are more likely to share information across layers, acting as hubs in the multi-omics network. These hubs coordinate core biological pathways, such as cell cycle control, DNA repair, and immune signaling, that both define molecular subtypes and drive differences in patient outcomes.

When these IMD-selected hubs are carried forward into downstream analyses, whether consensus clustering for subtyping or prognosis test, they yield more stable, biologically coherent clusters and higher prognostic accuracy than feature panels chosen by classical univariate survival filtering or single-omics integration methods. We agree with the reviewer that outcome-guided preselection can further strengthen variable choice, especially for a complex disorder such as Alzheimer's disease (AD). Accordingly, we added a new ADNI analysis of AD dementia onset in which methylation CpGs were preselected from the epigenome-wide association study by Zhang et al.[24] Incorporating this EWAS-informed prior into our model produced improved clustering stability and prognostic accuracy while preserving interpretability. We have revised the Methods and Results sections and added the corresponding figure and table to document these updates. We have revised our manuscript as follows:

Revision

(1) In **Introduction**, added a detailed explanation and references

Recent technological advances in high-throughput sequencing, mass spectrometry, and imaging have led to a surge in multi-omics data that span the genome, epigenome, transcriptome, proteome, and metabolome. However, each type of data alone captures only a slice of disease biology. Integrating these diverse data sources can provide a more comprehensive picture of complex biological systems than analyzing any single omics layer alone. Multi-omics analysis has been implemented in many studies for biomarker discovery, disease subtyping, and disease insights . A key goal in multi-omics integration is to extract "shared" biomarkers from multiple data – that is, to identify molecular features that are consistently important across different omics platforms. These biomarkers typically indicate robust, system-level regulatory mechanisms that single-omics analyses may miss[31,32]. Furthermore, integrating complementary data sources reduces noise and mitigates biological heterogeneity, enhancing the precision and clinical relevance of patient stratification and prognosis[33]. In general, multi-omics approaches tend to yield more reliable biomarkers and disease signatures than single-modality analyses, as demonstrated in recent studies: methods like DIABLO, which based on the sparse partial least squares (SPLS) method, seek common information across data types by selecting subsets of features that jointly capture variance in each dataset[34]. When done effectively, integration can highlight shared molecular features across different data types, offering new insights into disease mechanisms, patient stratification, and potential biomarkers for clinical applications[34–37].

(2) In **Introduction**, added clarification on method description

In this study, we introduce a new MRF-based framework that employs the inverse minimal depth (IMD) metric for unsupervised variable selection across multiple omics datasets. We model the relationships between two omics by assigning one omics to the response space in and the other omics to the feature space in an ensemble of decision trees. After fitting the forest, we compute the IMD to quantify feature importance and identify key variables shared across different data layers. We then extend our framework from pairwise (two-omics) integration to a comprehensive multi-omics approach by modeling different layer pairs guided by prior knowledge or precomputed inter-relationships. This strategy naturally reduces the risk of selecting noise variables and helps focus on those with consistent impact across datasets.

(3) In Material and Methods, added "ADNI Data" under section "Real Data Preprocessing"

ADNI Data We analyzed data from the Alzheimer's Disease Neuroimaging Initiative (ADNI), including DNA methylation profiles for 538 cognitive normal (CN) and mild cognitive impaired (MCI) patients measured on Illumina HumanMethylation EPIC v1 arrays preprocessed by Zhang et al.[24] and matched gene-expression data from Affymetrix Human Genome U219 microarrays. In this study, we mainly focus on dementia onsite. Given the heterogeneity and complex progression of Alzheimer's disease (AD), we first selected the top 2,000 CpG methylation sites most significantly associated with AD dementia onsite, using p-value–based

screening in the Framingham Heart Study (FHS) dataset as described by Zhang et al.[24] For expression, we removed probes lacking gene symbols, flagged and dropped any probe with gene expression below the 10th percentile in over 80% of samples, collapsed remaining probes by gene via median values, and finally selected the 2,000 most variable genes. Dementia conversion was defined as the conversion from CN to MCI or dementia, and from MCI to dementia. After intersecting on subject IDs, we obtained 468 common samples for all integrative analyses. DNA methylation, gene expression data and the dementia status of the subjects were obtained from the ADNI study website (adni.loni.usc.edu). Table 2 summarizes the datasets used in the analysis.

(4) In Results added section "Integrative Analysis Enhances Prediction of Dementia Progression in the ADNI Cohort"

Integrative Analysis Enhances Prediction of Dementia Progression in the ADNI Cohort

MRF-IMD Selected Genes To further illustrate the superior results of our MRF-IMD method, we applied the variable selection to the ADNI data using the filtering strategy. A total of 161 genes and 54 CpG sites were selected by the strategy. Figure 5a shows the top twenty gene-expression and DNA methylation features prioritized by our MRF-IMD framework in the ADNI cohort. On the left panel, *ARL11* has the largest weights. While its direct role in AD is still under investigation, *ARL11* is known to be involved in apoptosis and immune system processes, which are critical components of neuroinflammation in AD[25]. Followed by *ARL11*, *S1PR1* plays a significant role in the neuroinflammatory processes of AD[26]. *DAPK2* indicates the involvement of death-associated kinase-mediated neuronal apoptosis and tau dysregulation[27], although the precise nature of its contribution to AD pathology requires further investigation. *CCR7* reflects its established involvement in chemokine-mediated microglial trafficking and neuroinflammation, with studies indicating that reduced *CCR7* expression on meningeal T cells in aging is linked to worsened glymphatic function, cognition, neuroinflammation, and β –amyloid pathology[28]. Functional analysis of the selected genes identified 36 pathways with FDR < 0.05 (Supplementary Table 2). The enrichment profile was dominated by Lymphocyte (B cells, T cells, NK cells) programs, especially in T cells. Key terms included *T Cell Differentiation and Activation*, *Lymphocyte Differentiation and Activation*, supporting a peripheral inflammatory state relevant to Alzheimer's disease[29,30].

Pathway Analysis of Selected DNAm CpGs To assess the biological relevance of our MRF-IMD selected CpGs versus those significant CpGs reported by Zhang et al., we performed KEGG and GO enrichment with the missMethyl R package on both the 54 MRF-IMD prioritized sites (top 20 out of 54 sites showed in Figure 5a right panel) and the 44 meta-analysis significant sites. Supplementary Table 3 displays the top 15 most significant pathways for each method. In the KEGG analysis, MRF-IMD CpGs showed strongest enrichment in *NF-κB Signaling*, *C-type lectin Receptor Signaling*, and *Leukocyte Transendothelial Migration*. Together these pathways indicate coordinated innate immune activation and immune-cell trafficking across the endothelium, processes that escalate neuroinflammation and contribute to AD progression. These

results are concordant with the pathway enrichments obtained from the selected gene set. By contrast, the Zhang et al. meta-analysis CpGs were enriched for viral-infection and adhesion processes, top hits were *Virion–Ebolavirus, Lyssavirus and Morbillivirus*, *Cell Adhesion Molecules* and *mTOR Signaling*, indicating a shift toward pathogen-related and cell–matrix interaction pathways.

In the GO analysis, MRF-IMD CpGs emphasized post-transcriptional RNA processing (*Polyribonucleotide Nucleotidyltransferase Activity*; *Nuclear and Mitochondrial Polyadenylation-Dependent mRNA Catabolic Processes*; *Poly(U) RNA Binding*), along with cytokine regulation via I*nterleukin-1 Type I/II Receptor Antagonist Activity* and neuromodulatory pathways including *Wnt Signaling* and *Galanin Receptor Binding (Types 1–3)*. Notably, these CpG-derived enrichments align with the gene-based pathways through shared immune modules such as *Interleukin-1 Receptor Antagonist Activity* and *Wnt Signaling*, while the RNA processing and galanin receptor terms appear CpG-specific. In contrast, the meta-analysis CpGs were dominated by cell-division and cytokinesis terms (*Protein Localization to Division Site*; *Cleavage Furrow*; *Mitotic Cytokinetic Regulation*). These comparisons underscore that our MRF-IMD approach yields CpGs tied to innate immune signaling, mRNA processing and metabolic regulation that were more detailed and emphasized core processes directly associated in AD.

Integrative Validation on Dementia Progression To demonstrate that our integrative variable-selection outperforms both the single-layer methylation risk score (MRS) from Zhang et al. and an integrative approach without feature selection, we compared three stratifications of dementia conversion. We applied intNMF to the MRF-IMD selected features, obtaining two clusters, and evaluated time to dementia conversion using Kaplan–Meier curves and a log-rank test. The MRF-IMD panel showed a significant separation (Figure 5b; *P*-value = 0.033). We benchmarked three alternatives (Figure 5c): (left) intNMF on the full, unfiltered full omics set (*P*-value = 0.048); (middle) the published MRS based on 151 CpGs, dichotomized at the data-driven cut-point using MaxStat (*P*-value = 0.048); and (right) intNMF on the first five SPLS components from the full omics set (*P*-value = 0.60). Across comparisons, the MRF-IMD hubs delivered the strongest prognostic discrimination, outperforming methylation-only scoring and unfiltered or dimension-reduced integration.

In the original Zhang et al. study, the MRS was built via ridge regression on CpGs significantly associated with conversion and tested in a multivariate Cox model adjusted for age, sex, APOE $\epsilon 4$ status, years of education, baseline diagnosis, and baseline MMSE score (Surv(conversion event, follow-up) $\sim$ MRS + covariates). When we substituted our IntNMF-derived component from the MRF-IMD features into this identical Cox framework with the MRS, we observed a stronger association with progression to the next disease stage (Table 6). This result confirms that our integrative variable-selection not only refines molecular subtyping but also enhances prediction of disease progression beyond both single-omics risk scores and non-prioritized integrative analyses.

Integrative Validation on Dementia Progression To demonstrate that our integrative variable-selection outperforms both the single-layer methylation risk score (MRS) from Zhang et al. and an integrative approach without feature selection, we compared three stratifications of dementia conversion (as defined in Zhang et al.). First, we dichotomized the published MRS using 151 CpGs at cut off determined by maximum rank statistic, which implemented in the MaxStat R package, and evaluated its Kaplan–Meier survival curves with a log-rank test. Second, we applied intNMF clustering to the full set of omics variables and assessed the resulting subgroups in the same way. Third, we repeated the intNMF procedure using only our MRF-IMD-prioritized features. As shown in Figure 5b, the MRF-IMD panel achieved the most significant separation (lowest P-value), indicating that focusing on cross-omics hub variables yields stronger prognostic discrimination than using methylation alone or an unfiltered integrative feature set.

In the original Zhang et al. study, the MRS was built via ridge regression on CpGs significantly associated with conversion and tested in a multivariate Cox model adjusted for age, sex, APOE ε4 status, years of education, baseline diagnosis, and baseline MMSE score (Surv(conversion event, follow-up) ~ MRS + covariates). When we substituted our intNMF-derived component from the MRF-IMD features into this identical Cox framework, we observed a stronger association with progression to the next disease stage (Table 7). This result confirms that our integrative variable-selection not only refines molecular subtyping but also enhances prediction of disease progression beyond both single-omics risk scores and non-prioritized integrative analyses.

2. Following the discussion in 1, what is the biological meaning to extract shared biomarkers from multiple data layers? While it is straightforward to think that the shared biomarkers between multiple data layers or data types may induce the same biological responses, the unique biomarkers also matter depending on what biological responses we care.

Thank you for raising this point. We agree that, alongside cross-omics “shared” signals, layer-specific biomarkers can be biologically informative depending on the phenotype under study. Our framework intentionally prioritizes biomarkers that shared similar information, which are features whose variation is concordant across omics, because these tend to capture upstream regulatory programs, are less sensitive to platform-specific noise, and thus yield more stable, clinically transferable signatures[33]. This focus does not exclude omics-specific biology: IMD will still retain strong within-omics features when they help explain cross-omics variation. In the revision, we clarify this rationale and outline a simple extension (“shared-then-unique”) that first prioritizes shared hubs with MRF-IMD and, when a specific endpoint is of interest (e.g., survival), augments the panel with omics-specific markers tailored to that phenotype.

Looking ahead, we plan to extend the framework to simultaneously extract both shared cross-omics hubs and unique omics-specific signatures, thereby tailoring biomarker discovery to diverse

biological and clinical objectives. To address this point, we have revised the Introduction as described in Comment 1, and we have added a new limitation paragraph in the Discussion to highlight these considerations and outline our planned extensions.

Revision

(1) In **Introduction**, added detailed explanation and references (see Revision (1) in Comment 1)

(2) In **Discussion**, added limitation

While our method provides clear advantages, some limitations remain. First, computation time may increase with more datasets and extreme high-dimensionality. Future research could focus on improving efficiency, potentially through parallelization or dimensionality reduction strategies that preserve essential biological signals. Second, further integration with downstream validation steps, such as experimental verification or functional assays, would help confirm the biological significance of the selected variables and strengthen the evidence for potential biomarkers. Third, although we prioritize cross-omics shared biomarkers to capture system-level regulators, we recognize that unique, omics-specific features (e.g., methylation marks reflecting environmental exposure or miRNAs mediating post-transcriptional control) also carry important biological information. Future work in developing a promising extension of the framework that extracts both omics-specific and shared biomarkers would enable more comprehensive biological insights.

3. The Introduction section is not sufficient. The biological significance and technical details of "extract shared biomarkers from multiple data layers" need to be explained in more details.

We thank the reviewer for the suggestion on adding the explanation for "extract shared biomarkers from multiple data layers". In response, we have expanded the Introduction (see Comment 1). Additionally, we also revised our Introduction section by adding more details and description of our methods, simulations, and data applications.

Revision

(1) In **Introduction**, added detailed explanation

A key goal in multi-omics integration is to extract “shared” biomarkers from multiple data layers – that is, to identify molecular features that are biologically relevant consistently across different omics types. These biomarkers typically indicate robust, system-level regulatory mechanisms that single-layer analyses may miss[31,32].

(2) In **Introduction**, added clarification on method description and summary of applications

In this study, we introduce a new MRF-based framework that employs the inverse minimal depth (IMD) metric for unsupervised variable selection across multiple omics datasets. We model the relationships between each pair of two omics by assigning one omics to the response space in and the other omics to

the feature space in an ensemble of decision trees. After fitting the forest, we compute the IMD to quantify feature importance. We then extend our framework from pairwise (two-omics) integration to a multi-omics approach by modeling different layer pairs guided by prior knowledge or precomputed inter-relationships. This strategy reduces the risk of selecting noise variables and helps focus on those with consistent impact across datasets. To show that our method can effectively capture shared biomarkers in complex datasets, we benchmarked it against established integration approaches, including SPLS, CCA, and several nonlinear ensemble methods such as gradient boosting machine (GBM) and XGBoost through multiple simulations. We found that methods like SPLS and CCA are not stable in capturing the important features when data types are in nonlinear or contains interaction settings. Moreover, we validated our framework using several clinical cohorts, including TCGA-BRCA and TCGA-COAD, demonstrating superior ability to uncover biologically relevant pathways and to stratify patients by prognostic outcome compared to traditional integration methods such as SPLS and CCA. We further applied our approach to the TCGA-PANCAN and ADNI datasets, identifying biomarker panels tied to key biological pathways that show promise for enhancing molecular subtyping of PAN cancer, and prognosis of dementia onset.

4. It is advised to provide some examples of the statement in the Introduction: "may fail to capture nonlinear interactions" of the current methods (sPLS, CCA).

We appreciate the reviewer's advice on providing some examples of the current methods, such as SPLS or CCA may fail to capture nonlinear interaction. We have provided an example in the Introduction section that illustrates how these methods might fail in capturing variables that interact multiplicatively. In addition, we also added a three-way non-linear interaction model in the Simulation section to further demonstrates this limitation. Figure 2 (and new Supplementary Figure 2 in the revised manuscript) below shows that the SPLS and two CCA based methods failed to recover pure higher-order interactions.

Figure 2

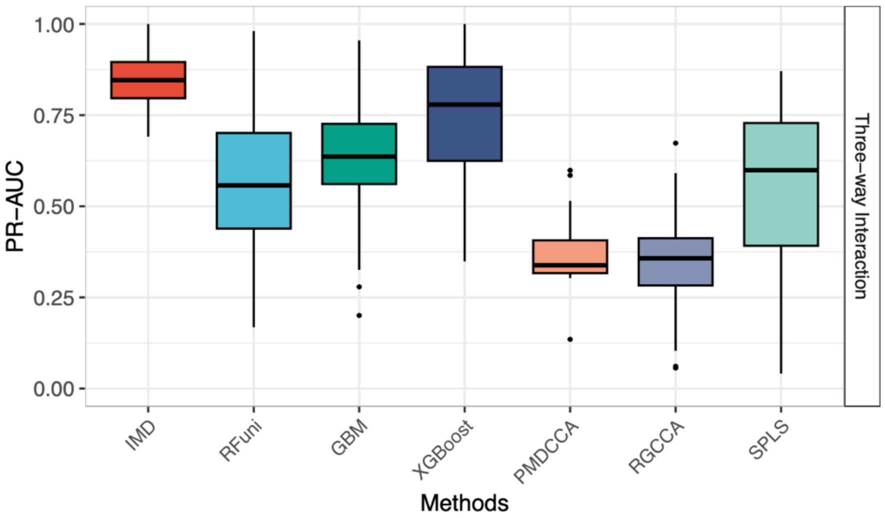

In response, we did the following revision:

Revision

In **Introduction**, added example of why current methods (SPLS, CCA) may fail to capture nonlinear interaction

Despite the promise of multi-omics integration, extracting shared signals across heterogeneous datasets remains challenging. Traditional penalized integration methods, such as SPLS[34,38,39] and canonical correlation analysis (CCA) [40–42], focus largely on linear relationships. Although widely used, these approaches can struggle in high-dimensional settings, are prone to overfitting, and may fail to capture nonlinear interactions. For example, in "XOR" simulation where two variables interact multiplicatively to determine the response, both SPLS or CCA, which optimize linear covariance/correlation, tend to yield near-random feature rankings and fail to recover the interacting pair (as we also show in our simulation study). Nonlinear extensions, including kernel CCA[43,44], help address some of these issues but often face scalability and interpretability limitations, making them less suitable for many practical scenarios.

In Material and Method, under "Simulation Study", added a "Three-way non-linear interaction" model

Three-way non-linear interaction To further challenge the ability of each method to uncover higher order effects for the ranking based comparison, we simulated a purely nonlinear three-way interaction. We generated $n = 200$ samples of $p = 300$ independent predictors $X_i \sim N(0,1)$, then selected (X_1, X_2, X_3) as the shared interacting trio and added two "side" predictions per outcome (X_4, X_5) for Y_1 and (X_6, X_7) for Y_2. Specifically, the latent signals were

$$\eta_1 = (X_1^2 - 1)(X_2 + 0.5)(X_3 - 0.5) + X_4 + X_5, \quad \eta_2 = (X_1^2 - 0.5)(X_2 + 0.5)(X_3 - 0.5) + X_6 - X_7.$$

and set the corresponding response

$$Y_1 = 1.5\eta_1 + \epsilon_1,\ \ Y_2 = 2\eta_2 + \epsilon_2 \quad \epsilon_{k\in 1,2} \sim \mathcal{N}(0, \sigma^2),$$

After centering and scaling all columns of X and Y to unit variance, we tasked each algorithm with ranking the full set of 300 predictors. We then compared nonlinear ensemble learners (random forests with permutation-importance, GBM, XGBoost) against multi-omics integration methods (SPLS, PMDCCA, SGCCA) by measuring each method's PR–AUC and true-positive rate among the top kp_c features per response.

5. It is also advised to explain and illustrate how the new method proposed in this study addressed the challenge of traditional methods for capturing nonlinear relationships. Ablation study could be one of the choices.

We thank the reviewer for this important suggestion. Following the response in Comment 4, we revised our simulation study to better illustrate our method's advantage in capturing nonlinear relationships. Specifically, we added a three-way nonlinear interaction model in the Simulation Study section to compare the performance of our approach with current methods, including SPLS, CCA, and several popular nonlinear ensemble methods (univariate random forests, gradient boosting machines [GBM], and XGBoost). All related changes are highlighted in red in the revised manuscript within the Materials and Methods section (under "Simulation Study") and the Results section (under "Evaluation of Simulated Data"). The resulting PR-AUC (Figure 2 [and new Supplementary Figure 2 in the revised manuscript]) demonstrates our method's clear advantage in detecting nonlinear interaction effects that traditional integrative methods often fail to capture.

In particular, we did the following revision:

Revision

(1) In Material and Method, under "Simulation Study", added a "Three-way non-linear interaction" model

Three-way non-linear interaction To further challenge the ability of each method to uncover higher order effects for the ranking based comparison, we simulated a purely nonlinear three-way interaction. We generated $n = 200$ samples of $p = 300$ independent predictors $X_i \sim N(0,1)$, then selected (X_1, X_2, X_3) as the shared interacting trio and added two "side" predictions per outcome (X_4, X_5) for Y_1 and (X_6, X_7) for Y_2. Specifically, the latent signals were

$$\eta_1 = (X_1^2 - 1)(X_2 + 0.5)(X_3 - 0.5) + X_4 + X_5, \quad \eta_2 = (X_1^2 - 0.5)(X_2 + 0.5)(X_3 - 0.5) + X_6 - X_7.$$

and set the corresponding response

$$Y_1 = 1.5\eta_1 + \epsilon_1,\ \ Y_2 = 2\eta_2 + \epsilon_2 \quad \epsilon_{k\in 1,2} \sim \mathcal{N}(0, \sigma^2),$$

After centering and scaling all columns of X and Y to unit variance, we tasked each algorithm with ranking the full set of 300 predictors. We then compared nonlinear ensemble learners (random forests with permutation-importance, GBM, XGBoost) against multi-omics integration methods (SPLS, PMDCCA, SGCCA) by measuring each method's PR–AUC and true-positive rate among the top kp_c features per response.

All MRF-IMD models were implemented via the *rfsrc* function in the randomForestSRC R package, using its out-of-the-box settings (default mtry, nodesize, and splitting rules) across every simulation scenario. For the integration variable selection comparison, we compared our methods to SPLS (mixOmics R package), PMDCCA (PMA R package), and SGCCA (RGCCA R package). For PMDCCA and SGCCA, we selected variables were tuned by the build-in tuning functions of their packages. For SPLS, we directly entered the number of true variables to the function. For the ranking based comparison, we added the ensemble methods, random forests (permutation-importance), GBM, and XGBoost and recorded the importance scores that generated by their respective R packages (randomForest, gbm, and xgboost). For the integrative methods (SPLS, PMDCCA, and SGCCA), we collapsed each into a single ranking by averaging the absolute loadings over the first five components. This approach ensured that every method returned a complete ranking over the same candidate set.

(2) In Results, under "Evaluation of Simulated Data", refined the summary of benchmark with the existing methods

Integrative Variable Selection Benchmark The variable selection results are summarized in Figure 2 and Table 3 (Supplementary Note 7). Under the latent-factor model (Figure 2a, Table 3a), all three MRF-IMD variants delivered competitive PR-AUC and TPR compared with SPLS, PMDCCA, and SGCCA, matching these methods in the very linear scenarios they were designed for. By contrast, in the nonlinear regression simulations (Figure 2b, Table 3b), IMD-filter, IMD-mixture, and IMD-transformation significantly outperformed the CCA-based integrators, maintaining high PR-AUC and stable TPR even as model complexity increased. Among the IMD methods, filter was most conservative, mixture struck the strongest sensitivity–specificity balance, and transformation excelled at highlighting signals when IMD weights were tightly clustered. Extended simulation results are provided in Supplementary Note 7.

6. The authors showed that their new approach "uncovered known cancer biological relevant pathways". How about the functional enrichment of genes selected from traditional methods, such as sPLS, CCA?

We appreciate the reviewer's suggestion on adding the functional enrichment analysis using traditional methods. to include a direct comparison of functional enrichment results from traditional integrative methods. Accordingly, we re-analyzed the TCGA-BRCA and TCGA-COAD cohorts by running SPLS, PMDCCA, and RGCCA with penalty parameters chosen to yield a total

number of features matching our MRF-IMD selections. We then applied identical pathway enrichment analyses to each method's gene sets (FDR < 0.05). Figure 3 (and new Figure 3c in the revised manuscript) presents Venn diagrams of the significant pathways identified by all four approaches. MRF-IMD recapitulated a large number of pathways in both cohorts. It uniquely recovered core oncogenic signatures, such as "Constitutive Signaling by Aberrant PI3K in Cancer" and "PI3K AKT Signaling in Cancer" in BRCA, and "Collagen Fibril Organization" and "Ossification" in COAD, that SPLS, PMDCCA, and RGCCA failed to detect. These results underscore MRF-IMD's superior ability to uncover both shared and novel pathway-level insights from multi-omics data.

Figure 3

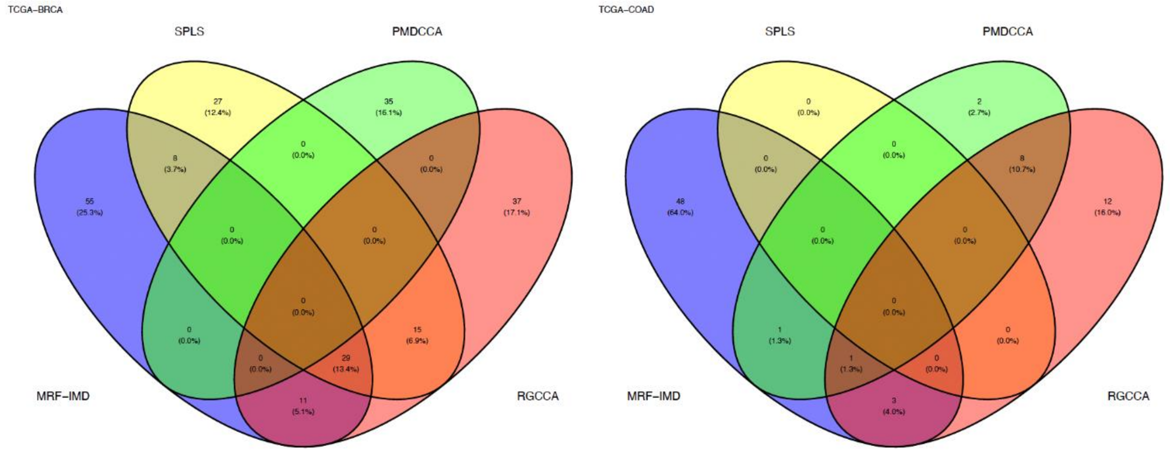

In the manuscript, we revised the following sections:

Revision

(1) In Results under "Comprehensive Analysis of Individual Cancer Data: Breast Cancer and Colorectal Cancer", added subsection "Signature Selection for Comparative Evaluation"

Signature Selection for Comparative Evaluation For the rest of the analysis, we adopted the IMD-mixture variable selection strategy as it delivers a balanced signature size (neither too sparse nor overly broad) while maintaining high selection stability across seeds. For reproducible research, we selected the seed for the model fitting that was closest to the median across 30 seeds. To enable a fair head-to-head evaluation, we then configured SPLS, PMDCCA, and RGCCA to yield the similar total number of features. For SPLS (mixOmics), we specified the `keepX` vector in `block.spls` function to match the per-component counts from IMD-mixture. PMDCCA (PMA) was run with its default `CCA.permute` routine, which, if no penalty is supplied, automatically selects optimal penalty terms via permutation testing. For RGCCA (RGCCA), we manually set the shrinkage penalties to 0.1 to ensure the selection sparsity. All integrative models were run

with five components for the downstream prognostic analyses, yielding comparable feature-set sizes across methods.

(2) In Results under "Comprehensive Analysis of Individual Cancer Data: Breast Cancer and Colorectal Cancer", added functional enrichment analysis comparison in subsection "Functional Analysis and Prognostic Comparison"

Figure 3c shows the four-way Venn diagrams of the significant pathways selected by MRF-IMD, SPLS, PMDCCA, and RGCCA in BRCA and COAD. In BRCA (left), MRF-IMD identified 55 unique pathways (25.3%), while SPLS, PMDCCA, and RGCCA only identified 27 (12.4%), 35 (16.1%), and 37 (17.1%) unique pathways, respectively. Only 37 pathways (17.1%) overlap between MRF-IMD and SPLS, and 40 (20.3%) between MRF-IMD and RGCCA; there are no shared pathways in PMDCCA or common to all four methods. The core oncogenic pathways such as *Constitutive Signaling by Aberrant PI3K in Cancer* and *PI3K AKT Signaling in Cancer* are uniquely retrieved by MRF-IMD that the linear integrators might miss. In COAD, MRF-IMD again dominates with 48 unique enrichments (64.0%), whereas SPLS yields none, PMDCCA yields 2 (2.7%), and RGCCA yields 12 (16.0%). Only 4 pathways (5.3%) overlap between MRF-IMD and RGCCA, and 2 (2.6%) between MRF-IMD and PMDCCA; there are no shared pathways in SPLS or common to all four methods. Among its unique COAD hits, MRF-IMD highlights *Collagen Fibril Organization* and *Ossification*, underscoring its ability to capture tissue-specific remodeling programs that go beyond the canonical and component-based integrators.

7. The authors showed that the selected RNA-seq and ATAC-seq features using the new approach are able to capture the distinction between different cancer types (Figure 8). It is suggested to quantitatively evaluate this capability using metrics of recall, precision, and et al. to calculate how many samples are corrected classified and how many are mis-classified in comparison with other methods.

We thank the reviewer for the suggestion to provide a more detailed quantitative evaluation of our pan-cancer clustering. To address this, we have substantially expanded the "TCGA Pan Cancer Clustering Analysis" section to describe our full analysis pipeline. Specifically, we now explain that we applied IntNMF to the MRF-IMD–selected features across 20 cancer types, producing 20 clusters (we excluded cancer types with fewer than five samples). We then computed the adjusted Rand index (ARI)—a chance-adjusted measure of agreement between two partitions that ranges from –1 (no agreement) to 1 (perfect agreement)—to compare these clusters against the true TCGA tumor-type labels. ARI is commonly used in clustering evaluations because it accounts for both co-assigned positive and negative sample pairs without requiring a "positive class" definition.

We also clarify why we did not include precision or recall metrics: in unsupervised clustering, these measures assume binary class labels and can be misleading when clusters vary widely in size. Instead, ARI provides a single, interpretable summary of overall clustering concordance. Finally, we benchmarked our MRF-IMD–based clusters against four alternative strategies (PAM on 30

SPLS components, IntNMF on the full feature set, and PAM on RNA-seq or ATAC-seq alone) and now report all ARI values side-by-side for transparency. In the manuscript, we did the following revision:

Revision

In Results, under "TCGA PAN Cancer Clustering Analysis", revised the clustering analysis into a subsection "Pan-Cancer Clustering" and added quantitative evaluation

Pan-Cancer Clustering We next applied IntNMF directly to the MRF-IMD feature set and determined an optimal rank of eight clusters using `nmf.opt.k` function from the IntNMF R package. Figure 4d shows the resulting confusion matrix, illustrating how our selected features effectively separated the samples into eight clusters. Each cluster highlighted unique molecular characteristics and captured established patterns of tumor heterogeneity, ranging from a combined basal-like breast and uterine carcinoma cluster (Group 1) through gastrointestinal adenocarcinomas (Group 5) and hepatobiliary tumors (Group 2) to hypermutated immunogenic cancers (Group 3), squamous-cell carcinomas (Group 7), endocrine neoplasms (Group 6), and renal epithelial tumors (Group 8). A detailed description of the these clusters is in Table 5. To further quantify the advantage conferred by MRF-IMD feature selection, we applied IntNMF clustering to the pan-cancer dataset, aiming for 20 clusters. We first excluded cancer types with fewer than five samples (TCGA-CESC and TCGA-CHOL), as very small cohorts can produce unstable clusters driven by outliers or noise. We then quantified cluster recovery by computing the adjusted rand index (ARI) between the 20 IntNMF clusters on MRF-IMD features and the true TCGA tumor-type labels Supplementary Figure 4a), benchmarking against four alternative strategies: PAM clustering on 30 SPLS components (Supplementary Figure 4b); IntNMF on the full feature set (Supplementary Figure 4c); PAM clustering on RNA-seq data alone (Supplementary Figure 4d); and PAM clustering on ATAC-seq data alone (Supplementary Figure 4e). Although all approaches are shown moderate to high performance, our method still shows advantage with slightly higher ARI of 0.728. Nearly every TCGA cohort is assigned to a single dominant cluster: COAD (36 out of 37 total samples in group 16), KIRP (31 out of 32 in group 7), PRAD (25 out of 26 in group 15), and UCEC (12 out of 12 in group 11) all show almost perfect one-to-one mapping. In breast cancer, the algorithm distinguished three biologically meaningful subgroups: Group 13 captured a pure basal-like subtype (all 13 basal tumors); Group 5 combined all HER2 (9 out of 10) and LumB (16 out of 16) cases with most LumA (18 out of 29) samples, reflecting a non-basal, high-risk profile; and Group 1 comprised predominantly LumA tumors (9/11 total samples) alongside normal controls, defining a lower-risk, luminal-A–driven cluster. Smaller lineages such as BLCA, ESCA, and STAD likewise concentrate into their own clusters with minimal leakage. By contrast, the other methods produced more fragmented assignments and lower ARI scores, sPLS+PAM (ARI = 0.697), full-feature IntNMF (0.687), RNA-seq only (0.713), and ATAC-seq only (0.675), underscoring MRF-IMD's superior ability to isolate coherent, biologically relevant tumor groups.

8. It is advised to re-find the Discussion. In what scenario their new method can be applied? What biological insights can be obtained and what can be missed by the new method?

We thank the reviewer for the suggestion to strengthen our Discussion. In response, we have fully revised the section to (i) illustrate application scenarios for MRF-IMD, (ii) refine the biological insights gleaned from our TCGA and ADNI analyses, and (iii) expand on the limitations and future extensions of our approach. All changes are highlighted in red in Discussion section of the revised manuscript. In particular:

Revision

(1) In Discussion, added suggested scenarios that our method can be applied

In summary, our method is particularly well suited for analyzing complex, cross-layer, and high-dimensional datasets, such as those encountered in cancer and neurodegenerative disorders. It also stabilizes "small-n, large-p" analyses, common in rare disease studies or clinical trials, by reducing the feature space to the most reproducible cross-omics signals. MRF-IMD is capable of uncovering biomarkers and regulatory patterns that may be missed by traditional linear methods, especially when nonlinear patterns or interactions are present. To ensure computational tractability and model convergence, we recommend a preliminary variable-filtering step, such as retaining the most variable features per layer (e.g. under 5,000 features). In downstream tasks like molecular subtyping and survival prediction, MRF-IMD produces compact, interpretable biomarker panels that achieve more robust patient stratification than single-omics approaches, as demonstrated across the TCGA-BRCA, TCGA-COAD, pan-cancer, and ADNI cohorts.

(1) In Discussion, added biological insights obtained from the real data analysis

We further demonstrated the framework's utility using multi-omics data from The Cancer Genome Atlas (TCGA). In breast and colorectal cancer, our approach uncovered known cancer-related genes, miRNAs, and DNA methylation features, as well as biologically relevant pathways. In breast cancer, our framework identified top gene candidates such as *FOXC1*, *PRR15*, and *PRKCQ*, along with key epigenetic (e.g. probes in *BCL9* and *SFT2D2*, associated with breast cancer prognosis) and miRNA features such as miR-584-5p and let-7c-5p that have documented roles in breast tumor progression. In colorectal cancer, the MRF-IMD-selected features likewise reflected key disease mechanisms. Top genes included *SFRP4* and *ANGPTL1*, which are both implicated in colorectal tumor aggressiveness. Our method also picked up a methylation site in the *PRAC2* locus (cg23960088), a region noted as a pan-cancer methylation biomarker in gastrointestinal and prostate tumors, indicating that the integrative approach can recover known epigenetic aberrations. These findings are well-aligned with known tumor biology. In addition, an advantage of our integrative approach is evident in the pathway analysis of selected genes. In TCGA-BRCA, the MRF-IMD gene panel was highly enriched for signaling cascades central to breast cancer. For example, *PI3K/AKT Signaling* appeared as a top canonical pathway, along with several ERBB4/HER-family routes. This is consistent with the pivotal role of the PI3K–AKT–mTOR axis in driving ER-positive breast tumors and endocrine resistance[45] (Figure 3b; Supplementary Table 1). In TCGA-COAD, the MRF-IMD-derived gene list showed strong en[46]richment for pathways related to the tumor microenvironment and EMT. Top hits

included *Extracellular Matrix Organization* and *Proteoglycan Remodeling* pathways, which align with the known importance of stromal reprogramming in colorectal cancer progression[46] (Figure 3b; Supplementary Table 1). It is worth emphasizing that many of these pathways were uniquely identified by MRF-IMD – linear integrative methods, CCA/SPLS, when given a comparable number of features, often failed to enrich for these key pathways (Figure 4c). Moreover, clustering patients based on selected variables revealed groups with distinct survival outcomes, underscoring the clinical relevance of our discoveries. Using the selected multi-omic features for patient stratification yielded significant prognostic separations in both BRCA and COAD cohorts. In a pan-cancer setting, we showed that the MRF-IMD method could detect key molecular differences among diverse tumor types, identifying clusters with characteristic genomic instabilities, pathway alterations, and tissue-of-origin patterns. For instance, basal-like breast cancers clustered tightly with uterine serous carcinomas (Group 1), reflecting their shared genomic profile of TP53 mutations and chromosomal instability[47]. Similarly, a cluster comprising colorectal, gastric, and esophageal adenocarcinomas emerged (Group 5), consistent with the known CIN (chromosomal instability) phenotype common to gastrointestinal epithelial cancers. Other clusters aligned with established categories: we observed a grouping of squamous cell carcinomas across different organs (esophagus, lung, head/neck; Group 7) characterized by TP53 mutations and RTK/RAS pathway activation, and a clear cluster of renal cell tumors (Group 8) distinguished by their unique metabolic and microenvironment profiles. These results suggest that the approach has broad applicability, enhancing our understanding of tumor heterogeneity and potential therapeutic targets. Furthermore, clustering on MRF-IMD features achieved a high adjusted Rand index (ARI) relative to true tumor types, outperforming clustering based on all features or features from linear dimension reduction. In practical terms, our integrative selection could potentially facilitate tumor classification and subtyping in large heterogeneous datasets, focusing attention on the most informative genomic and epigenomic markers while filtering out noisy, uninformative variables.

In the ADNI cohort, MRF-IMD again proved its merit by uncovering biologically plausible and prognostically relevant markers of cognitive decline. Unlike cancer, where tissue-specific pathways dominate, Alzheimer's disease involves complex systemic and brain processes. Our method highlighted genes such as *ARL11*, *S1PR1*, *CCR7*, and *DAPK2* among the top candidates. These findings illustrate how multi-omics integration can spotlight targets that are not the most significant in any single data source but are critical when considering disease pathways together. Moreover, our integrative approach improved the prediction of clinical outcomes in the ADNI cohort. We compared our multi-omics signature to a published methylation risk score (MRS) based on 151 CpGs from a large epigenome meta-analysis[24]. Whereas the MRS alone did stratify patients to some degree, the MRF-IMD integrative signature achieved a more significant separation between progressors and non-progressors (by Kaplan–Meier analysis). In fact, when we incorporated the integrative component into a Cox regression (mirroring the original study's covariate-adjusted model), it yielded a stronger association with time-to-dementia than the MRS. This suggests that combining gene expression with methylation (guided by MRF-IMD to focus on the most relevant features) captures a more predictive composite biomarker of cognitive decline.

(3) In **Discussion**, added limitation and future direction (see Revision (2) in Comment 2)

9. The authors did not provide sufficient details about the datasets they used in the section Method. How many samples in TCGA? How many features did they use? How many features left after filtering?

We thank the reviewer for this suggestion. To improve clarity, we have updated Table 2 to report, for each cohort, the total sample size, the original number of features per omics layer, and the number of features retained after filtering.

Table 2: Summary of datasets

Dataset	Number of arrays	Number of arrays for training	Number of samples	Number of arrays selected by MRF-IMD
TCGA-	*mRNA, DNAm, miRNA*			
BRCA	20530, 485577, 2238	2000, 2000, 228	674	102, 141, 22
COAD	20530, 485577, 2113	2000, 2000, 252	257	139, 107, 18
TCGA-	*ATAC-Seq, RNA-Seq*			
Pan-Cancer	562709, 59390	50000, 5000	383	186, 300
			TCGA-ACC: 9; TCGA-BLCA: 9; TCGA-BRCA: 72; TCGA-CESC: 2; TCGA-CHOL: 2; TCGA-COAD: 37; TCGA-ESCA: 18; TCGA-HNSC: 9; TCGA-KIRC: 16; TCGA-KIRP: 32; TCGA-LGG: 12; TCGA-LIHC: 17; TCGA-LUAD: 21; TCGA-LUSC: 16; TCGA-MESO: 7; TCGA-PCPG: 9; TCGA-PRAD: 26; TCGA-SKCM: 13; TCGA-STAD: 21; TCGA-TGCT: 9; TCGA-THCA: 14; TCGA-UCEC: 12.	
ADNI	*Gene expression, DNAm*			
	49395, 734743	2000, 2000	Total: 468<br>CN: 198, MCI: 288	161, 54

10. Although the performance of the new approach showed some kind of superior in comparison with other methods, the authors only used the currently known databases. It is advised to apply their approach to additional testing datasets or real-world datasets to increase the confidence of the conclusion of this study. It is also observed that the performance of sPLS is better than others in some cases (Figure 4).

We thank the reviewer for this thoughtful comment. As noted, part of this suggestion overlaps with Reviewer #1's comment 3 on benchmarking against other methods, to which we have responded by expanding our comparisons in both simulation and real-data analyses. Specifically, we added

functional enrichment and prognosis-based evaluations in TCGA-BRCA and TCGA-COAD, as well as clustering-based benchmarking using pan-cancer TCGA data, comparing our method directly to SPLS, PMDCCA, and SGCCA. These additional analyses are detailed in the revised Results section and further support the robustness and biological relevance of MRF-IMD–selected features.

Regarding the performance of SPLS, we agree with the reviewer's observation. We acknowledge that in the latent-factor simulation model, designed around linear relationships and sparsity assumptions, SPLS performs very well. This is expected, as the data-generating process closely aligns with the core assumptions of SPLS. However, our goal was to demonstrate that even under such favorable conditions for SPLS, our MRF-IMD framework remains competitive. More importantly, under complex nonlinear and interaction-driven scenarios (e.g., the three-way nonlinear model in our simulations), MRF-IMD consistently outperforms SPLS and other methods, highlighting its broader applicability.

11. It is suggested to re-fine the figures. The labels and legends are too tiny to be seen.

We thank the reviewer for highlighting the need to improve our figure. In response, we have reorganized related panels into cohesive composite figures, which streamlines visual comparisons and reduces redundancy. We have also increased all font sizes and corrected the sub-figure labels to enhance the clarity.

12. There is no sub-figure labels a,b,c,d,e,f in Figure 8. The positions of sub-figure labels in Figure 3, Figure 4, Figure 5, Figure 7 are not correct.

We thank the reviewer for highlighting the need to improve our figure. In response, we have reorganized related panels into cohesive composite figures, which streamlines visual comparisons and reduces redundancy. We have also increased all font sizes and corrected the sub-figure labels to enhance the clarity.

Reviewer #3

Summary:

This manuscript presents a novel multivariate random forest (MRF)-based framework, incorporating the Inverse Minimal Depth (IMD) metric, for integrative multi-omics variable selection and robust biomarker discovery. The method is thoughtfully developed, rigorously evaluated through comprehensive simulations, and effectively demonstrated on TCGA datasets. The topic is highly relevant, and the manuscript is generally well-organized and clearly written.

Thank you for your positive evaluation and supportive remarks. We are glad that you find the MRF-IMD framework novel, rigorous, and clearly presented. In response to your feedback, we have made targeted clarifications and edits throughout the manuscript—each change is highlighted in red to facilitate review. We believe these refinements further strengthen the organization, readability, and impact of our work.

Major comments:

The proposed MRF-IMD framework demonstrates significant advantages in handling nonlinear relationships and high-dimensional data integration. However, a more comprehensive comparison with other nonlinear ensemble methods (e.g., gradient boosting or deep learning approaches) is recommended to highlight its uniqueness.

We thank the reviewer for this valuable suggestion. To better position our MRF-IMD framework among nonlinear ensemble methods, we expanded our simulation study to include comparisons with univariate random forests, gradient boosting machines (GBM), and XGBoost. Since these methods do not natively support multi-response integration, we adapted them for our ranking-based evaluation by fitting a separate model for each response variable, computing per-feature importance scores, and averaging those scores across responses to produce a single global ranking. This allowed us to assess their ability to detect cross-layer signals in an unsupervised setting. As shown in the revised Results section (Supplementary Fig[57]ure 2), MRF-IMD consistently outperformed all three methods, particularly in the "three-way nonlinear interaction" simulation where high-order effects are difficult to capture.

We chose not to include deep learning–based integrative frameworks such as MOGONET or variational autoencoders in this benchmarking, which we acknowledge as a limitation of our current study. These approaches are typically supervised, require substantial labeled training data, and rely on outcome-dependent loss functions, making them fundamentally incompatible with our unsupervised, cross-omics variable selection goals. Moreover, most deep learning models lack transparent or interpretable feature importance mechanisms, and adapting them in the comparison described in our manuscript would require substantial architectural changes and additional assumptions. We have included this discussion in the revised manuscript's limitations section, and we agree that future work comparing our framework with unsupervised or semi-supervised deep

learning methods could further enrich the benchmarking and broaden the understanding of integrative strategies.

In particular, we did the following revision:

Revision

(1) In Material and Method, under "Simulation Study", added ranking-based simulation

To benchmark our IMD-based selection against nonlinear ensemble learners, we also included RF permutation importance measurement, gradient boosting machines (GBM) and XGBoost. Because these algorithms lack native support for multivariate or unsupervised multi-omics variable selection, we adapted them by fitting a separate univariate model for each response, computing per-feature importance scores, and then averaging those scores across responses to produce a single global ranking. We applied this procedure to three simulation frameworks, (i) a latent-factor model, (ii) a nonlinear regression model, and (iii) an interaction model in which the outcome depends on pairwise predictor interactions, each time generating paired two-omics data where one layer contained only true signal variables (as the outcome) and the other served as predictors. In every scenario, we compared the rankings produced by MRF-IMD to those from three integration methods (SPLS, PMDCCA, SGCCA) and the three ensemble learners, evaluating each method's ability to elevate known signal features via PR-AUC, true-positive rate in the top k, and ranking stability across replicates.

(2) In Results, under "Evaluation of Simulated Data", added subsection "Ranking-based Comparison"

Ranking-based Comparison In ranking-based simulations across three models, latent, three-way interaction, and nonlinear-regression models, our MRF-IMD methods consistently achieved competitive PR-AUC results at the top k predictors. The boxplot from (Supplementary Figure 2) shows that the IMD scores achieve the stable results across all scenarios. Extended simulation results are provided in Supplementary Note 7.

(3) In Discussion, added limitation (pp. 23-24)

… Fourth, we acknowledge that we did not benchmark MRF-IMD against deep learning–based integrative frameworks—a limitation of our current evaluation. Deep neural architectures (e.g., graph convolutional networks or variational autoencoders) have shown promise for multi-omics integration, yet they are typically supervised, rely on large labeled datasets, and lack transparent, unsupervised feature-importance mechanisms. Consequently, they cannot be directly applied to our unsupervised, multi-response variable-selection setting without substantial adaptation. Future work comparing our framework with unsupervised or semi-supervised deep learning methods could further enrich the benchmarking and broaden the understanding of integrative strategies…

Reference

1. Guo F, Ma J, Li C, et al. PRR15 deficiency facilitates malignant progression by mediating PI3K/Akt signaling and predicts clinical prognosis in triple-negative rather than non-triple-negative breast cancer. *Cell Death Dis*. 2023;14(4):272. doi:10.1038/s41419-023-05746-8

2. Byerly JH, Port ER, Irie HY. PRKCQ inhibition enhances chemosensitivity of triple-negative breast cancer by regulating Bim. *Breast Cancer Research*. 2020;22(1):72. doi:10.1186/s13058-020-01302-w

3. Molière S, Lodi M, Leblanc S, et al. MMP-11 expression in early luminal breast cancer: associations with clinical, MRI, pathological characteristics, and disease-free survival. *BMC Cancer*. 2024;24(1):295. doi:10.1186/s12885-024-11998-0

4. Zhuang Y, Li X, Zhan P, Pi G, Wen G. MMP11 promotes the proliferation and progression of breast cancer through stabilizing Smad2 protein. *Oncol Rep*. 2021;45(4):16. doi:10.3892/or.2021.7967

5. Khaled WT, Choon Lee S, Stingl J, et al. BCL11A is a triple-negative breast cancer gene with critical functions in stem and progenitor cells. *Nat Commun*. 2015;6(1):5987. doi:10.1038/ncomms6987

6. Martin EM, Orlando KA, Yokobori K, Wade PA. The estrogen receptor/GATA3/FOXA1 transcriptional network: lessons learned from breast cancer. *Curr Opin Struct Biol*. 2021;71:65-70. doi:10.1016/j.sbi.2021.05.015

7. Xu J, Xiang L, Liu Q, et al. Stacked Sparse Autoencoder (SSAE) for Nuclei Detection on Breast Cancer Histopathology Images. *IEEE Transactions on Medical Imaging*. 2016;35(1):119-130. doi:10.1109/TMI.2015.2458702

8. Denkiewicz M, Saha I, Rakshit S, Sarkar JP, Plewczynski D. Identification of Breast Cancer Subtype Specific MicroRNAs Using Survival Analysis to Find Their Role in Transcriptomic Regulation. *Front Genet*. 2019;10. doi:10.3389/fgene.2019.01047

9. Qattan A, Intabli H, Alkhayal W, Eltabache C, Tweigieri T, Amer SB. Robust expression of tumor suppressor miRNA’s let-7 and miR-195 detected in plasma of Saudi female breast cancer patients. *BMC Cancer*. 2017;17(1):799. doi:10.1186/s12885-017-3776-5

10. Huang D, Yu B, Deng Y, et al. SFRP4 was overexpressed in colorectal carcinoma. *J Cancer Res Clin Oncol*. 2010;136(3):395-401. doi:10.1007/s00432-009-0669-2

11. Nfonsam LE, Jandova J, Jecius HC, Omesiete PN, Nfonsam VN. SFRP4 expression correlates with epithelial mesenchymal transition-linked genes and poor

overall survival in colon cancer patients. *World J Gastrointest Oncol*. 2019;11(8):589-598. doi:10.4251/wjgo.v11.i8.589

12. Chang TY, Lan KC, Chiu CY, Sheu ML, Liu SH. ANGPTL1 attenuates cancer migration, invasion, and stemness through regulating FOXO3a-mediated SOX2 expression in colorectal cancer. *Clin Sci (Lond)*. 2022;136(9):657-673. doi:10.1042/CS20220043

13. Koestler DC, Li J, Baron JA, et al. Distinct patterns of DNA methylation in conventional adenomas involving the right and left colon. *Mod Pathol*. 2014;27(1):145-155. doi:10.1038/modpathol.2013.104

14. Hu W, Yang Y, Li X, et al. Multi-omics Approach Reveals Distinct Differences in Left- and Right-Sided Colon Cancer. *Molecular Cancer Research*. 2018;16(3):476-485. doi:10.1158/1541-7786.MCR-17-0483

15. Chen P, Xi Q, Wang Q, Wei P. Downregulation of microRNA-100 correlates with tumor progression and poor prognosis in colorectal cancer. *Med Oncol*. 2014;31(10):235. doi:10.1007/s12032-014-0235-x

16. Hibner G, Kimsa-Furdzik M, Francuz T. Relevance of MicroRNAs as Potential Diagnostic and Prognostic Markers in Colorectal Cancer. *International Journal of Molecular Sciences*. 2018;19(10):2944. doi:10.3390/ijms19102944

17. Liberzon A, Subramanian A, Pinchback R, Thorvaldsdóttir H, Tamayo P, Mesirov JP. Molecular signatures database (MSigDB) 3.0. *Bioinformatics*. 2011;27(12):1739-1740. doi:10.1093/bioinformatics/btr260

18. Paplomata E, O’Regan R. The PI3K/AKT/mTOR pathway in breast cancer: targets, trials and biomarkers. *Ther Adv Med Oncol*. 2014;6(4):154-166. doi:10.1177/1758834014530023

19. Wiseman BS, Werb Z. Stromal Effects on Mammary Gland Development and Breast Cancer. *Science*. 2002;296(5570):1046-1049. doi:10.1126/science.1067431

20. Dongre A, Weinberg RA. New insights into the mechanisms of epithelial–mesenchymal transition and implications for cancer. *Nat Rev Mol Cell Biol*. 2019;20(2):69-84. doi:10.1038/s41580-018-0080-4

21. Nenkov M, Ma Y, Gaßler N, Chen Y. Metabolic Reprogramming of Colorectal Cancer Cells and the Microenvironment: Implication for Therapy. *Int J Mol Sci*. 2021;22(12):6262. doi:10.3390/ijms22126262

22. Ma T, Guo L, Yan H, Wang L. Cobind: quantitative analysis of the genomic overlaps. *Bioinformatics Advances*. 2023;3(1):vbad104. doi:10.1093/bioadv/vbad104

23. Chalise P, Fridley BL. Integrative clustering of multi-level ’omic data based on non-negative matrix factorization algorithm. *PLoS One*. 2017;12(5):e0176278. doi:10.1371/journal.pone.0176278

24. Zhang W, Young JI, Gomez L, et al. Blood DNA methylation signature for incident dementia: Evidence from longitudinal cohorts. *Alzheimer’s & Dementia*. 2025;21(3):e14496. doi:10.1002/alz.14496

25. Choi SB, Kwon S, Kim JH, Ahn NH, Lee JH, Yang SH. The Molecular Mechanisms of Neuroinflammation in Alzheimer’s Disease, the Consequence of Neural Cell Death. *Int J Mol Sci*. 2023;24(14):11757. doi:10.3390/ijms241411757

26. Zhu Z, Zhang L, Elsherbini A, et al. The S1P receptor 1 antagonist Ponesimod reduces TLR4-induced neuroinflammation and increases Aβ clearance in 5XFAD mice. *eBioMedicine*. 2023;94. doi:10.1016/j.ebiom.2023.104713

27. Zhang T, Xia Y, Hu L, et al. Death-associated protein kinase 1 mediates Aβ42 aggregation-induced neuronal apoptosis and tau dysregulation in Alzheimer’s disease. *Int J Biol Sci*. 2022;18(2):693-706. doi:10.7150/ijbs.66760

28. Da Mesquita S, Herz J, Wall M, et al. Aging-associated deficit in CCR7 is linked to worsened glymphatic function, cognition, neuroinflammation, and β-amyloid pathology. *Sci Adv*. 2021;7(21):eabe4601. doi:10.1126/sciadv.abe4601

29. Dai L, Shen Y. Insights into T-cell dysfunction in Alzheimer’s disease. *Aging Cell*. 2021;20(12):e13511. doi:10.1111/acel.13511

30. Fehervari Z. Lymphocytes in Alzheimer’s disease. *Nat Immunol*. 2016;17(4):355-355. doi:10.1038/ni.3427

31. Subramanian I, Verma S, Kumar S, Jere A, Anamika K. Multi-omics Data Integration, Interpretation, and Its Application. *Bioinform Biol Insights*. 2020;14:1177932219899051. doi:10.1177/1177932219899051

32. Gutierrez Reyes CD, Alejo-Jacuinde G, Perez Sanchez B, et al. Multi Omics Applications in Biological Systems. *Curr Issues Mol Biol*. 2024;46(6):5777-5793. doi:10.3390/cimb46060345

33. Hasin Y, Seldin M, Lusis A. Multi-omics approaches to disease. *Genome Biology*. 2017;18(1):83. doi:10.1186/s13059-017-1215-1

34. Singh A, Shannon CP, Gautier B, et al. DIABLO: an integrative approach for identifying key molecular drivers from multi-omics assays. *Bioinformatics*. 2019;35(17):3055-3062. doi:10.1093/bioinformatics/bty1054

35. Wang T, Shao W, Huang Z, et al. MOGONET integrates multi-omics data using graph convolutional networks allowing patient classification and biomarker identification. *Nat Commun*. 2021;12(1):3445. doi:10.1038/s41467-021-23774-w

36. Xiao L, Zhang F, Zhao F. Large-scale microbiome data integration enables robust biomarker identification. *Nat Comput Sci*. 2022;2(5):307-316. doi:10.1038/s43588-022-00247-8

37. Coletti R, Lopes MB. Multi-omics Data Integration and Network Inference for Biomarker Discovery in Glioma. In: Moniz N, Vale Z, Cascalho J, Silva C, Sebastião R, eds. *Progress in Artificial Intelligence*. Springer Nature Switzerland; 2023:247-259. doi:10.1007/978-3-031-49011-8_20

38. Wold H. Estimation of principal components and related models by iterative least squares. Published online 1966. Accessed January 22, 2024. https://www.acemap.info/paper/64195297

39. Chun H, Keleş S. Sparse partial least squares regression for simultaneous dimension reduction and variable selection. *Journal of the Royal Statistical Society: Series B (Statistical Methodology)*. 2010;72(1):3-25. doi:10.1111/j.1467-9868.2009.00723.x

40. Hotelling H. Relations Between Two Sets of Variates. *Biometrika*. 1936;28(3/4):321-377. doi:10.2307/2333955

41. Witten DM, Tibshirani R, Hastie T. A penalized matrix decomposition, with applications to sparse principal components and canonical correlation analysis. *Biostatistics*. 2009;10(3):515-534. doi:10.1093/biostatistics/kxp008

42. Tenenhaus A, Tenenhaus M. Regularized Generalized Canonical Correlation Analysis. *Psychometrika*. 2011;76(2):257-284. doi:10.1007/s11336-011-9206-8

43. Lai PL, Fyfe C. Kernel and nonlinear canonical correlation analysis. *Int J Neur Syst*. 2000;10(05):365-377. doi:10.1142/S012906570000034X

44. Yoshida K, Yoshimoto J, Doya K. Sparse kernel canonical correlation analysis for discovery of nonlinear interactions in high-dimensional data. *BMC Bioinformatics*. 2017;18(1):108. doi:10.1186/s12859-017-1543-x

45. Gil EMC. Targeting the PI3K/AKT/mTOR pathway in estrogen receptor-positive breast cancer. *Cancer Treatment Reviews*. 2014;40(7):862-871. doi:10.1016/j.ctrv.2014.03.004

46. Lu J, Kornmann M, Traub B. Role of Epithelial to Mesenchymal Transition in Colorectal Cancer. *Int J Mol Sci*. 2023;24(19):14815. doi:10.3390/ijms241914815

47. Levine DA. Integrated genomic characterization of endometrial carcinoma. *Nature*. 2013;497(7447):67-73. doi:10.1038/nature12113

Manuscript
Click here to access/download;Manuscript;main_082525_clean.docx

An Integrative Multi-Omics Random Forest Framework for Robust Biomarker Discovery

Wei Zhang[1*], Hanchen Huang[1], Lily Wang[1,2,3,4], Brian D. Lehmann[5], Steven X. Chen[1,2,*]

[1] Division of Biostatistics and Bioinformatics, Department of Public Health Sciences, University of Miami, Miller School of Medicine, Miami, FL 33136, USA
[2] Sylvester Comprehensive Cancer Center, University of Miami, Miller School of Medicine, Miami, FL 33136, USA
[3] Dr. John T Macdonald Foundation Department of Human Genetics, University of Miami, Miller School of Medicine, Miami, FL, 33136, USA
[4] John P. Hussman Institute for Human Genomics, University of Miami Miller School of Medicine, Miami, FL, 33136, USA
[5] Division of Hematology and Oncology, Department of Medicine, Vanderbilt University Medical Center, Nashville, TN 37232, USA

* To whom correspondence should be addressed. Email: steven.chen@miami.edu. Correspondence may also be addressed to Wei Zhang. Email: wei.zhang60@med.miami.edu.

ABSTRACT

High-throughput technologies now produce a wide array of omics data, from genomic and transcriptomic profiles to epigenomic and proteomic measurements. Integrating multiple omics layers measured on the same samples can reveal cross-layer molecular hubs that single-layer analyses miss. We present an unsupervised, multivariate random forest (MRF) framework with an inverse minimal depth (IMD) importance to prioritize shared biomarkers across omics. In each forest, one layer serves as a multivariate response and the other as predictors; IMD summarizes how early a predictor (or response MSRV) appears across trees, yielding interpretable, cross-layer feature rankings. We provide three IMD-based selection strategies and introduce an optional IMD power transform to enhance sensitivity to interaction signals. In extensive simulations spanning linear, nonlinear, and interaction regimes, our method matches SPLS/CCA under linear settings and outperforms them as nonlinearity increases, while adapted univariate ensemble learners (RF, GBM, XGBoost) underperform in the multivariate, unsupervised context. Applied to TCGA BRCA and COAD, MRF-IMD identifies genes, CpGs, and miRNAs enriched for cancer-relevant pathways and yields more robust survival stratification than linear integrators with matched model sizes. In a TCGA pan-cancer analysis, MRF-IMD features achieve higher ARI than alternatives and recover coherent tumor-type clusters; in ADNI, the integrative signature improves dementia-progression stratification over a published methylation risk score. Our scalable, interpretable MRF-IMD framework advances reliable multi-omics biomarker discovery when nonlinear, cross-layer dependencies matter..

INTRODUCTION

Recent technological advances in high-throughput sequencing, mass spectrometry, and imaging have led to a surge in multi-omics data that span the genome, epigenome, transcriptome, proteome, and metabolome. However, each type of data alone captures only a slice of disease biology. Integrating these diverse data sources can provide a more comprehensive picture of complex biological systems than analyzing any single omics layer alone. Multi-omics analysis has been implemented in many studies for biomarker discovery, disease subtyping, and disease insights[1]. A key goal in multi-omics integration is to

extract "shared" biomarkers from multiple data – that is, to identify molecular features that are biologically relevant consistently across different omics type. These biomarkers typically indicate robust, system-level regulatory mechanisms that single-omics analyses may miss[1,2]. Furthermore, integrating complementary data sources reduces noise and mitigates biological heterogeneity, enhancing the precision and clinical relevance of patient stratification and prognosis[3]. In general, multi-omics approaches tend to yield more reliable biomarkers and disease signatures than single-modality analyses, as demonstrated in recent studies: methods like DIABLO, which based on the sparse partial least squares (SPLS) method, seek common information across data types by selecting subsets of features that jointly capture variance in each dataset[4]. When done effectively, integration can highlight shared molecular features across different data types, offering new insights into disease mechanisms, patient stratification, and potential biomarkers for clinical applications[4–7].

Despite the promise of multi-omics integration, extracting shared signals across heterogeneous datasets remains challenging. Traditional penalized integration methods, such as SPLS[4,8,9] and canonical correlation analysis (CCA) [10–12], focus largely on linear relationships. Although widely used, these approaches can struggle in high-dimensional settings, are prone to overfitting, and may fail to capture nonlinear interactions. For example, in "XOR" simulation where two variables interact multiplicatively to determine the response, both SPLS or CCA, which optimize linear covariance/correlation, tend to yield near-random feature rankings and fail to recover the interacting pair (as we also show in our simulations). Nonlinear extensions, including kernel CCA[13,14], help address some of these issues but often face scalability and interpretability limitations, making them less suitable for many practical scenarios.

Ensemble learning techniques, particularly random forests, are valued for their robustness, ability to model nonlinearities, and relative resilience to overfitting[15]. Extending random forests to handle multiple response variables leads to multivariate random forests (MRF)[16], which are well-positioned to tackle complex multi-omics data. However, applications of MRF to multi-omics integration have been limited, leaving an opportunity to develop methods that exploit the strengths of this approach for biomarker discovery and feature selection.

In this study, we introduce a new MRF-based framework that employs the inverse minimal depth (IMD) metric for unsupervised variable selection across multiple omics datasets. We model the relationships between each pair of two omics by assigning one omics to the response space in and the other omics to the feature space in an ensemble of decision trees. After fitting the forest, we compute the IMD to quantify feature importance and identify key variables shared across different data layers. We then extend our framework from pairwise (two-omics) integration to a comprehensive multi-omics approach by modeling different layer pairs guided by prior knowledge or precomputed inter-relationships. This strategy naturally reduces the risk of selecting noise variables and helps focus on those with consistent impact across datasets. To show that our method can effectively capture shared biomarkers in complex datasets, we benchmarked it against established integration approaches, including SPLS, CCA, and several nonlinear ensemble methods such as gradient boosting machine (GBM) and XGBoost through multiple simulations.

We found that methods like SPLS and CCA are not stable in capturing the important features when data types are in nonlinear or contains interaction settings. Other nonlinear methods are easily failed as they are not designed for multivariate analysis. Moreover, we validated our framework using several clinical cohorts, including TCGA-BRCA and TCGA-COAD, demonstrating superior ability to uncover biologically relevant pathways and to stratify patients by prognostic outcome compared to traditional integration methods such as SPLS and CCA. We further applied our approach to the TCGA-PANCAN and ADNI datasets, identifying biomarker panels tied to key biological pathways that show promise for enhancing molecular subtyping of PAN cancer, and prognosis of dementia onset.

In summary, our MRF-IMD framework provides a robust and flexible solution for multi-omics integration. By embracing nonlinear relationships, addressing high-dimensionality, and maintaining interpretability, this approach has the potential to advance biomarker discovery and contribute valuable insights to complex biological and clinical problems.

MATERIAL AND METHODS

Maximal Splitting Response Variable

Consider two datasets $\mathbf{X}_{n\times p}$ and $\mathbf{Y}_{n\times q}$ where n is the number of samples and p and q represent the number of features of $\boldsymbol{X}$ and $\boldsymbol{Y}$ respectively. Our goal is to integrate these datasets using a multivariate random forest (MRF) approach. In this framework, we use a splitting rule that considers all response variables together, rather than handling them individually. We begin with a splitting rule introduced by Tang and Ishwaran that extends the traditional univariate splitting criterion to a multivariate setting[17]. This rule extends univariate splitting by summing the splitting criterion across all response outcomes $Y_{.j}$. The splitting criterion for node t is defined as:

$$G_q(s,t) = \sum_{j=1}^{q} \left\{ \sum_{i\in t_L} \left(Y_{ij} - \bar{Y}_{t_{L_j}}\right)^2 + \sum_{i\in t_R} \left(Y_{ij} - \bar{Y}_{t_{R_j}}\right)^2 \right\} = \sum_{j=1}^{q} G_j\,(s,t) \tag{1}$$

where t_{L_j} and t_{R_j} represents the left and right daughter nodes for j_{th} response coordinate and $\bar{Y}_{t_{L_j}}$ and $\bar{Y}_{t_{R_j}}$ are the sample means in t_{L_j} and t_{R_j}. To determine the best split, we minimize $G_q(s,t)$, ensuring all response

variables $Y_{.1}, \dots, Y_{.q}$ are measured on the same scale by standardizing them to a 0-1 scale: $Y_{ij}^* = \frac{\sqrt{n}\left(Y_{ij}-\bar{Y}_{t_j}\right)}{\sqrt{\sum_{i\in t}\left(Y_{ij}-\bar{Y}_{t_j}\right)^2}}$, where

$$\frac{1}{n}\sum_{i\in t} Y_{ij}^* = 0, \quad \frac{1}{n}\sum_{i\in t} Y_{ij}^{*2} = 1, \quad \text{for } 1 \le j \le q$$

This standardization ensures that the contributions of all outcomes are comparable, preventing any single outcome from dominating the splitting process. After simplifying the expression, the minimization of $G_q(s,t)$ becomes equivalent to maximizing:

$$G_q^*(s,t) = \sum_{j=1}^{q} \left\{ \frac{1}{n_{t_L}} \left(\sum_{i\in t_L} Y_{ij}^* \right)^2 + \frac{1}{n_{t_R}} \left(\sum_{i\in t_R} Y_{ij}^* \right)^2 \right\}$$

(2)

In the case of two-omics data, we treat one dataset as the response and the other as the predictor, and apply the multivariate random forest (MRF) model using these splitting rules.

Using the above framework, we now focus on two-omics data. For each response variable Y_j, we define the splitting statistic as:

$$G_j = \frac{1}{n_{t_L}} \left(\sum_{i\in t_L} Y_{\mathrm{ij}}^* \right)^2 + \frac{1}{n_{t_R}} \left(\sum_{i\in t_R} Y_{\mathrm{ij}}^* \right)^2$$

(3)

This statistic quantifies how well a split separates the values of Y_j in the response $\mathbf{Y}$ across the left and right daughter nodes. For each node split, we identify the maximal splitting response variable (MSRV) as the response variable that maximizes the multivariate splitting rule $G_q^*(s,t)$, meaning it has the largest contribution to the split. The MSRV represents the variable most associated with the predictors at that particular node. A detailed explanation and study of MSRV is described in Supplementary Note 1.

Inverse Minimal Depth

Minimal Depth. Minimal depth, introduced by Ishwaran et al.[18,19], is a variable selection method that efficiently ranks strong variables higher than weak ones. The minimal depth of a variable refers to the shortest distance from the root of a decision tree to the node where the variable appears. Let D_v denote the minimal depth of a variable v and $D(T)$ represents the depth of a tree T, it has been proved that the distribution of D_v is :

$$\mathbb{P}\{D_v = d \mid \ell_0^*, \dots, \ell_{D(T)-1}^*\} = \left[\prod_{j=0}^{d-1} \left(1 - \pi_{v,j}\theta_{v,j}\right)^{\ell_j^*}\right] \left[1 - \left(1 - \pi_{v,d}\theta_{v,d}\right)^{\ell_d^*}\right], \quad 0 \le d \le D(T) - 1$$

(4)

where $\pi_{v,j}$ is the probability of v selected as a candidate variable for splitting of node t at depth j, $\theta_{v,j}$ is the probability of v splits a node t at depth j, and ℓ_j^* is the number of nodes at depth j. Note that if the tree is a balanced tree, $\ell_j^* = 2^j$ at depth j. For example, in Figure 1, the root node variable X_{20} is assigned a minimal depth $D_{X_{20}} = 0$. The left daughter node of X_{20}, X_6 has a minimal depth of $D_{X_6} = 1$. In the original paper on minimal depth, proposed two strategies for identifying strong variables. The first strategy uses the mean minimal depth under the null hypothesis that a variable v is a weak variable. Given v is a weak variable, the distribution of the minimal depth of v is

$$\mathbb{P}\{D_v = d \mid v \text{ is a weak variable}\} \approx \left(1 - \frac{1}{p}\right)^{L_d} \left[1 - \left(1 - \frac{1}{p}\right)^{\ell_d}\right]$$

(5)

where $L_d = 1 + 2 + \cdots + 2^{d-1} = \ell_d - 1$ and p is the number of features in the dataset. The threshold works well when n is large and is more computationally efficient than VIMP and jointly VIMP in high-dimensional MRF models. However, when dimensionality is high, meaning $p \gg \ell_{D(T)}$, the threshold will fail because all the probabilities $\mathbb{P}\{D_v = d \mid v \text{ is a weak variable}\}$ will approach 0. Later, we will demonstrate that the threshold fails in high dimensional noise settings with multivariate outcomes. In cases where the mean threshold approach fails, a second strategy known as variable hunting[19]. This approach involves iteratively selecting random subsets of variables, fitting the forest, and combining minimal depth with joint VIMP to prioritize the strongest variables. While effective when the number of features p greatly exceeds the number of samples (i.e. $p \gg n$), this method becomes computationally inefficient and can struggle in high-dimensional noise settings with multivariate outcomes. To overcome these limitations, we propose a variation of the minimal depth approach, which we outline in the next section. This variation allows for the selection of strong variables in both the response and predictor spaces within the multivariate random forest (MRF) model.

Distribution of Inverse Minimal Depth. To incorporate minimal depth into our variable selection method, we introduce a new statistic called inverse minimal depth (IMD), defined as:

$$\mathrm{M_v} = \begin{cases} \dfrac{1}{\mathrm{D_v} + 1} & \text{if } \mathrm{v} \in \mathcal{F} \\ 0 & \text{if } \mathrm{v} \notin \mathcal{F} \end{cases}$$

(6)

where $\mathcal{F}$ is the set of variables selected in the tree. For example, as shown Figure 1, $\mathrm{X_{20}}$ has a minimal depth of $\mathrm{D_{X_{20}}} = 0$ and its IMD is $M_{X_{20}} = 1$. Similarly, $\mathrm{X_6}$ has a minimal depth of $\mathrm{D_{X_6}} = 1$ and its IMD is $M_{X_6} = 1/2$. In this setup, larger IMD values correspond to stronger variables, making it easier to identify them. Additionally, we apply a penalization technique that assigns an IMD of 0 to variables that are not selected

in the tree. When $v \in \mathcal{F}$, the distribution of D_v can be directly transformed from the distribution of D_v as follows:

$$\mathbb{P}\{M_v = m\} = \left[\prod_{j=0}^{1/M-1} \left(1 - \pi_{v,j}\theta_{v,j}\right)^{\ell_j^*}\right]\left[1 - \left(1 - \pi_{v,d}\theta_{v,d}\right)^{\ell_{d^I}^*}\right], \quad 0 < m \leq 1$$

(7)

Note that ℓ_m^* is the same value as in the distribution of minimal depth. With IMD, values are confined between 0 and 1. Variables not selected ($v \notin \mathcal{F}$) have $M_v = 0$, while stronger variables exhibit higher IMD values. The overall IMD for a variable v across the forest is calculated as the average IMD across all trees:

$$M_v = \frac{\sum_{b \in B} M_v^{(b)}}{B}$$

(8)

To evaluate the importance of variables in the response space, we assigned the IMD to the variables selected as the MSRV in each node. This is intuitive because the variables with lower IMD in $\mathbf{X}$ are more likely to be noise variables. As a result, when a decision tree node splits using these noise variables, it is less likely to select stronger, more influential variables from the response set $\mathbf{Y}$ as MSRV. This pattern reflects the structure of decision trees, where stronger variables, having greater predictive power, tend to appear earlier and higher in the tree. To further investigate the relationship between IMD and key MRF parameters—such as the number of trees, tree depth, and the proportion of response variables in each split—we conducted extensive simulations, detailed in Supplementary Note 3. Additionally, to compare the performance of the original MD and the enhanced IMD metric, we performed simulations outlined in Supplementary Note 4.

Variable Selection in High-Dimensional Two-omics Data

As previously noted, one method for variable selection based on minimal depth involves using a pre-defined threshold derived from the distribution of weak variables' minimal depth. However, when $p \gg \ell_{D(T)}$, all the probabilities in (5) approach zero[18]. Applying the same thresholding method to IMD yields similar challenges in high-dimensional datasets, where thresholding values also tend toward zero. To address this, we propose two additional methods for detecting strong variables that are not based on weak variable distributions. First, we introduce these approaches for two-omics data in a multivariate random forest. Then, we extend the selection framework to multi-omics variable selection.

Variable Filtering. As the IMD of noise variables are close to or hovers over 0, we can select the threshold by multiplying a parameter τ to the standard deviation of IMD: $\tau \cdot \sigma_m$. The total number of variables selected can be controlled by varying the τ. To determine the optimal value of τ, we use the mean out-of-bag errors (OOB) of both response and predictor variables for tuning. The mean OOB error is averaged across all the

OOB errors in $\mathbf{X}$ and $\mathbf{Y}$. In the MRF setting, for each $\mathrm{j_{th}}$ response in $\mathbf{Y}_{\mathrm{n}\times\mathrm{q}}$, the loss function becomes $\mathrm{l}(\hat{\mathrm{f}}) = \left(\mathbf{Y}-\hat{\mathrm{f}}(\mathbf{X})\right)^2$. Therefore, the estimation of prediction error for response $\mathrm{Y_j}$ the OOB sample can be defined as:

$$l_j\left(\hat{f},\mathfrak{L}_{\mathrm{n}}^{OOB},X\right)=\frac{1}{|\mathfrak{L}_{\mathrm{n}}^{OOB}|}\sum_{i:(X_i,Y_{ij})\in\mathcal{L}_n^{OOB}}\left(Y_{ij}-\hat{f}(X_i\,\cdot)\right)^2 \tag{9}$$

Similarly, the OOB errors of predictors $\mathbf{X}$ can be derived from the forest weights statistics. The OOB prediction of $\mathbf{X}$ can be formulated as follows:

$$\widehat{f_{\mathrm{OOB}}^{X}}=\sum_{t=1}^{n}\frac{\sum_{b=1}^{B-B_i}\mathbf{1}_{\{n_{b,i}=0\}}\,\mathbf{1}_{\{\mathbf{X_i}\in R_j\}}x_t}{(B-B_i)\sum_{k=1}^{n}\mathbf{1}_{\{n_{b,k}=0\}}\,\mathbf{1}_{\{\mathbf{X_k}\in R_j\}}} \tag{10}$$

$$l_j\left(\widehat{f^X},\mathfrak{L}_{\mathrm{n}}^{OOB},\boldsymbol{X}\right)=\frac{1}{|\mathfrak{L}_{\mathrm{n}}^{OOB}|}\sum_{i:(\boldsymbol{X}_i,X_{ij})\in\mathcal{L}_n^{OOB}}\left(X_{ij}-\widehat{f^X}(X_i\,\cdot)\right)^2 \tag{11}$$

With a step size of 0.1, we computed the mean OOB error using the variables above $\tau\cdot\sigma_{d^{\mathrm{I}}}$. To stabilize the OOB error, we repeated the model fittings k times and averaged the results to select the optimal τ based on a tolerable error deviation.

Detecting Signals with Mixture Model. Given the distribution of differences in IMD between strong variables and noise variables, we can identify strong cross-correlated variables by fitting a two-component mixture model to the forest IMD. We describe the univariate Gaussian mixture model as follows:

$$\mathrm{f}(\mathrm{x};\Theta)=\mathrm{p}\phi(\mathrm{x};\mu_1,\sigma_1^2)+(1-\mathrm{p})\phi(\mathrm{x};\mu_2,\sigma_2^2), \tag{12}$$

where $\phi(\cdot)$ is the normal distribution. As the forest IMD ranges from 0 to 1, we consider modeling the IMD using truncated distribution. A previous study used the truncated normal mixture model to model the intraclass correlation coefficient of DNA methylation probes. The distribution of the truncated normal mixture model is as follows:

$$\mathrm{f}(\mathrm{x};\Theta)=\mathrm{p}\frac{\phi(\mathrm{x};\mu_1,\sigma_1^2)}{1-\Phi(0;\mu_1,\sigma_1^2)}+(1-\mathrm{p})\phi(\mathrm{x};\mu_2,\sigma_2^2), \tag{13}$$

where $p\in[0,1]$ is the proportion of the first component, and the intraclass correlation is bounded by $(0,1)$. Here, we assume that the noise variables have relatively low forest IMD and more likely lie in the first component modeled by the normal or truncated normal distribution. To estimate the parameter of the

mixture model, we used the Expectation-Maximization (EM) algorithm for the model fitting[20,21]. To accommodate the variables with forest IMD = 0, we used the modified log-likelihood function proposed in:

$$\log L(\Theta; x) = n_0 \log(p_0) + \sum_{x \in d^{\mathrm{I}} \neq 0} \log\left(p_1 f_1(x)\right) + \sum_{x \in d^{\mathrm{I}} \neq 0} \log\left(p_2 f_2(x)\right)$$

(14)

where p_0 is the proportion of forest IMD = 0, $p_1 = p(1 - p_0)$, and $p2 = (1 - p)(1 - p_0)$. In the forest IMD, we separated the forest IMD = 0 and modeled the forest IMD > 0 using (12) or (13). Supplementary Figure 1a shows the density of modeling the forest IMD using Gaussian mixture and truncated normal mixture models. The data is simulated by the latent model with the settings of $p = q = 500$, $n = 200$, and the first 30 variables of each dataset are cross-correlated with each other. Here, we can see that the forest IMD has a skewed distribution. The Gaussian mixture model shows a better fit of the forest IMD. For each component, the posterior probabilities can be calculated as:

$$Pr_i(x) = \frac{p_i f_i(x)}{\sum p_j f_j(x)}, \quad j \in 0,1,2$$

(15)

We selected variables with $Pr_1(x) < pr$ as the important variables, where pr is a predefined value (i.e., $pr = 0.05$).

IMD Transformation. In the previous section, we discovered that the mean IMD of strong variables is close to the mean IMD of noise variables in high-dimensional settings. However, the forest IMD of strong or cross-correlated variables may be low and mingled with noise variables. This can be challenging for the mixture model to capture due to the sparsity of strong variables. To address this issue, we propose a third method that explores the distribution of IMD for both noise and strong variables. Using the latent model, we simulated two datasets with the same settings as in the previous section. From Supplementary Figure 1b top panel, it is clear that the forest IMD of noise variables skews heavily toward the lower end of the IMD scale, clustering near zero. In contrast, the cross-correlated variables exhibit a broader distribution starting from zero, although this is less apparent due to their sparsity. This makes distinguishing between noise and strong variables using only a threshold on the original forest IMD difficult. Let μ (displayed as a black dashed line), μ_{Noise} (displayed as a red dashed line), and μ_{Strong} (displayed as a blue dashed line) denote the mean forest IMD of all variables, noise variables, and cross-correlated variables, respectively. It is clear to see that $\mu_{Noise} \leq \mu \leq \mu_{Strong}$ and that μ_{Noise} tends towards μ.

Based on these findings, we standardize the forest IMD of variable v using the mean μ and the standard error of IMD of v. The standardization is represented by:

$$t_{M_v} = \frac{M_v - \mu}{SE(M_v)}$$

(16)

We denote t_{M_v} as the t-score IMD of variable v. This transformation yields a symmetric distribution (Supplementary Figure 1b bottom panel). The t-score IMD effectively differentiates between noise and cross-correlated variables. Noise variables cluster below the mean (μ), while cross-correlated variables significantly diverge from μ. Using the lower tail of the t-distribution at the 0.05 level ($t_{0.05,\mathrm{df=ntree-1}}$, denoted by the navy line), we identified the cross-correlated variables that have $t_{M_v} > t_{0.05,\mathrm{df=ntree-1}}$. This specific threshold represents a point below which only an expected 5% of the IMD values for cross-correlated variables fall, thus indicating a higher level of importance.

Multi-omics Framework

We now extend the variable selection framework to multi-omics data. While we have introduced the variable selection method in two-omics data, it is essential to recognize that the choice of which dataset to assign as responses or predictors can influence the results, particularly in complex datasets. Additionally, the strength of connections between datasets plays a critical role in multi-omics variable selection, as some omics layers may share minimal information, which can introduce bias into the selection process. To address this, we introduce an algorithm designed to efficiently identify optimal connections among multi-omics datasets. If a predefined connection structure is available, such as prior biological knowledge or experimentally verified links, we use these connections directly. Otherwise, we apply the method described below to infer data-driven connections.

To improve computational efficiency and address the high-dimensionality challenge in multi-omics data analysis, we applied principal component analysis to each dataset. PCA reduces the dimensionality of the datasets by selecting components that explain a predefined level of cumulative variance. This ensures that we retain the most relevant information while minimizing computational complexity. For each reduced dataset, we conducted multivariate random forest (MRF) modeling, matching each dataset as a response to all others as predictors. We evaluated these models by calculating the mean out-of-bag (OOB) error, which was used to rank the models. The direction with the lowest OOB error was selected as the optimal connection between the datasets. This process enhances efficiency by avoiding exhaustive pairwise modeling and retains the most informative variables for further analysis.

For multi-omics datasets, let $\mathcal{X} = \{\mathbf{X}^{(1)}, \mathbf{X}^{(2)}, \dots, \mathbf{X}^{(K)}\}$ denote a multi-omics dataset with K omics data, where $\mathbf{X}^{(k)} = [X_1^{(k)}, \dots, X_{p_k}^{(k)}] \in \mathbb{R}^{N \times p_k}$ denotes the k_{th} omics data with N data samples and p_k features. Let $\mathcal{M}$ be the the model collection that contains all optimal connected MRF models and $\mathbf{m}_{X^{(i)} \leftarrow X^{(j)}}$ is the model in $\mathcal{M}$ with the direction of $X^{(i)}$ as responses and $X^{(j)}$ as predictors. Algorithm 2 in Supplementary Note 5 summarized the framework of multi-omics variable selection under the variable filtering and mixture model methods. First, we compute the mean IMD for each dataset across model set $\mathcal{M}$. Instead of individual IMD,

we select the important variables based on the mean IMD. For multi-omics variable selection under the IMD transformation, we choose the variables that the majority of the model selects (See Algorithm 3 in Supplementary Note 6).

Simulation Study

We designed a comprehensive simulation study to evaluate the performance of the proposed IMD-based methods under various conditions. Two different models were used: a **latent model** and a **non-linear regression model**. For each model, we generated synthetic multi-omics datasets to assess the impact of dimensionality, noise, and varying proportions of cross-correlated variables on model performance.

To evaluate the performance of our proposed methods in variable selection, we conducted a comprehensive simulation study. We generated synthetic multi-omics datasets under various conditions to assess the impact of noise, high dimensionality, and different proportions of cross-correlated variables on the accuracy of our model. The simulation was designed to mimic realistic data integration challenges, where datasets contain a mixture of relevant and irrelevant variables. We compared our IMD-based methods with existing techniques such as sparse PLS (SPLS), penalized matrix decomposition CCA (PMDCCA), and sparse regularized generalized CCA (SGCCA), assessing their ability to select important variables across different scenarios.

To benchmark our IMD-based selection against nonlinear ensemble learners, we also included RF permutation importance measurement, gradient boosting machines (GBM) and XGBoost. Because these algorithms lack native support for multivariate or unsupervised multi-omics variable selection, we adapted them by fitting a separate univariate model for each response, computing per-feature importance scores, and then averaging those scores across responses to produce a single global ranking. We applied this procedure to three simulation frameworks, (i) a latent-factor model, (ii) a nonlinear regression model, and (iii) an interaction model in which the outcome depends on pairwise predictor interactions, each time generating paired two-omics data where one layer contained only true signal variables (as the outcome) and the other served as predictors. In every scenario, we compared the rankings produced by MRF-IMD to those from three integration methods (SPLS, PMDCCA, SGCCA) and the three ensemble learners, evaluating each method's ability to elevate known signal features via PR-AUC, true-positive rate in the top k, and ranking stability across replicates.

Latent Model. For the simulation of the linear models, we use the following model:

$$\mathbf{X}^{(m)} = g_m(u)w_m^T + \epsilon_m,$$

where $\epsilon_{mi} \sim \mathbb{N}_{p_m}(\mathbf{0}, \mathbf{\Sigma})$ for $i = 1, \ldots, p_m$, and u is generated from normal distribution $u \sim N(\mathbf{0}, \sigma^2)$ using mean 0 and $\sigma = 2$. For the kernel functions, we set $g_1(\mu) = \mu^2$, $g_2(\mu) = \exp(\mu)$, and $g_3(\mu) = \mu$ to transform the latent variable μ. The weights variable w_m were first generated by variables w_m^0 from uniform

distribution $w_m^0 \sim U(-1,1)$. Then, the variables were normalized in the following way to ensure that the sum of squared of w_m is equal to 1:

$$w_{mj} = \frac{w_{mj}^0}{\sqrt{\sum_{i=1}^{p_m}(w_{mi}^0)^2}}$$

For this model, we generated two-dataset and three-dataset settings. For each dataset, only the first p_m^c of weights w_m are non-zero and selected as the features to identify. Scenarios were generated based on the following parameters: n, p_m, p_m^c, where n represents the sample size of all datasets, p_m represents the feature numbers of $\mathbf{X}^{(m)}$, and p_m^c the number of features that crossed-correlated w.r.t. $\mathbf{X}^{(m)}$. Here we set the diagonal of the variance-covariance matrix $\mathbf{\Sigma}$ to 0.3^2 and all variables in the $\mathbf{X}^{(m)}$ were re-scaled to have a mean of 0 and a standard deviation of 1. To generate data, we used the scenarios described in Table 1a for the two- and three-data settings. For the ranking based comparison, we focused exclusively on the two-omics settings. In these settings, one omics layer (the "outcome" block) contained only the p_m^c true predictors and the other layer served as its predictors. We then evaluated the ability of each models to recover the true features into the top p_m^c of its ranked list.

Non-linear Regression Model. This simulation model was inspired by the simulation model in Degenhardt et al.[22] . Let U be the basis variables that are generated from a distribution. For this model, we will add four additional parameters, g, p_d^1, and p_d^2, where g represents the group size of each correlated group in $\mathbf{X}$, and p_d^1, p_d^2 represent the number of variables that are not cross-correlated in $\mathbf{X}$ and $\mathbf{Y}$ respectively. This time, we use p_l to represent the total number of basis that formed $\mathbf{Y}$. To generate the correlation between $\mathbf{X}$, the simulation is according to:

$$X_i^{(j)} = \begin{cases} U_i + \left(0.01 + \dfrac{0.5(j-1)}{g-1}\right) \cdot \epsilon, & \text{for } g > 1 \\ X_i = U_i + \epsilon, & \text{for } g = 1 \end{cases}$$

for $j = 1, \dots, g$ and $i = 1, \dots, p_l$, where $X^{(j)}$ denotes the j_{th} variable in group i. Note that when $g = 1$, there is no correlation between X_i. When $g > 1$, there will be g correlated variable in variable group i, the increase in j will decrease the correlation between basis variable $U_i^{(j)}$ and X_i. And notice that the total number of features that crossed-correlated variables in $\mathbf{X}$, p_1^c, is equal to $g \cdot p_l$.

To generate $\mathbf{Y}$, we use the following kernel function:

$$Y_k = f_k(u) = 0.25\exp(4u_{3\cdot k-2}) + \frac{4}{1+\exp\left(-20(u_{3\cdot k-1} - 0.5)\right)}, \quad k = 1, \dots, p_2^c$$

where each cross-correlated Y_k is formed by 2 basis variables for a total of $2 \cdot p_2^c$ number of basis. Therefore, $p_l = 2 \cdot p_2^c$.

Finally, we will generate two sets of noise variables for $\mathbf{X}$ and $\mathbf{Y}$ respectively using multivariate Gaussian distribution with mean 0 and identity variance-covariance matrix. These two sets of variables are neither cross-correlated nor inner-correlated to each other. The number of independent sets for $\mathbf{X}$ is $\mathrm{p}_{\mathrm{d}}^{1}$ and the number of independent sets for $\mathbf{Y}$ is $\mathrm{p}_{\mathrm{d}}^{2}$. Note that all variables in the $\mathbf{X}$ and $\mathbf{Y}$ matrix are re-scaled to have mean 0 and standard deviation 1 after generating by the above models. We generated two settings of cross-correlated variables, and in each setting, we generated three different dimensional scenarios as shown in Table 1b. For the ranking based simulation, the true signal set comprises the first $p_1^c = gp_l$ features of $\mathbf{X}$. Each method produce a ranking over all p_1 candidate features and we qualify the recovery of the p_1^c true features.

Three-way non-linear interaction To further challenge the ability of each method to uncover higher order effects for the ranking based comparison, we simulated a purely nonlinear three-way interaction. We generated $n = 200$ samples of $p = 300$ independent predictors $X_i \sim N(0,1)$, then selected (X_1, X_2, X_3) as the shared interacting trio and added two "side" predictions per outcome (X_4, X_5) for Y_1 and (X_6, X_7) for Y_2. Specifically, the latent signals were

$$\eta_1 = (X_1^2 - 1)(X_2 + 0.5)(X_3 - 0.5) + X_4 + X_5, \quad \eta_2 = (X_1^2 - 0.5)(X_2 + 0.5)(X_3 - 0.5) + X_6 - X_7.$$

and set the corresponding response

$$Y_1 = 1.5\eta_1 + \epsilon_1, \;\; Y_2 = 2\eta_2 + \epsilon_2 \quad \epsilon_{k\in 1,2} \sim \mathcal{N}(0, \sigma^2),$$

After centering and scaling all columns of X and Y to unit variance, we tasked each algorithm with ranking the full set of 300 predictors. We then compared nonlinear ensemble learners (random forests with permutation-importance, GBM, XGBoost) against multi-omics integration methods (SPLS, PMDCCA, SGCCA) by measuring each method's PR–AUC and true-positive rate among the top kp_c features per response.

All MRF-IMD models were implemented via the *rfsrc* function in the randomForestSRC R package, using its out-of-the-box settings (default mtry, nodesize, and splitting rules) across every simulation scenario. For the integration variable selection comparison, we compared our methods to SPLS (mixOmics R package), PMDCCA (PMA R package), and SGCCA (RGCCA R package). For PMDCCA and SGCCA, we selected variables were tuned by the build-in tuning functions of their packages. For SPLS, we directly entered the number of true variables to the function. For the ranking based comparison, we added the ensemble methods, random forests (permutation-importance), GBM, and XGBoost and recorded the importance scores that generated by their respective R packages (randomForest, gbm, and xgboost). For the integrative methods (SPLS, PMDCCA, and SGCCA), we collapsed each into a single ranking by averaging the absolute loadings over the first five components. This approach ensured that every method returned a complete ranking over the same candidate set.

Evaluation Metrics

For each simulation scenario, we evaluated the performance of the variable selection methods using the following metrics: recall, precision, area under the precision-recall curve (PR-AUC), and model size.

Recall. This metric measures the proportion of true important variables identified by the model. Let tp be the number of variables the model selected as important that are true important variables, and fn be the number of variables that the model selected as important variables that are noise variables. The recall equals $\frac{\mathrm{tp}}{\mathrm{tp+fn}}$. A higher recall indicates that more important variables were selected.

Precision. This metric measures the accuracy of the variable selection, focusing on the proportion of correctly identified important variables out of all selected variables. Let fp be the number of variables that the model selected as important that are true non-important variables, and tp described above. The precision equals $\frac{\mathrm{tp}}{\mathrm{tp+fp}}$. Higher precision indicates that fewer noise variables were incorrectly selected.

PR-AUC. This is a common metric for imbalanced datasets where important variables (positive cases) are fewer compared to noise variables (negative cases). PR-AUC ranges from 0 to 1, with a value of 1 indicating a perfect classifier. We reported the average PR-AUC across all datasets.

Model size. This represents the number of variables selected by the model. We calculated the average model size for each simulation scenario and reported the standard deviation to capture the variability in model size across replicates. For stability, each scenario was simulated 50 times, and all evaluation metrics (except model size) were averaged across replicates. For model size, we reported the average and standard deviation to assess the variability in the number of selected variables.

Real Data Preprocessing

TCGA Data To demonstrate the effectiveness of our proposed methods, we applied our methods to three TCGA datasets: breast invasive carcinoma (BRCA)[23], colon adenocarcinoma (COAD)[24], and Pan-Cancer[25]. For TCGA-BRCA and TCGA-COAD data, we analyzed three types of omics data (i.e., mRNA expression data (Gene), miRNA expression data (miRNA), and DNA methylation data (Methyl)) were selected to perform analysis. For TCGA-Pan-Cancer, we selected the ATAC sequencing data and RNA sequencing (RNA-seq) datasets. All the RNA-seq data, which were log2-transformed transcripts per million (TPM) for each cancer type, were obtained from the R package *TCGAbiolinks*. Other datasets were downloaded through UCSC Xena (https://xena.ucsc.edu/)[26]. For TCGA-Pan-Cancer, we selected the ATAC sequencing data and RNA sequencing datasets. Each of these data types provides unique, yet complementary, information for distinguishing between different types or states. Analyzing RNA-seq and ATAC-seq independently, however, can lead to inconsistent classifications. Furthermore, studying these two modalities in isolation may diminish the overall power of the analysis, as they both represent the same fundamental

types or states. Only samples that existed across all data types were included in our study. We applied log2-transformation to mRNA and miRNA expression data. For DNA methylation, we initially filtered out probes not included in the Illumina Infinium HumanMethylation450k BeadChip to ensure a better interpretation of results. For miRNA, we removed all the variables that contain missing values. To streamline our analysis, we limited the features of mRNA and DNA methylation data to the top 2000 most variable expressions in both the BRCA and COAD datasets. For the Pan-Cancer dataset, we restricted ATAC-seq and RNA-seq to the top 50,000 and 5,000 most variable expressions, respectively. Table 2 summarizes the datasets used in the analysis.

ADNI Data We analyzed data from the Alzheimer's Disease Neuroimaging Initiative (ADNI), including DNA methylation m profiles for 538 participants cognitive normal (CN) and mild cognitive impaired (MCI) patients measured on Illumina HumanMethylation EPIC v1 arrays preprocessed by Zhang et al.27 and matched gene-expression data from Affymetrix Human Genome U219 microarrays. In this study, we mainly focus on dementia onset. Given the heterogeneity and complex progression of Alzheimer's disease (AD), we first selected the top 2,000 CpG methylation sites most significantly associated with AD dementia onset, using p-value–based screening in the Framingham Heart Study (FHS) dataset as described by Zhang et al.[27] For gene expression, we removed probes lacking gene symbols, removed lowly expressed probes with gene expression below the 10th percentile in over 80% of samples, collapsed remaining probes by gene via median values, and finally selected the 2,000 most variable genes. Dementia conversion was defined as the conversion from CN to MCI or dementia, and from MCI to dementia. After intersecting dementia phenotype, DNA methylation and gene expression data, we obtained 468 common samples for our integrative analyses. DNA methylation, gene expression data and the dementia status of the subjects were obtained from the ADNI study website (adni.loni.usc.edu). Table 2 summarizes the datasets used in this analysis.

RESULTS

Summary of the MRF-IMD Framework

Our integrative multi-omics pipeline targets datasets in which multiple omics layers share the same samples. We aim to identify shared biomarkers, that is, variables in one omics block whose variation is strongly associated with features in another block. To do this, we first establish a connection structure between layers: if prior biological knowledge or experimentally verified links exist, we adopt these directly; otherwise, we infer an optimal connection based on cross-block correlation. Given this pairing, we fit a multivariate random forest (MRF) model for each connected pair, treating one omics layer as the multivariate response and the other as predictors. From each tree in the forest, we compute the minimal depth of every predictor (the depth at which it first splits), convert these to inverse minimal depth (IMD) scores, and average across all trees to yield a global importance ranking. Finally, we apply one of three IMD-based selection strategies, Filter (user-defined threshold), Mixture (balanced sensitivity–specificity), or Transformation (distribution-

normalized ranking), to extract a compact set of cross-omics features. Downstream, these selected biomarkers are evaluated via functional enrichment, patient stratification, and clustering analyses. The entire workflow is illustrated in Figure 1.

Evaluation of Simulated Data

Integrative Variable Selection Benchmark The variable selection results are summarized in Figure 2 and Table 3 (Supplementary Note 7). Under the latent-factor model (Figure 2a, Table 3a), all three MRF-IMD variants delivered competitive PR-AUC and TPR compared with SPLS, PMDCCA, and SGCCA, matching these methods in the very linear scenarios they were designed for. By contrast, in the nonlinear-regression simulations (Figure 2b, Table 3b), IMD-filter, IMD-mixture, and IMD-transformation significantly outperformed the CCA-based integrators, maintaining high PR-AUC and stable TPR even as model complexity increased. Among the IMD methods, filter was most conservative, mixture struck the strongest sensitivity–specificity balance, and transformation excelled at highlighting signals when IMD weights were tightly clustered. Extended simulation results are provided in Supplementary Note 7.

Ranking-based Comparison In ranking-based simulations across three models, latent, three-way interaction, and nonlinear regression models, our MRF-IMD methods consistently achieved competitive PR-AUC results at the top k predictors. The boxplot from Supplementary Figure 2 shows that the IMD scores achieve the stable results across all scenarios. Extended simulation results are provided in Supplementary Note 7.

In summary, our simulation studies confirmed that the MRF-IMD framework can identify cross-correlated variables reliably under a wide array of conditions, outperforming several well-established methods including integrative methods and nonlinear ensemble methods. These results suggest that by embracing nonlinear modeling, leveraging IMD-based variable selection, and providing flexible selection strategies, our framework is well-suited for integrative multi-omics analysis. This strong performance in controlled simulations sets the stage for more complex real-world applications and further supports the potential utility of MRF-IMD methods in guiding biomarker discovery and driving meaningful biological interpretations.

Comprehensive Analysis of Individual Cancer Data: Breast Cancer and Colorectal Cancer

Data Summary and Model Configurations We applied our MRF-based framework to breast invasive carcinoma (BRCA) and colon adenocarcinoma (COAD) data from TCGA. For BRCA, we examined two directional models to reduce the model running: $gene \leftarrow methyl$ and $mirna \leftarrow methyl$, and for COAD we considered $mirna \leftarrow methyl$ and $mirna \leftarrow gene$ configuration that selected by the optimal connection. Key cohort and feature counts for each cancer and omics layer are summarized in Table 2.

Stability and Sensitivity Analysis To assess the stability of the identified variables of our MRF-IMD method at different seed settings. We repeated running the MRF-IMD model 30 x on different seeds and summarized (i) model size (ii) the pairwise overlap coefficient (also known as the Szymkiewicz–Simpson coefficient-intersection divided by the smaller set size) for each omics block. Across BRCA and COAD, **Filter** yielded the most compact yet stable signatures; **Mixture** provided a balanced middle ground; **Transformation** returned the broadest panels with still high reproducibility. These patterns were consistent across genes, CpGs, and miRNAs. Supplementary Figure 3 summarizes the stability of gene, CpG and miRNA selections across 30 MRF-IMD runs for both TCGA-BRCA (top panels) and TCGA-COAD (bottom panels). In BRCA, the filter strategy consistently yields the most compact signatures (median ≈ 73 genes, 91 CpGs, 33 miRNAs) and attains high reproducibility (overlap coefficients ≈ 0.80 for genes, 0.90 for CpGs, 0.95 for miRNAs). The mixture approach selects intermediate-sized sets (median ≈100 genes, 140 CpGs, 27 miRNAs) with slightly lower median overlaps (0.78/0.85/0.98), while transformation produces the largest signatures (≈ 235 genes, 290 CpGs, 45 miRNAs) but still maintains strong stability (0.75/0.80/0.90). In COAD, all three methods expand their gene and CpG panels relative to BRCA, yet the same ranking of stability holds. Filter picks ~120 genes, 75 CpGs, 43 miRNAs (overlaps ≈ 0.68/0.78/0.90); mixture yields median selection of 145/125/15 features (overlaps ≈ 0.70/0.75/1.00); and transformation selects median selection of 120/130/60 features with moderate reproducibility (0.62/0.65/0.85). These results demonstrate that, regardless of cohort or omics layer, IMD-transformation offers the best trade-off between breadth and consistency, IMD-filter delivers the most parsimonious yet stable core signature, and IMD-mixture provides a middle ground in both model size and overlap.

Signature Selection for Comparative Evaluation For the rest of the analysis, we adopted the IMD-mixture variable selection strategy as it delivers a balanced signature size (neither too sparse nor overly broad) while maintaining high selection stability across seeds. For reproducible research, we selected the seed for the model fitting that was closest to the median across 30 seeds. To enable a fair head-to-head evaluation, we then configured SPLS, PMDCCA, and RGCCA to yield the similar total number of features. For SPLS (mixOmics), we specified the `keepX` vector in `block.spls` function to match the per-component counts from IMD-mixture. PMDCCA (PMA) was run with its default `CCA.permute` routine, which, if no penalty is supplied, automatically selects optimal penalty terms via permutation testing. For RGCCA (RGCCA), we manually set the shrinkage penalties to 0.1 to ensure the selection sparsity. All integrative models were run with five components for the downstream prognostic analyses, yielding comparable feature-set sizes across methods.

Interpretation of Selected Biomarkers Figure 3a top panel highlights the top 20 BRCA biomarkers by the IMD-mixture weight across genes (left), CpG probes (center), and miRNA (right). Prominent genes included *FOXC1*, *PRR15*, and *PRKCQ*. *FOXC1* drives epithelial–mesenchymal transition and correlates with poor survival in breast cancer; *PRR15* has recently been identified as a luminal-subtype marker in hormone

receptor–positive tumors[28]; and *PRKCQ* suppresses ERα expression and is required for mammary tumorigenesis in triple-negative models[29]. Other high-ranking genes include *MMP11,* which is well-documented in the literature as playing a pivotal role in breast cancer, showing high expression levels in early luminal subtypes[30,31]. Additionally, *BCL11A* has also been shown to be related to triple-negative breast cancer[32]. Furthermore, *ESR1*, the gene that encodes the estrogen receptor (ER) along with pioneering transcription factor *FOXA1* is well established factors in hormonally dependent breast cancer[33]. Among the DNA methylation features, probes such as cg03441279 in *BCL9* and cg12427162 in *SFT2D2* have been associated with breast cancer prognosis[31][34]. On the miRNA side, MIMAT0003249 (hsa-miR-584-5p) and MIMAT0000064 (hsa-let-7c-5p) stood out, both having been previously implicated in breast cancer biology[35,36].

For the TCGA-COAD dataset (Figure 3a bottom panel), two of the top genes selected by the model, *SFRP4* and *ANGPTL1*, are known to be highly expressed in colorectal cancer (CRC) and have been linked to poor clinical outcomes in CRC patients[37–39]. Although fewer DNA methylation probes emerged prominently, cg12374721, which maps to the *PRAC2* locus, exceeded the 0.5 weight threshold. This site was previously flagged as a novel pan-cancer methylation biomarker, showing consistent differential methylation in colon, rectal, and prostate tissues[40,41]. For miRNAs, MIMAT0000098 (hsa-miR-100-5p) and MIMAT0000076 (hsa-miR-21-5p) are top ranked; miR-100 is significantly downregulated in colorectal tumors, and lower miR-100 expression correlates with advanced stage and poorer overall survival in CRC patients[42]. Additionally, study also shown that hsa-miR-21-5p is useful in diagnosis of CRC[43].

Functional Analysis and Prognostic Comparison We then applied the functional enrichment analysis to the selected genes. The functional analysis was performed using clusterProfiler R package focusing on Canonical (C2:CP), GO (C5:BP) and Hallmark pathways from the Molecular Signatures Database[44]. Figure 3b displays the top 10 significant pathways (i.e. FDR < 0.05). In BRCA, the leading Canonical pathway hits are *Constitutive Signaling by Aberrant PI3K in Cancer*, *PI3K AKT Signaling in Cancer*, and multiple *ERBB4*-driven routes, reflecting the centrality of PI3K/AKT and ERBB networks in hormone-receptor-positive breast tumors[45,46]. In GO, *Gland Development*, *Mammary Gland Epithelium Development*, and *Positive Regulation of Cell–Cell Junction Assembly* are crucial in understanding tumorigenesis, particularly in cancers like breast cancer[47]. In COAD, enriched Canonical pathways are dominated by extracellular matrix remodeling (*ECM Proteoglycans*, *Extracellular Matrix Organization*), while GO terms center on muscle and connective-tissue processes (*Muscle Contraction*, *Cartilage Development*, *Collagen Fibril Organization*). Notably, the Hallmark sets *Epithelial–Mesenchymal Transition* and *Myogenesis* also score among the top hits, underlining the dual roles of stromal reprogramming and EMT in colorectal cancer progression[48,49]. All the significant pathways obtained from MRF-IMD selected genes for BRCA and COAD are listed in Supplementary Table 1. In BRCA, MRF-IMD uniquely recovers PI3K/AKT– and ERBB-driven programs central to hormone-receptor–positive disease, which linear integrators often miss at matched model sizes.

In COAD, MRF-IMD enriches ECM remodeling and EMT pathways, aligning with stromal reprogramming in colorectal tumor progression.

Figure 3c shows the four-way Venn diagrams of the significant pathways selected by MRF-IMD, SPLS, PMDCCA, and RGCCA in BRCA and COAD. In BRCA (left), MRF-IMD identified 55 unique pathways (25.3%), while SPLS, PMDCCA, and RGCCA only identified 27 (12.4%), 35 (16.1%), and 37 (17.1%) unique pathways, respectively. Only 37 pathways (17.1%) overlap between MRF‑IMD and SPLS, and 40 (20.3%) between MRF‑IMD and RGCCA; there are no shared pathways in PMDCCA or common to all four methods. The core oncogenic pathways such as *Constitutive Signaling by Aberrant PI3K in Cancer* and *PI3K AKT Signaling in Cancer* are uniquely retrieved by MRF-IMD that the linear integrators might miss. In COAD, MRF-IMD again dominates with 48 unique enrichments (64.0%), whereas SPLS yields none, PMDCCA yields 2 (2.7%), and RGCCA yields 12 (16.0%). Only 4 pathways (5.3%) overlap between MRF‑IMD and RGCCA, and 2 (2.6%) between MRF‑IMD and PMDCCA; there are no shared pathways in SPLS or common to all four methods. Among its unique COAD hits, MRF‑IMD highlights *Collagen Fibril Organization* and *Ossification*, underscoring its ability to capture tissue-specific remodeling programs that go beyond the canonical and component-based integrators.

An important goal in cancer multi-omics studies is to identify biomarkers that not only reflect biological mechanisms but also correlate with clinical outcomes. To evaluate the prognostic value of the variables identified by our MRF-based methods, we applied the integrative non-negative matrix factorization (IntNMF) method[50] to combine the selected variables from the three omics data types for both BRCA and COAD. This integration allowed us to cluster patients into two groups representing high- and low-risk survival profiles. Figure 3d shows the resulting Kaplan–Meier curves of the grouping results from clustering using all variables, top 5 SPLS components, and the variables selected MRF-IMD-mixture methods. Table 4 reports the median log-rank p-values across 30 seeds of MRF-IMD methods and log-rank p-values of other benchmark methods. In BRCA, clustering on all variables yields no significant separation (*P*-value =0.28), while SPLS and the CCA‑based methods achieve modest significance only when using all five components (SPLS: *P*-value $=8.2 \times 10^{-3}$; PMDCCA: *P*-value $=1.4 \times 10^{-2}$; RGCCA: *P*-value $=2.4 \times 10^{-2}$) but fail when restricted to their selected features. In contrast, MRF-IMD's filter and mixture strategies produce highly significant stratification (median *P*-value $= 7.9 \times 10^{-4}$ and 1.0×10^{-3} respectively), with the filter set delivering the strongest separation in the Kaplan–Meier curves (*P*-value $=4 \times 10^{-4}$). In COAD, neither the full feature set (*P*-value =0.24) nor SPLS selected variables‑or RGCCA‑derived signatures yield significant stratification. SPLS with all five components and PMDCCA's selected variables attain significance (*P*-value$=1.3 \times 10^{-2}$ and 3.0×10^{-2}), but once again the MRF-IMD filter and mixture panels outperform, both achieving *P*-value $=1.2 - 1.4 \times 10^{-2}$.

Together, these results demonstrate that our MRF-IMD–derived biomarker sets consistently enable more robust risk stratification than existing integrative methods, confirming their potential clinical utility for patient stratification and prognostic modeling.

In summary, applying our MRF-based framework to BRCA and COAD datasets uncovered key genes, methylation probes, and miRNAs that are consistent with known cancer biology. The enriched pathways and strong associations with survival outcomes highlight the potential of these methods to identify meaningful biomarkers in multi-omics data, guiding future research and, potentially, clinical translation.

TCGA PAN Cancer Clustering Analysis

Visualization of PAN Cancer To further demonstrate the flexibility and scalability of our framework, we applied the IMD-transformation variable selection method to a pan-cancer dataset from TCGA, encompassing 22 distinct cancer types. The summary of sample size and feature counts are detailed in Table 3. After processing and feature selection, we retained 186 ATAC-seq features and 300 RNA-seq features for integrative analysis using IMD-transformation strategy. We first visualized the data using Uniform Manifold Approximation and Projection (UMAP), which revealed clear and distinct clusters that corresponded to different cancer types (Figure 4). In the UMAP embeddings based on all ATAC-seq (Figure 4a) or RNA-seq (Figure 4b) features, tumor types form loose, overlapping clouds that reflect broad tissue similarities but offer limited discrimination. By contrast, the UMAP projection of the joint IntNMF embedding from MRF-IMD–prioritized ATAC and RNA features (Figure 4c), revealed tightly bound, clearly separated clusters, demonstrating that these selected variables capture the core axes of regulatory and transcriptional variation across cancer types.

Pan-Cancer Clustering We next applied IntNMF directly to the MRF-IMD feature set and determined an optimal rank of eight clusters using `nmf.opt.k` function from the IntNMF R package. Figure 4d shows the resulting confusion matrix, illustrating how our selected features effectively separated the samples into eight clusters. Each cluster highlighted unique molecular characteristics and captured established patterns of tumor heterogeneity, ranging from a combined basal-like breast and uterine carcinoma cluster (Group 1) through gastrointestinal adenocarcinomas (Group 5) and hepatobiliary tumors (Group 2) to hypermutated immunogenic cancers (Group 3), squamous-cell carcinomas (Group 7), endocrine neoplasms (Group 6), and renal epithelial tumors (Group 8). A detailed description of the these clusters is in Table 5. To further quantify the advantage conferred by MRF-IMD feature selection, we applied IntNMF clustering to the pan-cancer dataset, aiming for 20 clusters. We first excluded cancer types with fewer than five samples (TCGA-CESC and TCGA-CHOL), as very small cohorts can produce unstable clusters driven by outliers or noise. We then quantified cluster recovery by computing the adjusted rand index (ARI) between the 20 IntNMF clusters on MRF-IMD features and the true TCGA tumor-type labels (Supplementary Figure 4a), benchmarking against four alternative strategies: PAM clustering on 30 SPLS components (Supplementary Figure 4b); IntNMF on the full feature set (Supplementary Figure 4c); PAM clustering on RNA-seq data alone (Supplementary Figure 4d); and PAM clustering on ATAC-seq data alone (Supplementary Figure 4e). Although all approaches are shown moderate to high performance, our method still shows advantage with slightly higher ARI of 0.728. Nearly every TCGA cohort is assigned to a single dominant cluster: COAD (36 out of 37 total samples in group 16), KIRP (31 out of 32 in group 7), PRAD (25 out of 26 in group 15), and

UCEC (12 out of 12 in group 11) all show almost perfect one-to-one mapping. In breast cancer, the algorithm distinguished three biologically meaningful subgroups: Group 13 captured a pure basal-like subtype (all 13 basal tumors); Group 5 combined all HER2 (9 out of 10) and LumB (16 out of 16) cases with most LumA (18 out of 29) samples, reflecting a non-basal, high-risk profile; and group 1 comprised predominantly LumA tumors (9 out of 11) alongside normal controls, defining a lower-risk, luminal-A–driven cluster. Smaller lineages such as BLCA, ESCA, and STAD likewise concentrate into their own clusters with minimal leakage. By contrast, the other methods produced more fragmented assignments and lower ARI scores, SPLS+PAM (ARI = 0.697), full-feature IntNMF (0.687), RNA-seq only (0.713), and ATAC-seq only (0.675), underscoring MRF-IMD's superior ability to isolate coherent, biologically relevant tumor groups.

We report ARI (chance-adjusted) rather than precision/recall because clustering is unsupervised and multi-class; ARI summarizes concordance without requiring a positive class. MRF-IMD features achieve the highest ARI among tested strategies and yield clean, tissue-coherent clusters.

By capturing these known and biologically meaningful patterns, our MRF-IMD framework shows its utility in differentiating tumor types, identifying key molecular signatures, and offering a more integrated view of tumor diversity. These results illustrate the method's promise for guiding future studies on cancer classification, patient stratification, and uncovering novel therapeutic targets across a wide range of malignancies.

Integrative Analysis Enhances Prediction of Dementia Progression in the ADNI Cohort

MRF-IMD Selected Genes To further illustrate the superior results of our MRF-IMD method, we applied the variable selection to the ADNI data using the filtering strategy. A total of 161 genes and 54 CpG sites were selected by this strategy. Figure 5a shows the top twenty gene-expression and DNA methylation features prioritized by our MRF-IMD framework in the ADNI cohort. On the left panel, *ARL11* has the largest weights. While its direct role in AD is still under investigation, *ARL11* is known to be involved in apoptosis and immune system processes, which are critical components of neuroinflammation in AD[51]. Followed by *ARL11*, *S1PR1* plays a significant role in the neuroinflammatory processes of AD[52]. *DAPK2* indicates the involvement of death-associated kinase-mediated neuronal apoptosis and tau dysregulation[53], although the precise nature of its contribution to AD pathology requires further investigation. *CCR7* reflects its established involvement in chemokine-mediated microglial trafficking and neuroinflammation, with studies indicating that reduced *CCR7* expression on meningeal T cells in aging is linked to worsened glymphatic function, cognition, neuroinflammation, and β –amyloid pathology[54]. Functional analysis of the selected genes identified 36 pathways with FDR < 0.05 (Supplementary Table 2). The enrichment profile was dominated by Lymphocyte (B cells, T cells, NK cells) programs, especially in T cells. Key terms included *T Cell Differentiation and Activation*, *Lymphocyte Differentiation and Activation*, supporting a peripheral inflammatory state relevant to Alzheimer's disease[55,57].

Pathway Analysis of Selected DNAm CpGs To assess the biological relevance of our MRF-IMD selected CpGs versus those significant CpGs reported by Zhang et al., we performed KEGG and GO enrichment with the missMethyl R package on both the 54 MRF-IMD prioritized sites (top 20 out of 54 sites showed in Figure 5a right panel) and the 44 meta-analysis significant sites. Supplementary Table 3 displays the top 15 most significant pathways for each method. In the KEGG analysis, MRF-IMD CpGs showed strongest enrichment in *NF-κB Signaling*, *C-type lectin Receptor Signaling*, and *Leukocyte Transendothelial Migration*. Together these pathways indicate coordinated innate immune activation and immune-cell trafficking across the endothelium, processes that escalate neuroinflammation and contribute to AD progression. These results are concordant with the pathway enrichments obtained from the selected gene set. By contrast, the Zhang et al. meta-analysis CpGs were enriched for viral-infection and adhesion processes, top hits were *Virion–Ebolavirus, Lyssavirus and Morbillivirus*, *Cell Adhesion Molecules* and *mTOR Signaling*, indicating a shift toward pathogen-related and cell–matrix interaction pathways.

In the GO analysis, MRF-IMD CpGs emphasized post-transcriptional RNA processing (*Polyribonucleotide Nucleotidyltransferase Activity*; *Nuclear and Mitochondrial Polyadenylation-Dependent mRNA Catabolic Processes*; *Poly(U) RNA Binding*), along with cytokine regulation via I*nterleukin-1 Type I/II Receptor Antagonist Activity* and neuromodulatory pathways including *Wnt Signaling* and *Galanin Receptor Binding (Types 1–3)*. Notably, these CpG-derived enrichments align with the gene-based pathways through shared immune modules such as *Interleukin-1 Receptor Antagonist Activity* and *Wnt Signaling*, while the RNA processing and galanin receptor terms appear CpG-specific. In contrast, the meta-analysis CpGs were dominated by cell-division and cytokinesis terms (*Protein Localization to Division Site*; *Cleavage Furrow*; *Mitotic Cytokinetic Regulation*). These comparisons underscore that our MRF-IMD approach yields CpGs tied to innate immune signaling, mRNA processing and metabolic regulation that were more detailed and emphasized core processes directly associated in AD.

Integrative Validation on Dementia Progression To demonstrate that our integrative variable-selection outperforms both the single-layer methylation risk score (MRS) from Zhang et al. and an integrative approach without feature selection, we compared three stratifications of dementia conversion. We applied intNMF to the MRF-IMD selected features, obtaining two clusters, and evaluated time to dementia conversion using Kaplan–Meier curves and a log-rank test. The MRF-IMD panel showed a significant separation (Figure 5b; *P*-value = 0.033). We benchmarked three alternatives (Figure 5c): (left) intNMF on the full, unfiltered full omics set (*P*-value = 0.048); (middle) the published MRS based on 151 CpGs, dichotomized at the data-driven cut-point using MaxStat (*P*-value = 0.048); and (right) intNMF on the first five SPLS components from the full omics set (*P*-value = 0.60). Across comparisons, the MRF-IMD hubs delivered the strongest prognostic discrimination, outperforming methylation-only scoring and unfiltered or dimension-reduced integration.

In the original Zhang et al. study, the MRS was built via ridge regression on CpGs significantly associated with conversion and tested in a multivariate Cox model adjusted for age, sex, APOE $\epsilon 4$ status, years of

education, baseline diagnosis, and baseline MMSE score (Surv(conversion event, follow-up) ~ MRS + covariates). When we substituted our IntNMF-derived component from the MRF-IMD features into this identical Cox framework with the MRS, we observed a stronger association with progression to the next disease stage (Table 6). This result confirms that our integrative variable-selection not only refines molecular subtyping but also enhances prediction of disease progression beyond both single-omics risk scores and non-prioritized integrative analyses.

DISCUSSION

The continuous growth of high-throughput technologies has enabled the profiling of multiple omics layers—spanning the genome, epigenome, transcriptome, and beyond—within the same samples. This multi-omics landscape offers the potential for more complete insights into disease mechanisms, biomarker discovery, and therapeutic targeting. However, integrating these disparate data types and identifying meaningful shared features remains an ongoing challenge.

In this study, we presented a multivariate random forest (MRF)-based framework enhanced by the inverse minimal depth (IMD) metric to address these challenges. By combining the strengths of MRF for capturing nonlinear relationships and the IMD-based strategies for unsupervised feature selection, our approach provides a flexible and robust solution for multi-omics integration. Unlike conventional linear methods such as SPLS and CCA, which often assume simpler data structures and can be prone to overfitting, the MRF-IMD framework scales well to complex, high-dimensional scenarios. To accommodate diverse analytical goals and user preferences, we offer three IMD-based selection strategies. The filter approach, selecting variables above a tunable threshold parameter, τ, gives the user direct control over signature sparsity via standard-deviation–based cutoff. For a balanced trade-off between sensitivity and specificity, the mixture strategy consistently produced intermediate-sized panels with strong predictive performance. Finally, when a broader, more exploratory feature set is desired, the transformation method excels at highlighting subtle signals by normalizing IMD distributions.

Our simulation studies showed that MRF-IMD methods consistently outperform established approaches in identifying cross-correlated variables. The results held true under diverse conditions, including linear, nonlinear, and interaction models, varying sample sizes, and multiple levels of dimensionality and noise. Notably, MRF-IMD matched the accuracy of SPLS, PMDCCA, and SGCCA in simple linear scenarios, and greatly outperformed these methods as data complexity increased (Figure 2; Table 3). Moreover, when benchmarked against popular nonlinear ensemble methods, such as univariate random forests, GBM and XGBoost, on variable ranking tasks, our approach achieved markedly better performance (Supplementary Figure 2). These findings highlight the robustness and adaptability of our method, reinforcing its suitability for real-world applications.

We further demonstrated the framework's utility using multi-omics data from The Cancer Genome Atlas (TCGA). In breast and colorectal cancer, our approach uncovered known cancer-related genes, miRNAs, and DNA methylation features, as well as biologically relevant pathways. In breast cancer, our framework

identified top gene candidates such as *FOXC1*, *PRR15*, and *PRKCQ*, along with key epigenetic (e.g. probes in *BCL9* and *SFT2D2*, associated with breast cancer prognosis) and miRNA features such as miR-584-5p and let-7c-5p that have documented roles in breast tumor progression. In colorectal cancer, the MRF-IMD-selected features likewise reflected key disease mechanisms. Top genes included *SFRP4* and *ANGPTL1*, which are both implicated in colorectal tumor aggressiveness. Our method also picked up a methylation site in the *PRAC2* locus (cg23960088), a region noted as a pan-cancer methylation biomarker in gastrointestinal and prostate tumors, indicating that the integrative approach can recover known epigenetic aberrations. These findings are well-aligned with known tumor biology. In addition, an advantage of our integrative approach is evident in the pathway analysis of selected genes. In TCGA-BRCA, the MRF-IMD gene panel was highly enriched for signaling cascades central to breast cancer. For example, *PI3K/AKT Signaling* appeared as a top canonical pathway, along with several ERBB4/HER-family routes. This is consistent with the pivotal role of the PI3K–AKT–mTOR axis in driving ER-positive breast tumors and endocrine resistance[46] (Figure 3b; Supplementary Table 1). In TCGA-COAD, the MRF-IMD-derived gene list showed strong enrichment for pathways related to the tumor microenvironment and EMT. Top hits included *Extracellular Matrix Organization* and *Proteoglycan Remodeling* pathways, which align with the known importance of stromal reprogramming in colorectal cancer progression (Figure 3b; Supplementary Table 1). It is worth emphasizing that many of these pathways were uniquely identified by MRF-IMD – linear integrative methods, CCA/SPLS, when given a comparable number of features, often failed to enrich for these key pathways (Figure 3c). Moreover, clustering patients based on selected variables revealed groups with distinct survival outcomes, underscoring the clinical relevance of our discoveries. Using the selected multi-omic features for patient stratification yielded significant prognostic separations in both BRCA and COAD cohorts. In a pan-cancer setting, we showed that the MRF-IMD method could detect key molecular differences among diverse tumor types, identifying clusters with characteristic genomic instabilities, pathway alterations, and tissue-of-origin patterns. For instance, basal-like breast cancers clustered tightly with uterine serous carcinomas (Group 1), reflecting their shared genomic profile of TP53 mutations and chromosomal instability[58]. Similarly, a cluster comprising colorectal, gastric, and esophageal adenocarcinomas emerged (Group 5), consistent with the known CIN (chromosomal instability) phenotype common to gastrointestinal epithelial cancers. Other clusters aligned with established categories: we observed a grouping of squamous cell carcinomas across different organs (esophagus, lung, head/neck; Group 7) characterized by TP53 mutations and RTK/RAS pathway activation, and a clear cluster of renal cell tumors (Group 8) distinguished by their unique metabolic and microenvironment profiles. These results suggest that the approach has broad applicability, enhancing our understanding of tumor heterogeneity and potential therapeutic targets. Furthermore, clustering on MRF-IMD features achieved a high adjusted Rand index (ARI) relative to true tumor types, outperforming clustering based on all features or features from linear dimension reduction. In practical terms, our integrative selection could potentially facilitate tumor classification and subtyping in large heterogeneous datasets, focusing attention on the most informative genomic and epigenomic markers while filtering out noisy, uninformative variables.

In the ADNI cohort, MRF-IMD again proved its merit by uncovering biologically plausible and prognostically relevant markers of cognitive decline. Unlike cancer, where tissue-specific pathways dominate, Alzheimer's disease involves complex systemic and brain processes. Our method highlighted genes such as *ARL11*, *S1PR1*, *CCR7*, and *DAPK2* among the top candidates. These findings illustrate how multi-omics integration can spotlight targets that are not the most significant in any single data source but are critical when considering disease pathways together. Moreover, our integrative approach improved the prediction of clinical outcomes in the ADNI cohort. We compared our multi-omics signature to a published methylation risk score (MRS) based on 151 CpGs from a large epigenome meta-analysis . Whereas the MRS alone did stratify patients to some degree, the MRF-IMD integrative signature achieved a more significant separation between progressors and non-progressors (by Kaplan–Meier analysis). In fact, when we incorporated the integrative component into a Cox regression (mirroring the original study's covariate-adjusted model), it yielded a stronger association with time-to-dementia than the MRS. This suggests that combining gene expression with methylation (guided by MRF-IMD to focus on the most relevant features) captures a more predictive composite biomarker of cognitive decline.

In summary, our method is particularly well suited for analyzing complex, cross-layer, and high-dimensional datasets, such as those encountered in cancer and neurodegenerative disorders. It also stabilizes "small-n, large-p" analyses, common in rare disease studies or clinical trials, by reducing the feature space to the most reproducible cross-omics signals. MRF-IMD is capable of uncovering biomarkers and regulatory patterns that may be missed by traditional linear methods, especially when nonlinear patterns or interactions are present. To ensure computational tractability and model convergence, we recommend a preliminary variable-filtering step, such as retaining the most variable features per layer (e.g. under 5,000 features). In downstream tasks like molecular subtyping and survival prediction, MRF-IMD produces compact, interpretable biomarker panels that achieve more robust patient stratification than single-omics approaches, as demonstrated across the TCGA-BRCA, TCGA-COAD, pan-cancer, and ADNI cohorts.

Our framework is most advantageous when (i) multiple omics layers share samples, (ii) cross-layer dependencies may be nonlinear or involve interactions, (iii) labels are scarce (unsupervised discovery), and (iv) stability/interpretability of selected features matter. In linear regimes with strong sparsity, SPLS/CCA can perform very well as our simulations confirm, but they degrade in nonlinear settings, where MRF-IMD maintains high PR–AUC and stable recovery. Conversely, deep learning models often require labels and do not provide transparent unsupervised importance, making them less suitable for our selection goal.

While our method provides clear advantages, some limitations remain. First, computation time may increase with more datasets and extreme high-dimensionality. Future research could focus on improving efficiency, potentially through parallelization or dimensionality reduction strategies that preserve essential biological signals. Second, further integration with downstream validation steps, such as experimental verification or functional assays, would help confirm the biological significance of the selected variables and strengthen the evidence for potential biomarkers. Third, although we prioritize cross-layer shared

biomarkers to capture system-level regulators, we recognize that unique, layer-specific features (e.g., methylation marks reflecting environmental exposure or miRNAs mediating post-transcriptional control) also carry important biological information. Future work in developing a promising extension of the framework that extracts both omics-specific and shared biomarkers would enable more comprehensive biological insights. Fourth, we acknowledge that we did not benchmark MRF-IMD against deep learning, based integrative frameworks, a limitation of our current evaluation. Deep neural architectures (e.g., graph convolutional networks or variational autoencoders) have shown promise for multi-omics integration, yet they are typically supervised, rely on large labeled datasets, and lack transparent, unsupervised feature-importance mechanisms. Consequently, they cannot be directly applied to our unsupervised, multi-response variable-selection setting without substantial adaptation. Future work comparing our framework with unsupervised or semi-supervised deep learning methods could further enrich the benchmarking and broaden the understanding of integrative strategies. Finally, additional studies are needed to assess the utility of MRF-IMD–selected features in downstream applications, such as tumor subtype clustering, survival prognosis, and treatment-response prediction, to fully demonstrate the translational potential of our integrative selection strategy.

In conclusion, our MRF-IMD framework represents a step forward in integrative multi-omics analysis. By balancing flexibility, scalability, interpretability, and robustness, it enables researchers to identify meaningful biomarkers and pathways that would be difficult to pinpoint using conventional approaches. As the field continues to generate increasingly complex data, methods like ours will be instrumental in translating multi-omics information into actionable insights that advance our understanding of health and disease.

DATA AVAILABILITY

- TCGA-BRCA
  - mRNA expression data (Gene): https://xenabrowser.net/datapages/?dataset=TCGA.BRCA.sampleMap%2FHiSeqV2&host=https%3A%2F%2Ftcga.xenahubs.net&removeHub=https%3A%2F%2Fxena.treehouse.gi.ucsc.edu%3A443
  - miRNA expression data (miRNA): https://xenabrowser.net/datapages/?dataset=TCGA.BRCA.sampleMap%2FmiRNA_HiSeq_gene&host=https%3A%2F%2Ftcga.xenahubs.net&removeHub=https%3A%2F%2Fxena.treehouse.gi.ucsc.edu%3A443
  - DNA methylation data (Methyl): https://xenabrowser.net/datapages/?dataset=TCGA.BRCA.sampleMap%2FHumanMethylation450&host=https%3A%2F%2Ftcga.xenahubs.net&removeHub=https%3A%2F%2Fxena.treehouse.gi.ucsc.edu%3A443
- TCGA-COAD
  - mRNA expression data (Gene): https://xenabrowser.net/datapages/?dataset=TCGA.COAD.sampleMap%2FHiSeqV2&ho

st=https%3A%2F%2Ftcga.xenahubs.net&removeHub=https%3A%2F%2Fxena.treehouse.gi.ucsc.edu%3A443
    - miRNA expression data (miRNA): https://xenabrowser.net/datapages/?dataset=TCGA.COAD.sampleMap%2FmiRNA_HiSeq_gene&host=https%3A%2F%2Ftcga.xenahubs.net&removeHub=https%3A%2F%2Fxena.treehouse.gi.ucsc.edu%3A443
    - DNA methylation data (Methyl): https://xenabrowser.net/datapages/?dataset=TCGA.COAD.sampleMap%2FHumanMethylation450&host=https%3A%2F%2Ftcga.xenahubs.net&removeHub=https%3A%2F%2Fxena.treehouse.gi.ucsc.edu%3A443
- TCGA-Pan-Cancer
    - ATAC sequencing data (ATACseq): https://xenabrowser.net/datapages/?dataset=TCGA_ATAC_peak_Log2Counts_dedup_sample&host=https%3A%2F%2Fatacseq.xenahubs.net&removeHub=https%3A%2F%2Fxena.treehouse.gi.ucsc.edu%3A443
    - RNA sequencing (RNAseq) data downloading code: https://github.com/TransBioInfoLab/multiRF-vs/blob/main/code/real_data/data_prepare.Rmd
- ADNI: adni.loni.usc.edu

Access:
- RNAseq datasets: from R package TCGAbiolinks
- Others: from UCSC Xena: https://xena.ucsc.edu/

AVAILABILITY OF SUPPORTING SOURCE CODE AND REQUIREMENTS

Project name: An Integrative Multi-Omics Random Forest Framework for Robust Biomarker Discovery

Project homepage: https://github.com/TransBioInfoLab/multiRF-vs

Vignette: https://rpubs.com/noblegasss/multiRF-vs-vignette

Operating system: Linux (Ubuntu)

Programming language: R version 4.4.2

License: GPL 3.0 or higher

SUPPLEMENTARY DATA

Supplementary Data are available at GigaScience online.

FUNDING

This work was supported by National Cancer Institute grants R01CA200987, P50CA098131, P30CA240139, R01AG062634, R61NS135587, RF1NS128145, Department of Defense Breast Cancer Research Program BC201286, and funding from Sylvester Comprehensive Cancer Center

CONFLICT OF INTEREST

The authors declare no conflicts of interest.

REFERENCES

1. Subramanian I, Verma S, Kumar S, Jere A, Anamika K. Multi-omics Data Integration, Interpretation, and Its Application. *Bioinform Biol Insights*. 2020;14:1177932219899051. doi:10.1177/1177932219899051

2. Gutierrez Reyes CD, Alejo-Jacuinde G, Perez Sanchez B, et al. Multi Omics Applications in Biological Systems. *Curr Issues Mol Biol*. 2024;46(6):5777-5793. doi:10.3390/cimb46060345

3. Hasin Y, Seldin M, Lusis A. Multi-omics approaches to disease. *Genome Biology*. 2017;18(1):83. doi:10.1186/s13059-017-1215-1

4. Singh A, Shannon CP, Gautier B, et al. DIABLO: an integrative approach for identifying key molecular drivers from multi-omics assays. *Bioinformatics*. 2019;35(17):3055-3062. doi:10.1093/bioinformatics/bty1054

5. Wang T, Shao W, Huang Z, et al. MOGONET integrates multi-omics data using graph convolutional networks allowing patient classification and biomarker identification. *Nat Commun*. 2021;12(1):3445. doi:10.1038/s41467-021-23774-w

6. Xiao L, Zhang F, Zhao F. Large-scale microbiome data integration enables robust biomarker identification. *Nat Comput Sci*. 2022;2(5):307-316. doi:10.1038/s43588-022-00247-8

7. Coletti R, Lopes MB. Multi-omics Data Integration and Network Inference for Biomarker Discovery in Glioma. In: Moniz N, Vale Z, Cascalho J, Silva C, Sebastião R, eds. *Progress in Artificial Intelligence*. Springer Nature Switzerland; 2023:247-259. doi:10.1007/978-3-031-49011-8_20

8. Wold H. Estimation of principal components and related models by iterative least squares. Published online 1966. Accessed January 22, 2024. https://www.acemap.info/paper/64195297

9. Chun H, Keleş S. Sparse partial least squares regression for simultaneous dimension reduction and variable selection. *Journal of the Royal Statistical Society: Series B (Statistical Methodology)*. 2010;72(1):3-25. doi:10.1111/j.1467-9868.2009.00723.x

10. Hotelling H. Relations Between Two Sets of Variates. *Biometrika*. 1936;28(3/4):321-377. doi:10.2307/2333955

11. Witten DM, Tibshirani R, Hastie T. A penalized matrix decomposition, with applications to sparse principal components and canonical correlation analysis. *Biostatistics*. 2009;10(3):515-534. doi:10.1093/biostatistics/kxp008

12. Tenenhaus A, Tenenhaus M. Regularized Generalized Canonical Correlation Analysis. *Psychometrika*. 2011;76(2):257-284. doi:10.1007/s11336-011-9206-8

13. Lai PL, Fyfe C. Kernel and nonlinear canonical correlation analysis. *Int J Neur Syst*. 2000;10(05):365-377. doi:10.1142/S012906570000034X

14. Yoshida K, Yoshimoto J, Doya K. Sparse kernel canonical correlation analysis for discovery of nonlinear interactions in high-dimensional data. *BMC Bioinformatics*. 2017;18(1):108. doi:10.1186/s12859-017-1543-x

15. Breiman L. Random Forests. *Machine Learning*. 2001;45(1):5-32. doi:10.1023/A:1010933404324

16. Segal M, Xiao Y. Multivariate random forests. *WIREs Data Mining and Knowledge Discovery*. 2011;1(1):80-87. doi:10.1002/widm.12

17. Tang F, Ishwaran H. Random Forest Missing Data Algorithms. *Stat Anal Data Min*. 2017;10(6):363-377. doi:10.1002/sam.11348

18. Ishwaran H, Kogalur UB, Gorodeski EZ, Minn AJ, Lauer MS. High-Dimensional Variable Selection for Survival Data. *Journal of the American Statistical Association*. 2010;105(489):205-217. doi:10.1198/jasa.2009.tm08622

19. Ishwaran H, Kogalur UB, Chen X, Minn AJ. Random survival forests for high-dimensional data. *Statistical Analysis and Data Mining: The ASA Data Science Journal*. 2011;4(1):115-132. doi:10.1002/sam.10103

20. Dempster AP, Laird NM, Rubin DB. Maximum Likelihood from Incomplete Data Via the EM Algorithm. *Journal of the Royal Statistical Society: Series B (Methodological)*. 1977;39(1):1-22. doi:10.1111/j.2517-6161.1977.tb01600.x

21. Lee G, Scott C. EM algorithms for multivariate Gaussian mixture models with truncated and censored data. *Computational Statistics & Data Analysis*. 2012;56(9):2816-2829. doi:10.1016/j.csda.2012.03.003

22. Degenhardt F, Seifert S, Szymczak S. Evaluation of variable selection methods for random forests and omics data sets. *Briefings in Bioinformatics*. 2019;20(2):492-503. doi:10.1093/bib/bbx124

23. Cancer Genome Atlas Network. Comprehensive molecular portraits of human breast tumours. *Nature*. 2012;490(7418):61-70. doi:10.1038/nature11412

24. Cancer Genome Atlas Network. Comprehensive molecular characterization of human colon and rectal cancer. *Nature*. 2012;487(7407):330-337. doi:10.1038/nature11252

25. Corces MR, Granja JM, Shams S, et al. The chromatin accessibility landscape of primary human cancers. *Science*. 2018;362(6413):eaav1898. doi:10.1126/science.aav1898

26. Goldman MJ, Craft B, Hastie M, et al. Visualizing and interpreting cancer genomics data via the Xena platform. *Nat Biotechnol*. 2020;38(6):675-678. doi:10.1038/s41587-020-0546-8

27. Zhang W, Young JI, Gomez L, et al. Blood DNA methylation signature for incident dementia: Evidence from longitudinal cohorts. *Alzheimer's & Dementia*. 2025;21(3):e14496. doi:10.1002/alz.14496

28. Guo F, Ma J, Li C, et al. PRR15 deficiency facilitates malignant progression by mediating PI3K/Akt signaling and predicts clinical prognosis in triple-negative rather than non-triple-negative breast cancer. *Cell Death Dis*. 2023;14(4):272. doi:10.1038/s41419-023-05746-8

29. Byerly JH, Port ER, Irie HY. PRKCQ inhibition enhances chemosensitivity of triple-negative breast cancer by regulating Bim. *Breast Cancer Research*. 2020;22(1):72. doi:10.1186/s13058-020-01302-w

30. Molière S, Lodi M, Leblanc S, et al. MMP-11 expression in early luminal breast cancer: associations with clinical, MRI, pathological characteristics, and disease-free survival. *BMC Cancer*. 2024;24(1):295. doi:10.1186/s12885-024-11998-0

31. Zhuang Y, Li X, Zhan P, Pi G, Wen G. MMP11 promotes the proliferation and progression of breast cancer through stabilizing Smad2 protein. *Oncol Rep*. 2021;45(4):16. doi:10.3892/or.2021.7967

32. Khaled WT, Choon Lee S, Stingl J, et al. BCL11A is a triple-negative breast cancer gene with critical functions in stem and progenitor cells. *Nat Commun*. 2015;6(1):5987. doi:10.1038/ncomms6987

33. Martin EM, Orlando KA, Yokobori K, Wade PA. The estrogen receptor/GATA3/FOXA1 transcriptional network: lessons learned from breast cancer. *Curr Opin Struct Biol*. 2021;71:65-70. doi:10.1016/j.sbi.2021.05.015

34. Xu J, Xiang L, Liu Q, et al. Stacked Sparse Autoencoder (SSAE) for Nuclei Detection on Breast Cancer Histopathology Images. *IEEE Transactions on Medical Imaging*. 2016;35(1):119-130. doi:10.1109/TMI.2015.2458702

35. Denkiewicz M, Saha I, Rakshit S, Sarkar JP, Plewczynski D. Identification of Breast Cancer Subtype Specific MicroRNAs Using Survival Analysis to Find Their Role in Transcriptomic Regulation. *Front Genet*. 2019;10. doi:10.3389/fgene.2019.01047

36. Qattan A, Intabli H, Alkhayal W, Eltabache C, Tweigieri T, Amer SB. Robust expression of tumor suppressor miRNA's let-7 and miR-195 detected in plasma of Saudi female breast cancer patients. *BMC Cancer*. 2017;17(1):799. doi:10.1186/s12885-017-3776-5

37. Huang D, Yu B, Deng Y, et al. SFRP4 was overexpressed in colorectal carcinoma. *J Cancer Res Clin Oncol*. 2010;136(3):395-401. doi:10.1007/s00432-009-0669-2

38. Nfonsam LE, Jandova J, Jecius HC, Omesiete PN, Nfonsam VN. SFRP4 expression correlates with epithelial mesenchymal transition-linked genes and poor overall survival in colon cancer patients. *World J Gastrointest Oncol*. 2019;11(8):589-598. doi:10.4251/wjgo.v11.i8.589

39. Chang TY, Lan KC, Chiu CY, Sheu ML, Liu SH. ANGPTL1 attenuates cancer migration, invasion, and stemness through regulating FOXO3a-mediated SOX2 expression in colorectal cancer. *Clin Sci (Lond)*. 2022;136(9):657-673. doi:10.1042/CS20220043

40. Koestler DC, Li J, Baron JA, et al. Distinct patterns of DNA methylation in conventional adenomas involving the right and left colon. *Mod Pathol*. 2014;27(1):145-155. doi:10.1038/modpathol.2013.104

41. Hu W, Yang Y, Li X, et al. Multi-omics Approach Reveals Distinct Differences in Left- and Right-Sided Colon Cancer. *Molecular Cancer Research*. 2018;16(3):476-485. doi:10.1158/1541-7786.MCR-17-0483

42. Chen P, Xi Q, Wang Q, Wei P. Downregulation of microRNA-100 correlates with tumor progression and poor prognosis in colorectal cancer. *Med Oncol*. 2014;31(10):235. doi:10.1007/s12032-014-0235-x

43. Hibner G, Kimsa-Furdzik M, Francuz T. Relevance of MicroRNAs as Potential Diagnostic and Prognostic Markers in Colorectal Cancer. *International Journal of Molecular Sciences*. 2018;19(10):2944. doi:10.3390/ijms19102944

44. Liberzon A, Subramanian A, Pinchback R, Thorvaldsdóttir H, Tamayo P, Mesirov JP. Molecular signatures database (MSigDB) 3.0. *Bioinformatics*. 2011;27(12):1739-1740. doi:10.1093/bioinformatics/btr260

45. Paplomata E, O'Regan R. The PI3K/AKT/mTOR pathway in breast cancer: targets, trials and biomarkers. *Ther Adv Med Oncol*. 2014;6(4):154-166. doi:10.1177/1758834014530023

46. Gil EMC. Targeting the PI3K/AKT/mTOR pathway in estrogen receptor-positive breast cancer. *Cancer Treatment Reviews*. 2014;40(7):862-871. doi:10.1016/j.ctrv.2014.03.004

47. Wiseman BS, Werb Z. Stromal Effects on Mammary Gland Development and Breast Cancer. *Science*. 2002;296(5570):1046-1049. doi:10.1126/science.1067431

48. Dongre A, Weinberg RA. New insights into the mechanisms of epithelial–mesenchymal transition and implications for cancer. *Nat Rev Mol Cell Biol*. 2019;20(2):69-84. doi:10.1038/s41580-018-0080-4

49. Nenkov M, Ma Y, Gaßler N, Chen Y. Metabolic Reprogramming of Colorectal Cancer Cells and the Microenvironment: Implication for Therapy. *Int J Mol Sci*. 2021;22(12):6262. doi:10.3390/ijms22126262

50. Chalise P, Fridley BL. Integrative clustering of multi-level 'omic data based on non-negative matrix factorization algorithm. *PLoS One*. 2017;12(5):e0176278. doi:10.1371/journal.pone.0176278

51. Choi SB, Kwon S, Kim JH, Ahn NH, Lee JH, Yang SH. The Molecular Mechanisms of Neuroinflammation in Alzheimer's Disease, the Consequence of Neural Cell Death. *Int J Mol Sci*. 2023;24(14):11757. doi:10.3390/ijms241411757

52. Zhu Z, Zhang L, Elsherbini A, et al. The S1P receptor 1 antagonist Ponesimod reduces TLR4-induced neuroinflammation and increases Aβ clearance in 5XFAD mice. *eBioMedicine*. 2023;94. doi:10.1016/j.ebiom.2023.104713

53. Zhang T, Xia Y, Hu L, et al. Death-associated protein kinase 1 mediates Aβ42 aggregation-induced neuronal apoptosis and tau dysregulation in Alzheimer's disease. *Int J Biol Sci*. 2022;18(2):693-706. doi:10.7150/ijbs.66760

54. Da Mesquita S, Herz J, Wall M, et al. Aging-associated deficit in CCR7 is linked to worsened glymphatic function, cognition, neuroinflammation, and β-amyloid pathology. *Sci Adv*. 2021;7(21):eabe4601. doi:10.1126/sciadv.abe4601

55. Dai L, Shen Y. Insights into T-cell dysfunction in Alzheimer's disease. *Aging Cell*. 2021;20(12):e13511. doi:10.1111/acel.13511

56. Fehervari Z. Lymphocytes in Alzheimer's disease. *Nat Immunol*. 2016;17(4):355-355. doi:10.1038/ni.3427

57. Lu J, Kornmann M, Traub B. Role of Epithelial to Mesenchymal Transition in Colorectal Cancer. *Int J Mol Sci*. 2023;24(19):14815. doi:10.3390/ijms241914815

58. Levine DA. Integrated genomic characterization of endometrial carcinoma. *Nature*. 2013;497(7447):67-73. doi:10.1038/nature12113

TABLES

Table 1: Simulation model scenarios for integration variable selection comparison.

(a) Latent model

**Scenario**	**S1**	**S2**	**S3**
**Sample size**	100	200	200
**Dimension**	200	500	1000
**True model size**			
Two-omics	20	30	50
Three-omics			

(b) Non-linear regression model

**Scenario**	**S1**	**S2**	**S3**
**Sample size**	100	200	200
**Dimension**	200	500	1000
**True model size**			
Setting 1	20/5		
Setting 2	40/10		

Table 2: Summary of datasets.

Dataset	Number of arrays	Number of arrays for training	Number of samples	Number of arrays selected by MRF-IMD
TCGA-	*mRNA, DNAm, miRNA*			
BRCA	20530, 485577, 2238	2000, 2000, 228	674	102, 141, 22
COAD	20530, 485577, 2113	2000, 2000, 252	257	139, 107, 18
TCGA-	*ATAC-Seq, RNA-Seq*			
Pan-Cancer	562709, 59390	50000, 5000	383	186, 300
			TCGA-ACC: 9; TCGA-BLCA: 9; TCGA-BRCA: 72; TCGA-CESC: 2; TCGA-CHOL: 2; TCGA-COAD: 37; TCGA-ESCA: 18; TCGA-HNSC: 9; TCGA-KIRC: 16; TCGA-KIRP: 32; TCGA-LGG: 12; TCGA-LIHC: 17; TCGA-LUAD: 21; TCGA-LUSC: 16; TCGA-MESO: 7; TCGA-PCPG: 9; TCGA-PRAD:	

			26; TCGA-SKCM: 13; TCGA-STAD: 21; TCGA-TGCT: 9; TCGA-THCA: 14; TCGA-UCEC: 12.	
ADNI	*<u>Gene expression, DNAm</u>*<br>49395, 734743	2000, 2000	Total: 468<br>CN: 198, MCI: 288	161, 54

Table 3: Simulation results. Performance measures are the mean of PR-AUC, precision, recall, and model size (standard deviations).

(a) Latent model

Model	Scenario	Selection Method	Setting							
			Two Omics				Three Omics			
			PR-AUC	Precision	Recall	Model Size	PR-AUC	Precision	Recall	Model Size
MRF-IMD	S1	Filter	0.90 (0.04)	0.87 (0.09)	0.88 (0.06)	41.36 (6.33)	0.82 (0.05)	0.86 (0.14)	0.79 (0.07)	57.78 (18.41)
		Mixture	0.90 (0.04)	0.86 (0.06)	0.88 (0.05)	41.08 (4.25)	0.85 (0.04)	0.87 (0.06)	0.82 (0.04)	57.16 (4.80)
		Trans	0.91 (0.03)	0.87 (0.06)	0.89 (0.04)	41.10 (3.48)	0.86 (0.04)	0.77 (0.08)	0.84 (0.04)	65.96 (7.28)
	S2	Filter	0.89 (0.05)	0.94 (0.05)	0.87 (0.06)	55.50 (5.65)	0.82 (0.04)	0.91 (0.12)	0.79 (0.05)	81.14 (23.95)
		Mixture	0.90 (0.04)	0.93 (0.04)	0.89 (0.04)	57.74 (3.44)	0.85 (0.03)	0.92 (0.04)	0.83 (0.04)	81.30 (4.77)
		Trans	0.92 (0.03)	0.88 (0.05)	0.91 (0.04)	62.54 (4.38)	0.87 (0.03)	0.82 (0.06)	0.85 (0.04)	94.44 (7.57)
	S3	Filter	0.83 (0.04)	0.94 (0.04)	0.81 (0.05)	87.14 (8.82)	0.77 (0.05)	0.91 (0.12)	0.74 (0.06)	126.28 (34.31)
		Mixture	0.86 (0.03)	0.90 (0.03)	0.84 (0.04)	94.32 (5.80)	0.81 (0.04)	0.92 (0.04)	0.79 (0.04)	128.62 (8.21)
		Trans	0.88 (0.03)	0.87 (0.04)	0.87 (0.03)	99.80 (6.60)	0.82 (0.03)	0.80 (0.06)	0.81 (0.04)	152.40 (13.99)
Other Benchmarks	S1	PMDCCA	0.90 (0.09)	0.95 (0.10)	0.89 (0.06)	37.48 (3.22)	0.76 (0.15)	0.77 (0.13)	0.79 (0.10)	62.54 (5.51)
		RGCCA	0.69 (0.08)	0.98 (0.08)	0.62 (0.06)	25.40 (2.70)	0.66 (0.03)	1.00 (0.00)	0.57 (0.04)	34.24 (2.25)
		SPLS	0.94 (0.10)	0.93 (0.07)	0.93 (0.07)	40.00 (0.00)	0.79 (0.16)	0.82 (0.11)	0.82 (0.11)	60.00 (0.00)
	S2	PMDCCA	0.76 (0.04)	1.00 (0.00)	0.72 (0.04)	43.04 (2.64)	0.67 (0.12)	0.90 (0.12)	0.65 (0.08)	65.52 (4.44)
		RGCCA	0.84 (0.03)	1.00 (0.00)	0.81 (0.04)	48.46 (2.13)	0.82 (0.02)	0.98 (0.04)	0.79 (0.03)	72.68 (4.46)
		SPLS	0.97 (0.02)	0.97 (0.02)	0.97 (0.02)	60.00 (0.00)	0.82 (0.17)	0.83 (0.12)	0.83 (0.12)	90.00 (0.00)
	S3	PMDCCA	0.57 (0.04)	1.00 (0.00)	0.51 (0.05)	51.08 (4.51)	0.50 (0.09)	0.93 (0.12)	0.46 (0.06)	74.84 (5.24)

		RGCCA	0.91 (0.01)	0.99 (0.02)	0.89 (0.02)	90.36 (2.77)	0.88 (0.01)	0.82 (0.05)	0.87 (0.01)	159.36 (8.33)
		SPLS	0.97 (0.01)	0.96 (0.02)	0.96 (0.02)	100.00 (0.00)	0.83 (0.10)	0.83 (0.07)	0.83 (0.07)	150.00 (0.00)

(b) Non-linear regression model

Model	Scenario	Selection Method	Setting							
			Setting 1				Setting 2			
			PR-AUC	Precision	Recall	Model Size	PR-AUC	Precision	Recall	Model Size
MRF-IMD	S1	Filter	0.71 (0.07)	0.77 (0.30)	0.73 (0.11)	73.06 (134.66)	0.68 (0.05)	0.92 (0.06)	0.66 (0.06)	36.02 (4.61)
		Mixture	0.70 (0.07)	0.82 (0.09)	0.70 (0.07)	21.54 (2.87)	0.67 (0.04)	0.90 (0.05)	0.65 (0.04)	36.20 (3.14)
		Trans	0.76 (0.06)	0.48 (0.06)	0.79 (0.06)	41.72 (3.99)	0.71 (0.04)	0.70 (0.06)	0.70 (0.04)	50.72 (3.73)
	S2	Filter	0.81 (0.07)	0.96 (0.05)	0.80 (0.07)	20.88 (2.47)	0.79 (0.05)	0.98 (0.02)	0.75 (0.06)	38.46 (3.35)
		Mixture	0.83 (0.06)	0.94 (0.05)	0.81 (0.06)	21.58 (2.12)	0.78 (0.04)	0.99 (0.02)	0.74 (0.05)	37.64 (2.43)
		Trans	0.87 (0.05)	0.68 (0.07)	0.87 (0.05)	32.00 (3.10)	0.80 (0.03)	0.91 (0.05)	0.77 (0.04)	42.48 (2.71)
	S3	Filter	0.81 (0.07)	0.92 (0.08)	0.78 (0.08)	21.34 (3.96)	0.80 (0.06)	0.95 (0.05)	0.73 (0.08)	38.56 (5.42)
		Mixture	0.80 (0.06)	0.92 (0.05)	0.77 (0.08)	20.86 (2.60)	0.76 (0.06)	0.96 (0.03)	0.68 (0.07)	35.30 (4.21)
		Trans	0.81 (0.05)	0.77 (0.06)	0.78 (0.06)	25.56 (2.06)	0.73 (0.04)	0.95 (0.04)	0.64 (0.05)	33.96 (2.69)
Other Benchmarks	S1	PMDCCA	0.12 (0.11)	0.03 (0.03)	0.15 (0.15)	107.08 (45.68)	0.13 (0.06)	0.05 (0.03)	0.15 (0.07)	131.40 (34.01)
		RGCCA	0.15 (0.12)	0.02 (0.02)	0.23 (0.17)	226.58 (56.22)	0.15 (0.05)	0.04 (0.02)	0.22 (0.08)	260.08 (25.79)
		SPLS	0.04 (0.08)	0.09 (0.21)	0.03 (0.07)	9.00 (0.00)	0.06 (0.05)	0.13 (0.17)	0.04 (0.05)	14.00 (0.00)
	S2	PMDCCA	0.22 (0.12)	0.05 (0.03)	0.27 (0.16)	124.66 (33.19)	0.21 (0.06)	0.09 (0.03)	0.24 (0.08)	134.76 (11.47)
		RGCCA	0.22 (0.14)	0.04 (0.03)	0.26 (0.17)	137.80 (39.43)	0.22 (0.08)	0.09 (0.04)	0.26 (0.09)	149.80 (22.49)

		SPLS	0.07 (0.09)	0.14 (0.23)	0.05 (0.08)	9.00 (0.00)	0.13 (0.05)	0.27 (0.16)	0.08 (0.04)	14.00 (0.00)
	S3	PMDCCA	0.31 (0.12)	0.11 (0.03)	0.39 (0.12)	88.48 (12.20)	0.31 (0.06)	0.19 (0.05)	0.33 (0.08)	87.24 (8.20)
		RGCCA	0.27 (0.11)	0.14 (0.06)	0.28 (0.12)	48.86 (10.07)	0.26 (0.06)	0.21 (0.08)	0.20 (0.06)	49.28 (8.49)
		SPLS	0.20 (0.10)	0.39 (0.26)	0.14 (0.09)	9.00 (0.00)	0.21 (0.04)	0.32 (0.15)	0.09 (0.04)	14.00 (0.00)

Table 4 TCGA-BRCA and COAD prognosis results

TCGA-BRCA			TCGA-COAD		
**Methods**		**(Median) Logrank P-value**	**Methods**		**(Median) Logrank P-value**
All Variables		2.75E-01	All Variables		2.43E-01
SPLS	All 5 components	8.21E-03	SPLS	All 5 components	1.31E-02
	Selected variables from first component	6.50E-02		Selected variables from first component	9.86E-01
PMDCCA	All 5 components	1.43E-02	PMDCCA	All 5 components	9.89E-01
	Selected variables from first component	6.15E-01		Selected variables from first component	2.99E-02
RGCCA	All 5 components	2.59E-02	RGCCA	All 5 components	8.73E-01
	Selected variables from first component	2.18E-02		Selected variables from first component	5.34E-02
MRF-IMD	filter	7.94E-04	MRF-IMD	filter	1.23E-02
	mixture	1.02E-03		mixture	1.38E-02
	test	6.17E-02		test	2.14E-02

Table 5 Pan-cancer clustering annotations

Group	Cluster Name (Abbr.)	TCGA Cohorts	Key Features
1	Basal-like Breast & UCEC (BRCA–UCEC)	BRCA, UCEC	High genomic instability; frequent TP53 and BRCA1/2 alterations; dysregulated DNA repair and cell-cycle pathways.
2	Hepatobiliary Carcinomas (HBC)	LIHC, CHOL	Hepatic lineage tumors with altered metabolic programs and frequent TP53 mutations; cholangiocarcinoma-like epigenetic patterns.
3	Hypermutated/Immunogenic Tumors (HIM)	BLCA, SKCM	Extremely high mutational burden; strong immune-infiltration signatures; enriched for PD-L1 expression and antigen-presentation machinery.
4	Non-basal Breast Cancer (Non-Basal BRCA)	BRCA	Luminal and HER2-positive subtypes; hormone-receptor signaling; PI3K/mTOR pathway activation and endocrine-therapy response markers.
5	Gastrointestinal Adenocarcinoma CIN (GA-CIN)	COAD, STAD, ESCA	Marked chromosomal instability (CIN); Wnt/β-catenin and TGF-β pathway dysregulation; common APC and TP53 alterations.
6	Endocrine Tumors (ENDO)	ACC, PCPG	Hormone-secreting neoplasms of adrenal cortex and chromaffin cells; endocrine-axis gene dysregulation (e.g., steroidogenesis, catecholamine synthesis).
7	Squamous Cell Carcinomas (SCC)	HNSC, LUSC, ESCA, BLCA	Squamous histology tumors; frequent TP53 mutations; activation of RTK-RAS and PI3K pathways; strong epithelial-to-mesenchymal transition signatures.
8	Renal Epithelial Carcinomas (REC)	KIRC, KIRP	Clear-cell and papillary carcinomas; VHL/HIF pathway alterations; characteristic metabolic reprogramming (glycolysis, lipid metabolism).

Table 6 Results from Cox regression model evaluating the association between IntNMF-derived component using MRF-IMD prioritized variable and disease progression (CN to MCI/dementia, MCI to dementia) in 538 subjects, adjusted for age, sex, APOE ε4 status, baseline diagnosis, MMSE, and education using ADNI dataset. Significant association was observed for IntNMF-derived component using MRF-IMD prioritized variables (estimate = 0.201, P-value = 0.034), indicating higher IntNMF-derived component increases risk.

**Characteristic**	**Coefficient**	**HR (95% CI)**	***P*-value**
**IntNMF-derived component**	0.201	1.222 (1.016,1.471)	0.034
**Age, years**	0.063	1.071 (1.037,1.093)	3.16×10^{-6}
**Male**	0.178	1.195 (0.834,1.714)	0.332
**Baseline diagnosis**			
CN		1 [Reference]	
MCI	0.423	1.527 (1.041,2.238)	0.03
***APOE* ε4 allele**	0.647	1.910 (1.472,2.478)	1.13×10^{-6}
**MMSE**	-0.143	0.866 (0.772, 0.972)	0.015
**Education, years**	-0.054	0.947 (0.887,1.012)	0.105

FIGURES

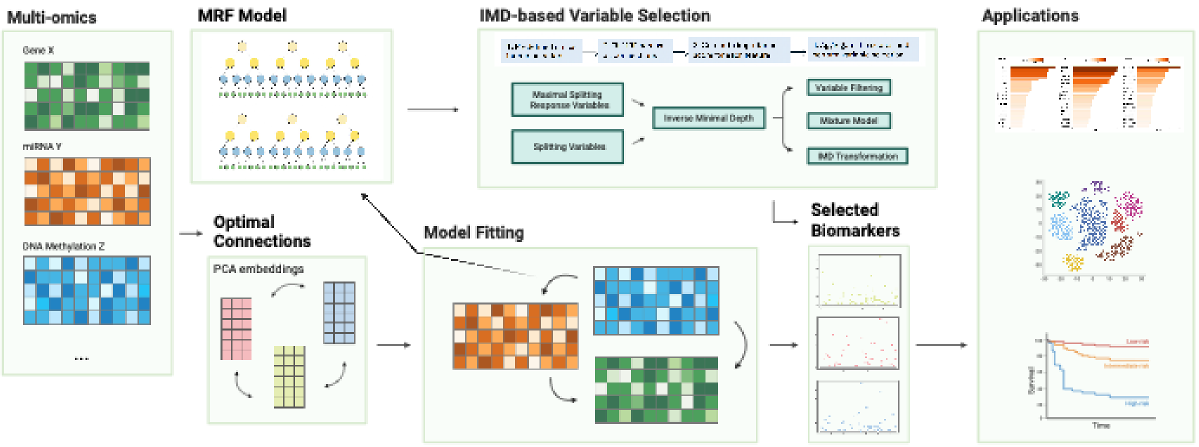

Figure 1 Workflow of MRF-IMD framework. The overall workflow of our integrative multi-omics biomarker discovery pipeline using the Multivariate Random Forest with Inverse Minimal Depth (MRF-IMD) strategy.

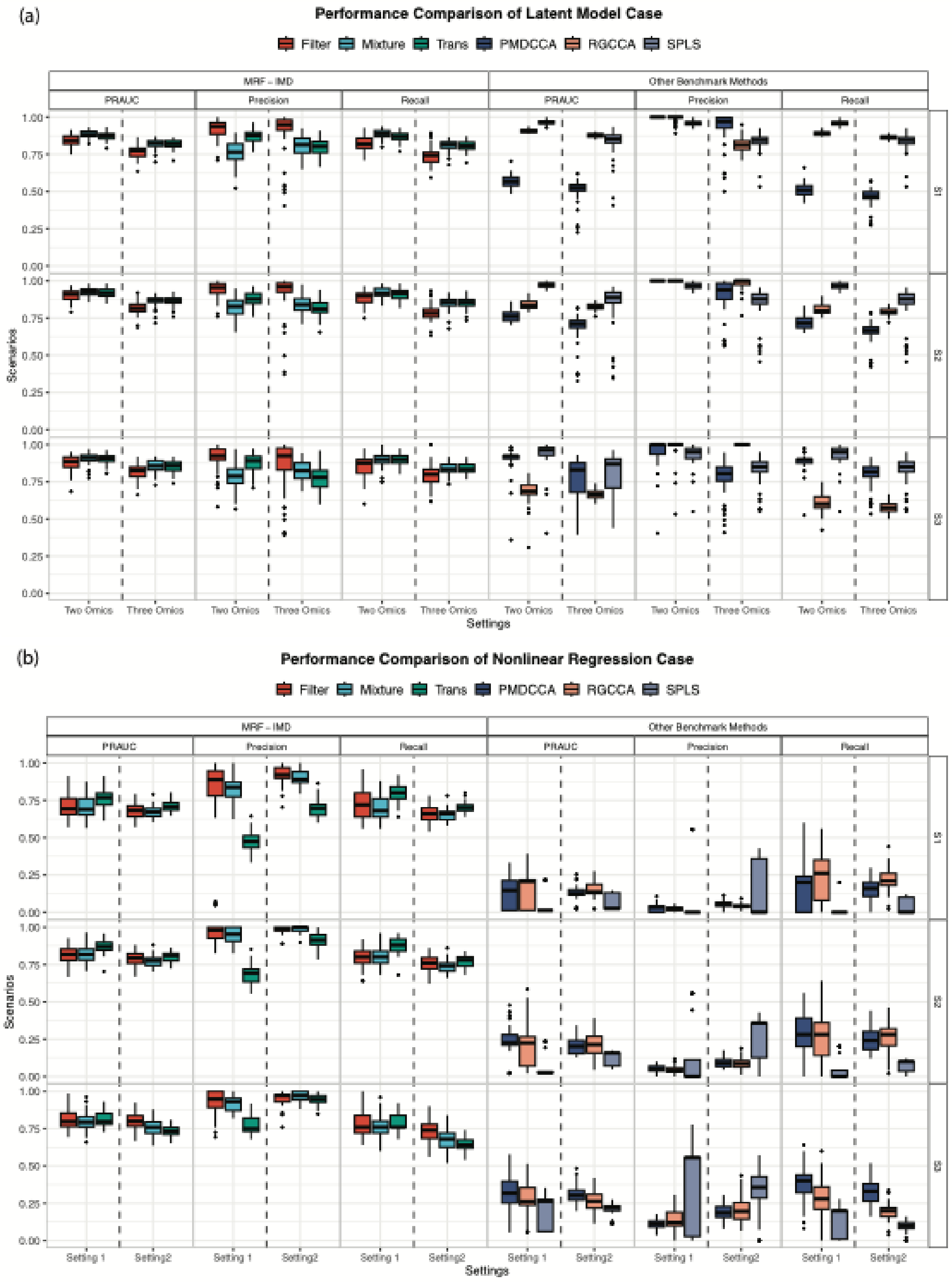

Figure 2 Simulation results. Boxplots of simulation results. Performance measures are PR-AUC, precision, and recall. In most scenarios, the MRF-IMD methods showed competitive selection outcomes in both (a) Latent model and (b) Non-linear regression model.

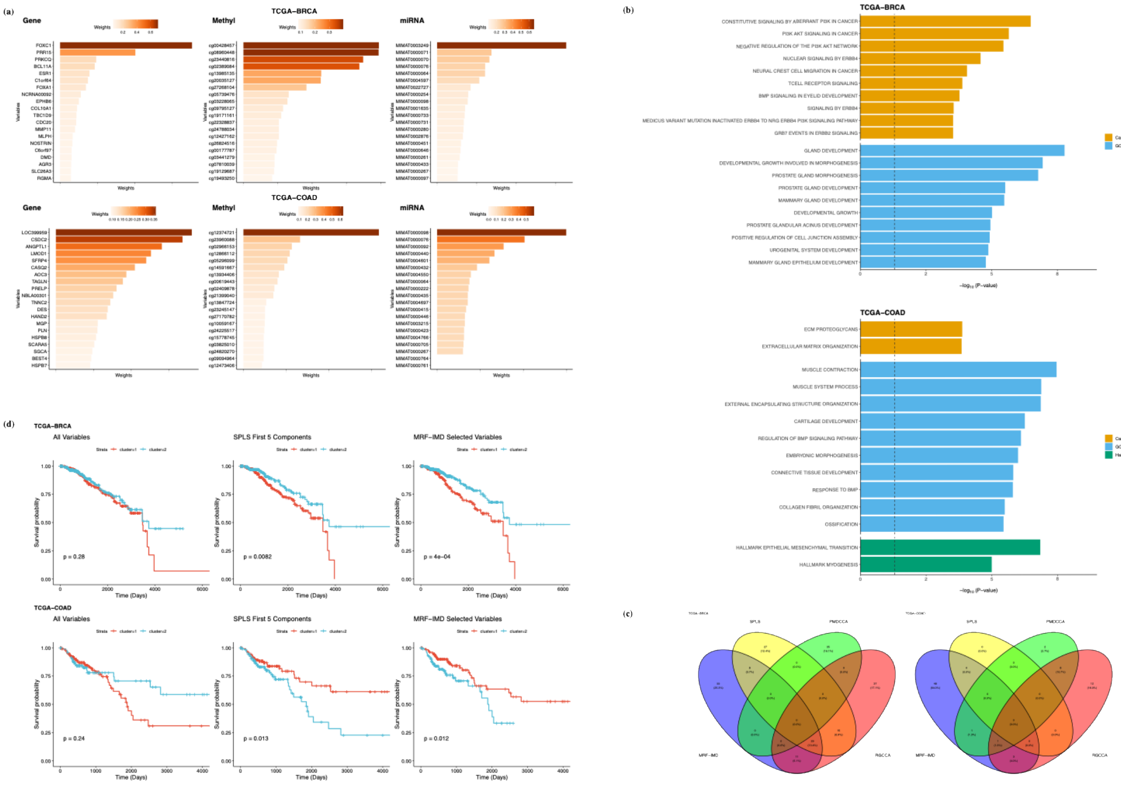

Figure 3 TCGA-BRCA and TCGA-COAD analysis results. (a) Top 20 variable weights chosen by one IMD-mixture model; (b) Functional enrichment analysis to the selected genes with one IMD-filter model performed using clusterProfiler R package. Top 10 significant pathways of C2:CP, GO and Hallmark (i.e. FDR < 0.05) were displayed for both TCGA-BRCA and TCGA-COAD; (c) Four-way Venn diagrams of the significant pathways selected by MRF-IMD, SPLS, PMDCCA, and RGCCA in BRCA and COAD; (d) Kaplan–Meier curves of the patient stratification results from all variables, SPLS 5 components, and the selected MRF-IMD-mixture methods.

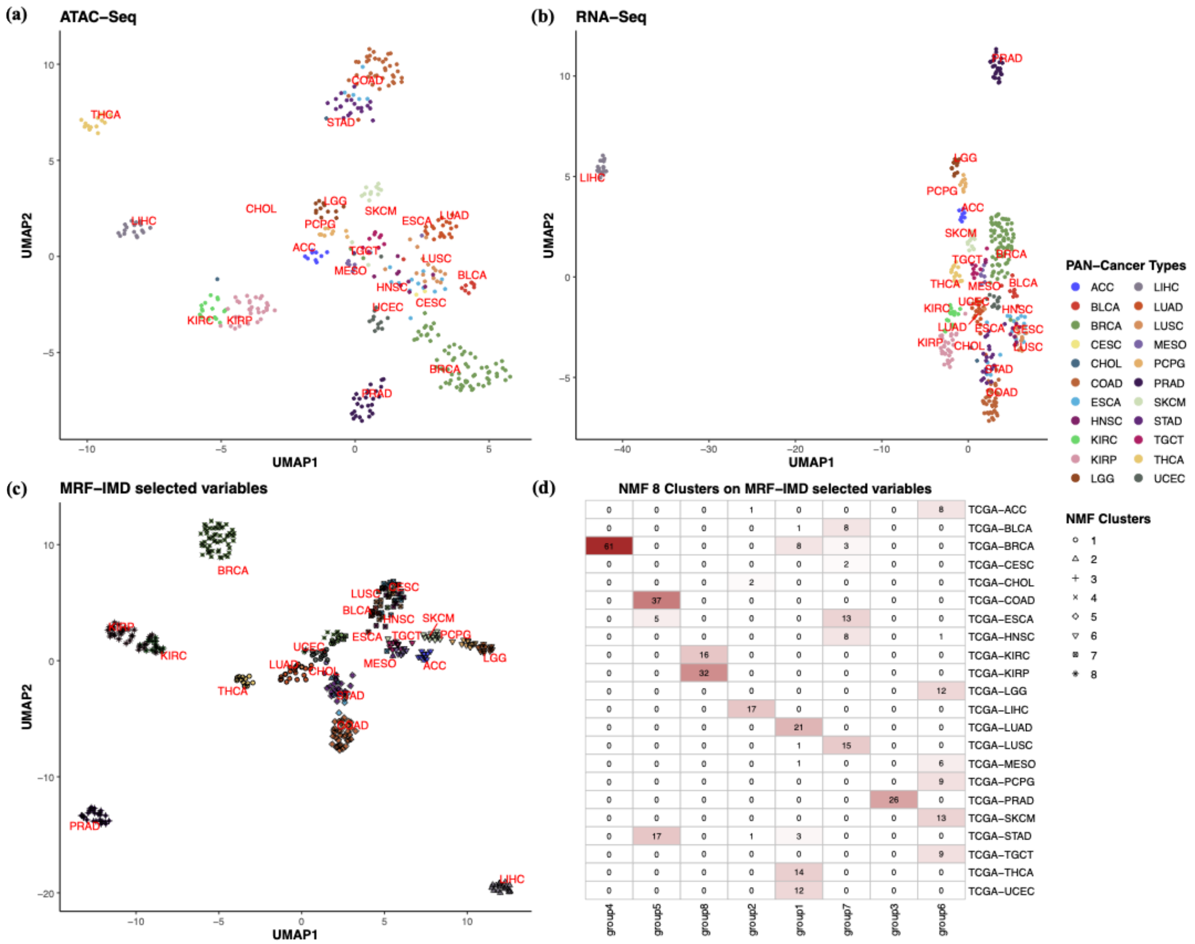

Figure 4 Two-dimensional UMAP embedding of TCGA PAN cancer data and confusion matrix. (a) and (b) are UMAP of 5,000 RNA-Seq and 50,000 ATAC-Seq features; (c) UMAP of IntNMF embeddings of 300 RNA-Seq and 186 ATAC-Seq MRF-IMD prioritized features, colored by 22 PAN cancer types and shaped by the eight IntNMF clusters; (d) Confusion matrix of eight IntNMF clusters and 22 PAN cancer types.

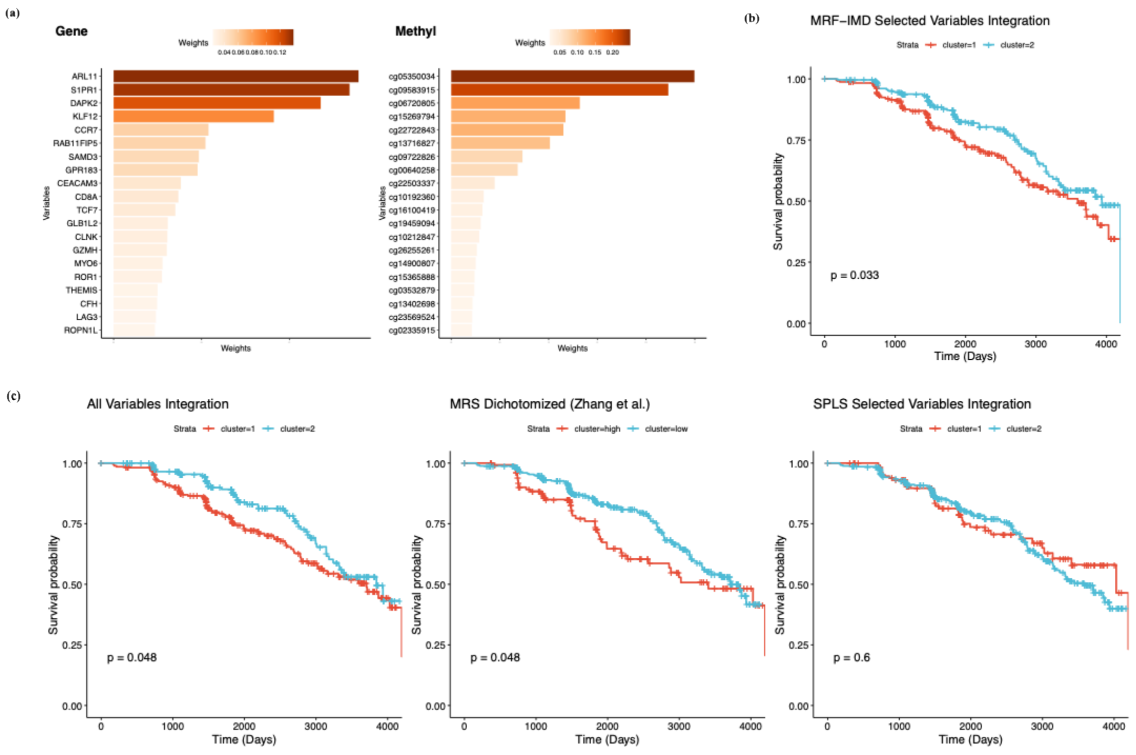

Figure 5 ADNI results. (a) Top 20 variable weights chosen by IMD-mixture model; (b) stratification of Kaplan–Meier curves of MRF-IMD selected variables using IntNMF; (c) stratification of the full omics set integration using IntNMF (left), MRS dichotomized, cut off determined by MaxStat method (middle), and the first five SPLS components from the full omics set using IntNMF (right).

Click here to access/download;Table;Table 1 - simulation settings.xlsx

Table 1: Simulation model scenarios.

(a) Latent model

<table>
<tr><th>Scenario</th><th>S1</th><th>S2</th><th>S3</th></tr>
<tr><td>Sample size</td><td>100</td><td>200</td><td>200</td></tr>
<tr><td>Dimension</td><td>200</td><td>500</td><td>1000</td></tr>
<tr><td>True model size</td><td></td><td></td><td></td></tr>
<tr><td>Two-omics</td><td rowspan="2">20</td><td rowspan="2">30</td><td rowspan="2">50</td></tr>
<tr><td>Three-omics</td></tr>
</table>

(b) Non-linear reg

Scenario
**Sample size**
**Dimension**
**True model size**
Setting 1
Setting 2

gression model

S1	S2	S3
100	200	200
200	500	1000
	20/5	
	40/10	

Click here to access/download;Table;Table 2 - cohorts summary.xlsx

Table 2: Summary of datasets

Dataset	Number of features	Number of features for training
TCGA-	*mRNA, DNAm, miRNA*	
BRCA	20530, 485577, 2238	2000, 2000, 228
COAD	20530, 485577, 2113	2000, 2000, 252
TCGA-	*ATAC-Seq, RNA-Seq*	
Pan-Cancer	562709, 59390	50000, 5000
ADNI	*Gene expression, DNAm*	
	49395, 734743	2000, 2000

Number of samples	Number of arrays selected by MRF-IMD
674<br>257	102, 141, 22<br>139, 107, 18
383<br><br>TCGA-ACC: 9; TCGA-BLCA: 9; TCGA-BRCA: 72; TCGA-CESC: 2; TCGA-CHOL: 2; TCGA-COAD: 37; TCGA-ESCA: 18; TCGA-HNSC: 9; TCGA-KIRC: 16; TCGA-KIRP: 32; TCGA-LGG: 12; TCGA-LIHC: 17; TCGA-LUAD: 21; TCGA-LUSC: 16; TCGA-MESO: 7; TCGA-PCPG: 9; TCGA-PRAD: 26; TCGA-SKCM: 13; TCGA-STAD: 21; TCGA-TGCT: 9; TCGA-THCA: 14; TCGA-UCEC: 12.	186, 300
Total: 468<br>CN: 198, MCI: 288	161, 54

 Click here to access/download;Table;Table 3 - simulation results.xlsx

Table 3: Simulation results of integrative variable selection. Performance

(a) Latent model

Model	Scenario	Selection Method	PR-AUC
MRF-IMD	S1	Filter	0.90 (0.04)
		Mixture	0.90 (0.04)
		Trans	0.91 (0.03)
	S2	Filter	0.89 (0.05)
		Mixture	0.90 (0.04)
		Trans	0.92 (0.03)
	S3	Filter	0.83 (0.04)
		Mixture	0.86 (0.03)
		Trans	0.88 (0.03)
Other Benchmarks	S1	PMDCCA	0.90 (0.09)
		RGCCA	0.69 (0.08)
		SPLS	0.94 (0.10)
	S2	PMDCCA	0.76 (0.04)
		RGCCA	0.84 (0.03)
		SPLS	0.97 (0.02)
	S3	PMDCCA	0.57 (0.04)
		RGCCA	0.91 (0.01)
		SPLS	0.97 (0.01)

(b) Non-linear regression model

Model	Scenario	Selection Method	PR-AUC
MRF-IMD	S1	Filter	0.71 (0.07)
		Mixture	0.70 (0.07)
		Trans	0.76 (0.06)
	S2	Filter	0.81 (0.07)
		Mixture	0.83 (0.06)
		Trans	0.87 (0.05)
	S3	Filter	0.81 (0.07)
		Mixture	0.80 (0.06)
		Trans	0.81 (0.05)
Other Benchmarks	S1	PMDCCA	0.12 (0.11)
		RGCCA	0.15 (0.12)
		SPLS	0.04 (0.08)
	S2	PMDCCA	0.22 (0.12)
		RGCCA	0.22 (0.14)
		SPLS	0.07 (0.09)
	S3	PMDCCA	0.31 (0.12)
		RGCCA	0.27 (0.11)

		SPLS	0.20 (0.10)

measures are the mean of PR-AUC, precision, recall, and model size (standard deviatior

Setting				
Two Omics			Three	
Precision	Recall	Model Size	PR-AUC	Precision
0.87 (0.09)	0.88 (0.06)	41.36 (6.33)	0.82 (0.05)	0.86 (0.14)
0.86 (0.06)	0.88 (0.05)	41.08 (4.25)	0.85 (0.04)	0.87 (0.06)
0.87 (0.06)	0.89 (0.04)	41.10 (3.48)	0.86 (0.04)	0.77 (0.08)
0.94 (0.05)	0.87 (0.06)	55.50 (5.65)	0.82 (0.04)	0.91 (0.12)
0.93 (0.04)	0.89 (0.04)	57.74 (3.44)	0.85 (0.03)	0.92 (0.04)
0.88 (0.05)	0.91 (0.04)	62.54 (4.38)	0.87 (0.03)	0.82 (0.06)
0.94 (0.04)	0.81 (0.05)	87.14 (8.82)	0.77 (0.05)	0.91 (0.12)
0.90 (0.03)	0.84 (0.04)	94.32 (5.80)	0.81 (0.04)	0.92 (0.04)
0.87 (0.04)	0.87 (0.03)	99.80 (6.60)	0.82 (0.03)	0.80 (0.06)
0.95 (0.10)	0.89 (0.06)	37.48 (3.22)	0.76 (0.15)	0.77 (0.13)
0.98 (0.08)	0.62 (0.06)	25.40 (2.70)	0.66 (0.03)	1.00 (0.00)
0.93 (0.07)	0.93 (0.07)	40.00 (0.00)	0.79 (0.16)	0.82 (0.11)
1.00 (0.00)	0.72 (0.04)	43.04 (2.64)	0.67 (0.12)	0.90 (0.12)
1.00 (0.00)	0.81 (0.04)	48.46 (2.13)	0.82 (0.02)	0.98 (0.04)
0.97 (0.02)	0.97 (0.02)	60.00 (0.00)	0.82 (0.17)	0.83 (0.12)
1.00 (0.00)	0.51 (0.05)	51.08 (4.51)	0.50 (0.09)	0.93 (0.12)
0.99 (0.02)	0.89 (0.02)	90.36 (2.77)	0.88 (0.01)	0.82 (0.05)
0.96 (0.02)	0.96 (0.02)	100.00 (0.00)	0.83 (0.10)	0.83 (0.07)

Setting				
Setting 1			Set	
Precision	Recall	Model Size	PR-AUC	Precision
0.77 (0.30)	0.73 (0.11)	73.06 (134.66)	0.68 (0.05)	0.92 (0.06)
0.82 (0.09)	0.70 (0.07)	21.54 (2.87)	0.67 (0.04)	0.90 (0.05)
0.48 (0.06)	0.79 (0.06)	41.72 (3.99)	0.71 (0.04)	0.70 (0.06)
0.96 (0.05)	0.80 (0.07)	20.88 (2.47)	0.79 (0.05)	0.98 (0.02)
0.94 (0.05)	0.81 (0.06)	21.58 (2.12)	0.78 (0.04)	0.99 (0.02)
0.68 (0.07)	0.87 (0.05)	32.00 (3.10)	0.80 (0.03)	0.91 (0.05)
0.92 (0.08)	0.78 (0.08)	21.34 (3.96)	0.80 (0.06)	0.95 (0.05)
0.92 (0.05)	0.77 (0.08)	20.86 (2.60)	0.76 (0.06)	0.96 (0.03)
0.77 (0.06)	0.78 (0.06)	25.56 (2.06)	0.73 (0.04)	0.95 (0.04)
0.03 (0.03)	0.15 (0.15)	107.08 (45.68)	0.13 (0.06)	0.05 (0.03)
0.02 (0.02)	0.23 (0.17)	226.58 (56.22)	0.15 (0.05)	0.04 (0.02)
0.09 (0.21)	0.03 (0.07)	9.00 (0.00)	0.06 (0.05)	0.13 (0.17)
0.05 (0.03)	0.27 (0.16)	124.66 (33.19)	0.21 (0.06)	0.09 (0.03)
0.04 (0.03)	0.26 (0.17)	137.80 (39.43)	0.22 (0.08)	0.09 (0.04)
0.14 (0.23)	0.05 (0.08)	9.00 (0.00)	0.13 (0.05)	0.27 (0.16)
0.11 (0.03)	0.39 (0.12)	88.48 (12.20)	0.31 (0.06)	0.19 (0.05)
0.14 (0.06)	0.28 (0.12)	48.86 (10.07)	0.26 (0.06)	0.21 (0.08)

0.39 (0.26)	0.14 (0.09)	9.00 (0.00)	0.21 (0.04)	0.32 (0.15)

ns).

e Omics	
Recall	Model Size
0.79 (0.07)	57.78 (18.41)
0.82 (0.04)	57.16 (4.80)
0.84 (0.04)	65.96 (7.28)
0.79 (0.05)	81.14 (23.95)
0.83 (0.04)	81.30 (4.77)
0.85 (0.04)	94.44 (7.57)
0.74 (0.06)	126.28 (34.31)
0.79 (0.04)	128.62 (8.21)
0.81 (0.04)	152.40 (13.99)
0.79 (0.10)	62.54 (5.51)
0.57 (0.04)	34.24 (2.25)
0.82 (0.11)	60.00 (0.00)
0.65 (0.08)	65.52 (4.44)
0.79 (0.03)	72.68 (4.46)
0.83 (0.12)	90.00 (0.00)
0.46 (0.06)	74.84 (5.24)
0.87 (0.01)	159.36 (8.33)
0.83 (0.07)	150.00 (0.00)

tting 2	
Recall	Model Size
0.66 (0.06)	36.02 (4.61)
0.65 (0.04)	36.20 (3.14)
0.70 (0.04)	50.72 (3.73)
0.75 (0.06)	38.46 (3.35)
0.74 (0.05)	37.64 (2.43)
0.77 (0.04)	42.48 (2.71)
0.73 (0.08)	38.56 (5.42)
0.68 (0.07)	35.30 (4.21)
0.64 (0.05)	33.96 (2.69)
0.15 (0.07)	131.40 (34.01)
0.22 (0.08)	260.08 (25.79)
0.04 (0.05)	14.00 (0.00)
0.24 (0.08)	134.76 (11.47)
0.26 (0.09)	149.80 (22.49)
0.08 (0.04)	14.00 (0.00)
0.33 (0.08)	87.24 (8.20)
0.20 (0.06)	49.28 (8.49)

0.09 (0.04)	14.00 (0.00)

Click here to access/download;Table;Table 4 - BRCA COAD prognosis results.xlsx

Table 4 TCGA-BRCA and COAD prognosis results

TCGA-BRCA		
**Methods**		**(Median) Logrank P-value**
All Variables		2.75E-01
SPLS	All 5 components	8.21E-03
	Selected variables from first component	6.50E-02
PMDCCA	All 5 components	1.43E-02
	Selected variables from first component	6.15E-01
RGCCA	All 5 components	2.59E-02
	Selected variables from first component	2.18E-02
MRF-IMD	filter	7.94E-04
	mixture	1.02E-03
	test	6.17E-02

TCGA-COAD		
**Methods**		**(Median) Logrank P-value**
All Variables		2.43E-01
SPLS	All 5 components	1.31E-02
	Selected variables from first component	9.86E-01
PMDCCA	All 5 components	9.89E-01
	Selected variables from first component	2.99E-02
RGCCA	All 5 components	8.73E-01
	Selected variables from first component	5.34E-02
MRF-IMD	filter	1.23E-02
	mixture	1.38E-02
	test	2.14E-02

Click here to access/download;Table;Table 5 - PANCAN clusters.xlsx

Table 5 Pan-cancer clustering annotations

Group	Cluster Name (Abbr.)
1	Basal-like Breast & UCEC (BRCA–UCEC)
2	Hepatobiliary Carcinomas (HBC)
3	Hypermutated/Immunogenic Tumors (HIM)
4	Non-basal Breast Cancer (Non-Basal BRCA)
5	Gastrointestinal Adenocarcinoma CIN (GA-CIN)
6	Endocrine Tumors (ENDO)
7	Squamous Cell Carcinomas (SCC)
8	Renal Epithelial Carcinomas (REC)

**TCGA Cohorts**
BRCA, UCEC
LIHC, CHOL
BLCA, SKCM
BRCA
COAD, STAD, ESCA
ACC, PCPG
HNSC, LUSC, ESCA, BLCA
KIRC, KIRP

Key Features
High genomic instability; frequent TP53 and BRCA1/2 alterations; dysregulated DNA repair and
Hepatic lineage tumors with altered metabolic programs and frequent TP53 mutations; cholangi[illegible]
Extremely high mutational burden; strong immune-infiltration signatures; enriched for PD-L1 exp
Luminal and HER2-positive subtypes; hormone-receptor signaling; PI3K/mTOR pathway activati
Marked chromosomal instability (CIN); Wnt/β-catenin and TGF-β pathway dysregulation; commo[illegible]
Hormone-secreting neoplasms of adrenal cortex and chromaffin cells; endocrine-axis gene dysr[illegible]
Squamous histology tumors; frequent TP53 mutations; activation of RTK-RAS and PI3K pathwa[illegible]
Clear-cell and papillary carcinomas; VHL/HIF pathway alterations; characteristic metabolic repro

cell-cycle pathways.
ocarcinoma-like epigenetic patterns.
oression and antigen-presentation machinery.
ion and endocrine-therapy response markers.
on APC and TP53 alterations.
egulation (e.g., steroidogenesis, catecholamine synthesis).
ys; strong epithelial-to-mesenchymal transition signatures.
gramming (glycolysis, lipid metabolism).

 Click here to access/download;Table;Table 6 - ADNI cox regression.xlsx

Table 6 Results from Cox regression model evaluating the association be
in 538 subjects, adjusted for age, sex, APOE ε4 status, baseline diagnos
(estimate = 0.201, P-value = 0.034), indicating higher IntNMF-derived coı

Characteristic	Coefficient	HR (95% CI)
**IntNMF-derived component**	0.201	1.222 (1.016,1.471)
**Age, years**	0.063	1.071 (1.037,1.093)
**Male**	0.178	1.195 (0.834,1.714)
**Baseline diagnosis**		
CN		1 [Reference]
MCI	0.423	1.527 (1.041,2.238)
***APOE* ε4 allele**	0.647	1.910 (1.472,2.478)
**MMSE**	-0.143	0.866 (0.772, 0.972)
**Education, years**	-0.054	0.947 (0.887,1.012)

etween IntNMF-derived component using MRF-IMD prioritized variable and disease prog
sis, MMSE, and education using ADNI dataset. Significant association was observed for
mponent increases risk.

***P*-value**
0.034
3.16×10^{-6}
0.332
0.03
1.13×10^{-6}
0.015
0.105

gression (CN to MCI/dementia, MCI to dementia)
IntNMF-derived component using MRF-IMD prioritized variables

Fig.1

Click here to access/download;Figure;Figure 1 - workflow.pdf

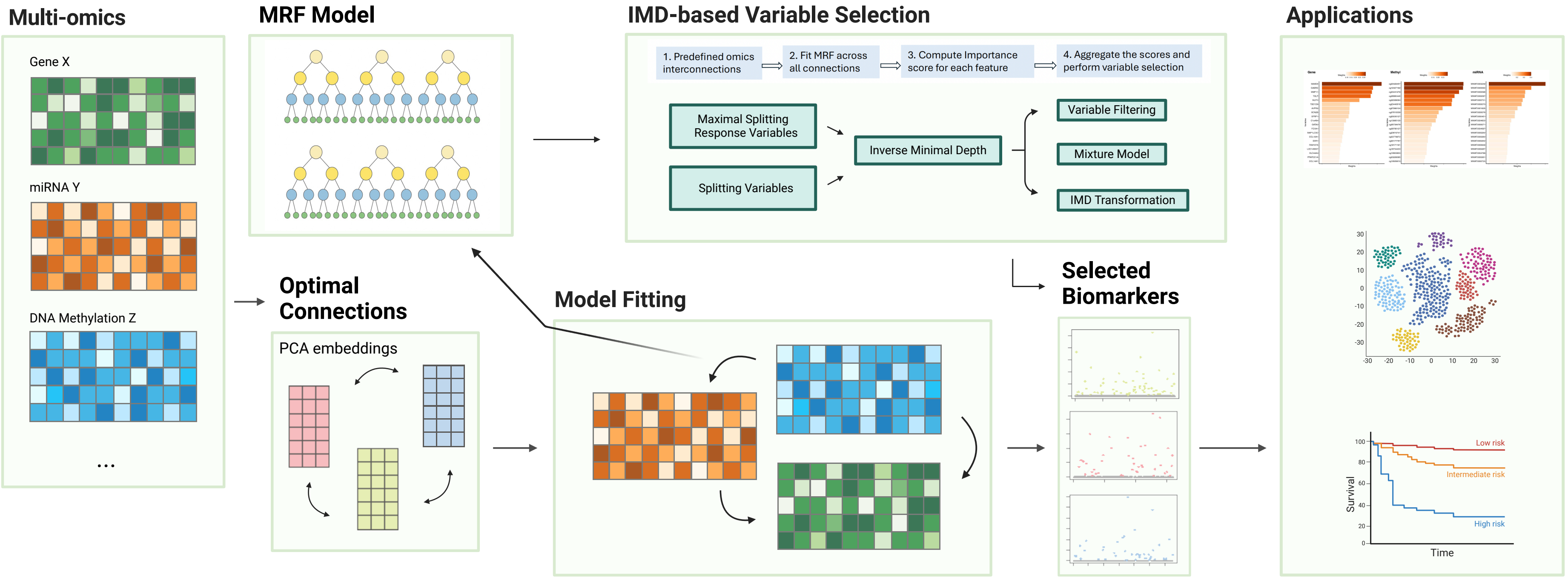

Fig.2

Click here to access/download;Figure;Figure 2 - simulation results.pdf

(a)

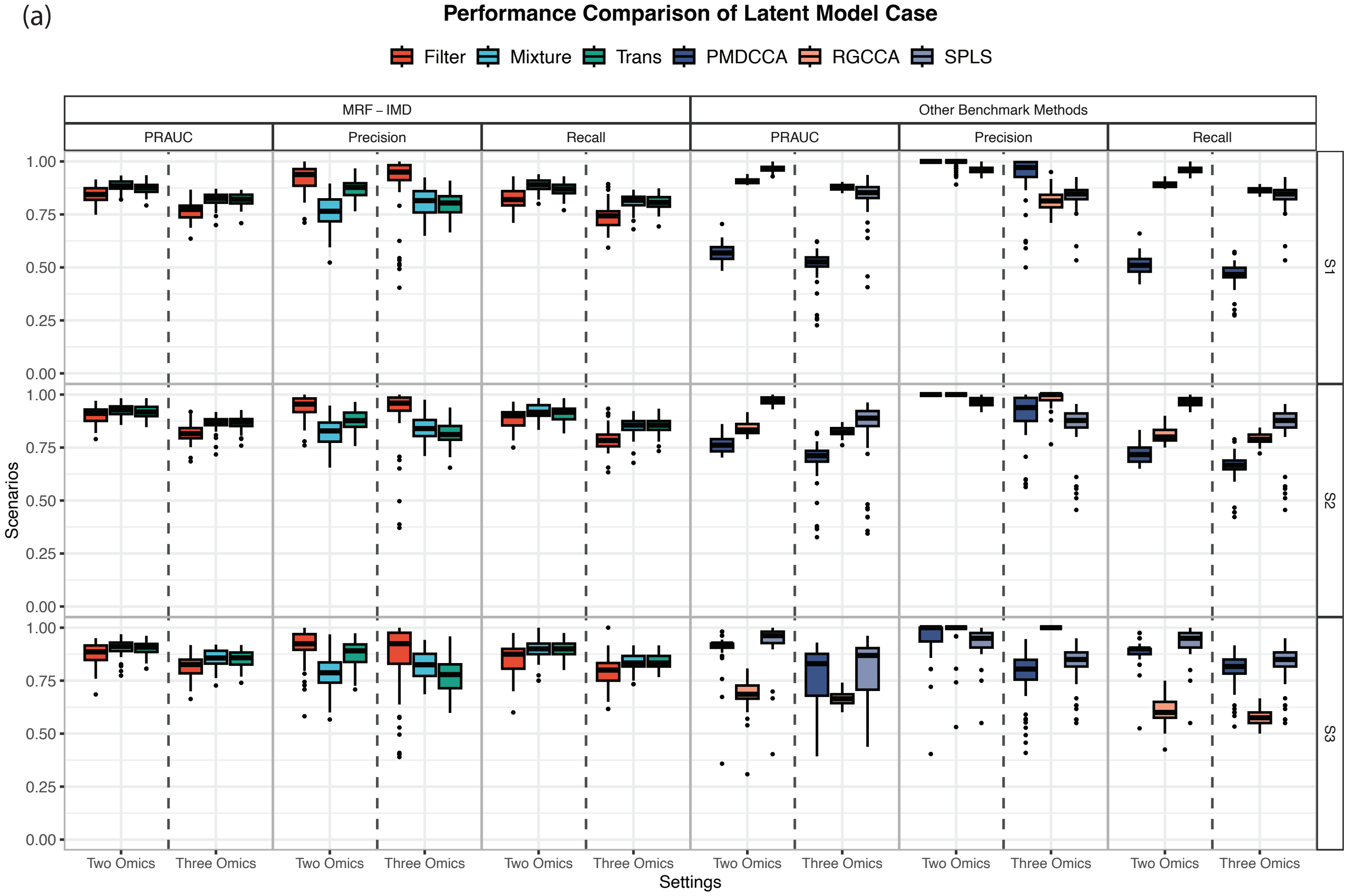

(b)

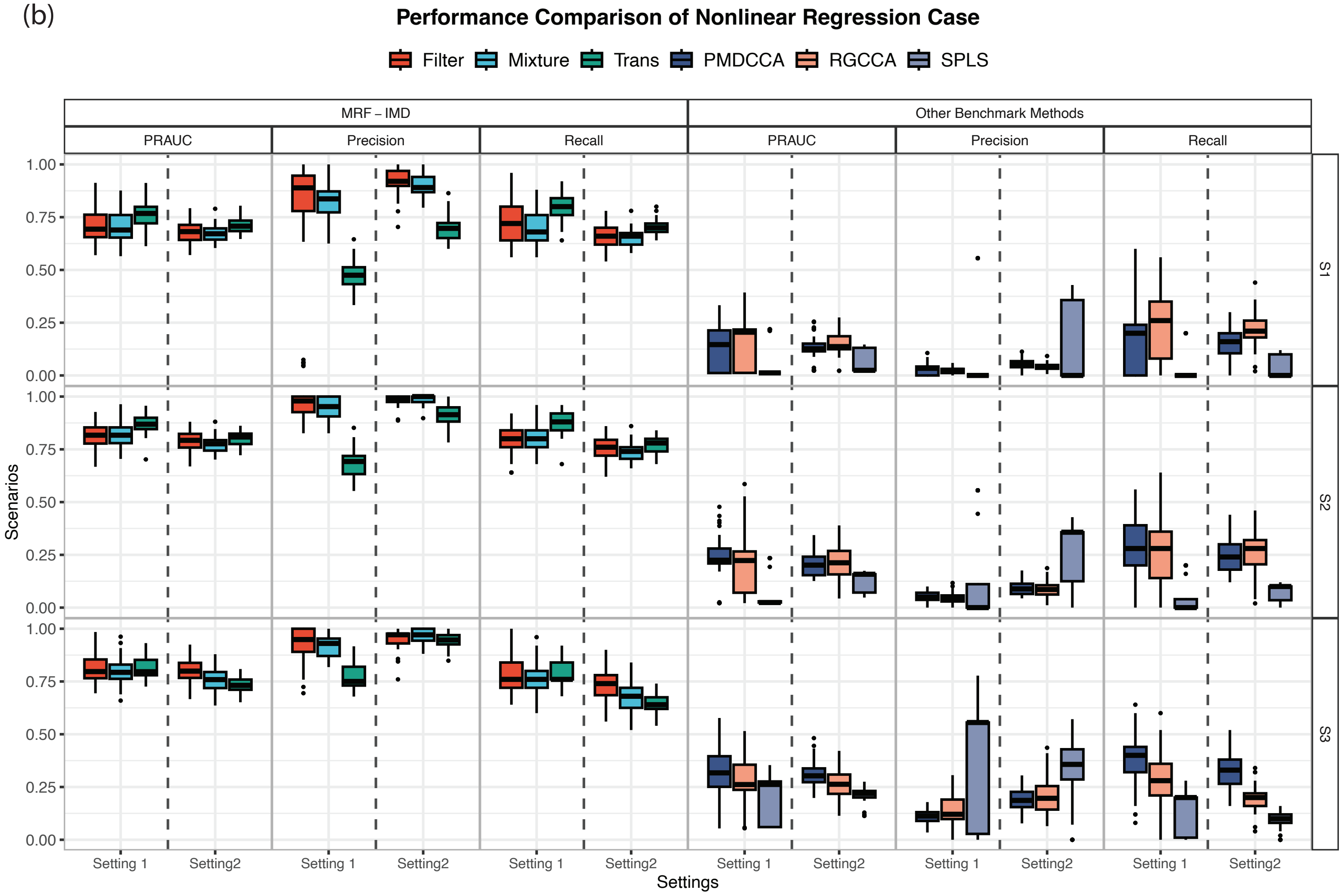

Fig.3

Click here to access/download;Figure;Figure 3 - BRCA COAD analysis.pdf

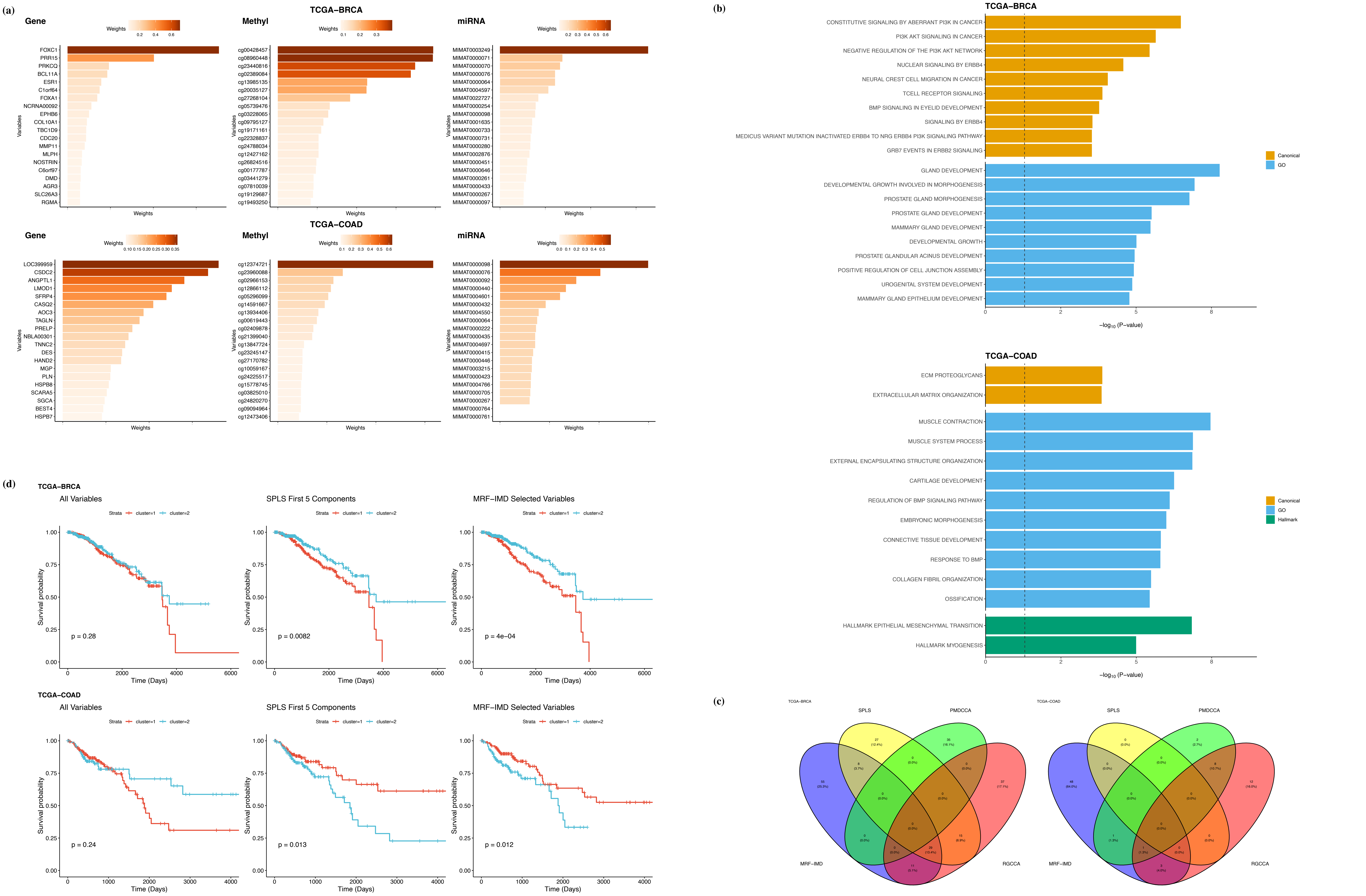

Fig.4
Click here to access/download;Figure;Figure 4 - PAN umap.pdf

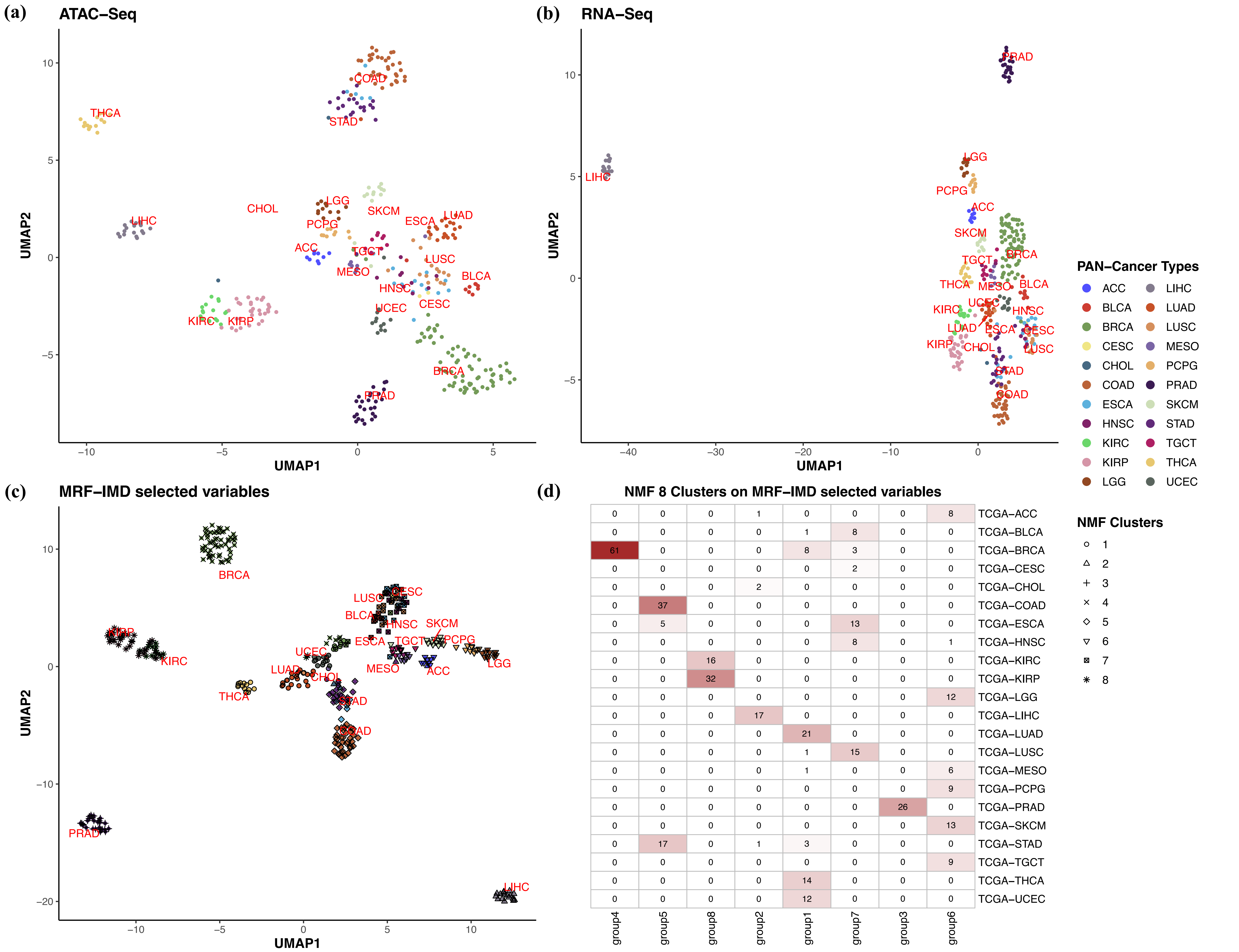

Fig.5

Click here to access/download;Figure;Figure 5 - ADNI results.pdf

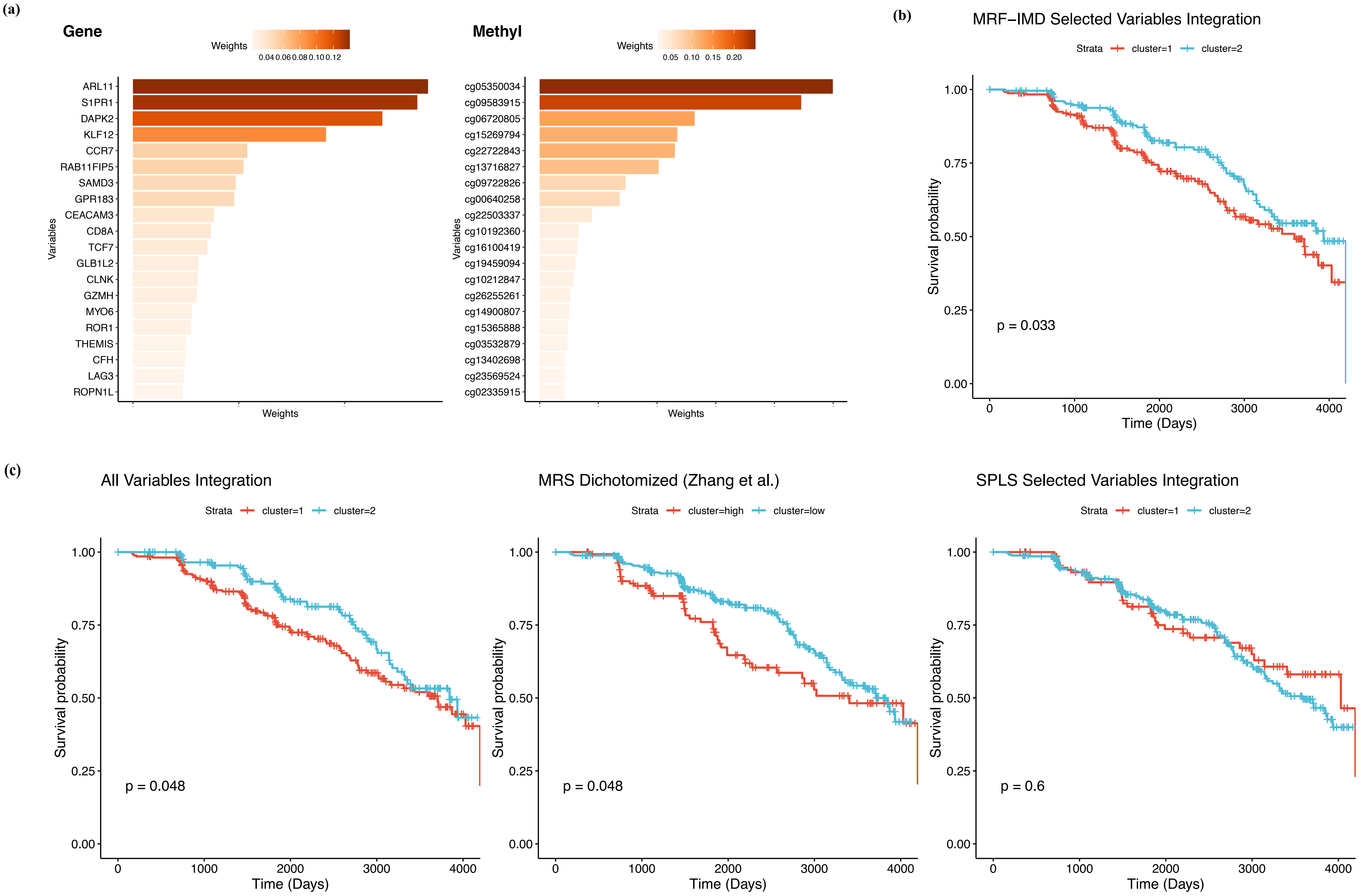

Supplementary Figures

Click here to access/download
Supplementary Material
Supplementary Figures.pdf

Supplementary Table 1

Click here to access/download
Supplementary Material
Supplementary Table 1 - pathway analysis BRCA COAD.xlsx

Supplementary Table 2

Click here to access/download
Supplementary Material
Supplementary Table 2 - ADNI pathway analysis.xlsx

Click here to access/download
Supplementary Material
Supplementary Table 3 - ADNI pathway analysis missMethyl.xlsx

Personal Cover

Click here to access/download;Personal Cover;CoverLetter_082025.pdf

SYLVESTER COMPREHENSIVE CANCER CENTER
UNIVERSITY OF MIAMI HEALTH SYSTEM
MILLER SCHOOL OF MEDICINE
UNIVERSITY OF MIAMI

Aug 25, 2025

Dear Dr. Nogoy and the GigaScience Editorial Team,

Thank you for the constructive reviews and guidance. We are pleased to resubmit our revised manuscript, *An Integrative Multi-Omics Random Forest Framework for Robust Biomarker Discovery* (GIGA-D-25-00021). We carefully addressed all points raised by Reviewers #1–#3 and strengthened the paper methodologically, biologically, and in terms of reproducibility.

Major revisions and additions

1. **Deeper biological interpretation and broader application**
   - Expanded interpretation for TCGA-BRCA and TCGA-COAD**,** linking top features to known drivers and pathways.
   - Added an **ADNI** use case (paired DNAm + expression) to demonstrate generalizability beyond oncology and improved progression stratification.
2. **Stability and sensitivity**
   - New **seed-wise stability** analyses for BRCA/COAD (model size distributions, overlap/Jaccard across runs), showing robust selection across random states.
3. **Head-to-head integrations on real data**
   - Added **SPLS, PMDCCA, and RGCCA** comparisons in BRCA/COAD with matched model sizes, side-by-side **pathway enrichment** and **prognostic** evaluations (IntNMF clustering + Kaplan–Meier/log-rank).
4. **Nonlinear and interaction benchmarks**
   - Added **nonlinear regression and interaction** simulations.
   - Included **univariate RF/GBM/XGBoost** as ranking baselines (aggregate feature importance across responses). MRF-IMD remains competitive in linear regimes and improves as nonlinearity/interactions grow.
5. **Pan-Cancer integration with quantitative clustering**
   - For **ATAC + RNA** across 22 TCGA cohorts, IntNMF on MRF-IMD features yields higher **Adjusted Rand Index (ARI)** versus SPLS-component, full-feature, and single-omics baselines.
6. **Method clarifications and interpretability aids**
   - Clarified the **unsupervised multivariate** setup (one block as a multivariate response to another; no clinical labels used in fitting).
   - Added guidance on when to use **filter**, **mixture**, or **transformation** IMD strategies.
   - Improved figure readability (larger fonts, corrected panel labels) and reported all dataset sizes, filtering thresholds, model sizes, and p-values.

Open science, data, and code

- **Code and documentation:**
  GitHub: https://github.com/TransBioInfoLab/multiRF-vs

  Vignette (RPubs): https://rpubs.com/noblegasss/multiRF-vs-vignette

  License: **GPL-3.0** (now added to the repository and stated in the manuscript).
  Environment details (R 4.4.2, OS) are included.

- **Data sources:**
  TCGA data via **UCSC Xena** and **TCGAbiolinks** (links and accession details now consolidated in **Data Availability**); ADNI data source listed.
  We will provide any additional figure-underlying data or TSV reformats requested at acceptance.
- **Reproducibility:**
  The repository includes scripts for all analyses and the vignette for step-by-step reproduction. (We will supply any additional metadata or persistent identifiers the journal requests at the curation stage.)

Declaration of Potential Competing Interests
The authors confirm that there are no conflicts of interest related to this work. All authors contributed significantly to the study and approved the final version of the manuscript. No AI tools were used to create or alter the research data or figures.

We appreciate the opportunity to revise this work for *GigaScience*. The revisions substantially improve clarity, benchmarking, and interpretability while aligning with the journal's emphasis on open, FAIR, and reproducible big-data science. We look forward to your evaluation.

With thanks and best regards,

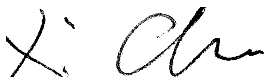

Xi Steven Chen
Professor of Biostatistics
Department of Public Health Sciences
University of Miami Miller School of Medicine
1120 NW 14th Street
Clinical Research Building, Suite #1044
Miami, FL 33136
Telephone: (305) 243-3081
steven.chen@miami.edu

Graphical Abstract

Click here to access/download;Graphical Abstract;graphical_abstract.pdf

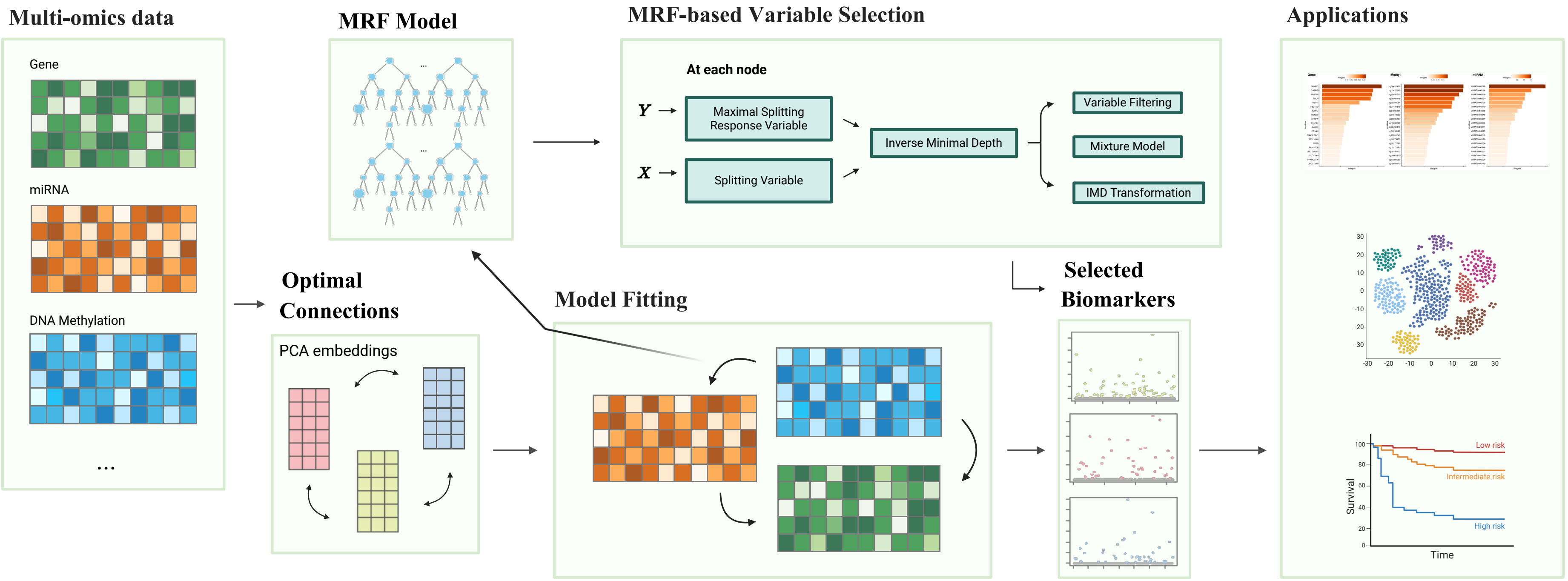

Supplement: giaf148_GIGA-D-25-00021_Revision_1 [file giaf148_giga-d-25-00021_revision_1.pdf]
