# Supplementary material for: An integrative multiomics random forest framework for robust biomarker discovery
Source: Gigascience. 2025 Dec 9;15:giaf148. doi: 10.1093/gigascience/giaf148 (PMC12821379; doi:10.1093/gigascience/giaf148)
Supplement: giaf148_Reviewer_1_Report_Original_Submission — Moran Chen -- 3/6/2025 [file giaf148_reviewer_1_report_original_submission.pdf]

**Reviewer Report**

**Title: An Integrative Multi-Omics Random Forest Framework for Robust Biomarker Discovery**

**Version: Original Submission** **Date:** 3/6/2025

**Reviewer name: Moran Chen**

**Reviewer Comments to Author:**

This manuscript presents a novel multivariate random forest (MRF) framework enhanced by the inverse minimal depth (IMD) metric for integrative multi-omics biomarker discovery. The authors clearly demonstrate the robustness and superiority of the proposed methods through comprehensive simulation studies and validation on TCGA datasets. The manuscript provides clear methodological explanations, offering valuable insights into its practical utility. I recommend accepting the manuscript after minor revisions.
Minor Concern:
1. Biological Interpretation Depth: While the authors identified biologically relevant biomarkers, the biological interpretations remain somewhat superficial. A deeper exploration of novel or less-known biomarkers in the context of disease mechanisms would strengthen the biological relevance of the findings.
2. Sensitivity Analysis of Randomness: The authors should conduct and discuss sensitivity analyses regarding different random states or random seeds to assess the stability of the method's results.
3. Comparison with Existing Methods on Real Data: While the simulation studies provide thorough benchmarking, the manuscript could enhance its practical value by including detailed comparisons with methods such as SPLS, PMDCCA, and SGCCA using the real-world TCGA datasets.
4. Applicability to Other Diseases: The authors primarily focus on cancer datasets. It is recommended to discuss potential applicability to other disease contexts, such as neurodegenerative or immunological diseases, to illustrate broader utility.
5. Improved Visualization: Some figures in the manuscript have font sizes that are too small, which might impair readability. It is recommended to enlarge the text labels, legends, and axis annotations to ensure that all information is clearly visible and accessible. In Figure 8, the use of sub-labels (such as a, b, c) is mentioned in the text, but these labels are not visible in the figure itself.

**Methods**

Are the methods appropriate to the aims of the study, are they well described, and are necessary controls included? Choose an item.

**Conclusions**

Are the conclusions adequately supported by the data shown? Choose an item.

**Reporting Standards**

Does the manuscript adhere to the journal's guidelines on minimum standards of reporting? Choose an item.

Choose an item.

**Statistics**

Are you able to assess all statistics in the manuscript, including the appropriateness of statistical tests used? Choose an item.

**Quality of Written English**

Please indicate the quality of language in the manuscript: Choose an item.

**Declaration of Competing Interests**

Please complete a declaration of competing interests, considering the following questions:

- Have you in the past five years received reimbursements, fees, funding, or salary from an organisation that may in any way gain or lose financially from the publication of this manuscript, either now or in the future?
- Do you hold any stocks or shares in an organisation that may in any way gain or lose financially from the publication of this manuscript, either now or in the future?
- Do you hold or are you currently applying for any patents relating to the content of the manuscript?
- Have you received reimbursements, fees, funding, or salary from an organization that holds or has applied for patents relating to the content of the manuscript?
- Do you have any other financial competing interests?
- Do you have any non-financial competing interests in relation to this paper?

If you can answer no to all of the above, write 'I declare that I have no competing interests' below. If your reply is yes to any, please give details below.

I declare that I have no competing interests.

I agree to the open peer review policy of the journal. I understand that my name will be included on my report to the authors and, if the manuscript is accepted for publication, my named report including any attachments I upload will be posted on the website along with the authors' responses. I agree for my report to be made available under an Open Access Creative Commons CC-BY license (http://creativecommons.org/licenses/by/4.0/). I understand that any comments which I do not wish to be included in my named report can be included as confidential comments to the editors, which will not be published.

Choose an item.

To further support our reviewers, we have joined with Publons, where you can gain additional credit to further highlight your hard work (see: https://publons.com/journal/530/gigascience). On publication of this paper, your review will be automatically added to Publons, you can then choose whether or not to claim your Publons credit. I understand this statement.

Yes Choose an item.
