# Supplementary material for: An integrative multiomics random forest framework for robust biomarker discovery
Source: Gigascience. 2025 Dec 9;15:giaf148. doi: 10.1093/gigascience/giaf148 (PMC12821379; doi:10.1093/gigascience/giaf148)
Supplement: giaf148_Revision_2_Report_Original_Submission — Yun-Juan Bao -- 3/16/2025 [file giaf148_revision_2_report_original_submission.pdf]

Reviewer Report

Title: An Integrative Multi-Omics Random Forest Framework for Robust Biomarker Discovery

Version: Original Submission **Date:** 3/16/2025

Reviewer name: Yun-Juan Bao

Reviewer Comments to Author:

The article presents an Integrative Multi-Omics Random Forest Framework for Robust Biomarker Discovery. It addresses the challenge of extracting key shared biomarkers from multiple omics data types by introducing a multivariate random forest-based approach enhanced by an inverse minimal depth metric.

I have some concerns and comments below:

1. The new algorithm described in the study selected omics variables by assigning response variable to decision tree nodes. How the response variables relate to biological responses/outcomes? From the authors' description, it seems that the selected omics variables using the IMD are almighty, i.e., they can predict anything needed, such as prognosis, cancer types, and et al. Actually, the usual logic to select omics variables to predict prognosis is to evaluate the association between omics variables and survival time.
2. Following the discussion in 1, what is the biological meaning to extract shared biomarkers from multiple data layers? While it is straightforward to think that the shared biomarkers between multiple data layers or data types may induce the same biological responses, the unique biomarkers also matter depending on what biological responses we care.
3. The Introduction section is not sufficient. The biological significance and technical details of "extract shared biomarkers from multiple data layers" need to be explained in more details.
4. It is advised to provide some examples of the statement in the Introduction: "may fail to capture nonlinear interactions" of the current methods (sPLS, CCA).
5. It is also advised to explain and illustrate how the new method proposed in this study addressed the challenge of traditional methods for capturing nonlinear relationships. Ablation study could be one of the choices.
6. The authors showed that their new approach "uncovered known cancer biological relevant pathways". How about the functional enrichment of genes selected from traditional methods, such as sPLS, CCA?
7. The authors showed that the selected RNA-seq and ATAC-seq features using the new approach are able to capture the distinction between different cancer types (Figure 8). It is suggested to quantitatively evaluate this capability using metrics of recall, precision, and et al. to calculate how many samples are corrected classified and how many are mis-classified in comparison with other methods.
8. It is advised to re-find the Discussion. In what scenario their new method can be applied? What biological insights can be obtained and what can be missed by the new method?
9. The authors did not provide sufficient details about the datasets they used in the section Method. How many samples in TCGA? How many features did they use? How many features left after filtering?

10. Although the performance of the new approach showed some kind of superior in comparison with other methods, the authors only used the currently known databases. It is advised to apply their approach to additional testing datasets or real-world datasets to increase the confidence of the conclusion of this study. It is also observed that the performance of sPLS is better than others in some cases (Figure 4).
11. It is suggested to re-fine the figures. The labels and legends are too tiny to be seen.
12. There is no sub-figure labels a,b,c,d,e,f in Figure 8. The positions of sub-figure labels in Figure 3, Figure 4, Figure 5, Figure 7 are not correct.

Methods

Are the methods appropriate to the aims of the study, are they well described, and are necessary controls included? Choose an item.

Conclusions

Are the conclusions adequately supported by the data shown? Choose an item.

Reporting Standards

Does the manuscript adhere to the journal's guidelines on minimum standards of reporting? Choose an item.

Choose an item.

Statistics

Are you able to assess all statistics in the manuscript, including the appropriateness of statistical tests used? Choose an item.

Quality of Written English

Please indicate the quality of language in the manuscript: Choose an item.

Declaration of Competing Interests

Please complete a declaration of competing interests, considering the following questions:

- Have you in the past five years received reimbursements, fees, funding, or salary from an organisation that may in any way gain or lose financially from the publication of this manuscript, either now or in the future?
- Do you hold any stocks or shares in an organisation that may in any way gain or lose financially from the publication of this manuscript, either now or in the future?

- Do you hold or are you currently applying for any patents relating to the content of the manuscript?
- Have you received reimbursements, fees, funding, or salary from an organization that holds or has applied for patents relating to the content of the manuscript?
- Do you have any other financial competing interests?
- Do you have any non-financial competing interests in relation to this paper?

If you can answer no to all of the above, write 'I declare that I have no competing interests' below. If your reply is yes to any, please give details below.

I declare that I have no competing interests

I agree to the open peer review policy of the journal. I understand that my name will be included on my report to the authors and, if the manuscript is accepted for publication, my named report including any attachments I upload will be posted on the website along with the authors' responses. I agree for my report to be made available under an Open Access Creative Commons CC-BY license (http://creativecommons.org/licenses/by/4.0/). I understand that any comments which I do not wish to be included in my named report can be included as confidential comments to the editors, which will not be published.

Choose an item.

To further support our reviewers, we have joined with Publons, where you can gain additional credit to further highlight your hard work (see: https://publons.com/journal/530/gigascience). On publication of this paper, your review will be automatically added to Publons, you can then choose whether or not to claim your Publons credit. I understand this statement.

Yes Choose an item.
